# Checklist of vascular plants of the Department of Ñeembucú, Paraguay

**DOI:** 10.3897/phytokeys.9.2279

**Published:** 2012-01-30

**Authors:** Juana De Egea, Maria Peña-Chocarro, Cristina Espada, Sandra Knapp

**Affiliations:** 1 Department of Botany, The Natural History Museum, Cromwell Road, London SW7 5BD, United Kingdom; 2 Wildlife Conservation Society Paraguay, Capitán Benitez Vera 610, Asunción, Paraguay

**Keywords:** conservation, humid Chaco, Ñeembucú, new distribution records, Paraguay, wetlands

## Abstract

The Department of Ñeembucú is one of the least well-documented areas of eastern Paraguay, and the flora is composed of a mixture of forest and Chaco elements. Regions like Ñeembucú are often considered of lower diversity and interest that more forested regions; this results from both actual species richness figures and from under-collecting due to perception as uninteresting. We present here a checklist of the vascular plants of Ñeembucú, which includes 676 taxa (including infraspecific taxa and collections identified only to genus) in 100 families and 374 genera. Four hundred and thirty nine (439) of these are new records for Ñeembucú and of these, 4 are new published records for Paraguay. Synonyms, distribution details within Paraguay and a voucher specimen or literature record are provided for each taxon, and a brief analysis of the diversity and importance of the flora is presented.

## Introduction

Paraguay is a land-locked country between 19° and 28° south latitude and 54° and 63° west longitude at the heart of the South American continent, and lies entirely within the Río de la Plata drainage system, second only in size to the Amazon basin. The country is divided by the Río Paraguay into the Oriental, or eastern, region, also known as the Paraná region; and the Occidental, or western, region, also known as the Chaco, part of the Gran Chaco Americano that is shared by Argentina, Bolivia and Paraguay. Paraguay is divided into 17 departments, 14 of which are to the east of the Río Paraguay.

**Figure 1.**
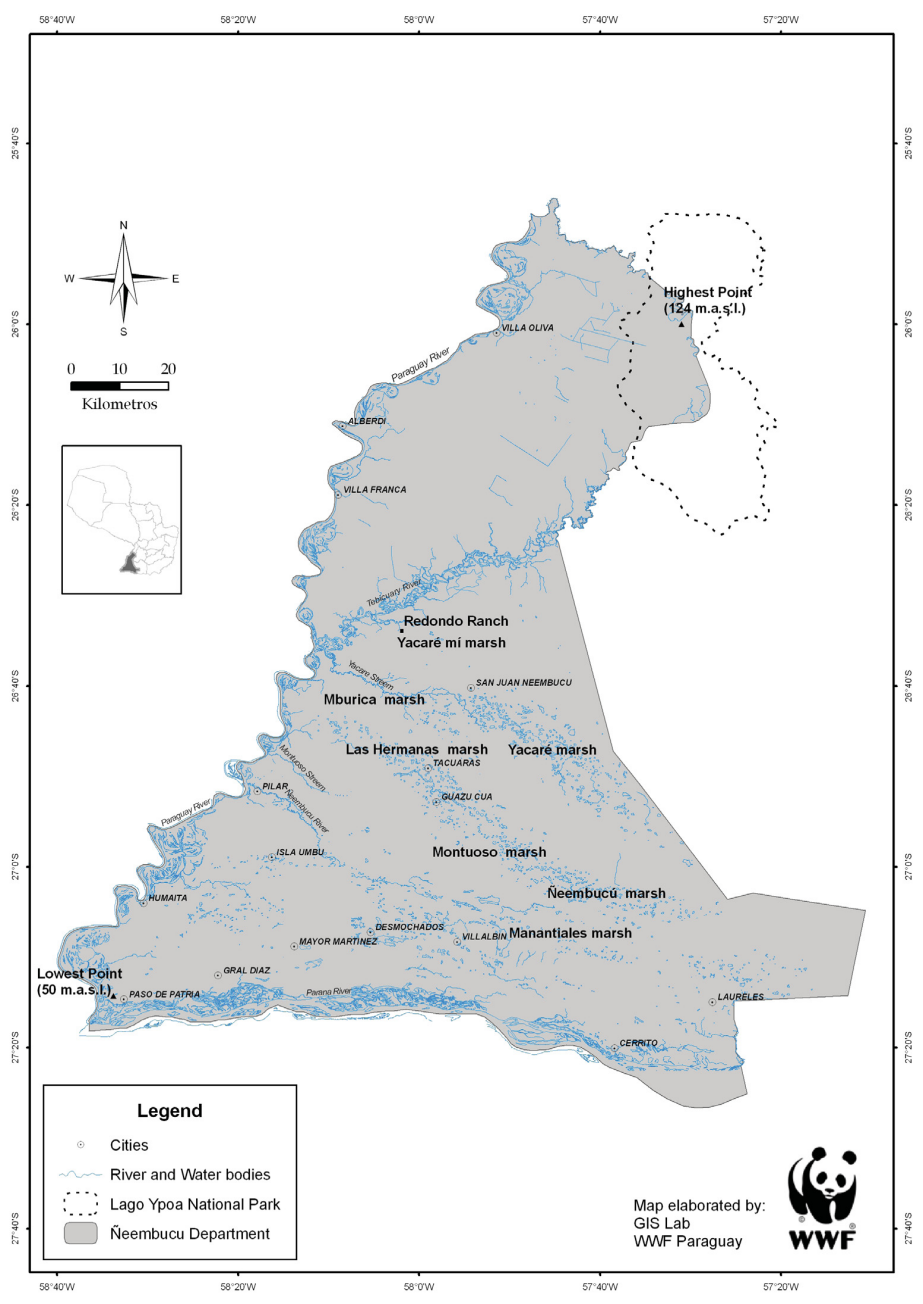
Map of the wetlands of eastern Paraguay; Estancia Redondo (Redondo Ranch) is located south of the Río Tebicuary at approximately 26°30’ S latitude. The complex set of marshy wetlands is the dominant feature of the angle between the Río Paraguay and the Río Paraná.

The department of Ñeembucú is the southernmost tip of Paraguay and is located in the south-western corner of the Oriental region, between 57°35' – 57°55' W latitude and 25°50' – 27°19' S longitude (Fig. 1). Ñeembucú covers an approximate area of 12,147 square kilometres (SINASIP 2009). The average annual temperature is between 22° and 23°C, and average annual rainfall is around 1500 mm (Acevedo et al. 1990). Most of this area is made up of a plain comprised of alluvial sediments from the Quaternary period, with hydromorphic soils formed from the transport of sediment by rivers and streams, and dominated by shallow hydromorphic and alluvial gley types, planosols or gley humic acids with high organic matter content (Tortorelli 1966).

Ñeembucú is partially surrounded by both the Paraguay and Parana rivers and has a number of extensive wetlands, the largest of these being the Ñeembucú and the Cambá marshes. The area is low elevation and relatively flat; altitudinal range is between 50 and 124 metres above sea level. As a result of these particular topographic and hydrographic features, Ñeembucú is usually described as one extensive floodplain; over 85% of this area is wetlands (Fogel 2000), where water is the primary factor that regulates seasonal variation and biological and ecological characteristics of the vegetation composition. The wetlands of Ñeembucú are the largest in the Oriental region and are very rich in terms of alpha and beta diversity, particularly when it comes to species of flora and fauna closely linked to water (Vogt and Mereles 2005).

The wetlands of the Oriental region were studied by Mereles and Aquino-Shuster (1990), Mereles et al. (1992), and Mereles (1993, 2004). Vegetation of the Ñeembucú stream basin, which can be considered as representative of the department, was described in detail by Vogt and Mereles (2005), who identified two principal vegetation types; 1) those subject to permanent flooding, and 2) those linked to periodic flooding.

Vegetation that remains flooded throughout the year can be characterised as lotic or lentic. Lotic environments are typical of the extensive network of flowing waterways distributed throughout the department; common species include *Nymphoides indica* (L.) Kuntze, *Myriophyllum aquaticum* (Vell.) Verdc., *Eichhornia crassipes* (Mart.) Solms, *Sagittaria montevidensis* Cham. & Schltdl., and several species of *Persicaria*. Lentic environments occur in areas where water stagnates and does not flow, or does so very poorly, as a result of geomorphological conditions. These include the lagoons and estuaries or areas of permanent inundation which harbour different types of plant communities; free-floating vegetation (with species such as *Eichhornia crassipes*, *Eichhornia azurea* (Sw.) Kunth, *Pontederia rotundifolia* L.f., *Pistia stratiotes* L., *Azolla* spp., *Salvinia* spp.); half-submerged vegetation with plants that are submerged but not rooted and whose leaves and flowers are emergent (*Myriophyllum aquaticum* and *Utricularia* spp.); and half-submerged, rooted vegetation, with vegetative and reproductive parts of plants primarily above the water surface (*Nymphaea* spp., *Nymphoides indica*, *Eleocharis* spp., *Typha domingensis* Pers., *Thalia geniculata* L., *Rhynchospora corymbosa* (L.) Britton, *Schoenoplectus californicus* and *Cyperus giganteus* Vahl). In extreme cases masses of floating vegetation in still waters become impounded, forming floating islands of aquatic species associations which go through various stages of evolution, depending on the amount of substrate they retain. Thus, in the more advanced stages, rooted species can be found in a thick floating substrate that resembles soil.

Vegetation subject to periodic flooding includes hydromorphic savannahs and hygrophilous (riparian) forests. The vascular plant species composition of hydromorphic savannahs varies according to soil type. Palm savannahs are found on soils with a high content of clay (up to 30%) and salts, especially chlorides; the dominant species is the palm *Copernicia alba* Morong, sometimes associated with scattered trees of *Aspidosperma quebracho-blanco* Schltdl. and *Astronium balansae* Engl., while the understory is dominated by marshy species; common plants include various grasses and sedges, *Ludwigia*, *Persicaria* and *Ruellia*. In the palaeobasins of the Río Parana, where the soils are sandy, the landscape is dominated by grasslands of *Elionurus muticus* and *Schizachyrium* spp., sometimes with the presence of scattered woody species such as *Cecropia pachystachya* Trécul and *Enterolobium contortisiliquum* (Vell.) Morong.

Riparian forests are directly or indirectly associated with water, as they are subjected to periodic flooding or waterlogging at certain times of year; these can be found along the edges of rivers, streams and lakes, or forming islands of forests surrounded by wetlands and floodplains. The edges of these forests include hygrophilous species such as *Croton urucurana* Baill., *Microlobius foetidus* (Jacq.) M.Sousa & G.Andrade subsp. *paraguensis* (Benth.) M.Sousa & G.Andrade, *Sapium haematospermum* Müll.Arg., *Sebastiania brasiliensis* Spreng., *Guadua* sp., *Chusquea ramossisima* Lindm., *Inga vera* Willd. and *Enterolobium contortisiliquum*. The understory is dominated by *Bromelia balansae* Mez and there are a number of epiphytic ferns and flowering plants. As soil depth increases, the forests change from purely riparian to humid semi-deciduous, characterized by 2 to 4 layers of vegetation and a maximum canopy height of about 25 m. The dominant species in the tree layer are *Handroanthus heptaphyllus* (Vell.) Mattos, *Peltophorum dubium* (Spreng.) Taub., *Syagrus romanzoffiana* (Cham.) Glassman, *Gleditsia amorphoides* (Griseb.) Taub., *Luehea divaricata* Mart., *Holocalyx balansae* Micheli and species of figs and Lauraceae (Acevedo et al. 1990; Vogt and Mereles 2005). These patches of forest are similar to more extensive interior Atlantic forest found in the Oriental region, now very limited in extent (Huang et al. 2009).

Although the wetlands and forests of Ñeembucú comprise physical features and species composition that are similar to the humid Chaco, such that they are regarded as intrusions of Chaco vegetation in the Oriental region (Mereles 1993, 2004), the sandy layer which emerges in many parts of the department results in the development of more diverse and transitional habitats; consequently, the vegetation Ñeembucú is a mosaic of species typical of both the Eastern and Western (Chaco) regions (Mereles 2004). Spichiger et al. (1995) described the tree flora of Ñeembucú as “a most interesting boundary zone between the Chaquenian, the Palm-Savannas, the Residual Pleistocenic Dry Seasonal, and the Paranean Floras”, i.e., an ecotone between dry forests, wet forests and savannah.

The earliest collections specifically located in the Ñeembucú area are those of the French botanist Benedict (Benjamin) Balansa, who collected in Paraguay in the late 19^th^ century. In the early half of the 20^th^ century Teodoro Rojas and August Gustavo Schulz, both German Argentine collectors, made significant collections in the region, but the bulk of specimens from the 20^th^ century were made by the Swiss botanists Luciano Bernardi (1978, 1980) and Rodolphe Spichiger (1990s), the German/Argentine Teodoro Meyer (mid-20^th^ century), and Shirley Keel and Lucio Spinzi (1988) working for the Nature Conservancy.

More recent collecting efforts have been carried out consistently but not intensively by the Paraguayan botanists Christian Vogt in 2004, Fatima Mereles in 2009, and our team between the years 2004-2006 as part of a Darwin Initiative project (see http://darwin.defra.gov.uk/). Our collections were concentrated in a single locality, Estancia Redondo (see Fig. 1), but of the 419 specimens collected, 182 were new records for the Department; this degree of novelty prompted us to compile this checklist. Estancia Redondo, with an extent of approximately 5,959 hectares, represents a well-preserved sample of the Ñeembucú wetlands and forests, and has been declared Important Bird Area (IBA) No. 29 for Paraguay (Guyra Paraguay 2008).

The Department of Ñeembucú lacks large protected areas for the conservation of its wetlands and forests; only 361.9 square kilometres are under protection, the equivalent to only 3% of its area, which makes up part of the Ypoá National Park, shared with the Central and Paraguarí departments (SINASIP 2009) and which was designated a Ramsar Site (Number 728) in 1995. It is also worth mentioning that the Ñeembucú marsh, with an area of 800 square kilometres, was proposed by Acevedo et al. (1990) as a priority area for conservation in this department; however, it was never officially declared as a reserve. Parallel to the conservation efforts at the political level, the expanding agricultural frontier, the deforestation and the degradation of the environment which encourages the colonization of invasive alien species, all represent serious threats to the wetlands and forests of Ñeembucú. The drainage of wetlands with the purpose of gaining areas of productive land and intensive cattle ranching through the implementation of unsustainable practices that require the introduction of non-native grasses, intensive grazing, introduction of weeds and the uncontrolled burning of pastures are all activities that have caused the greatest impact in recent years (Vogt and Mereles 2005). Consequently, the shortage of protected areas, the changes in land use and the lack of alternatives for sustainable production compatible with conservation threaten biodiversity and the complex ecological systems of the Ñeembucú wetlands.

Despite harbouring significant biodiversity, Ñeembucú has been one of the least explored areas in Paraguay and few collections have been made. Zuloaga et al. (2008) record 204 taxa for Ñeembucú in the published checklist of the flora of the Southern Cone; the updated on-line version of the checklist (Flora del Conosur, http://www2.darwin.edu.ar/Proyectos/FloraArgentina/FA.asp, updated January 2009) records 15 more; total recorded vascular plant diversity for Ñeembucú was 237 taxa (including those genera recorded as sp. here) prior to our studies. Our collecting in the region (see below) resulted in significant novelty, and the checklist presented here is a first step in documenting the relatively understudied plant diversity of Ñeembucú.

## Materials and methods

In assembling the checklist we revised and re-determined all historical and recent collections from Ñeembucú held in the Natural History Museum (BM) and the Facultad de Ciencias Químicas of the Universidad Nacional de Asunción (FCQ). We also reviewed the recent monographic literature for collection records from Ñeembucú, and where we have not seen a specimen of a taxon cited in these sources we include the relevant literature citation, including the page. We also include identified specimens from Ñeembucú cited in TROPICOS (http://www.tropicos.org/) and in the database of the Botanical Garden of Geneva (http://www.ville-ge.ch/musinfo/bd/cjb/fdp/). For those records for which we have examined a specimen we only cite a single voucher, other specimens seen and additional literature citations are not included here.

Flowering plant family circumscription follows APG III (Chase et al. 2009) and the pteridophyte classification follows Smith et al. (2006) as modified by Christenhusz et al. (2011). Families are sorted alphabetically and genera are sorted alphabetically within families. For each taxon found in the Department, we present the accepted name, synonyms, habit, introduced status (all species are native except where indicated), general distribution in Paraguay and one voucher specimen (if available). We use infraspecific names only in the cases where taxa other than or additional to the typical occur. Those taxa cited to the level of genus only may represent new species or new records for the department, but we were unable to identify them to the specific level. Synonymy follows Zuloaga et al. (2008) and TROPICOS (http://www.tropicos.org/) except for Myrtaceae, where we followed the *World Checklist of Myrtaceae* (Govaerts et al. 2008) and for *Inga* (Fabaceae) where we followed Pennington (1997). For some very common weedy species with extensive worldwide synonymy we have not cited all synonyms; these can be found in Zuloaga et al. (2008) and TROPICOS (http://www.tropicos.org/). We also cite commonly encountered illegitimate names and names not validly published in the synonymy, with indications as to their status. Herbarium acronyms used in the list follow *Index Herbariorum* (Holmgren et al. 1990, available on-line at http://sciweb.nybg.org/science2/IndexHerbariorum.asp).

New reports of taxa for the department of Ñeembucú are marked with an arrowhead (►), those taxa not cited in Zuloaga et al. (2008) or the on-line updated version of the Southern Cone checklist (Flora del Conosur 2009) for Ñeembucú but recorded in other publications or databases (the URL or bibliographic reference of each source given with the voucher specimen cited) are marked with an asterisk (*) and those that have not been cited previously as occurring in Paraguay are indicated with a circle (●).

## Data resources

The occurrence data underpinning the analysis have been uploaded as a Darwin Core Archive (DwC-A), to the Global Biodiversity Information Facility (GBIF) via the Pensoft Data Hosting Center at the GBIF’s Integrated Publishing Toolkit (IPT) (http://ipt.pensoft.net/ipt/resource.do?r=neembucu).

In addition, three Excel spreadsheets containing the taxon checklist, the habitat description and the departmental distribution in Paraguay, generated from the original MS Word manuscript file (Remsen et al. 2012), are published as Appendix to the present paper and are available under doi: 10.3897/phytokeys.9.2279.app.

## Results and discussion

Our checklist for Ñeembucú records 676 taxa in 374 genera in 100 families (8 families of ferns and fern allies). A total of 439 taxa not previously recorded for the department are included here; 254 of these are new additions for the department resulting from our studies (marked ►), 171 (marked *) have been cited in other sources but not in Zuloaga et al. (2008), and 4 taxa (marked ●) have not been cited previously as occurring in Paraguay. In total this represents a more than tripling (676 versus 254, >300%) in vascular plant records for Ñeembucú over the most recent complete published compilation of distributional records for the department (Zuloaga et al. 2008, including updates online in Flora del Conosur 2009).

Nine families have more than 20 taxa in Ñeembucú (Table 1). As one might expect for an area that is in part dominated by wetlands and grasslands, Poaceae and Cyperaceae feature prominently. Legumes (Fabaceae), composites (Asteraceae) and Malvaceae are the most taxon-rich families of eudicots and are represented by a large number of herbaceous taxa common to open habitats in the region. Herbs (including aquatic herbs) are the commonest habit class (Table 2) and are the most common new records for Ñeembucú recorded here. We suggest this is a result of the emphasis on trees in general collections for evaluation of habitats for conservation priorities (with the exception of Vogt and Mereles 2005). Future collecting in Ñeembucú should concentrate on all habit categories as all are represented by significant novelty with half or more than half of the records being new to the department; herbaceous plants, including epiphytes and aquatics will be particularly promising (e.g., see Table 2).

**Table 1.** Taxon-rich families in the flora of Ñeembucú.

**Family**	**Number of taxa**
Poaceae	59
Fabaceae	50
Asteraceae	37
Cyperaceae	36
Malvaceae	28
Euphorbiaceae	24
Solanaceae	24
Verbenaceae	22
Rubiaceae	20
Myrtaceae	19

**Table 2.** Habit of vascular plants in Ñeembucú; numbers in parentheses are new records for the department in each habit category.

**Habit**	**Number of taxa (new records., % new to Ñeembucú)**
Tree	70 (48, 66% new)
Shrubs or small trees	103 (52, 50% new)
Herbs or subshrubs	383 (269, 70% new)
Climbers	53 (32, 60% new)
Epiphytes (including parasites)	26 (26, 100% new)
Aquatic herbs	37 (28, 76% new)
Palms	4 (2, 50% new)

Priority setting in global biodiversity conservation is one way to target efforts to safeguard habitats, species and genes, all tasks required of countries signatory to the Convention on Biological Diversity (CBD). Approaches to priority setting are quite variable –ranging from just personal interest, to taxon based approaches such as the Important Bird Areas (IBAs; ) and Endemic Bird Areas (EBAs; Stattersfield et al. 1998) or the Centres of Plant Diversity (Davis et al. 1997), to “hotspot” approaches (Myers et al. 2000), “megadiversity-country” approaches (Mittermeier and Werner 1990), ecoregional priority setting techniques (Dinerstein et al. 1995) and complementarity-based approaches (Margules et al. 2002). Species richness and endemism have been considered important by some, while others have focused more on the conservation of representative habitats or ecosystems. It is clear that multiple indices are more appropriate than a one-size-fits-all approach (Orme et al. 2005).

The mosaic of wetland and shrubland habitats of Ñeembucú do not feature as a “hotspot” in analyses using the combined richness and endemism criteria of Myers et al. (2000), nor is the area designated a priority using the ecosystems approach of Dinerstein et al. (1995). The status of the adjacent humid Chaco in the latter publication is “locally outstanding”; does this mean then that these regions are thus not important for conservation at a regional or global scale? Although part of the Oriental region of Paraguay, Ñeembucú combines many different floristic elements (Mereles 1993, 2004), making it of interest for analysis of relationships between habitat types in southern South America. The wetlands of Ñeembucú can be seen as the southernmost extension of the Pantanal, as they are connected along the drainage basin of the Río Paraguay (see Swarts 2000). Drainage of land for soybean production has not yet been an important factor in Ñeembucú, but the importance of ranching cannot be underestimated in the region. Because the various productive activities in the region involve private ownership of large tracts of land, there are many different players in the conservation equation in the region. On the one hand this could be seen as a difficulty, involving many sectors can be complicated and logistically time-consuming, but on the other hand, the broad cross-section of society involved in the region is a real opportunity.

Knowledge of the organisms present in a region is an important step for future conservation action. Although our preliminary work has greatly increased knowledge of the floristic diversity of Ñeembucú, it is clear that knowledge of the flora of the department is far from complete and future collecting and documentation will certainly add to the records presented here. With increasing habitat alteration in eastern Paraguay (see Huang et al. 2009) there is an urgent need to improve knowledge on the biodiversity of this understudied region of Paraguay, and concomitantly with these efforts, design initiatives for the strengthening protection and sustainable management of the unique and diverse wetlands and forests of Ñeembucú. Checklists of floristic diversity are a basic first step in this effort.

## Checklist

### Lycopods

#### SELAGINELLACEAE

##### *Selaginella* sp.

Herb.

Voucher: *C. Vogt 149* (CTES, FCQ).

### Ferns

#### ANEMIACEAE

##### ►*Anemia tomentosa* (Savigny) Sw. var. *anthrisifolia* (Schrad.) Mickel, Iowa State J. Sci. 36(4): 424. 1962.

Syn.: *Anemia anthriscifolia* Schrad.; *Anemia fulva* auct. non (Cav.) Sw.

Herb.

Departmental distribution in Paraguay: Alto Paraguay, Amambay, Caazapá, Canindeyú, Central, Ñeembucú.

Voucher: *J. De Egea et al. 706* (BM, FCQ, MO).

#### AZOLLACEAE

##### ►*Azolla filiculoides* Lam., Encycl. (Lamarck) 1(1): 343. 1783.

Syn.: *Azolla arbuscula* Desv.; *Azolla bonariensis* Bertol.; *Azolla caroliniana* Willd.; *Azolla magellanica* Willd.; *Azolla microphylla* Kaulf.; *Azolla squamosa* Molina

Aquatic herb.

Departmental distribution in Paraguay: Alto Paraná, Alto Paraguay, Cordillera, Ñeembucú, Presidente Hayes.

Voucher: *M.A. Walter 39* (BM).

##### *Azolla* sp.

Aquatic herb.

Voucher: *M. Peña-Chocarro et al. 2363* (BM, FCQ).

#### MARSILEACEAE

##### ►*Marsilea ancylopoda* A.Braun, Monatsber. Konigl. Preuss. Akad. Wiss. Berlin 1863: 434. 1864.

Syn.: *Marsilea berteroi* A.Braun; *Marsilea concinna* Baker; *Marsilea ernestii* A.Braun; *Marsilea hickenii* Herter; *Marsilea mexicana* A.Braun; *Zalusianskia ancyclopoda* (A.Braun) Kuntze; *Zalusianskia berteroi* (A.Braun) Kuntze; *Zalusianskia concinna* (A.Braun) Kuntze; *Zalusianskia ernestii* (A.Braun) Kuntze; *Zalusianskia mexicana* (A.Braun) Kuntze

Aquatic herb.

Departmental distribution in Paraguay: Alto Paraguay, Central, Ñeembucú.

Voucher: *M. Peña-Chocarro et al. 2346* (FCQ).

#### POLYPODIACEAE

##### ►*Microgramma vacciniifolia* (Langsd. & Fisch.) Copel., Gen. Fil. [Copeland] 185. 1947.

Syn.: *Craspedaria cordifolia* Fée; *Craspedaria vacciniifolia* (Langsd. & Fisch.) Link; *Lepicystis vacciniifolia* (Langsd. & Fisch.) Diels; *Polypodium lycopodioides* L.; *Polypodium vacciniifolium* Langsd. & Fisch.

Epiphyte.

Departmental distribution in Paraguay: Alto Paraguay, Caazapá, Canindeyú, Concepción, Ñeembucú, Paraguarí, Presidente Hayes.

Voucher: *M. Peña-Chocarro et al. 2219* (BM, FCQ).

#### PTERIDACEAE

##### ►*Cheilanthes tweediana* Hook., Sp. Fil. 2: 84. 1852.

Herb.

Departmental distribution in Paraguay: Alto Paraguay, Caaguazú, Central, Cordillera, Ñeembucú, Paraguarí.

Voucher: *J. De Egea et al. 646* (BM, FCQ, MO).

##### ►*Doryopteris concolor* (Langsd. & Fisch.) Kuhn, Reisen Ost-Afrika 3(3): 19. 1879.

Syn.: *Cheilanthes concolor* (Langsd. & Fisch.) R.M.Tryon & A.F.Tryon; *Pellaea concolor* (Langsd. & Fisch.) Baker; *Pteris concolor* Langsd. & Fisch.

Herb.

Departmental distribution in Paraguay: Amambay, Caaguazú, Caazapá, Canindeyú, Cordillera, Ñeembucú, Paraguarí.

Voucher: *M. Peña-Chocarro et al. 2250* (BM, CTES, FCQ).

##### ►*Doryopteris pentagona* Pic.Serm., Webbia 60(1): 231. 2005.

Herb.

Departmental distribution in Paraguay: Canindeyú, Ñeembucú, Paraguarí, San Pedro.

Voucher: *M. Peña-Chocarro et al. 2293* (BM, CTES, FCQ, MO).

##### ►*Hemionitis tomentosa* (Lam.) Raddi, Opusc. Sci. 3: 284. 1819.

Syn.: *Asplenium tomentosum* Lam.; *Gymnopteris tomentosa* (Lam.) Underw.; *Gymnopteris rufa* auct. non (L.) Benth. ex Underw.

Herb.

Departmental distribution in Paraguay: Amambay, Canindeyú, Guairá, Ñeembucú, Paraguarí.

Voucher: *M. Peña-Chocarro et al. 2353* (BM, FCQ).

##### ►*Pityrogramma calomelanos* (L.) Link, Handbuch 3: 20. 1833.

Syn.: *Acrostichum calomelanos* L.; *Ceropteris calomelanos* (L.) Link, nom. superfl.; *Gymnogramma calomelanos* (L.) Kaulf.

Herb.

Departmental distribution in Paraguay: Amambay, Caazapá, Canindeyú, Central, Cordillera, Ñeembucú, Paraguarí, San Pedro.

Voucher: *C. Vogt 146* (CTES, FCQ).

#### SALVINIACEAE

##### ►*Salvinia auriculata* Aubl., Hist. Pl. Guiane 2: 969. 1775.

Syn.: *Salvinia rotundifolia* Willd.; *Salvinia hispida* Kunth

Aquatic.

Departmental distribution in Paraguay: Alto Paraguay, Ñeembucú, Presidente Hayes.

Voucher: *J. De Egea et al. 364* (BM, FCQ, MO).

##### ►*Salvinia biloba* Raddi, Pl. Bras. Nov. Gen. 1, 1: 4. 1825.

Syn.: *Salvinia herzogii* de la Sota

Aquatic.

Departmental distribution in Paraguay: Alto Paraguay, Ñeembucú.

Voucher: *C. Vogt 128* (CTES, FCQ).

##### ►*Salvinia minima* Baker, J. Bot. 4: 98. 1886.

Syn.: *Salvinia rotundifolia* auct. non Willd.

Aquatic.

Departmental distribution in Paraguay: Canindeyú, Ñeembucú.

Voucher: *S. Keel & L. Spinzi 1442* (FCQ).

#### THELYPTERIDACEAE

##### *Thelypteris interrupta* (Willd.) K.Iwats., J. Jap. Bot. 38(10): 314. 1963.

Syn.: *Aspidium gongylodes* Schkuhr; *Aspidium pohlianum* C.Presl; *Aspidium unitum* Mett.; *Cyclosorus gongylodes* (Schkuhr) Link; *Dryopteris gongylodes* (Schkuhr) Kuntze; *Goniopteris macrocladia* Fée; *Nephrodium gongylodes* (Schkuhr) Schott; *Nephrodium unitum* (Mett.) R.Br.; *Polypodium tottum* Thunb.; *Pteris interrupta* Willd.; *Thelypteris totta* (Thunb.) Schelpe

Herb.

Departmental distribution in Paraguay: Alto Paraná, Central, Cordillera, Guairá, Itapúa, Misiones, Ñeembucú, Paraguarí, Presidente Hayes, San Pedro.

Voucher: *S. Keel & L. Spinzi 1478* (FCQ).

### Seed Plants

#### ACANTHACEAE

##### *Dicliptera squarrosa Nees, Fl. Bras. (Martius) 9: 161. 1847

Syn.: *Diapedium niederleinianum* (Lindau) Kuntze; *Diapedium tweedianum* (Nees) Kuntze; *Dicliptera deltica* Bridar.; *Dicliptera imminuta* Rizzini; *Dicliptera lutea* Bridar.; *Dicliptera niederleiniana* Lindau; *Dicliptera pohliana* Nees; *Dicliptera sericea* Nees; *Dicliptera suberecta* (André) Bremek.; *Dicliptera tomentosa* Griseb.; *Dicliptera tweediana* Nees; *Dicliptera tweediana* Nees forma *chacoensis* Hassl.; *Dicliptera tweediana* Nees var. *flaviflora* Stuck.; *Jacobinia suberecta* André

Herb.

Departmental distribution in Paraguay: Alto Paraguay, Amambay, Caaguazú, Caazapá, Central, Cordillera, Guairá, Itapúa, Misiones, Ñeembucú, Paraguarí, Presidente Hayes, San Pedro.

Voucher: *L. Bernardi 20469* (cited in Ramella 2008: 21).

##### *Justicia brasiliana* Roth, Sp. Pl. Nov. 17. 1821.

Syn.: *Beloperone amherstiae* Nees; *Beloperone amherstiae* Nees var. *debilis* Nees; *Beloperone amherstiae* Nees var. *graciliflora* Nees; *Beloperone amherstiae* Nees var. puberula Hassl.; *Beloperone amherstiae* Nees var. *pubescens* Hassl.; *Beloperone amherstiae* Nees var. *villosula* Hassl.; *Beloperone brasiliana* (Roth) Bremek.; *Dianthera nodosa* (Hook.) Benth.; *Jacobinia affinis* (Rizzini) Wassh. & L.B.Sm.; *Jacobinia parabolica* (Nees) Lindau; *Justicia nodosa* Hook.; *Sericographis affinis* Rizzini; *Sericographis cordifolia* Rizzini; *Sericographis parabolica* Nees; *Sericographis squarrosa* Nees

Herb or shrub.

Departmental distribution in Paraguay: Alto Paraná, Amambay, Caaguazú, Caazapá, Canindeyú, Central, Concepción, Cordillera, Guairá, Itapúa, Misiones, Ñeembucú, Paraguarí, Presidente Hayes, San Pedro.

Voucher: *J. De Egea et al. 721* (BM, CTES, FCQ, G, MO, PY, SI, UNR).

##### ►*Justicia comata* (L.) Lam., Encycl. (Lamarck) 1(2): 632. 1785.

Syn.: *Dianthera comata* L.; *Ecbolium comatum* (L.) Kuntze; *Justicia humifusa* Sw.; *Justicia parviflora* (Nees) Lindau; *Leptostachya comata* (L.) Nees; *Leptostachya martiana* Nees; *Leptostachya parviflora* Nees; *Psacadocalymma comatum* (L.) Bremek.; *Stethoma comata* (L.) Britton; *Thalestris graminiformis* Rizzini

Herb.

Departmental distribution in Paraguay: Alto Paraná, Alto Paraguay, Amambay, Caaguazú, Caazapá, Central, Concepción, Cordillera, Guairá, Itapúa, Misiones, Ñeembucú, Paraguarí, Presidente Hayes, San Pedro.

Voucher: *M. Peña-Chocarro et al. 2335* (BM, CTES).

##### *Justicia laevilinguis* (Nees) Lindau, Bot. Jahrb. Syst. 19 (Beibl. 48): 20. 1894.

Syn.: *Dianthera laevilinguis* (Nees) Durand & B.D.Jacks.; *Dianthera obtusifolia* (Nees) Griseb.; *Dianthera paludosa* S.Moore; *Justicia anagallis* (Nees) Lindau; *Justicia ascendens* Bridar.; *Justicia laevilinguis* (Nees) Lindau var. *longifolia* Nees; *Justicia obtusifolia* (Nees) Lindau; *Justicia obtusifolia* var. *longispicata* Hassl.; *Justicia paludosa* (S.Moore) V.A.W.Graham; *Justicia repens* (Nees) Lindau, nom. illeg.; *Rhytiglossa anagallis* Nees; *Rhytiglossa laevilinguis* Nees; *Rhytiglossa laevilinguis* Nees var. *longifolia* Nees; *Rhytiglossa obtusifolia* Nees; *Rhytiglossa obtusifolia* Nees var. *hirsuticaulis* Nees; *Rhytiglossa repens* Nees

Herb. In swamps.

Departmental distribution in Paraguay: Alto Paraguay, Amambay, Caaguazú, Caazapá, Canindeyú, Central, Concepción, Cordillera, Guairá, Itapúa, Ñeembucú, Paraguarí, Presidente Hayes, San Pedro.

Voucher: *J. De Egea et al. 692* (BM, FCQ, MO).

##### *Ruellia angustiflora (Nees) Lindau ex Rambo, Iheringia, Bot. 12: 23. 1964.

Syn.: *Ruellia sanguinea* Griseb. var. *flaviflora* Hassl.; *Ruellia sanguinea* Griseb. var. *glabrescens* Hassl.; *Ruellia sanguinea* Griseb. var. *tomentosula* Hassl.; *Stephanophysum angustiflorum* Nees; *Stephanophysum quadrifarium* Nees

Shrub.

Departmental distribution in Paraguay: Alto Paraná, Amambay, Caaguazú, Caazapá, Canindeyú, Central, Concepción, Cordillera, Guairá, Itapúa, Misiones, Ñeembucú, Paraguarí, San Pedro.

Voucher: *R. Spichiger et al. 5326* (Ramella 2008, pp.56).

##### ►*Ruellia brevifolia* (Pohl) C.Ezcurra, Darwiniana 29: 278. 1989.

Syn.: *Cyrtacanthus corymbosus* Nees, nom. nud.; *Echinacanthus dichotomus* Kuntze; *Ruellia amoena* Nees, nom. nud.; *Ruellia graecizans* Backer; *Ruellia longifolia* (Pohl) Griseb.; *Ruellia serratitheca* Rusby; *Ruellia ventricosa* Kunth; *Stephanophysum brevifolium* Pohl; *Stephanophysum longifolium* Pohl; *Stephanophysum macrandrum* Bremek.; *Stephanophysum ventricosum* Nees

Herb.

Departmental distribution in Paraguay: Alto Paraná, Caazapá, Canindeyú, Ñeembucú, San Pedro.

Voucher: *C. Vogt 164* (CTES, FACEN, FCQ).

##### *Ruellia bulbifera Lindau, Nat. Pflanzenfam. IV (3b): 311. 1895.

Syn.: *Dipteracanthus tuberosus* Nees; *Ruellia loefgrenii* Lindau

Herb.

Departmental distribution in Paraguay: Alto Paraguay, Caaguazú, Caazapá, Central, Concepción, Cordillera, Guairá, Itapúa, Misiones, Ñeembucú, Paraguarí, Presidente Hayes, San Pedro.

Voucher: *R. Spichiger et al. 5215* (Ramella 2008: 56).

##### *Ruellia geminiflora* Kunth, Nov. Gen. Sp. (quarto ed.) 2: 240. 1817.

Syn.: *Copioglossa pilosa* Miers; *Dipteracanthus geminiflorus* Nees var. *angustifolius* Nees; *Dipteracanthus geminiflorus* Nees var. *erectus* Nees; *Dipteracanthus geminiflorus* Nees var. *hirsutior* Nees; *Dipteracanthus geminiflorus* Nees var. *procumbens* Nees; *Dipteracanthus geminiflorus* Nees var. *subacaulis* Nees; *Dipteracanthus humilis* Nees; *Dipteracanthus vindex* Nees; *Ruellia geminiflora* Kunth var. *hirsutior* Nees; *Ruellia hirsuta* Vell.

Herb or subshrub.

Departmental distribution in Paraguay: Amambay, Caaguazú, Caazapá, Canindeyú, Central, Concepción, Cordillera, Guairá, Itapúa, Misiones, Ñeembucú, Paraguarí, Presidente Hayes, San Pedro.

Voucher: *A.G. Schulz 7969* (cited in Ezcurra 1993: 834).

##### *Ruellia simplex* C.Wright, Anales Acad. Ci. Med. Habana 6 (41): 321. 1870.

Syn.: *Arrhostoxylum microphyllum* Nees; *Cryphiacanthus angustifolius* Nees; *Ruellia angustifolia* (Nees) Griseb. ex Lillo; *Ruellia brittoniana* Leonard ex Fernald; *Ruellia coerulea* Morong; *Ruellia ignorantiae* Herter

Herb.

Departmental distribution in Paraguay: Alto Paraguay, Amambay, Canindeyú, Central, Concepción, Cordillera, Guairá, Misiones, Ñeembucú, Paraguarí, Presidente Hayes, San Pedro.

Voucher: *M. Peña-Chocarro et al. 1971* (BM, CTES, FCQ, G, MO).

##### *Stenandrium dulce* (Cav.) Nees, Prodr. (DC.) 11: 282. 1847.

Syn.: *Gerardia dulcis* (Cav.) S.F.Blake; *Nierembergia prunellifolia* Dunal; *Ruellia dulcis* Cav.; *Stenandrium diphyllum* Nees var. *exscapum* Nees; *Stenandrium parodii* Bridar.; *Stenandrium trinerve* Nees

Herb.

Departmental distribution in Paraguay: Amambay, Central, Concepción, Cordillera, Guairá, Ñeembucú, Paraguarí.

Voucher: *T. Meyer 16203* (cited in Ariza Espinar and Ferrucci, 1982: 72).

#### ACHATOCARPACEAE

##### ►*Achatocarpus balansae* Schinz & Autran, Bull. Herb. Boissier 1: 6. 1893.

Tree.

Departmental distribution in Paraguay: Caaguazú, Guairá, Ñeembucú.

Voucher: *M. Vera et al. 217* (BM, CTES, FCQ, G, MO, PY, SI).

##### ►*Achatocarpus microcarpus* Schinz & Autran, Bull. Herb. Boissier 1: 8. 1893.

Tree or shrub.

Departmental distribution in Paraguay: Concepción, Guairá, Ñeembucú.

Voucher: *J. De Egea & R. Elsam 795* (BM, CTES, FCQ, MO).

#### AGAVACEAE

##### *Herreria bonplandii Lecomte, Bull. Soc. Bot. France 56: 346. 1909.

Syn.: *Dioscorea tuberosa* Rojas Acosta, nom. illeg.; *Herreria linearifolia* Dammer, nom. nud.; *Herreria montevidensis* Klotzsch ex Griseb. var. *bonplandii* (Lecomte) L.B.Sm.; *Herreria tuberosa* (Rojas Acosta) Rojas Acosta, nom. superfl.

Herb or climber.

Departmental distribution in Paraguay: Alto Paraná, Ñeembucú.

Voucher: *J. De Egea et al. 727* (BM, FCQ).

#### ALISMATACEAE

##### *Echinodorus grandiflorus (Cham. & Schltdl.) Micheli, Monogr. Phan. [A.DC. & C.DC.] 3: 57. 1881.

Syn.: *Alisma grandiflorum* Cham. & Schltdl.; *Echinodorus argentinensis* Rataj; *Echinodorus ellipticus* (Mart.) Micheli var. *latifolius* auct. non Micheli; *Echinodorus ellipticus* (Mart.) Micheli var. *ovatus* auct. non Micheli; *Echinodorus grandiflorus* (Cham. & Schltdl.) Micheli var. *ovatus* Micheli; *Echinodorus pellucidus* Rataj

Herb. In swamps.

Departmental distribution in Paraguay: Alto Paraguay, Caaguazú, Caazapá, Canindeyú, Central, Cordillera, Guairá, Misiones, Ñeembucú, Paraguarí, Presidente Hayes.

Voucher: *M. Peña-Chocarro et al. 1978* (BM, CTES, FCQ).

##### ►*Echinodorus paniculatus* Micheli, Monogr. Phan. [A.DC. & C.DC.] 3: 51. 1881.

Syn.: *Echinodorus paniculatus* Micheli forma *latifolia* Chodat & Hassl.; *Echinodorus paniculatus* Micheli var. *brevifolia* Hauman

Aquatic herb.

Departmental distribution in Paraguay: Alto Paraguay, Canindeyú, Central, Concepción, Cordillera, Ñeembucú, Presidente Hayes.

Voucher: *C. Vogt 172* (CTES, FACEN, FCQ).

##### *Helanthium bolivianum (Rusby) Lehtonen & Myllys, Cladistics 24(2): 228. 2008.

Syn.: *Alisma boliviana* Rusby; *Alisma ranunculoides* L. var. *brasiliense* A.St.-Hil.; *Echinodorus angustifolius* Rataj; *Echinodorus austroamericanus* Rataj; *Echinodorus bolivianus* (Rusby) Holm-Niels.; *Echinodorus quadricostatus* Fassett; *Echinodorus quadricostatus* Fassett var. *xinguensis* Rataj; *Echinodorus tenellus* (Mart.) Buchenau var. *latifolius* (Seub.) Fassett

Aquatic herb.

Departmental distribution in Paraguay: Amambay, Caaguazú, Canindeyú, Central, Guairá, Ñeembucú, Paraguarí.

Voucher: *C. Vogt et al. 148* (CTES, FCQ).

##### ►*Helanthium tenellum* (Mart.) Britton, Man. Fl. N. States [Britton], ed. 2. 54. 1905.

Syn.: *Alisma tenellum* Mart.; *Echinodorus tenellus* (Mart.) Buchenau

Aquatic herb.

Departmental distribution in Paraguay: Amambay, Canindeyú, Ñeembucú.

Voucher: *S. Keel & L. Spinzi 1458* (FCQ).

##### ►*Hydrocleys* *nymphoides* (Willd.) Buchenau, Index Crit. Butom. Alism. Juncag. 9. 1868.

Syn.: *Hydrocleys commersonii* Rich.; *Hydrocleys humboldtii* (Rich.) Endl., nom. illeg.; *Limnocharis commersonii* (Rich.) Spreng.; *Limnocharis nymphoides* (Willd.) Michx.; *Stratioides nymphoides* Willd.

Aquatic herb.

Departmental distribution in Paraguay: Alto Paraguay, Amambay, Central, Concepción, Cordillera, Ñeembucú, Presidente Hayes.

Voucher: *J. De Egea et al. 735* (BM, CTES, FCQ, G, MO, PY, UNR).

##### *Sagittaria montevidensis* Cham. & Schltdl., Linnaea 2: 156. 1827.

Syn.: *Sagittaria andina* Phil.; *Sagittaria montevidensis* Cham. & Schltdl. forma *normalis* Hauman, nom. illeg.; *Sagittaria multinervia* Larrañaga; *Sagittaria pugioniformis* L. var. *montevidensis* (Cham. & Schltdl.) Kuntze

Herb. In swamps.

Departmental distribution in Paraguay: Alto Paraguay, Amambay, Caaguazú, Central, Cordillera, Guairá, Ñeembucú, Paraguarí, San Pedro.

Voucher: *J. De Egea et al. 644* (BM, CTES, FCQ, G, MO, PY, SI).

##### *Sagittaria rhombifolia Cham., Linnaea 10: 219. 1835.

Syn.: *Sagittaria affinis* Seub.; *Sagittaria amazonica* Huber; *Sagittaria lagoensis* Seub. & Warm.; *Sagittaria pugioniformis* auct. non L.; *Sagittaria pugioniformis* L. var. *affinis* (Seub.) Kuntze; *Sagittaria pugioniformis* L. var. *platyphylla* Micheli; *Sagittaria pugioniformis* L. var. *rhombifolia* (Cham.) Kuntze; *Sagittaria rhomboidalis* Micheli

Herb. In swamps.

Departmental distribution in Paraguay: Amambay, Canindeyú, Central, Ñeembucú.

Voucher: *R. Spichiger et al. 5285* (cited by Ramella 2008: 77).

#### AMARANTHACEAE

##### *Alternanthera paronychioides A.St.-Hil. subsp. *chacoensis* (Morong ex Morong & Britton) Pedersen, Darwiniana 14: 440. 1967.

Syn.: *Alternanthera chacoensis* Morong ex Morong & Britton; *Alternanthera ficoidea* (L.) Sm. subsp. *chacoensis* (Morong ex Morong & Britton) Pedersen; *Alternanthera morongii* Uline; *Alternanthera paronychioides* A.St.-Hil. var. *chacoensis* (Morong ex Morong & Britton) Pedersen; *Alternanthera paronychioides* A.St.-Hil. var. *robusta* Chodat

Herb.

Departmental distribution in Paraguay: Alto Paraguay, Central, Concepción, Cordillera, Guairá, Ñeembucú, Paraguarí.

Voucher: *J. De Egea et al. 697-B* (FCQ).

##### *Alternanthera philoxeroides (Mart.) Griseb forma *angustifolia* Suess., Feddes Repert. 35: 303. 1934.

Syn.: *Alternanthera philoxeroides* (Mart.) Griseb. var. *lancifolia* Chodat; *Alternanthera philoxeroides* (Mart.) Griseb. var. *luxurians* Chodat; *Telanthera philoxeroides* (Mart.) Moq. var. *linearifolia* Chodat

Aquatic herb.

Departmental distribution in Paraguay: Central, Ñeembucú.

Voucher: *M.A. Walter 105* (BM, G).

##### ►*Alternanthera reineckii* Briq., Annuaire Conserv. Jard. Bot. Genève 3: 151. 1899.

Syn.: *Achyranthes reineckii* (Briq.) Standl.; *Alternanthera pilosa* Moq. forma *petiolata* Chodat; *Alternanthera pilosa* Moq. var. *microphylla* Chodat

Herb.

Departmental distribution in Paraguay: Cordillera, Ñeembucú.

Voucher: *M.A. Walter 101* (BM).

##### *Alternanthera* sp.

Herb (?).

Voucher: *L. Bernardi 18501* (cited in Ramella 2008: 80).

##### *Amaranthus hybridus L., Sp. Pl. 2: 990.1753.

Syn.: *Amaranthus chlorostachys* Willd.; *Amaranthus hybridus* L. var. *hypochondriacus* (L.) Rob.; *Amaranthus hybridus* L. var. *quitensis* (Kunth) Covas; *Amaranthus hypochondriacus* L.; *Amaranthus quitensis* Kunth; *Amaranthus quitensis* Kunth var. *stuckertianus* Thell.

Herb.

Departmental distribution in Paraguay: Alto Paraguay, Ñeembucú.

Voucher: *L. Bernardi 18506* (Ramella 2008: 80).

##### *Gomphrena celosioides Mart. forma *aureiflora* (Chodat & Hassl.) Pedersen, Darwiniana 20: 272. 1976.

Syn.: *Gomphrena celosioides* Mart. forma *grandifolia* Stuchlik; *Gomphrena celosioides* Mart. forma *parvifolia* Stuchlik; *Gomphrena celosioides* Mart. var. *aureiflora* Stuchlik; *Gomphrena decumbens* Jacq. forma *aureiflora* Chodat & Hassl.; *Gomphrena decumbens* Jacq. var. *aureiflora* (Chodat & Hassl.) Stuchlik; *Gomphrena hygrophila* Mart. forma *luteiflora* Herzog

Herb.

Departmental distribution in Paraguay: Ñeembucú.

Voucher: *M. Peña-Chocarro et al. 2227* (BM, CTES, FCQ, G, MO, PY, SI, UNR).

##### *Gomphrena celosioides Mart. forma *celosioides*, Nova Acta Phys.-Med. Acad. Caes. Leop.-Carol. Nat. Cur. 13: 301. 1826.

Syn.: *Gomphrena decumbens* Jacq. forma *albiflora* Chodat; *Gomphrena decumbens* Jacq. var. *albiflora* (Chodat & Hassl.) Stuchlik; *Gomphrena decumbens* Jacq. var. *genuina* Stuchlik, nom. illeg.; *Gomphrena perennis* L. forma *ramosissima* Stuchlik; *Xeraea celosioides* (Mart.) Kuntze

Herb.

Departmental distribution in Paraguay: Alto Paraguay, Caazapá, Cordillera, Ñeembucú.

Voucher: *J. De Egea et al. 697-A* (BM).

##### ►*Gomphrena celosioides* Mart. forma *roseiflora* (Chodat & Hassl.) Pedersen, Darwiniana 20: 273. 1976

Syn.: *Gomphrena decumbens* Jacq. forma *roseiflora* Chodat & Hassl.; *Gomphrena decumbens* Jacq. var. *roseiflora* (Chodat & Hassl.) Stuchlik; *Gomphrena hygrophila* Mart. forma *subecristata* Herzog

Herb.

Departmental distribution in Paraguay: Caaguazú, Central, Cordillera, Ñeembucú.

Voucher: *M. Peña-Chocarro et al. 2229* (BM, FCQ).

##### *Gomphrena pulchella Mart., Nova Acta Phys.-Med. Acad. Caes. Leop.-Carol. Nat. Cur. 13: 302. 1826.

Syn.: *Gomphrena pulchella* Mart. forma *grandifolia* Stuchlik; *Gomphrena pulchella* Mart. forma *linearifolia* Stuchlik; *Gomphrena pulchella* Mart. var. *ecristata* Chodat; *Gomphrena pulchella* Mart. var. *rosea* Stuchlik, pro parte; *Xeraea pulchella* (Mart.) Kuntze

Herb.

Departmental distribution in Paraguay: Alto Paraguay, Central, Concepción, Cordillera, Ñeembucú, Paraguarí, Presidente Hayes.

Voucher: *L. Bernardi 18377* (Ramella 2008: 80).

##### *Iresine diffusa Humb. & Bonpl. ex Willd., Sp. Pl., ed. 4 [Willdenow] 4(2): 765. 1806.

Syn.: *Celosia paniculata* L.; *Iresine celosia* L., nom. superfl.; *Iresine celosia* L. var. *diffusa* (Humb. & Bonpl. ex Willd.) Suess.; *Iresine celosioides* L., nom. superfl.; *Iresine celosioides* var. *polymorpha* (Mart.) Griseb.; *Iresine paniculata* (L.) Kuntze, nom. illeg.; *Iresine polymorpha* Mart.

Subshrub.

Departmental distribution in Paraguay: Alto Paraguay, Caazapá, Canindeyú, Ñeembucú, Paraguarí.

Voucher: *J. De Egea & R. Elsam 797* (BM, CTES, FCQ, MO).

##### *Pfaffia glomerata (Spreng.) Pedersen, Darwiniana 14: 450. 1967.

Syn.: *Alternanthera glauca* (Mart.) Hosseus; *Gomphrena glauca* (Mart.) Moq.; *Gomphrena luzuliflora* (Mart.) Moq.; *Gomphrena stenophylla* Spreng.; *Iresine glomerata* Spreng.; *Iresine luzuliflora* (Mart.) Griseb.; *Mogiphanes dunaliana* (Moq.) Griseb.; *Mogiphanes glauca* (Mart.) Griseb.; *Pfaffia divergens* O.Stützer; *Pfaffia glabrescens* Suess.; *Pfaffia glauca* (Mart.) Spreng.; *Pfaffia iresinoides* (Kunth) Spreng. var. *angustifolia* O.Stützer; *Pfaffia luzuliflora* (Mart.) D.Dietr.; *Pfaffia luzuliflora* (Mart.) D.Dietr. forma *gracilis* O.Stützer; *Pfaffia luzuliflora* (Mart.) D.Dietr. forma *virgata* O.Stützer; *Pfaffia luzuliflora* (Mart.) D.Dietr. var. *microcephala* O.Stützer ex Suess.; *Pfaffia luzuliflora* (Mart.) D.Dietr. var. *paniculata* O.Stützer; *Pfaffia luzuliflora* (Mart.) D.Dietr. var. *squarrosa* O.Stützer; *Pfaffia stenophylla* (Spreng.) Stuchlik; *Sertuernera glauca* Mart.; *Sertuernera luzuliflora* Mart.

Herb or subshrub.

Departmental distribution in Paraguay: Alto Paraguay, Cordillera, Ñeembucú, Paraguarí, Presidente Hayes, San Pedro.

Voucher: *M. Peña-Chocarro et al. 1993* (BM, CTES, FCQ, G, MO, PY, SI).

##### *Pfaffia gnaphaloides (L.f.) Mart., Nov. Gen. Sp. Pl. [Martius] 2: 24. 1826.

Syn.: *Celosia gnaphaloides* L.f.; *Gomphrena gnaphaloides* (L.f.) Vahl ex Roem. & Schult.; *Gomphrena lanata* Poir.; *Gomphrena lanata* Poir. var. *latifolia* Seub.; *Gomphrena lanata* Poir. var. *oblongifolia* Moq.; *Gomphrena lanata* Poir. var. *parvifolia* Moq.; *Gomphrena perennis* L. var. *brunnea* Stuchlik; *Gomphrena phagnaloides* Griseb.; *Gomphrena poiretiana* Roem. & Schult.; *Oplotheca poiretiana* (Schult.) Mart.; *Pfaffia lanata* (Poir.) Gibert; *Pfaffia lanata* (Poir.) Gibert forma *parvifolia* (Moq.) O.Stützer; *Pfaffia lanata* (Poir.) Gibert var. *discolor* Suess.; *Pfaffia lanata* (Poir.) Gibert var. *latifolia* (Seub.) O.Stützer; *Pfaffia lanata* (Poir.) Gibert var. *oblongifolia* (Moq.) O.Stützer; *Pfaffia lanata* (Poir.) Gibert var. *peteriana* Kuntze; *Pfaffia poiretiana* (Schult.) Stuchlik; *Pfaffia tenuis* N.E.Br.; *Pfaffia tomentosa* Mart.; *Xeraea phagnaloides* (Griseb.) Kuntze

Herb.

Departmental distribution in Paraguay: Caaguazú, Ñeembucú.

Voucher: *T. Rojas 12637* (LIL) (http://www.ville-ge.ch/musinfo/bd/cjb/fdp/).

##### *Pfaffia tuberosa (Spreng.) Hicken, Apuntes Hist. Nat. 2: 93. 1910.

Syn.: *Gomphrena sericea* Spreng., nom. illeg.; *Gomphrena tuberosa* Spreng.; *Gomphrena tuberosa* Spreng. var. *glabrescens* Moq.; *Gomphrena tuberosa* Spreng. var. *sericea* (Mart.) Seub; *Gomphrena tuberosa* Spreng. var. *vestita* Moq.; *Pfaffia sericea* (Spreng.) Mart.; *Pfaffia sericea* (Spreng.) Mart. forma *glabrescens* (Moq.) O.Stützer; *Pfaffia sericea* (Spreng.) Mart. forma *vestita* (Moq.) O.Stützer

Herb.

Departmental distribution in Paraguay: Caazapá, Canindeyú, Itapúa, Misiones, Ñeembucú.

Voucher: *T. Meyer 16151* (G) (http://www.ville-ge.ch/musinfo/bd/cjb/fdp/).

#### AMARYLLIDACEAE

##### *Nothoscordum* sp.

Herb.

Voucher: *L. Bernardi 18481* (cited in Ramella 2008: 286).

#### ANACARDIACEAE

##### *Astronium balansae Engl., Bot. Jahrb. Syst. 1: 45. 1881.

Tree.

Departmental distribution in Paraguay: Alto Paraná, Central, Cordillera, Itapúa, Ñeembucú, Paraguarí, Presidente Hayes.

Voucher: *J. De Egea et al. 725* (BM, CTES, FCQ, G, MO, PY, UNR).

##### *Lithraea molleoides* (Vell.) Engl., Fl. Bras. (Martius) 12(2): 394.1876.

Syn.: *Lithraea gilliesii* Griseb.; *Lithraea molleoides* (Vell.) Engl. var. *lorentziana* Hieron. ex Lillo; *Lithraea ternifolia* (Hook.) F.A.Barkley; *Schinus brasiliensis* Marchand ex Cabrera, pro parte; *Schinus molleoides* Vell.; *Schinus ternifolius* Hook.

Tree.

Departmental distribution in Paraguay: Amambay, Caaguazú, Caazapá, Canindeyú, Central, Cordillera, Guairá, Itapúa, Misiones, Ñeembucú, Paraguarí, San Pedro.

Voucher: *M. Peña-Chocarro et al. 2212* (BM, CTES, FCQ, G, MO, PY, SI).

##### *Schinopsis balansae* Engl., Bot. Jahrb. Syst. 6: 286. 1885.

Syn.: *Quebrachia morongii* Britton; *Schinopsis balansae* Engl. var. *pendula* Tortorelli

Tree.

Departmental distribution in Paraguay: Alto Paraná, Alto Paraguay, Concepción, Ñeembucú, Paraguarí, Presidente Hayes, San Pedro.

Voucher: *M. Peña-Chocarro et al. 2284* (BM, CTES, FCQ, G, MO, PY, SI, UNR).

##### *Schinus fasciculatus* (Griseb.) I.M.Johnst., J. Arnold Arbor. 19: 257. 1938.

Syn.: *Duvaua fasciculata* Griseb.; *Duvaua praecox* Griseb. var. *glomerata* Griseb.; *Schinus huyngan* Molina var. *heterophylla* Kuntze; *Schinus polygamus* (Cav.) Cabrera forma *fasciculatus* (Griseb.) Cabrera; *Schinus polygamus* (Cav.) Cabrera forma *heterophyllus* (Kuntze) Cabrera

Tree or shrub.

Departmental distribution in Paraguay: Alto Paraguay, Central, Concepción, Ñeembucú, Presidente Hayes.

Voucher: *L. Bernardi 18432* (BM).

#### ANNONACEAE

##### *Rollinia emarginata* Schltdl., Linnaea 9: 318. 1835.

Syn.: *Rollinia emarginata* Schltdl. var. *longipetala* (R.E.Fr.) R.E.Fr.; *Rollinia glaucescens* Sond.; *Rollinia hassleriana* R.E.Fr.; *Rollinia hassleriana* R.E.Fr. var. *vestita* R.E.Fr.; *Rollinia longipetala* R.E.Fr.; *Rollinia sonderiana* Walp.

Tree or shrub.

Departmental distribution in Paraguay: Alto Paraná, Amambay, Caazapá, Canindeyú, Central, Concepción, Cordillera, Guairá, Itapúa, Misiones, Ñeembucú, Paraguarí, San Pedro.

Voucher: *J. De Egea et al. 716* (BM, FCQ, MO).

#### APIACEAE

##### ►*Cyclospermum leptophyllum* (Pers.) Sprague, J. Bot. 61: 131. 1923.

Syn.: *Apium ammi* Urb.; *Apium ammi* forma *filamentosum* (Kuntze) H.Wolff; *Apium ammi* Urb.forma *nanum* Kuntze; *Apium ammi* Urb.forma *pedunculata* Chodat; *Apium ammi* Urb.var. *filamentosum* Kuntze; *Apium ammi* Urb.. var. *genuinum* H.Wolff, nom illeg.; *Apium ammi* Urb. var. *leptophyllum* (Pers.) Kuntze; *Apium laciniatum* (DC.) Urb. forma *elatius* (Hook. & Arn.) H.Wolff; *Apium leptophyllum* (Pers.) F.Muell.; *Apium ranunculifolium* (DC.) Reiche, nom. illeg.; *Apium ranunculifolium* auct. non Kunth; *Helosciadium laciniatum* DC. var. *elatius* Hook. & Arn.; *Helosciadium leptophyllum* (Pers.) DC.; *Helosciadium ranunculifolium* DC.; *Pimpinella leptophylla* Pers.

Herb.

Departmental distribution in Paraguay: Alto Paraná, Alto Paraguay, Ñeembucú.

Voucher: *M. Peña-Chocarro et al. 2264* (BM, CTES, FCQ, MO).

##### ►*Eryngium ebracteatum* Lam., Encycl. (Lamarck) 4(2): 759. 1798.

Syn.: *Eryngium ebracteatum* Lam. var. *poterioides* (Griseb.) Urb.; *Eryngium poterioides* Griseb.

Herb.

Departmental distribution in Paraguay: Alto Paraguay, Caazapá, Canindeyú, Ñeembucú, Presidente Hayes.

Voucher: *M. Peña-Chocarro et al. 2221* (BM, CTES, FCQ).

##### ►*Eryngium eburneum* Decne., Bull. Soc. Bot. France 20: 23. 1873.

Syn.: *Eryngium bracteosum* (DC.) Griseb.

Herb.

Departmental distribution in Paraguay: Caaguazú, Canindeyú, Cordillera, Ñeembucú.

Voucher: *M.A. Walter 30* (BM).

##### *Eryngium elegans Cham. & Schltdl., Linnaea 1: 348. 1826.

Syn.: *Eryngium elegans* Cham. & Schltdl. var. *genuinum* Urb., nom. illeg.; *Eryngium elegans* Cham. & Schltdl. var. *macrocephalum* Urb.; *Eryngium elegans* Cham. & Schltdl. var. *microcephalum* Urb.; *Eryngium elegans* Cham. & Schltdl. var. *uncinatum* (Cham. & Schltdl.) Urb.; *Eryngium uncinatum* Cham. & Schltdl.

Herb.

Departmental distribution in Paraguay: Amambay, Cordillera, Itapúa, Ñeembucú, Paraguarí.

Voucher: *M. Peña-Chocarro et al. 2233* (FCQ).

##### ►*Eryngium horridum* Malme

Syn.: *Eryngium schwackeanum* Urb.

Herb.

Departmental distribution in Paraguay: Canindeyú, Cordillera, Ñeembucú, Paraguarí.

Voucher: *S. Keel & L. Spinzi v-144* (FCQ).

##### ►*Eryngium pandanifolium* Cham. & Schltdl., Linnaea 1: 336. 1826.

Syn.: *Eryngium decaisneanum* Urb.; *Eryngium oligodon* Griseb.

Herb.

Departmental distribution in Paraguay: Alto Paraná, Ñeembucú.

Voucher: *M. Peña-Chocarro et al. 2234* (BM, CTES, FCQ, MO).

##### *Eryngium* sp. 1

Herb.

Voucher: *M. Peña-Chocarro et al. 2201* (FCQ).

##### *Eryngium* sp. 2

Herb.

Voucher: *M. Peña-Chocarro et al. 2202* (FCQ).

##### ►*Hydrocotyle bonariensis* Lam., Encycl. (Lamarck) 3(1): 153. 1789.

Syn.: *Hydrocotyle multiflora* Ruiz & Pav.; *Hydrocotyle umbellata* L. var. *bonariensis* (Lam.) Spreng.

Herb.

Departmental distribution in Paraguay: Canindeyú, Itapúa, Ñeembucú.

Voucher: *S. Keel & L. Spinzi v-136* (FCQ).

##### *Hydrocotyle leucocephala Cham. & Schltdl., Linnaea 1: 364. 1826.

Syn.: *Hydrocotyle callicephala* Cham. & Schltdl.; *Hydrocotyle leucocephala* Cham. & Schltdl. var. *minuta* (Pohl ex DC.) Urb.; *Hydrocotyle leucocephala* Cham. & Schltdl. var. *obtusiloba* Urb.; *Hydrocotyle leucocephala* Cham. & Schltdl. var. *truncatiloba* Urb.; *Hydrocotyle minuta* Pohl ex DC.

Herb.

Departmental distribution in Paraguay: Alto Paraná, Caazapá, Canindeyú, Cordillera, Itapúa, Ñeembucú.

Voucher: *R. Spichiger et al. 5282* (CTES, G, MO) (http://www.ville-ge.ch/musinfo/bd/cjb/fdp/).

##### ►*Hydrocotyle ranunculoides* L.f., Suppl. Pl. 177. 1782.

Syn.: *Hydrocotyle batrachoides* DC.; *Hydrocotyle natans* Cirillo; *Hydrocotyle ranunculoides* L.f. var. *brasiliensis* Urb.; *Hydrocotyle ranunculoides* L.f. var. *inciso*-*crenata* Urb.; *Hydrocotyle ranunculoides* L.f. var. *lobata* Urb.

Herb.

Departmental distribution in Paraguay: Caazapá, Ñeembucú, Presidente Hayes.

Voucher: *M.A. Walter 48* (BM).

#### APOCYNACEAE

##### ►*Asclepias mellodora* A.St.-Hil., Hist. Pl. Remarq. Bresil 227. 1826.

Syn.: *Asclepias campestris* Decne.; *Asclepias campestris* Decne. var. *angustifolia* Kuntze; *Asclepias campestris* Decne. var. *schlechteri* Kuntze; *Asclepias curupi* E.Fourn.; *Asclepias jangadensis* S.Moore; *Asclepias marginata* Decne. var. *bodenbenderi* Kuntze; *Asclepias mellodora* A.St.-Hil. var. *bodenbenderi* (Kuntze) Bollwinkel; *Asclepias mellodora* A.St.-Hil. var. *minor* A.St.-Hil.; *Asclepias mellodora* A.St.-Hil. var. *multinervis* (E.Fourn.) Bollwinkel; *Asclepias multinervis* E.Fourn.; *Asclepias nervosa* Decne.; *Asclepias papillosa* Silveira; *Asclepias umbellata* Vell.

Herb.

Departmental distribution in Paraguay: Amambay, Canindeyú, Cordillera, Guairá, Itapúa, Ñeembucú, Paraguarí, San Pedro.

Voucher: *M. Peña-Chocarro et al. 1976* (BM, CTES, FCQ, G, MO).

##### *Asclepias* sp.

Herb.

Voucher: *J. De Egea et al. 714* (FCQ).

##### *Aspidosperma quebracho-blanco* Schltdl., Bot. Zeitung (Berlin) 19: 137. 1861.

Syn.: *Aspidosperma crotalorum* Speg.; *Aspidosperma quebracho-blanco* Schltdl. forma *malmeana* Markgr.; *Aspidosperma quebracho-blanco* Schltdl. forma *schlechtendaliana* Markgr.; *Aspidosperma quebracho-blanco* Schltdl. forma *spegazziana* Markgr.; *Aspidosperma quebracho-blanco* Schltdl. var. *pendula* Speg.; *Aspidosperma quebrachoideum* Rojas Acosta; *Macaglia quebracho* Kuntze

Tree.

Departmental distribution in Paraguay: Alto Paraguay, Central, Concepción, Ñeembucú, Paraguarí, Presidente Hayes.

Voucher: *L. Bernardi 18445* (BM, G, MO, NY).

##### ►*Forsteronia glabrescens* Müll.Arg., Fl. Bras. (Martius) 6(1): 102. 1860.

Syn.: *Thyrsanthus glabrescens* (Müll.Arg.) Miers

Climber.

Departmental distribution in Paraguay: Alto Paraná, Amambay, Caaguazú, Caazapá, Canindeyú, Central, Concepción, Cordillera, Guairá, Ñeembucú, Paraguarí, San Pedro.

Voucher: *J. De Egea et al. 366* (BM, CTES).

##### *Morrenia odorata* (Hook. & Arn.) Lindl., Edward’s Bot. Reg. 24: 71. 1838.

Syn.: *Cynanchum odoratum* Hook. & Arn.

Climber.

Departmental distribution in Paraguay: Alto Paraguay, Central, Cordillera, Itapúa, Ñeembucú, Paraguarí, Presidente Hayes.

Voucher: *M. Peña-Chocarro et al. 2290* (BM, CTES, FCQ).

##### ►*Oxypetalum balansae* Malme, Kongl. Svenska Vetensk. Acad. Handl. 34(7): 51. 1900.

Climber.

Departmental distribution in Paraguay: Canindeyú, Central, Concepción, Cordillera, Guairá, Ñeembucú, Paraguarí, Presidente Hayes.

Voucher: *M. Peña-Chocarro et al. 2282* (BM, CTES, FCQ, MO).

##### *Rhabdadenia ragonesei Woodson, Lilloa 5: 199. 1940.

Climber.

Departmental distribution in Paraguay: Caaguazú, Canindeyú, Central, Cordillera, Guairá, Misiones, Ñeembucú, Paraguarí, Presidente Hayes, San Pedro.

Voucher: *M. Vera 210* (BM).

##### ►*Schubertia grandiflora* Mart., Nov. Gen. Sp. Pl. (Martius) 1: 57. 1824.

Syn.: *Araujia grandiflora* (Mart.) Morong

Climber.

Departmental distribution in Paraguay: Alto Paraná, Amambay, Canindeyú, Cordillera, Ñeembucú, San Pedro.

Voucher: *M. Peña-Chocarro et al. 2287* (BM, CTES, FCQ, G, MO, PY, SI).

##### *Tabernaemontana catharinensis* A.DC., Prodr. (DC.) 8: 365. 1844.

Syn.: *Peschiera acuminata* Miers; *Peschiera affinis* (Müll.Arg.) Miers; *Peschiera albidiflora* Miers; *Peschiera australis* (Müll.Arg.) Miers; *Peschiera australis* (Müll.Arg.) Miersvar. *hilariana* (Müll.Arg.) L.Allorge; *Peschiera catharinensis* (A.DC.) Miers; *Peschiera hilariana* (Müll.Arg.) Miers; *Tabernaemontana australis* Müll.Arg.; *Tabernaemontana hilariana* Müll.Arg.

Tree or shrub.

Departmental distribution in Paraguay: Alto Paraná, Amambay, Caaguazú, Canindeyú, Central, Concepción, Cordillera, Guairá, Itapúa, Misiones, Ñeembucú, Paraguarí, Presidente Hayes, San Pedro.

Voucher: *M. Morinigo et al. 1* (BM, CTES, FCQ, G, MO, PY, SI).

##### *Thevetia bicornuta Müll.Arg., Linnaea 30: 392. 1860.

Syn.: *Cascabela bicornuta* (Müll.Arg.) Lippold, nom. nud.; *Thevetia paraguayensis* Britton

Herb. In swamps.

Departmental distribution in Paraguay: Alto Paraguay, Central, Concepción, Ñeembucú, Presidente Hayes, San Pedro.

Voucher: *A.G. Schulz 7924* (cited in Ezcurra et al. 1992: 115).

#### ARACEAE

##### *Anthurium paraguayense* Engl., Bot. Jahrb. Syst. 25: 361. 1898.

Syn.: *Anthurium coriaceum* auct. non G. Don; *Anthurium rodrigoi* A.D.Hawkes

Herb.

Departmental distribution in Paraguay: Alto Paraguay, Amambay, Caazapá, Central, Concepción, Cordillera, Ñeembucú, Paraguarí, Presidente Hayes, San Pedro.

Voucher: *O. Kuntze 1492* (cited in Croat and Mount 1988: 11).

##### *Philodendron tweedieanum* Schott, Bonplandia 7: 29. 1859.

Syn.: *Philodendron dubium* Chodat; *Philodendron petraeum* Chodat; *Philodendron petraeum* Chodatvar. *triangulare* Chodat & Vischer; *Philodendron petraeum* Chodatvar. *valenzuelae* Chodat & Vischer

Herb.

Departmental distribution in Paraguay: Alto Paraguay, Amambay, Caaguazú, Central, Cordillera, Guairá, Itapúa, Ñeembucú, Paraguarí, San Pedro.

Voucher: *M.A. Walter 19* (BM).

##### *Pistia stratiotes L., Sp. Pl. 2: 963. 1753.

Syn.: *Pistia occidentalis* Blume; *Pistia stratiotes* L. var. *linguiformis* (Blume) Engl.; *Pistia stratiotes* L. var. *obcordata* (Schleid.) Engl.

Aquatic herb.

Departmental distribution in Paraguay: Alto Paraguay, Central, Cordillera, Guairá, Ñeembucú, Paraguarí, Presidente Hayes.

Voucher: *M. Peña-Chocarro et al. 2364* (BM, UNR).

#### ARECACEAE

##### *Acrocomia aculeata (Jacq.) Lodd. ex Mart., Hist. Nat. Palm. 3(8) 286. 1845.

Syn.: *Acrocomia chunta* Covas & Ragonese; *Acrocomia totai* Mart.; *Cocos aculeata* Jacq.

Palm.

Departmental distribution in Paraguay: Amambay, Canindeyú, Central, Concepción, Cordillera, Guairá, Misiones, Ñeembucú, Paraguarí, San Pedro.

Voucher: Cited in Vogt and Mereles 2005: 10.

##### *Butia paraguayensis* (Barb.Rodr.) L.H.Bailey, Gentes Herb. 4: 47. 1936.

Syn.: *Butia amadelpha* (Barb.Rodr.) Burret; *Butia arenicola* (Barb.Rodr.) Burret; *Butia dyerana* (Barb.Rodr.) Burret; *Butia pungens* Becc.; *Butia wildemaniana* (Barb.Rodr.) Burret; *Butia yatay* (Mart.) Becc. subsp. *paraguayensis* (Barb.Rodr.) Xifreda & Sanso; *Butia yatay* (Mart.) Becc. var. *paraguayensis* (Barb.Rodr.) Becc.; *Cocos amadelpha* Barb.Rodr.; *Cocos arenicola* Barb.Rodr.; *Cocos dyerana* Barb.Rodr.; *Cocos paraguayensis* Barb.Rodr.; *Cocos wildemaniana* Barb.Rodr.; *Syagrus amadelpha* (Barb.Rodr.) Frambach; *Syagrus arenicola* (Barb.Rodr.) Frambach; *Syagrus dyerana* (Barb.Rodr.) Becc.; *Syagrus paraguayensis* (Barb.Rodr.) Glassman; *Syagrus wildemaniana* (Barb.Rodr.) Frambach ex Dahlgren

Palm.

Departmental distribution in Paraguay: Amambay, Caaguazú, Caazapá, Canindeyú, Concepción, Cordillera, Guairá, Misiones, Ñeembucú, San Pedro.

Voucher: *L. Bernardi 20497* (BM, G, NY).

##### *Copernicia alba* Morong, Ann. New York Acad. Sci. 7: 246. 1893.

Syn.: *Copernicia australis* Becc.; *Copernicia australis* Becc. var. *alba* (Morong) Bertoni ex Hauman; *Copernicia australis* Becc. var. *nigra* (Morong) Bertoni; *Copernicia cerifera* Mart.; *Copernicia chacoana* Rojas Acosta, nom. superfl.; *Copernicia nigra* Morong ex Becc., nom. nud.; *Copernicia ramulosa* Burret; *Copernicia rubra* Morong; *Coryphomia tectorum* Rojas Acosta

Palm.

Departmental distribution in Paraguay: Alto Paraguay, Central, Concepción, Cordillera, Ñeembucú, Paraguarí, Presidente Hayes.

Voucher: *T. Meyer 16106* (LIL) (http://www.ville-ge.ch/musinfo/bd/cjb/fdp/).

##### *Syagrus romanzoffiana (Cham.) Glassman, Fieldiana, Bot. 31(17): 382. 1968.

Syn.: *Arecastrum romanzoffianum* (Cham.) Becc.; *Arecastrum romanzoffianum* (Cham.) Becc. var. *australe* (Mart.) Becc.; *Arecastrum romanzoffianum* (Cham.) Becc. var. *genuinum* Becc. nom. illeg.; *Cocos acrocomoides* Drude; *Cocos arechavaletana* Barb. Rodr.; *Cocos australis* Mart.; *Cocos datil* Drude & Griseb.; *Cocos geriba* Barb.Rodr.; *Cocos martiana* Drude; *Cocos plumosa* Hook.f.; *Cocos romanzoffiana* Cham.; *Cocos romanzoffiana* Cham. var. *macropindo* Becc.

Palm.

Departmental distribution in Paraguay: Alto Paraná, Amambay, Canindeyú, Central, Cordillera, Guairá, Ñeembucú, Paraguarí.

Voucher: Cited in Vogt and Mereles 2005: 10.

#### ARISTOLOCHIACEAE

##### *Aristolochia fimbriata Cham., Linnaea 7: 210. 1832.

Syn.: *Aristolochia ciliata* Hook.; *Aristolochia ciliosa* Benth.; *Howardia fimbriata* (Cham.) Klotzsch

Herb.

Departmental distribution in Paraguay: Central, Itapúa, Ñeembucú, Paraguarí.

Voucher: *T. Rojas 12683* (LIL) (cited in Ahumada 2010: 30).

#### ASTERACEAE

##### *Acanthospermum hispidum DC., Prodr. (DC.) 5: 522. 1836.

Syn.: *Acanthospermum humile* DC. var. *hispidum* (DC.) Kuntze

Herb.

Departmental distribution in Paraguay: Alto Paraguay, Central, Concepción, Cordillera, Ñeembucú, Paraguarí, Presidente Hayes.

Voucher: *L. Bernardi 18505* (G) (http://www.ville-ge.ch/musinfo/bd/cjb/fdp/).

##### ►*Acmella decumbens* (Sm.) R.K.Jansen, Syst. Bot. Monogr. 8: 80. 1985.

Syn.: *Ceratocephalus decumbens* (Sm.) Kuntze; *Ceratocephalus decumbens* (Sm.) Kuntzevar. *macropoda* (DC.) Kuntze; *Rudbeckia decumbens* Sm.; *Spilanthes americana* (L.f.) Hieron. var. *stolonifera* (DC.) A.H.Moore; *Spilanthes arnicoides* DC. var. *macropoda* (DC.) Baker; *Spilanthes decumbens* (Sm.) A.H.Moore; *Spilanthes decumbens* var. *macropoda* (DC.) A.H.Moore; *Spilanthes helenioides* Hook. & Arn.; *Spilanthes macropoda* DC.; *Spilanthes stenophylla* Hook. & Arn.; *Spilanthes stolonifera* DC.

Herb.

Departmental distribution in Paraguay: Alto Paraguay, Ñeembucú.

Voucher: *M. Peña-Chocarro et al. 2220* (BM, CTES, FCQ, G, MO, SI).

##### ►*Aspilia pascalioides* Griseb., Symb. Fl. Argent. 191. 1879.

Syn.: *Wedelia pascalioides* (Griseb.) B.L.Turner

Herb.

Departmental distribution in Paraguay: Alto Paraná, Cordillera, Guairá, Misiones, Ñeembucú, Paraguarí, Presidente Hayes.

Voucher: *M. Peña-Chocarro et al. 1981-B* (FCQ)

##### ►*Aspilia silphioides* (Hook. & Arn.) Benth. & Hook.f., Gen. Pl. [Bentham & Hooker f.] 2(1): 372. 1873.

Syn.: *Gymnopsis helianthoides* DC.; *Leighia silphioides* Hook. & Arn.; *Wedelia silphioides* (Hook. & Arn.) B.L.Turner

Herb.

Departmental distribution in Paraguay: Amambay, Central, Cordillera, Ñeembucú, Presidente Hayes.

Voucher: *F. Mereles 9729* (FCQ).

##### *Baccharis microcephala* (Less.) DC., Prodr. (DC.) 5: 425. 1836.

Syn.: *Baccharis mendes-magalhaensis* Barreto, nom. nud.; *Baccharis microptera* Baker; *Molina microcephala* Less.

Shrub or subshrub.

Departmental distribution in Paraguay: Caaguazú, Caazapá, Central, Cordillera, Guairá, Ñeembucú, Paraguarí, San Pedro.

Voucher: *A.G. Schulz 7664* (CTES, G) (cited in Soria and Zardini 1995: 366).

##### *Baccharis phyteumoides* (Less.) DC., Prodr. (DC.) 5: 425. 1836.

Syn.: *Molina phyteumoides* Less.

Subshrub.

Departmental distribution in Paraguay: Ñeembucú.

Voucher: *S. Keel & L. Spinzi 1462* (FCQ) (cited in Soria and Zardini 1995: 367).

##### *Centratherum punctatum* Cass., Dict. Sci. Nat. (ed. 2) 7: 384. 1817.

Syn.: *Ampherephis aristata* Kunth; *Ampherephis intermedia* Link; *Ampherephis mutica* Kunth; *Ampherephis pilosa* Cass.; *Ampherephis pulchella* Cass.; *Ampherephis violacea* Schrank; *Baccharodes holtonii* (Baker) Kuntze; *Baccharodes muticum* (Kunth) Kuntze; *Baccharodes punctatum* (Cass.) Kuntze; *Baccharodes violaceum* (Schrank) Kuntze; *Baccharodes brachylepis* (Sch.Bip. ex Baker) Kuntze; *Baccharodes punctatum* (Cass.) Kuntze; *Centratherum aristatum* Cass.; *Centratherum brachylepis* Sch.Bip. ex Baker; *Centratherum brevispirum* Cass.; *Centratherum camporum* (Hassl.) Malme; *Centratherum camporum* (Hassl.) Malmevar. *longipes* (Hassl.) Malme; *Centratherum holtonii* Baker; *Centratherum intermedium* (Link) Less.; *Centratherum longispinum* Cass.; *Centratherum muticum* (Kunth) Less.; *Centratherum pulchellum* (Cass.) Steud.; *Centratherum punctatum* Cass. forma *brachyphyllum* Hassl.; *Centratherum punctatum* Cass. forma *foliosum* (Chodat) Hassl.; *Centratherum punctatum* Cass. subsp. *camporum* Hassl.; *Centratherum punctatum* Cass. var. *foliosa* Chodat; *Centratherum punctatum* Cass. var. *parviflorum* Baker; *Centratherum violaceum* (Schrank) Gleason; *Crantzia ovata* Vell.; *Spixia violacea* Schrank

Subshrub.

Departmental distribution in Paraguay: Alto Paraná, Amambay, Caaguazú, Caazapá, Canindeyú, Central, Concepción, Cordillera, Guairá, Itapúa, Misiones, Ñeembucú, Paraguarí.

Voucher: *A.G. Schulz 7963* (cited in Cabrera et al. 2009: 72).

##### ●*Cirsium vulgare* (Savi) Ten., Fl.Napoli 5: 209. 1836.

Syn.: *Carduus lanceolatus* L.; *Carduus vulgaris* Savi; *Cirsium lanceolatum* (L.) Scop.; *Cirsium vulgare* (Savi) Airy Shaw, comb. superfl.

Herb. Introduced.

Departmental distribution in Paraguay: Ñeembucú.

Voucher: *M. Peña-Chocarro et al. 2308* (BM, CTES, FCQ).

##### *Conyza primulifolia* (Lam.) Cuatrec. & Lourteig, Phytologia 58: 475. 1985.

Syn.: *Conyza chilensis* Spreng.; *Conyza chilensis* Spreng. var. *carnea* Chodat & Hassl.; *Conyza chilensis* Spreng. var. *guaranitica* Chodat & Hassl.; *Conyza myosotifolia* Kunth; *Conyza scabiosifolia* J.Remy; *Conyza yungasensis* Rusby; *Erigeron chilensis* (Spreng.) D.Don ex G.Don; *Inula primulifolia* Lam.; *Marsea chilensis* (Spreng.) Kuntze; *Marsea chilensis* (Spreng.) V.M.Badillo, nom. illeg.

Herb.

Departmental distribution in Paraguay: Alto Paraguay, Caaguazú, Canindeyú, Cordillera, Itapúa, Ñeembucú, Paraguarí, Presidente Hayes.

Voucher: Cited in Zuloaga et al. 2008: 1253.

##### *Egletes viscosa* (L.) Less., Syn. Gen. Compos. 252. 1832.

Syn.: *Cotula viscosa* L.

Herb.

Departmental distribution in Paraguay: Alto Paraguay, Ñeembucú, Presidente Hayes.

Voucher: *T. Meyer 15856* (LP) (cited in Soria and Zardini 1995: 373).

##### *Enydra anagallis Gardner, London J. Bot. 7: 409. 1848.

Herb.

Departmental distribution in Paraguay: Ñeembucú, Paraguarí, Presidente Hayes.

Voucher: *M.A. Walter 120* (BM).

##### *Eupatorium candolleanum* Hook. & Arn., Companion Bot. Mag. 1: 243. 1835.

Syn.: *Barrosoa candolleana* (Hook. & Arn.) R.M.King & H.Rob.; *Eupatorium candolleanum* Hook. & Arn. var. *lancifolium* Baker; *Eupatorium candolleanum* Hook. & Arn. var. *paranaensis* Baker; *Eupatorium palustre* (DC.) Baker var. *guaraniticum* Chodat, nom. illeg.; *Eupatorium palustre* (DC.) Baker var. *verbenaceum* Chodat

Herb.

Departmental distribution in Paraguay: Alto Paraguay, Canindeyú, Central, Concepción, Cordillera, Guairá, Itapúa, Ñeembucú, Paraguarí, Presidente Hayes, San Pedro.

Voucher: *A.G. Schulz 7949* (CTES) (cited in Cabrera et al. 1996: 57).

##### *Eupatorium catarium* Veldkamp, Gard. Bull. Singapore 51(1): 121. 1999.

Syn.: *Eupatorium clematideum* Griseb., nom. illeg.; *Eupatorium pauciflorum* auct. non Kunth; *Eupatorium urticifolium* L.f.; *Eupatorium urticifolium* L.f. var. *clematideum* (Griseb.) Hieron.; *Eupatorium urticifolium* L.f. var. *nanum* Hieron.; *Praxelis clematidea* (Griseb.) R.M.King & H.Rob.

Herb.

Departmental distribution in Paraguay: Alto Paraguay, Amambay, Caaguazú, Canindeyú, Central, Concepción, Cordillera, Guairá, Misiones, Ñeembucú, Paraguarí, Presidente Hayes, San Pedro.

Voucher: *T. Meyer 16044* (LIL) (cited in Cabrera et al. 1996: 65).

##### ►*Eupatorium hecatanthum* (DC.) Baker, Fl. Bras. (Martius) 6(2): 365. 1876.

Syn.: *Eupatorium populifolium* Hook. & Arn., nom. illeg.; *Hebeclinium hecatanthum* DC.; *Hebeclinium urolepis* DC.; *Urolepis hecatantha* (DC.) R.M.King & H.Rob.

Herb.

Departmental distribution in Paraguay: Alto Paraná, Amambay, Caaguazú, Caazapá, Canindeyú, Central, Concepción, Cordillera, Guairá, Misiones, Ñeembucú, Paraguarí, Presidente Hayes, San Pedro.

Voucher: *M. Peña-Chocarro et al. 2332* (BM, CTES, FCQ).

##### *Eupatorium hirsutum* Hook. & Arn., Companion Bot. Mag. 1: 239. 1835.

Syn.: *Chromolaena hirsuta* (Hook. & Arn.) R.M.King & H.Rob.; *Eupatorium congestum* Hook. & Arn. var. *hirsutum* (Hook. & Arn.) Cabrera; *Eupatorium congestum* Hook. & Arn. var. *truncatum* Hassl.; *Eupatorium hirsutum* Hook. & Arn. var. *alfa* Hook. & Arn.; *Eupatorium hirsutum* Hook. & Arn. var. *triseriale* Hassl.; *Eupatorium tozziifolium* DC. var. *subpetiolatum* Chodat

Subshrub.

Departmental distribution in Paraguay: Amambay, Caazapá, Canindeyú, Cordillera, Ñeembucú, Paraguarí.

Voucher: *T. Rojas 12634* (LIL, LP, SI) (cited in Cabrera et al. 1996: 83).

##### *Eupatorium kleinioides Kunth, Nov. Gen. Sp. (folio ed.) 4: 94. 1820.

Syn.: *Campuloclinium kleinioides* (Kunth) DC.; *Eupatorium kleinioides* Kunth var. *microcephalum* Chodat; *Eupatorium kleinioides* Kunth var. *subglabratum* Hieron.; *Eupatorium sanctopaulense* B.L.Rob. var. *subglabratum* (Hieron.) Malme; *Eupatorium subglabratum* (Hieron.) Cabrera & Vittet; *Praxelis kleinioides* (Kunth) Sch.Bip.

Herb.

Departmental distribution in Paraguay: Amambay, Canindeyú, Concepción, Ñeembucú.

Voucher: *R. Spichiger et al. 5226* (G) (http://www.ville-ge.ch/musinfo/bd/cjb/fdp/).

##### *Eupatorium macrocephalum* Less., Linnaea 5(1): 136. 1830.

Syn.: *Campuloclinium macrocephalum* (Less.) DC.; *Campuloclinium macrocephalum* (Less.) DC. var. *strigosum* DC.; *Chromolaena pratensis* Gardner; *Eupatorium denudatum* Chodat; *Eupatorium donianum* Hook. & Arn.; *Eupatorium macrocephalum* Less. var. *angustifolium* Baker; *Eupatorium macrocephalum* Less. var. *stigmatosum* (Chodat) Hassl.; *Eupatorium macrocephalum* Less. var. *strigosum* Baker; *Eupatorium stigmatosum* Chodat; *Eupatorium stigmatosum* Chodatvar. *subcalvatum* Chodat; *Eupatorium stigmatosum* Chodatvar. *violaceum* Chodat

Herb.

Departmental distribution in Paraguay: Alto Paraná, Alto Paraguay, Amambay, Caaguazú, Caazapá, Canindeyú, Central, Concepción, Cordillera, Guairá, Itapúa, Ñeembucú, Paraguarí, Presidente Hayes, San Pedro.

Voucher: *F. Mereles 9727* (FCQ).

##### ►*Eupatorium orbignyanum* Klatt, Neue Compos. 3. 1881.

Syn.: *Chromolaena densiflora* (Morong) R.M.King & H.Rob.; *Chromolaena orbignyana* (Klatt) R.M.King & H.Rob.; *Eupatorium densiflorum* Morong; *Eupatorium oxylepis* DC. var. *densiflorum* (Morong) Hassl.

Shrub.

Departmental distribution in Paraguay: Alto Paraguay, Amambay, Caazapá, Canindeyú, Central, Cordillera, Guairá, Ñeembucú, Paraguarí, Presidente Hayes, San Pedro.

Voucher: *F. Mereles 9732* (FCQ).

##### *Eupatorium ostenii B.L.Rob., Ostenia 356. 1953.

Syn.: *Praxelis ostenii* (B.L.Rob.) R.M.King & H.Rob.

Herb.

Departmental distribution in Paraguay: Amambay, Caaguazú, Central, Concepción, Cordillera, Guairá, Ñeembucú, Paraguarí, San Pedro.

Voucher: *R. Spichiger et al. 5209* (CTES, G, MO) (http://www.ville-ge.ch/musinfo/bd/cjb/fdp/).

##### *Eupatorium squarrulosum* Hook. & Arn., Companion Bot. Mag. 1: 239. 1835.

Syn.: *Chromolaena ascendens* (Sch.Bip. ex Baker) R.M.King & H.Rob.; *Chromolaena congesta* (Hook. & Arn.) R.M.King & H.Rob.; *Chromolaena squarrulosa* (Hook. & Arn.) R.M.King & H.Rob.; *Eupatorium ascendens* Sch.Bip. ex Baker; *Eupatorium ascendens* Sch.Bip. ex Baker var. *parcisetosum* B.L Rob.; *Eupatorium caaguazuense* Hieron. var. *nervosum* Chodat; *Eupatorium congestum* Hook. & Arn.; *Eupatorium liatridium* DC.; *Eupatorium tozziifolium* DC.

Subshrub.

Departmental distribution in Paraguay: Alto Paraná, Amambay, Caaguazú, Canindeyú, Concepción, Cordillera, Guairá, Misiones, Ñeembucú, Paraguarí, San Pedro.

Voucher: *M.M. Arbo 1834* (CTES) (cited in Cabrera et al. 1996: 186).

##### ►*Gymnocoronis spilanthoides* (Hook. & Arn.) DC. var. *subcordata* (DC.) Baker, Fl. Bras. (Martius) 6(2): 184. 1876.

Syn.: *Gymnocoronis subcordata* DC.

Herb.

Departmental distribution in Paraguay: Alto Paraguay, Amambay, Central, Cordillera, Ñeembucú, Paraguarí, Presidente Hayes.

Voucher: *M.A. Walter 113* (BM).

##### *Hypochaeris chillensis (Kunth) Britton, Bull. Torrey Bot. Club 19(12): 371. 1892.

Syn.: *Achyrophorus brasiliensis* Gardner, nom. illeg.; *Achyrophorus chillensis* (Kunth) Sch.Bip.; *Achyrophorus chillensis* (Kunth) Sch.Bip.; *Achyrophorus sagittatus* Phil.; *Achyrophorus selloi* Sch.Bip.; *Apargia chillensis* Kunth; *Hypochaeris brasiliensis* (Less.) Benth. & Hook.f. ex Griseb.; *Hypochaeris brasiliensis* (Less.) Benth. & Hook.f. ex Griseb. var. *chacoensis* Hassl.; *Hypochaeris brasiliensis* (Less.) Benth. & Hook.f. ex Griseb. var. *sulfurea* Kuntze; *Hypochaeris brasiliensis* (Less.) Benth. & Hook.f. ex Griseb. var. *tweediei* (Hook & Arn.) Baker; *Hypochaeris chillensis* (Kunth) Hieron.; *Hypochaeris tweediei* (Hook. & Arn.) Cabrera; *Leontodon chillense* (Kunth) DC.; *Porcellites brasiliensis* Less.; *Seriola brasiliensis* (Less.) Less.; *Seriola tweediei* Hook. & Arn.

Herb.

Departmental distribution in Paraguay: Amambay, Caaguazú, Canindeyú, Guairá, Misiones, Ñeembucú, Paraguarí.

Voucher: *M. Peña-Chocarro et al. 2207* (FCQ).

##### *Mikania periplocifolia Hook. & Arn., Companion Bot. Mag. 1: 243. 1835.

Syn.: *Mikania araucana* Phil.; *Mikania auricularis* Griseb.; *Mikania niederleinii* Hieron.; *Mikania scandens* (L.) Willd. subsp. *paraguayensis* Malme; *Mikania scandens* (L.) Willd. var. *periplocifolia* (Hook. & Arn.) Baker; *Mikania scandens* (L.) Willd. var. *sagittifolia* Hassl.

Climber.

Departmental distribution in Paraguay: Alto Paraguay, Canindeyú, Central, Cordillera, Ñeembucú, Paraguarí, Presidente Hayes.

Voucher: *R. Spichiger et al. 5275* (CTES, G, MO) (http://www.ville-ge.ch/musinfo/bd/cjb/fdp/).

##### *Pacourina edulis* Aubl., Hist. Pl. Guiane 2: 800. 1775.

Syn.: *Pacourinopsis dentata* Cass.; *Pacourinopsis edulis* Aubl. var. *spinosissima* Britton; *Pacourinopsis integrifolia* Cass.

Herb.

Departmental distribution in Paraguay: Alto Paraguay, Central, Concepción, Cordillera, Ñeembucú, Paraguarí, Presidente Hayes, San Pedro.

Voucher: *M.A. Walter 114* (BM).

##### ►*Picrosia longifolia* D.Don, Trans. Linn. Soc. London 16: 184. 1830.

Syn.: *Picrosia australis* Decne.; *Picrosia longifolia* D.Don var. *angustissima* Kuntze; *Prenanthes subdentata* Hook.; *Psilopogon albiflorus* Phil.; *Tragopogon fritillarioides* Less.

Herb.

Departmental distribution in Paraguay: Alto Paraguay, Central, Ñeembucú, Presidente Hayes, San Pedro.

Voucher: *M. Peña-Chocarro et al. 1990* (BM, CTES, FCQ, MO).

##### *Podocoma notobellidiastrum* (Griseb.) G.L.Nesom, Phytologia 76: 112. 1994.

Syn.: *Baccharidastrum notobellidastrum* (Griseb.) Herter; *Conyza notobellidastrum* Griseb.; *Conyza notobellidastrum* Griseb. var. *oblongifolia* Griseb.; *Erigeron notobellidastrum* (Griseb.) Blake; *Erigeron pauciflorus* Less., nom. nud.; *Marsea notobellidiastrum* (Griseb.) Kuntze

Herb.

Departmental distribution in Paraguay: Alto Paraná, Amambay, Caazapá, Canindeyú, Cordillera, Guairá, Ñeembucú, Paraguarí, San Pedro.

Voucher: Cited in Zuloaga et al. 2008: 1433.

##### *Pseudogynoxys benthamii* Cabrera, Brittonia 7(2): 56. 1950.

Syn.: *Pseudogynoxys cabrerae* H.Rob. & Cuatrec.; *Senecio benthamii* Baker, nom. illeg.

Shrub.

Departmental distribution in Paraguay: Alto Paraná, Central, Cordillera, Guairá, Itapúa, Misiones, Ñeembucú, Paraguarí, Presidente Hayes, San Pedro.

Voucher: *A.G. Schulz 7780* (CTES, G) (cited in Cabrera et al. 2009: 28).

##### ►*Pterocaulon lorentzii* Malme, Bih. Kongl. Svenska Vetensk.-Akad. Handl. 27(3-13): 22. 1901.

Syn.: *Pterocaulon malmeanum* Chodat; *Pterocaulon virgatum* (L.) DC. forma *angustifolia* Arechav.; *Pterocaulon virgatum* (L.) DC. forma *spicata* Arechav.

Herb.

Departmental distribution in Paraguay: Amambay, Central, Cordillera, Guairá, Ñeembucú, Presidente Hayes.

Voucher: *M. Peña-Chocarro et al. 2326* (BM, FCQ).

##### ►*Sphagneticola brachycarpa* (Baker) Pruski, Novon 6(4): 411. 1996.

Syn.: *Aspilia callosa* Chodat; *Aspilia silphioides* (Hook. & Arn.) Benth. & Hook. forma *parvifolia* Chodat; *Stemmodontia brachycarpa* (Baker) Morong; *Thelechitonia brachycarpa* (Baker) H.Rob. & Cuatrec.; *Wedelia brachycarpa* Baker; *Wedelia callosa* (Chodat) B.L.Turner; *Wedelia pilosa* Baker; *Wedelia pilosa* Bakervar. *brachycarpa* Hassl.

Herb.

Departmental distribution in Paraguay: Central, Concepción, Cordillera, Itapúa, Misiones, Ñeembucú, Paraguarí, Presidente Hayes.

Voucher: *M. Peña-Chocarro et al. 1985* (BM, CTES, FCQ, G, MO).

##### ►*Senecio grisebachii* Baker, Fl. Bras. (Martius) 6(3): 313. 1884.

Herb.

Departmental distribution in Paraguay: Alto Paraguay, Amambay, Caaguazú, Caazapá, Canindeyú, Central, Cordillera, Guairá, Itapúa, Misiones, Paraguarí, Presidente Hayes, San Pedro.

Voucher: *C. Vogt 154* (CTES, FACEN, FCQ).

##### *Tessaria integrifolia* Ruiz & Pav., Syst. Veg. Fl. Peruv. Chil. 213. 1798.

Syn.: *Gynheteria incana* Spreng., pro parte; *Tessaria ambigua* DC. var. *uniflora* Cuatrec.; *Tessaria dentata* Ruiz & Pav.; *Tessaria legitima* DC.

Tree or shrub.

Departmental distribution in Paraguay: Alto Paraguay, Central, Ñeembucú, Presidente Hayes.

Voucher: *M. Peña-Chocarro et al. 2281* (BM, CTES, FCQ).

##### ►*Vernonia chamaedrys* Less., Linnaea 4: 259. 1829.

Syn.: *Cacalia chamaedrys* (Less.) Kuntze; *Vernonanthura chamaedrys* (Less.) H.Rob.; *Vernonia ilex* Chodat

Subshrub.

Departmental distribution in Paraguay: Amambay, Caaguazú, Central, Concepción, Cordillera, Guairá, Misiones, Ñeembucú, Paraguarí, Presidente Hayes, San Pedro.

Voucher: *M. Peña-Chocarro et al. 2307* (BM, CTES, FCQ, G, MO).

##### ►*Vernonia incana* Less., Linnaea 4: 277. 1829.

Syn.: *Cacalia incana* (Less.) Kuntze; *Vernonia inmunis* Griseb.

Herb.

Departmental distribution in Paraguay: Alto Paraná, Amambay, Central, Ñeembucú, Paraguarí, Presidente Hayes.

Voucher: *M.A. Walter 117* (BM).

##### *Vernonia pseudoincana* (Hieron.) Cabrera, Candollea 54: 110. 1999.

Syn.: *Cacalia rubricaulis* (Bonpl.) Kuntze var. *pseudoincana* (Hieron.) Kuntze; *Vernonia rubricaulis* Bonpl. var. *australis* auct. non Hieron.; *Vernonia rubricaulis* Bonpl. var. *denudata* Baker; *Vernonia rubricaulis* Bonpl. var. *latifolia* Less.; *Vernonia rubricaulis* Bonpl. var. *pseudoincana* Hieron.; *Vernonia salicifolia* Gillies ex Hook. & Arn.

Subshrub.

Departmental distribution in Paraguay: Central, Ñeembucú.

Voucher: *R. Spichiger et al. 5239* (G) (cited in Cabrera et al. 2009: 225).

##### *Vernonia remotiflora* Rich., Actes Soc. Hist. Nat. Paris 1: 112. 1792.

Syn.: *Lepidaploa remotiflora* (Rich.) H.Rob.; *Vernonia hirtiflora* Sch.Bip. ex Baker; *Vernonia lithospermoides* Baker; *Vernonia sessiliflora* Willd. ex Less.; *Vernonia tricholepis* DC.

Herb.

Departmental distribution in Paraguay: Alto Paraguay, Amambay, Caaguazú, Caazapá, Canindeyú, Central, Concepción, Cordillera, Guairá, Itapúa, Misiones, Ñeembucú, Paraguarí, Presidente Hayes, San Pedro.

Voucher: *M. Peña-Chocarro et al. 2210* (FCQ).

##### ►*Vernonia rubricaulis* Bonpl., Pl. Aequinoct. [Humboldt & Bonpland] 2: 66. 1809.

Syn.: *Cacalia rubricaulis* (Bonpl.) Kuntze; *Lessingianthus rubricaulis* (Bonpl.) H.Rob.; *Vernonia chromolepis* Gardner; *Vernonia intermedia* DC. var. *ramosior* DC.; *Vernonia linearis* D.Don ex Hook. & Arn., nom. nud.; *Vernonia rubricaulis* Bonpl. var. *australis* Hieron.; *Vernonia rubricaulis* Bonpl. var. *bonplandiana* Less.

Herb. Pastures on saturated soil.

Departmental distribution in Paraguay: Alto Paraguay, Amambay, Canindeyú, Central, Misiones, Ñeembucú, Paraguarí, Presidente Hayes, San Pedro.

Voucher: *S. Keel & L. Spinzi 1477* (FCQ).

##### *Vernonia scorpioides* (Lam.) Pers., Syn. Pl. (Persoon) 2: 404. 1807.

Syn.: *Cacalia scorpiodes* (Lam.) Kuntze; *Chrysocoma repanda* Vell.; *Conyza scorpioides* Lam.; *Cyrtocymura scorpioides* (Lam.) Cass.; *Vernonia arborescens* (L.) Sw. var. *corrientensis* Hieron.; *Vernonia centriflora* Link & Otto; *Vernonia flavescens* Less.; *Vernonia lanuginosa* Gardner; *Vernonia longeracemosa* Mart. ex DC.; *Vernonia subrepanda* Pers.; *Vernonia tournefortioides* Kunth

Shrub.

Departmental distribution in Paraguay: Canindeyú, Cordillera, Ñeembucú, Paraguarí.

Voucher: *C. Vogt 160* (CTES, FACEN, FCQ).

#### BEGONIACEAE

##### *Begonia cucullata Willd. var. *cucullata*, Sp. Pl., ed. 4 [Willdenow] 4(1): 414. 1805.

Syn.: *Begonia cucullata* Willd. var. *hookeri* (A.DC.) L.B.Sm. & B.G.Schub.; *Begonia hookeri* Sw.; *Begonia paludicola* C.DC.; *Begonia semperflorens* Link & Otto; *Begonia semperflorens* Link & Otto var. *hookeri* A.DC.

Herb.

Departmental distribution in Paraguay: Caaguazú, Caazapá, Canindeyú, Cordillera, Ñeembucú, Presidente Hayes.

Voucher: *J. De Egea et al. 648* (BM, CTES, FCQ, G, MO, PY, UNR).

##### *Begonia cucullata Willd. var. *arenosicola* (C.DC.) L.B.Sm. & B.G.Schub., Darwiniana 5: 106. 1941.

Syn.: *Begonia arenosicola* C.DC.

Herb.

Departmental distribution in Paraguay: Itapúa, Ñeembucú.

Voucher: *A.G. Schulz 7725* (G) (cited in Ramella 2008: 163).

#### BIGNONIACEAE

##### *Anemopaegma flavum* Morong, Ann. New York Acad. Sci. 7: 188. 1893.

Syn.: *Anemopaegma sylvestre* S.Moore; *Anemopaegma symmetricum* Rusby

Climber.

Departmental distribution in Paraguay: Central, Cordillera, Guairá, Ñeembucú.

Voucher: Cited in Zuloaga et al. 2008: 1585.

##### *Arrabidaea corallina* (Jacq.) Sandwith, Kew Bull. 8: 460.1953 [1954].

Syn.: *Adenocalymma friesianum* Kraenzl.; *Arrabidaea acuminata* (J.R.Johnst.) Urb.; *Arrabidaea barquisimetensis* Pittier; *Arrabidaea guaricensis* Pittier; *Arrabidaea lenticellosa* Pittier; *Arrabidaea obliqua* (Kunth) Bureau; *Arrabidaea obliqua* var. *hirsuta* (DC.) Dugand; *Arrabidaea obovata* DC.; *Arrabidaea ovalifolia* Pittier; *Arrabidaea praecox* Hassl.; *Arrabidaea rhodantha* Bureau & K.Schum.; *Arrabidaea rhodantha* Bureau & K.Schum. forma *glabrescens* Sprague; *Arrabidaea rhodantha* Bureau & K.Schum. forma *mollis* (Sprague) Hassl.; *Arrabidaea rhodantha* Bureau & K.Schum. forma *puberula* Hassl.; *Arrabidaea rhodantha* Bureau & K.Schum. forma *subglabra* Hassl.; *Arrabidaea rhodantha* Bureau & K.Schum. var. *elliptica* Sprague; *Arrabidaea rhodantha* Bureau & K.Schum. var. *genuina* Hassl., nom. illeg.; *Arrabidaea rhodantha* Bureau & K.Schum. var. *induta* Hassl.; *Arrabidaea rhodantha* Bureau & K.Schum. var. *oxyphylla* Sprague & Sandwith; *Arrabidaea rhodantha* Bureau & K.Schum. var. *praecox* (Hassl.) Hassl.; *Arrabidaea rhodantha* Bureau & K.Schum. var. *typica* Sprague, nom. illeg.; *Arrabidaea rotundata* (DC.) Bureau ex K.Schum.; *Arrabidaea sickiana* J.C.Gomes; *Arrabidaea spraguei* Pittier; *Bignonia acuminata* J.R.Johnst.; *Bignonia balbisiana* DC.; *Bignonia balbisiana* DC. var. *glabra* DC.; *Bignonia balbisiana* DC. var. *hirsuta* DC.; *Bignonia boliviana* Rusby; *Bignonia columbiana* Morong; *Bignonia corallina* Jacq.; *Bignonia dichotoma* Jacq.; *Bignonia glabrata* Kunth; *Bignonia hibiscifolia* Cham.; *Bignonia obliqua* Kunth; *Bignonia rotundata* DC.; *Bignonia villosa* Bertero ex Spreng.; *Cremastus balbisianus* (DC.) Miers; *Cuspidaria hibiscifolia* (Cham.) Bureau; *Panterpa dichotoma* (Jacq.) Miers; *Petastoma obliqum* (Kunth) Miers; *Piriadacus hibiscifolius* (Cham.) Pichon; *Tabebuia chapadensis* S.Moore

Climber.

Departmental distribution in Paraguay: Alto Paraguay, Amambay, Central, Concepción, Cordillera, Guairá, Itapúa, Misiones, Ñeembucú, Paraguarí, Presidente Hayes, San Pedro.

Voucher: *J. De Egea & R. Elsam 812* (BM, CTES, FCQ, MO).

Note: This species is being transferred to the genus *Fridericia* (basionym *Bignonia dichotoma* Jacq.) as a result of phylogenetic study, see Lohmann (in press).

##### ►*Bignonia binata* Thunb., Pl. Bras. 3: 35. 1821.

Syn.: *Adenocalymma ocositense* Donn.Sm.; *Arrabidaea schumanniana* Huber; *Bignonia eximia* Morong; *Bignonia noterophila* DC.; *Bignonia purpurea* Lodd. ex Hook.f.; *Bignonia umbellulata* DC.; *Clytostoma binatum* (Thunb.) Sandwith; *Clytostoma elegans* Standl.; *Clytostoma isthmicum* Pittier; *Clytostoma noterophilum* (DC.) Bureau & K.Schum.; *Petastoma multiglandulosum* Kraenzl.

Climber.

Departmental distribution in Paraguay: Alto Paraná, Alto Paraguay, Caaguazú, Caazapá, Canindeyú, Guairá, Itapúa, Misiones, Ñeembucú, Paraguarí, San Pedro.

Voucher: *M. Peña-Chocarro et al. 2324* (BM, CTES, FCQ, G, MO).

##### *Dolichandra cynanchoides* Cham., Linnaea 7: 658. 1832.

Syn.: *Bignonia coccinea* Vell.; *Dolichandra cynanchoides* Cham. var. *latifolia* Hassl.; *Macfadyena coccinea* (Vell.) Miers; *Macfadyena cynanchoides* (Cham.) Morong; *Macfadyena cynanchoides* (Cham.) Morong var. *latifolia* Hassl.; *Spathodea dolichandra* Steud.

Climber.

Departmental distribution in Paraguay: Alto Paraguay, Amambay, Central, Concepción, Cordillera, Guairá, Misiones, Ñeembucú, Paraguarí, Presidente Hayes, San Pedro.

Voucher: *M. Vera 215 et al.* (BM, CTES, FCQ).

##### *Dolichandra uncata* (Andrews) L.G.Lohmann, Nuevo Cat. Fl. Vasc. Venezuela 273. 2008.

Syn.: *Bignonia pachyptera* DC., nom. nud.; *Bignonia uncata* Andrews; *Bignonia uncata* Sims, nom. illeg.; *Bignonia uncinata* G.Mey.; *Dolichandra fenzliana* Miq.; *Doxantha uncata* (Andrews) Miers; *Macfadyena fenzliana* (Miq.) Miq.; *Macfadyena guatemalensis* S.F.Blake; *Macfadyena hassleri* Sprague; *Macfadyena hispida* (DC.) Seem.; *Macfadyena mollis* (Sond.) Seem.; *Macfadyena pubescens* S.Moore; *Macfadyena uncata* (Andrews) Sprague & Sandwith; *Macfadyena uncinata* (G.Mey.) A.DC.; *Macfadyena unculata* K.Schum.; *Pachyptera puberula* DC.; *Spathodea hispida* DC.; *Spathodea mollis* Sond.; *Spathodea uncata* (Andrews) Spreng.; *Spathodea uncinata* (G.Mey.) Spreng.

Climber.

Departmental distribution in Paraguay: Alto Paraná, Alto Paraguay, Amambay, Caaguazú, Caazapá, Canindeyú, Central, Concepción, Cordillera, Guairá, Itapúa, Misiones, Ñeembucú, Paraguarí, Presidente Hayes.

Voucher: Cited in Zuloaga et al. 2008: 1602.

##### ►*Dolichandra unguis-cati* (L.) L.G.Lohmann, Nuevo Cat. Fl. Vasc. Venezuela 273. 2008.

Syn.: *Batocydia exoleta* Mart. ex DC.; *Batocydia unguis* Mart. ex DC., nom. nud.; *Batocydia unguis-cati* (L.) Mart. ex Britton, nom. illeg.; *Bignonia acutistiopula* Schltdl.; *Bignonia californica* Brandegee; *Bignonia catharinensis* Schenk; *Bignonia dasyonyx* S.F.Blake; *Bignonia exoleta* Vell.; *Bignonia gracilis* Lodd.; *Bignonia inflata* Griseb.; *Bignonia lanuginosa* Hemsl.; *Bignonia pseudounguis* Desf.; *Bignonia triantha* DC.; *Bignonia tweediana* Lindl.; *Bignonia unguis* L.; *Bignonia unguis-cati* L.; *Bignonia unguis-cati* L. var. *exoleta* (Vell.) Sprague; *Bignonia unguis-cati* L. var. *gracilis* (Lodd.) DC.; *Bignonia unguis-cati* L. var. *guatemalensis* K.Schum. & Loes.; *Bignonia unguis-cati* L. var. *radicans* DC.; *Bignonia unguis-cati* L. var. *serrata* Bureau & K.Schum.; *Bignonia vespertilia* Barb.Rodr.; *Dolichandra kohautiana* C.Presl; *Doxantha acutistipula* (Schltdl.) Miers; *Doxantha adunca* Miers; *Doxantha chelophora* Miers, nom. nud.; *Doxantha dasyonyx* (S.F.Blake) S.F.Blake; *Doxantha exoleta* (Vell.) Miers; *Doxantha mexicana* Miers, nom. nud.; *Doxantha praesignis* Miers, nom. nud.; *Doxantha radicans* (DC.) Miers; *Doxantha serrulata* Miers, nom. nud.; *Doxantha tenuicula* Miers, nom. nud.; *Doxantha torquata* Miers, nom. nud.; *Doxantha tweediana* (Lindl.) Miers; *Doxantha unguis* (L.) Miers; *Doxantha unguis* var. *microphylla* Chodat; *Doxantha unguis-cati* (L.) Miers; *Doxantha unguis-cati* (L.) Miers var. *exoleta* (Vell.) Fabris; *Macfadyena unguis-cati* (L.) A.H.Gentry; *Microbignonia auristellae* Kraenzl.

Climber.

Departmental distribution in Paraguay: Alto Paraná, Alto Paraguay, Amambay, Caaguazú, Caazapá, Canindeyú, Central, Concepción, Cordillera, Guairá, Itapúa, Misiones, Ñeembucú, Paraguarí, Presidente Hayes, San Pedro.

Voucher: *J. De Egea & R. Elsam 802* (BM, CTES, FCQ, MO).

##### *Handroanthus heptaphyllus* (Vell.) Mattos, Loefgrenia 50: 2. 1970.

Syn.: *Bignonia heptaphylla* Vell.; *Handroanthus avellanedae* (Lorentz ex Griseb.) Mattos var. *paullensis* (Toledo) Mattos; *Handroanthus eximus* (Miq.) Mattos; *Tabebuia avellanedae* Lorentz ex Griseb. var. *paulensis* Toledo; *Tabebuia eximia* (Miq.) Sandwith; *Tabebuia heptaphylla* (Vell.) Toledo; *Tabebuia impetiginosa* (Mart. ex DC.) Standl. var. *lepidota* (Bureau) Toledo; *Tecoma curialis* Saldanha; *Tecoma eximia* Miq.; *Tecoma impetiginosa* Mart. ex DC. var. *lepidota* Bureau; *Tecoma ipe* (Mart. ex K.Schum.) Standl.; *Tecoma ipe* (Mart. ex K.Schum.) Standl. var. *desinens* Sprague; *Tecoma ipe* (Mart. ex K.Schum.) Standl. forma *glabra* Sprague; *Tecoma ipe* (Mart. ex K.Schum.) Standl. forma *grandiflora* Sprague; *Tecoma ipe* (Mart. ex K.Schum.) Standl. forma *parviflora* Sprague

Tree.

Departmental distribution in Paraguay: Alto Paraná, Alto Paraguay, Amambay, Canindeyú, Central, Concepción, Cordillera, Guairá, Misiones, Ñeembucú, Paraguarí, Presidente Hayes, San Pedro.

Voucher: *Cited in Zuloaga et al. 2008: 1619* (as *Tabebuia heptaphylla*).

##### *Handroanthus pulcherrimus* (Sandwith) S.Grose, Syst. Bot. 32(5): 666. 2007.

Syn.: *Tabebuia pulcherrima* Sandwith; *Tecoma petropolitana* Glaz., nom. nud.

Tree.

Departmental distribution in Paraguay: Alto Paraná, Caaguazú, Caazapá, Itapúa, Misiones, Ñeembucú.

Voucher: *L. Bernardi 20498* (BM, G).

##### *Pithecoctenium cynanchoides* DC., Prodr. (DC.) 9: 195. 1845.

Syn.: *Anemopaegma clematideum* Griseb.; *Bignonia baclei* DC.; *Pithecoctenium clematideum* (Griseb.) Griseb.; *Pithecoctenium cynanchoides* DC. var. *pellucidum* Hassl.

Climber.

Departmental distribution in Paraguay: Alto Paraguay, Ñeembucú, Presidente Hayes.

Voucher: *J. De Egea & R. Elsam 803* (BM, CTES, FCQ, G, MO).

Note: This species is being transferred to the genus *Amphilophium* as a result of phylogenetic study, see Lohmann (in press).

##### ►*Tabebuia aurea* (Silva Manso) Benth. & Hook.f. ex S.Moore, Trans. Linn. Soc. London, Bot. 4(3): 423. 1895.

Syn.: *Bignonia aurea* Silva Manso; *Bignonia squamellulosa* DC., nom. nud.; *Handroanthus caraiba* (Mart.) Mattos; *Handroanthus leucophlaeos* (Mart. ex DC.) Mattos; *Tabebuia argentea* (Bureau & K.Schum.) Britton; *Tabebuia caraiba* (Mart.) Bureau; *Tabebuia suberosa* Rusby; *Tecoma argentea* Bureau & K.Schum.; *Tecoma argentea* Bureau & K.Schum. forma *parviflora* Hassl.; *Tecoma aurea* (Silva Manso) DC.; *Tecoma caraiba* Mart.; *Tecoma caraiba* Mart. var. *grandiflora* Hassl.; *Tecoma caraiba* Mart. var. *squamellulosa* (DC.) Bureau & K.Schum.; *Tecoma leucophlaeos* Mart. ex DC.; *Tecoma squamellulosa* DC.; *Tecoma trichocalycina* DC.

Tree.

Departmental distribution in Paraguay: Alto Paraguay, Amambay, Canindeyú, Concepción, Cordillera, Ñeembucú, Presidente Hayes.

Voucher: *M. Vera et al. 219* (BM, CTES, FCQ).

##### *Tabebuia nodosa* (Griseb.) Griseb., Abh. Königl. Ges. Wiss. Göttingen 24: 258. 1879.

Syn.: *Bignonia morongii* Britton; *Tabebuia nodosa* (Griseb.) Griseb. var. *parviflora* Griseb.; *Tecoma nodosa* Griseb.

Tree.

Departmental distribution in Paraguay: Alto Paraguay, Amambay, Central, Concepción, Ñeembucú, Paraguarí, Presidente Hayes.

Voucher: *M. Peña-Chocarro et al. 2288* (BM, CTES).

#### BORAGINACEAE

##### ►*Cordia americana* (L.) Gottschling & J.S.Mill., Syst. Bot. 31(2): 364. 2006.

Syn.: *Cordia patagonula* Aiton; *Patagonula alba* Rojas Acosta, nom. nud.; *Patagonula americana* L.; *Patagonula americana* L. var. *glabra* Cham.; *Patagonula americana* L. var. *hirsuta* Fresen; *Patagonula australis* Salisb.; *Patagonula fuscata* Rojas Acosta, nom. nud.; *Patagonula glabra* (Cham.) Miers; *Patagonula tweediana* Miers

Tree.

Departmental distribution in Paraguay: Alto Paraná, Amambay, Caazapá, Canindeyú, Cordillera, Ñeembucú, Paraguarí, Presidente Hayes.

Voucher: *M. Peña-Chocarro et al. 2235* (BM, CTES, FCQ, G, MO, PY, SI, UNR).

##### *Cordia ecalyculata Vell., Fl. Flumin. 96. 1829.

Syn.: *Cordia leptocaula* Fresen.; *Cordia salicifolia* Cham.

Tree.

Departmental distribution in Paraguay: Alto Paraná, Alto Paraguay, Caazapá, Canindeyú, Central, Cordillera, Guairá, Ñeembucú, Paraguarí.

Voucher: *L. Bernardi 20493* (G) (Ramella 2008: 175).

##### *Heliotropium elongatum Hoffm. ex Roem. & Schult., Syst. Veg. ed. 15 bis [Roemer & Schultes] 4: 736. 1819.

Syn.: *Heliotropium elongatum* Hoffm. ex Roem. & Schult. var. *genuinum* I.M.Johnst., nom. illeg.

Herb or subshrub.

Departmental distribution in Paraguay: Alto Paraguay, Central, Cordillera, Guairá, Ñeembucú, Paraguarí.

Voucher: *L. Bernardi 18507* (BM, G).

##### *Heliotropium indicum L., Sp. Pl. 2: 130. 1753.

Syn.: *Heliophytum indicum* (L.) DC.

Herb.

Departmental distribution in Paraguay: Alto Paraguay, Misiones, Ñeembucú, Presidente Hayes.

Voucher: *F. Mereles 9726* (FCQ).

##### ►*Heliotropium procumbens* Mill., Gard. Dict., ed. 8. Heliotropium no. 10. 1768.

Syn.: *Heliotropium inundatum* Sw.; *Heliotropium inundatum* Sw. forma *elliptica* Chodat.; *Heliotropium inundatum* Sw. forma *pusilla* Hassl.; *Heliotropium inundatum* Sw. var. *chacoense* R.E.Fr.; *Heliotropium riparium* Mart. ex Chodat

Herb.

Departmental distribution in Paraguay: Alto Paraguay, Amambay, Canindeyú, Central, Concepción, Cordillera, Guairá, Ñeembucú, Paraguarí, Presidente Hayes, San Pedro.

Voucher: *F. Mereles 9725* (FCQ).

##### *Heliotropium transalpinum* Vell. var. *arcanum* Di Fulvio & Espinar, Bol. Soc. Argent. Bot. 39(1-2): 119. 2004.

Shrub.

Departmental distribution in Paraguay: Caaguazú, Misiones, Ñeembucú.

Voucher: *Reales 236* (LIL) (cited in Di Fulvio and Ariza Espinar 2004: 122).

##### *Heliotropium transalpinum* Vell. var. *transalpinum*, Fl. Flumin. 68. 1829.

Syn.: *Heliotropium monostachyum* Cham.; *Heliotropium tiaridioides* Cham. var. *schizocarpum* I.M.Johnst.

Shrub.

Departmental distribution in Paraguay: Alto Paraná, Caazapá, Canindeyú, Central, Concepción, Cordillera, Guairá, Itapúa, Ñeembucú, Paraguarí.

Voucher: *A.G. Schulz 7806* (LIL) (cited in Di Fulvio and Ariza Espinar 2004: 119).

##### *Tournefortia rubicunda Salzm. ex DC., Prodr. (DC.) 9: 536. 1845.

Shrub.

Departmental distribution in Paraguay: Alto Paraná, Alto Paraguay, Caazapá, Canindeyú, Central, Concepción, Cordillera, Ñeembucú, Paraguarí, Presidente Hayes.

Voucher: *L. Bernardi 20467* (G) (cited in Ramella 2008: 175).

##### *Tournefortia salzmannii DC., Prodr. (DC.) 9: 524. 1845.

Syn.: *Tournefortia salzmannii* DC. var. *blanchetiana* Chodat & Hassl.

Shrub.

Departmental distribution in Paraguay: Alto Paraguay, Amambay, Central, Cordillera, Ñeembucú, Paraguarí, Presidente Hayes.

Voucher: *J. De Egea & R. Elsam 796* (BM, CTES).

#### BROMELIACEAE

##### *Aechmea bromeliifolia (Rudge) Baker, Gen. Pl. [Bentham & Hooker f.] 3: 664. 1883.

Syn.: *Aechmea pulchra* (Beer) Mez; *Aechmea tinctoria* (Mart.) Mez; *Macrochordion bromeliifolium* (Rudge) Beer; *Tillandsia bromeliifolia* Rudge

Epiphytic herb.

Departmental distribution in Paraguay: Caazapá, Central, Cordillera, Guairá, Ñeembucú.

Voucher: *C. Vogt 162* (CTES, FCQ).

##### *Aechmea distichantha Lem. var. *schlumbergeri* E.Morren ex Mez, Fl. Bras. (Martius) 3(3): 343. 1892.

Syn.: *Aechmea grandiceps* (Griseb.) Mez; *Aechmea polystachya* (Vell.) Mez var. *longifolia* A.Cast.; *Chevaliera grandiceps* Griseb.; *Platyaechmea distichantha* (Lem.) L.B.Sm. & W.J.Kress var. *schlumbergeri* (E.Morren ex Mez) L.B.Sm. & W.J.Kress

Epiphytic herb.

Departmental distribution in Paraguay: Alto Paraguay, Canindeyú, Central, Concepción, Cordillera, Guairá, Ñeembucú, Paraguarí, Presidente Hayes.

Voucher: *C. Vogt & P. Ríos 122* (CTES, FACEN, FCQ).

##### *Bromelia balansae Mez, Fl. Bras. (Martius) 3(3): 191. 1891.

Syn.: *Bromelia argentina* Baker; *Bromelia laciniosa* auct. non Mart. ex Schult. f.; *Bromelia pinguin* auct. non L.

Herb.

Departmental distribution in Paraguay: Alto Paraná, Alto Paraguay, Amambay, Caazapá, Canindeyú, Central, Cordillera, Guairá, Itapúa, Ñeembucú, Paraguarí, San Pedro.

Voucher: Cited in Vogt and Mereles 2005: 10.

##### *Pseudananas sagenarius (Arruda) Camargo, Revist Agric. (Piracicaba) 14(7, 8): reprint page 4. 1939.

Syn.: *Ananas bracteatus* (Lindl.) Schult. var. *sagenarius* (Arruda) Bertoni; *Ananas macrodontes* E.Morren; *Ananas microcephalus* Bertoni; *Ananas sagenaria* (Arruda) Schult.f.; *Ananas sativus* Schult. var. *macrodontes* (Arruda) Bertoni; *Ananas sativus* Schult. var. *sagenarius* (Arruda) Bertoni; *Bromelia sagenaria* Arruda; *Pseudananas macrodontes* (E.Morren) Harms

Herb.

Departmental distribution in Paraguay: Alto Paraguay, Caaguazú, Canindeyú, Central, Concepción, Cordillera, Guairá, Ñeembucú, Paraguarí.

Voucher: *M. Peña-Chocarro et al. 2355* (BM, CTES, FCQ).

##### ►*Tillandsia bandensis* Baker, J. Bot. 25: 234. 1887.

Syn.: *Tillandsia bandensis* Baker var. *intermedia* Hassl.; *Tillandsia recurvata* (L.) L. var. *majuscula* Mez

Epiphytic herb.

Departmental distribution in Paraguay: Ñeembucú, Presidente Hayes.

Voucher: *J. De Egea et al. 715* (FCQ).

##### ►*Tillandsia didisticha* (E.Morren) Baker, J. Bot. 26: 16. 1888.

Syn.: *Anoplophytum didistichum* E.Morren; *Tillandsia crassifolia* Baker; *Tillandsia disticha* auct. non Kunth; *Tillandsia oranensis* Baker; *Tillandsia purpurea* Griseb., nom. illeg.; *Tillandsia vernicosa* A.Cast., nom. illeg.

Epiphytic herb.

Departmental distribution in Paraguay: Alto Paraguay, Concepción, Ñeembucú, Paraguarí.

Voucher: *C. Vogt 180* (FCQ).

##### ►*Tillandsia duratii* Vis. var. *saxatilis* (Hassl.) L.B.Sm., Phytologia 16: 79. 1968.

Syn.: *Tillandsia confusa* Hassl.; *Tillandsia confusa* Hassl. var. *minor* Hassl.; *Tillandsia confusa* Hassl. var. *saxatilis* Hassl.; *Tillandsia confusa* Hassl. var. *saxitilis* Hassl.; *Tillandsia decomposita* Baker; *Tillandsia duratii* Vis. var. *confusa* (Hassl.) L.B.Sm.; *Tillandsia tomentosa* N.E.Br.; *Tillandsia weddellii* Baker

Epiphytic herb.

Departmental distribution in Paraguay: Alto Paraguay, Amambay, Central, Cordillera, Ñeembucú, Presidente Hayes.

Voucher: *C. Vogt 157* (CTES, FACEN, FCQ).

##### *Tillandsia meridionalis Baker, J. Bot. 26: 15. 1888.

Syn.: *Tillandsia pulchella* Mez, nom. illeg.; *Tillandsia stricta* Mez, nom. illeg.; *Tillandsia stricta* Sol. ex Sims var. *paraguariensis* Hassl.

Epiphytic herb.

Departmental distribution in Paraguay: Alto Paraguay, Amambay, Central, Cordillera, Guairá, Ñeembucú, Paraguarí, Presidente Hayes.

Voucher: Cited in Vogt and Mereles 2005: 10.

##### *Tillandsia recurvata (L.) L., Sp. Pl. (ed. 2) 1: 410. 1762.

Syn.: *Diaphoranthema recurvata* (L.) Beer; *Renealmia recurvata* L.; *Tillandsia monostachys* Gillies ex Baker, nom. nud.; *Tillandsia recurvata* (L.) L. forma *argentea* André; *Tillandsia recurvata* (L.) L. forma *brevifolia* André; *Tillandsia recurvata* (L.) L. forma *caespitosa* André; *Tillandsia recurvata* (L.) L. forma *contorta* André; *Tillandsia recurvata* (L.) L. forma *elongata* André; *Tillandsia recurvata* (L.) L. forma *genuina* André, nom. illeg.; *Tillandsia recurvata* (L.) L. forma *major* André; *Tillandsia recurvata* (L.) L. forma *minor* André; *Tillandsia recurvata* (L.) L. forma *minuta* (Mez) A.Cast.; *Tillandsia recurvata* (L.) L. var. *ciliata* E.Morren ex Mez; *Tillandsia recurvata* var. *minuta* Mez

Epiphytic herb.

Departmental distribution in Paraguay: Alto Paraguay, Amambay, Caaguazú, Central, Concepción, Guairá, Misiones, Ñeembucú, Paraguarí, Presidente Hayes.

Voucher: Cited in Vogt and Mereles 2005: 10.

##### ►*Tillandsia tenuifolia* L., Sp. Pl. 1: 286. 1753.

Syn.: *Tillandsia astragaloides* Mez; *Tillandsia autumnalis* F.Muell.; *Tillandsia bicolor* auct. non Brongn.; *Tillandsia pulchella* Hook.; *Tillandsia pulchella* Hook. var. *rosea* Lindl.; *Tillandsia pulchella* Hook. var. *surinamensis* Mez; *Tillandsia rosea* Lindl.; *Tillandsia tenuifolia* L. var. *surinamensis* (Mez) L.B.Sm.

Epiphytic herb.

Departmental distribution in Paraguay: Alto Paraná, Alto Paraguay, Caazapá, Canindeyú, Guairá, Misiones, Ñeembucú, Paraguarí.

Voucher: *C. Vogt & Contreras 705* (CTES, FACEN, FCQ).

##### ►*Tillandsia tricholepis* Baker, J. Bot. 16: 237. 1878.

Syn.: *Tillandsia polytrichoides* E. Morren; *Tillandsia tricholepis* var. *argentea* Hassl.

Epiphytic herb.

Departmental distribution in Paraguay: Alto Paraguay, Amambay, Central, Concepción, Cordillera, Guairá, Misiones, Ñeembucú, Paraguarí, Presidente Hayes, San Pedro.

Voucher: *F. Mereles 9720* (FCQ).

##### *Tillandsia usneoides (L.) L., Sp. Pl. (ed. 2) 1: 411. 1762.

Syn.: *Renealmia usneoides* L.; *Tillandsia usneoides* (L.) L. forma *cretacea* Mez; *Tillandsia usneoides* (L.) L. forma *crispa* André; *Tillandsia usneoides* (L.) L. forma *ferruginea* André; *Tillandsia usneoides* (L.) L. forma *filiformis* André; *Tillandsia usneoides* (L.) L. forma *genuina* André, nom. illeg.; *Tillandsia usneoides* (L.) L. forma *longissima* André; *Tillandsia usneoides* (L.) L. forma *major* André; *Tillandsia usneoides* (L.) L. var. *cretacea* (Mez) Mez; *Tillandsia usneoides* (L.) L. var. *ferruginea* (André) Mez; *Tillandsia usneoides* (L.) L. var. *longissima* (André) Mez; *Tillandsia usneoides* (L.) L. var. *robusta* (Mez) Mez

Epiphytic herb.

Departmental distribution in Paraguay: Caaguazú, Caazapá, Concepción, Guairá, Itapúa, Ñeembucú, Presidente Hayes.

Voucher: *C. Vogt 133* (CTES, FCQ).

##### *Tillandsia vernicosa Baker, J. Bot. 25: 241. 1887.

Syn.: *Tillandsia drepanophylla* Baker

Epiphytic herb.

Departmental distribution in Paraguay: Alto Paraguay, Central, Concepción, Cordillera, Ñeembucú, Paraguarí, Presidente Hayes, San Pedro.

Voucher: *M. Peña-Chocarro et al. 2329* (BM, CTES, FCQ).

#### CABOMBACEAE

##### ►*Cabomba caroliniana* A.Gray var. *flavida* Orgaard, Nordic J. Bot. 11(2): 201. 1991.

Syn.: *Cabomba australis* Speg.

Aquatic herb.

Departmental distribution in Paraguay: Alto Paraguay, Ñeembucú, Presidente Hayes.

Voucher: *M. Peña-Chocarro et al. 2328* (BM, CTES, FCQ, G, MO, PY, SI).

#### CACTACEAE

##### ►*Cereus stenogonus* K.Schum., Monatsschr. Kakteenk. 9: 165. 1899.

Syn.: *Cereus dayami* Speg.; *Cereus roseiflorus* Speg.; *Piptanthocereus dayami* (Speg.) F.Ritter, nom. illeg.

Tree.

Departmental distribution in Paraguay: Alto Paraguay, Amambay, Central, Concepción, Ñeembucú, Paraguarí, San Pedro.

Voucher: *J. De Egea et al. 709* (FCQ).

##### ►*Epiphyllum phyllanthus* (L.) Haw., Syn. Pl. Succ. 197. 1812.

Syn.: *Cactus phyllantus* L.; *Epiphyllum phyllanthus* (L.) Haw. var. *paraguayense* (F.A.C.Weber) Backeb.; *Phyllocactus phyllanthus* (L.) Link; *Phyllocactus phyllanthus* (L.) Linkvar. *paraguayense* F.A.C.Weber

Epiphytic herb.

Departmental distribution in Paraguay: Alto Paraná, Amambay, Canindeyú, Central, Cordillera, Guairá, Itapúa, Ñeembucú, Paraguarí, San Pedro.

Voucher: *M. Vera 229 et al.* (FCQ).

##### ►*Harrisia martinii* (Labour.) Britton, Addisonia 2: 55. 1917.

Syn.: *Cereus martinii* Labour.; *Eriocereus martinii* (Labour.) Riccob.

Subshrub.

Departmental distribution in Paraguay: Concepción, Cordillera, Ñeembucú, Presidente Hayes.

Voucher: *J. De Egea et al. 710* (BM, CTES, FCQ, MO).

##### ►*Monvillea cavendishii* (Monv.) Britton & Rose, Cactaceae [Britton & Rose] 2: 21. 1920.

Syn.: *Cereus cavendishii* Monv.; *Cereus paxtonianus* Monv.; *Cereus rhodoleucanthus* K.Schum.; *Cereus saxicolus* Morong; *Praecereus saxicolus* (Morong) N.P.Taylor

Subshrub.

Departmental distribution in Paraguay: Alto Paraguay, Amambay, Central, Concepción, Cordillera, Guairá, Ñeembucú, Paraguarí, Presidente Hayes, San Pedro.

Voucher: *J. De Egea et al. 733* (BM, FCQ, MO).

##### ►*Opuntia anacantha* Speg. var. *retrorsa* (Speg.) R.Kiesling, Candollea 53(2): 475. 1998.

Syn.: *Opuntia retrorsa* Speg.; *Opuntia fuscolineata* Starmühler & Muncher; *Platyopuntia retrorsa* (Speg.) F. Ritter

Subshrub.

Departmental distribution in Paraguay: Alto Paraguay, Ñeembucú, Paraguarí, Presidente Hayes.

Voucher: *J. De Egea et al. 647* (FCQ).

##### ►*Opuntia elata* Salm-Dyck var. *cardiosperma* (K.Schum.) R.Kiesling, Fl. Entre Rios [Burkart & Bacigalupo] IVB: 412. 2005.

Syn.: *Opuntia cardiosperma* K.Schum.; *Opuntia chakensis* Speg.; *Opuntia delaetiana* (F.A.C.Weber) Vaupel; *Opuntia elata* Salm-Dyck var. *delaetiana* F.A.C.Weber

Shrub.

Departmental distribution in Paraguay: Alto Paraguay, Ñeembucú.

Voucher: *J. De Egea et al. 711* (FCQ).

##### *Rhipsalis cereuscula Haw., Philos. Mag. Ann. Chem. 7: 112. 1830.

Syn.: *Cereus saglionis* Lem.; *Rhipsalis penduliflora* N.E.Br.; *Rhipsalis saglionis* (Lem.) Otto

Epiphyte.

Departmental distribution in Paraguay: Alto Paraná, Amambay, Caaguazú, Caazapá, Canindeyú, Concepción, Guairá, Itapúa, Ñeembucú, Paraguarí, San Pedro.

Voucher: Cited in Vogt and Mereles 2005: 10.

##### *Rhipsalis* sp.

Epiphyte.

Voucher: *J. De Egea et al. 479* (FCQ).

#### CALYCERACEAE

##### *Acicarpha tribuloides Juss., Ann. Mus. Natl. Hist. Nat. 2: 348. 1803.

Syn.: *Acicarpha laxa* R.E.Fr.; *Acicarpha pinnatifida* Miers; *Acicarpha tribuloides* Juss. var. *dentata* Kuntze; *Acicarpha tribuloides* Juss. var. *pinnatifida* (Miers) Kuntze; *Cryptocarpha tribuloides* (Juss.) Cass.

Herb.

Departmental distribution in Paraguay: Cordillera, Guairá, Misiones, Ñeembucú, Paraguarí.

Voucher: *C. Vogt 142* (CTES, FACEN, FCQ).

#### CAMPANULACEAE

##### ►*Wahlenbergia linarioides* (Lam.) A.DC., Mongr. Campan. 158. 1830.

Syn.: *Campanula arida* Kunth; *Campanula chilensis* Molina; *Campanula linarioides* Lam.; *Wahlenbergia arida* (Kunth) Griseb.; *Wahlenbergia linarioides* (Lam.) A.DC. var. *arida* (Kunth) Griseb.

Herb.

Departmental distribution in Paraguay: Alto Paraná, Caaguazú, Caazapá, Central, Guairá, Ñeembucú, Paraguarí.

Voucher: *M. Peña-Chocarro et al. 2251* (FCQ).

#### CANNABACEAE

##### ►*Celtis iguanaea* (Jacq.) Sarg., Silva 7: 64. 1895.

Syn.: *Celtis aculeata* Sw.; *Celtis diffusa* Planch.; *Celtis glycycarpa* Mart. ex Miq.; *Celtis morifolia* Planch.; *Celtis pubescens* (Kunth) Spreng.; *Celtis spinosa* Spreng.; *Celtis spinosissima* (Wedd.) Miq.; *Celtis triflora* (Klotzsch) Miq.; *Mertensia aculeata* (Wedd.) Schult.; *Mertensia iguanaea* (Jacq.) Schult.; *Mertensia pubescens* Kunth; *Momisia aculeata* (Sw.) Klotzsch; *Momisia iguanaea* (Jacq.) Rose & Standl.; *Momisia pubescens* (Kunth) F.G.Dietr.; *Momisia spinosissima* Wedd.; *Momisia tarijensis* Wedd.; *Momisia triflora* Ruiz ex Klotzsch; *Rhamnus iguanaeus* Jacq.

Tree or shrub.

Departmental distribution in Paraguay: Alto Paraná, Caaguazú, Caazapá, Canindeyú, Concepción, Cordillera, Guairá, Itapúa, Ñeembucú, Paraguarí, Presidente Hayes.

Voucher: *M. Peña-Chocarro et al. 2291* (BM, CTES, FCQ, MO).

#### CANNACEAE

##### ►*Canna glauca* L., Sp. Pl. 1: 1. 1753.

Syn.: *Canna glauca* L. var. *angusta* J.W.Richardson; *Canna glauca* L. var. *rubro-lutea* Hook.; *Canna hassleriana* Kraenzl.; *Canna pedicellata* C.Presl; *Canna stolonifera* A.Dietr.

Herb.

Departmental distribution in Paraguay: Central, Misiones, Ñeembucú, Paraguarí, Presidente Hayes, San Pedro.

Voucher: *M. Peña-Chocarro et al. 2314* (BM, CTES).

##### *Canna indica L., Sp. Pl. 1: 1. 1753.

Syn.: *Canna achiras* Gillies ex Lindl.; *Canna amabilis* T.Koyama & Nb.Tanaka; *Canna brasiliensis* Link; *Canna coccinea* Mill.; *Canna compacta* Roscoe; *Canna edulis* Ker Gawl.; *Canna esculenta* Lodd., nom. nud.; *Canna indica* Ruiz & Pav., nom. superfl.; *Canna lanuginosa* Roscoe; *Canna limbata* Roscoe; *Canna plurituberosa* T.Koyama & Nb.Tanaka; *Canna rubricaulis* Link; *Canna speciosa* Roscoe ex Sims; *Canna variegatifolia* Ciciarelli; *Canna warszewiczii* A.Dietr.

Herb.

Departmental distribution in Paraguay: Alto Paraná, Ñeembucú, Paraguarí, Presidente Hayes, San Pedro.

Voucher: *R. Spichiger et al. 5317* (G) (cited in Ramella 2008: 247).

#### CAPPARACEAE

##### *Capparicordis tweediana* (Eichler) H.H.Iltis & X.Cornejo, Brittonia 59(3): 251. 2007.

Syn.: *Capparis tweediana* Eichler

Shrub.

Departmental distribution in Paraguay: Alto Paraguay, Central, Ñeembucú, Presidente Hayes.

Voucher: *M. Bernardi 18446* (BM, MO).

##### ►*Capparis flexuosa* (L.) L., Sp. Pl. (ed. 2) 1: 722. 1762.

Syn.: *Capparis cynophallophora* L.; *Morisonia flexuosa* L.

Tree.

Departmental distribution in Paraguay: Central, Concepción, Cordillera, Guairá, Ñeembucú, Paraguarí.

Voucher: *L. Bernardi 18468* (BM, G).

##### ►*Capparis retusa* Griseb., Abh. Königl. Ges. Wiss. Göttingen 24: 18. 1879.

Syn.: *Capparis cynophallophora* L. var. *cuneata* Malme; *Capparis cynophallophora* var. *retusa* (Griseb.) Kuntze

Shrub.

Departmental distribution in Paraguay: Alto Paraguay, Amambay, Central, Concepción, Ñeembucú, Paraguarí, Presidente Hayes.

Voucher: *J. De Egea et al. 726* (BM, CTES, FCQ, G, MO, PY, UNR).

##### ►*Cleome paludosa* Willd. ex Eichler, Fl. Bras. (Martius) 13(1): 255. 1865.

Syn.: *Cleome paludosa* Willd. ex Eichlervar. *brevipes* Hauman

Herb.

Departmental distribution in Paraguay: Alto Paraguay, Concepción, Ñeembucú, Presidente Hayes.

Voucher: *M. Peña-Chocarro et al. 2254* (BM, CTES, FCQ).

#### CARYOPHYLLACEAE

##### *Polycarpon suffruticosum Griseb., Abh. Königl. Ges. Wiss. Göttingen 19: 77. 1874.

Syn.: *Polycarpon anomalum* Hassl.; *Polycarpon australis* Britton; *Polycarpon suffruticosum* Griseb. var. *virens* Griseb.

Herb.

Departmental distribution in Paraguay: Alto Paraguay, Ñeembucú, Presidente Hayes.

Voucher: *B. Balansa 4588* (BM, G).

#### CELASTRACEAE

##### *Maytenus officinalis Mabb., Feddes Repert. 101: 274. 1990.

Syn.: *Celastrus spinifolium* Larrañaga; *Maytenus aquifolium* Mart.; *Maytenus hassleri* Briq.; *Maytenus ilicifolia* Mart. ex Reissek; *Maytenus ilicifolia* Mart. ex Reissek forma *angustior* Briq.; *Maytenus pilcomayensis* Briq.

Shrub.

Departmental distribution in Paraguay: Alto Paraguay, Canindeyú, Central, Cordillera, Guairá, Misiones, Ñeembucú, Paraguarí, Presidente Hayes.

Voucher: *L. Bernardi 18376* (G) (http://www.ville-ge.ch/musinfo/bd/cjb/fdp/, as *Maytenus ilicifolia*).

#### COMBRETACEAE

##### *Terminalia triflora (Griseb.) Lillo, Contr. Conoc. Arb. Argent. 20. 1910.

Syn.: *Chuncoa triflora* Griseb.; *Myrobalanus balansae* Kuntze; *Myrobalanus triflora* (Griseb.) Kuntze; *Terminalia balansae* (Kuntze) K.Schum.; *Terminalia balansae* (Kuntze) Hassl., comb. superfl.; *Terminalia hassleriana* Chodat; *Terminalia hassleriana* Chodatvar. *bernardiensis* Chodat

Tree or shrub.

Departmental distribution in Paraguay: Alto Paraná, Alto Paraguay, Amambay, Caaguazú, Caazapá, Canindeyú, Central, Concepción, Cordillera, Guairá, Itapúa, Misiones, Ñeembucú, Paraguarí, Presidente Hayes, San Pedro.

Voucher: *J. De Egea & R. Elsam 821* (BM, CTES, FCQ, G, MO).

#### COMMELINACEAE

##### ►*Commelina diffusa* Burm.f., Fl. Ind. (N.L. Burman) 18. 1768.

Syn.: *Commelina cayennensis* Rich.; *Commelina cayennensis* Rich.var. *pubescens* Griseb.; *Commelina longicaulis* Jacq.; *Commelina nudiflora* auct. non L.; *Commelina sellowiana* Kunth

Herb.

Departmental distribution in Paraguay: Concepción, Guairá, Ñeembucú, Paraguarí.

Voucher: *J. De Egea et al. 693* (BM, FCQ).

##### *Commelina erecta L., Sp. Pl. 1: 41. 1753.

Syn.: *Commelina elegans* Kunth; *Commelina pohliana* Seub.; *Commelina sulcata* Willd.; *Commelina virginica* auct. non L.; *Commelina virginica* L. var. *australis* C.B.Clarke

Herb.

Departmental distribution in Paraguay: Amambay, Canindeyú, Central, Ñeembucú, Paraguarí, Presidente Hayes.

Voucher: *R. Spichiger et al. 5212* (G) (http://www.ville-ge.ch/musinfo/bd/cjb/fdp/).

##### *Commelina platyphylla Klotzch, Reis. Br.-Guiana [Ri. Schomburgk] 3: 897. 1849.

Syn.: *Commelina balansae* (C.B.Clarke) Herter; *Commelina platyphylla* Klotzsch ex Seub. var. *balansae* C.B.Clarke

Herb.

Departmental distribution in Paraguay: Caaguazú, Central, Cordillera, Ñeembucú, Paraguarí, Presidente Hayes.

Voucher: *J. De Egea et al. 631* (BM, CTES).

##### ►*Floscopa glabrata* (Kunth) Hassk., Commelin. Ind. 166. 1870.

Syn.: *Dithyrocarpus glabratus* Kunth

Herb.

Departmental distribution in Paraguay: Alto Paraná, Caaguazú, Caazapá, Canindeyú, Cordillera, Guairá, Misiones, Ñeembucú, Paraguarí.

Voucher: *S. Keel & L. Spinzi 1447* (FCQ).

##### *Tradescantia anagallidea Seub., Fl. Bras. (Martius) 3(1): 249. 1871.

Herb.

Departmental distribution in Paraguay: Alto Paraná, Canindeyú, Guairá, Ñeembucú.

Voucher: *M. Vera et al. 230* (FCQ).

##### *Tripogandra glandulosa (Seub.) Rohweder, Abh. Auslandsk., Reihe C, Naturwiss. 18: 156. 1956.

Syn.: *Descantaria glandulosa* (Seub.) G.Brückn.; *Descantaria pflanzii* G.Brückn.; *Descantaria radiata* (C.B.Clarke) G.Brückn.; *Tradescantia glandulosa* Seub.; *Tradescantia pflanzii* G.Brückn.; *Tradescantia radiata* C.B.Clarke; *Tripogandra pflanzii* (G.Brückn.) Burkart, comb. superfl.; *Tripogandra pflanzii* (G.Brückn.) Celarier, comb. superfl.; *Tripogandra pflanzii* (G.Brückn.) Rohweder; *Tripogandra radiata* (C.B.Clarke) Bacigalupo

Herb.

Departmental distribution in Paraguay: Alto Paraguay, Amambay, Central, Concepción, Cordillera, Guairá, Ñeembucú, Presidente Hayes, San Pedro.

Voucher: *M. Peña-Chocarro et al. 1992* (BM, CTES, FCQ, G, MO).

#### CONVOLVULACEAE

##### ►*Aniseia argentina* (N.E.Br.) O´Donell, Lilloa 23: 473. 1950.

Syn.: *Aniseia cernua* Moric. forma *parviflora* Chodat & Hassl.; *Aniseia cernua* Moric. var. *ambigua* Meisn.; *Ipomoea argentina* N.E.Br.; *Ipomoea argentinensis* Speg.; *Ipomoea cernua* (Moric.) Arechav. forma *chacoensis* Hassl.; *Ipomoea cernua* (Moric.) Arechav. forma *obtusiflora* Hassl.; *Ipomoea cernua* (Moric.) Arechav. forma *palmirense* Arechav.; *Ipomoea cernua* (Moric.) Arechav. forma *platensis* Hassl.; *Ipomoea cernua* (Moric.) Arechav. forma *yapeyuana* Arechav.; *Ipomoea cernua* (Moric.) Arechav. var. *ambigua* (Meisn.) Arechav.

Climber.

Departmental distribution in Paraguay: Concepción, Ñeembucú, Presidente Hayes.

Voucher: *S. Keel & L. Spinzi 1461* (FCQ).

##### *Convolvulus crenatifolius Ruiz & Pav., Fl. Peruv. [Ruiz & Pavon] 2: 10. 1799.

Syn.: *Convolvulus crenatifolius* Ruiz & Pav. var. *argentinica* Hallier f.; *Convolvulus crenatifolius* Ruiz & Pav. var. *montevidensis* (Spreng.) Hallier f.; *Convolvulus montevidensis* Spreng.; *Convolvulus montevidensis* Spreng. var. *megapotamicum* Meisn.; *Ipomoea montevidensis* (Spreng.) G.Don

Climber.

Departmental distribution in Paraguay: Alto Paraguay, Central, Cordillera, Guairá, Ñeembucú.

Voucher: *J. De Egea et al. 717* (FCQ).

##### ►*Dichondra microcalyx* (Hallier f.) Fabris, Fl. Prov. Buenos Aires 5a: 74. 1965.

Syn.: *Dichondra repens* auct. non J.R.Forst. & G.Forst.; *Dichondra repens* J.R.Forst. & G.Forst var. *microcalyx* Hallier f.; *Dichondra sericea* Sw. var. *microcalyx* (Hallier f.) H.T.Buck

Herb.

Departmental distribution in Paraguay: Cordillera, Guairá, Ñeembucú.

Voucher: *M. Peña-Chocarro et al. 2257* (BM, CTES, FCQ, G, MO, PY, SI).

##### *Evolvulus glomeratus Nees & Mart. subsp. *grandiflorus* (D.Parodi) Ooststr., Meded. Bot. Mus. Herb. Rijks Univ. Utrecht 14: 232. 1934.

Syn.: *Evolvulus grandiflorus* D.Parodi; *Evolvulus martii* Meisn. forma *saltense* Arechav.; *Evolvulus paraguariensis* Chodat & Hassl.

Herb.

Departmental distribution in Paraguay: Guairá, Ñeembucú.

Voucher: *A.G. Schulz 7974* (G) (http://www.ville-ge.ch/musinfo/bd/cjb/fdp/).

##### ►*Evolvulus sericeus* Sw., Prodr. (Swartz) 55. 1788.

Syn.: *Evolvulus anomalus* Meisn.; *Evolvulus araucanus* Phil.; *Evolvulus holosericeus* Kunth; *Evolvulus sericeus* Sw. var. *holosericeus* (Kunth) Ooststr.; *Evolvulus sericeus* Sw. var. *latior* Meisn.

Herb.

Departmental distribution in Paraguay: Alto Paraguay, Central, Concepción, Cordillera, Guairá, Ñeembucú, Paraguarí.

Voucher: *J. De Egea & R. Elsam 800* (BM, FCQ).

##### *Ipomoea carnea Jacq. subsp. *fistulosa* (Mart. ex Choisy) D.F.Austin, Taxon 26: 237. 1977.

Syn.: *Batatas crassicaulis* Benth.; *Ipomoea crassicaulis* (Benth.) Robinson; *Ipomoea fistulosa* Mart. ex Choisy; *Ipomoea gossypioides* D.Parodi; *Ipomoea texana* Coult.

Shrub.

Departmental distribution in Paraguay: Alto Paraguay, Caazapá, Central, Cordillera, Ñeembucú, Presidente Hayes.

Voucher: *J. De Egea et al. 367* (BM, CTES, FCQ, G, MO, PY, SI, UNR).

##### ►*Ipomoea grandifolia* (Dammer) O’Donell, Arq. Mus. Paranaense 9: 222. 1952.

Syn.: *Ipomoea triloba* auct. non L.; *Jacquemontia grandifolia* Dammer

Climber.

Departmental distribution in Paraguay: Alto Paraguay, Canindeyú, Central, Cordillera, Guairá, Ñeembucú, Paraguarí.

Voucher: *Rosaliz Garcia et al. 1* (FCQ, UNP).

##### *Ipomoea indica (Burm.f.) Merr., Interpr. Herb. Amboin. 445. 1917.

Syn.: *Convolvulus acuminatus* Vahl; *Convolvulus bogotensis* Kunth; *Convolvulus congestus* (R.Br.) Spreng.; *Convolvulus indicus* Burm.f.; *Convolvulus mollis* Kunth; *Convolvulus portoricensis* Spreng.; *Ipomoea acuminata* (Vahl) Roem. & Schult.; *Ipomoea bogotensis* (Kunth) G.Don; *Ipomoea cathartica* Poir.; *Ipomoea congesta* R.Br.; *Ipomoea mollis* (Kunth) G.Don; *Ipomoea portoricensis* (Spreng.) G.Don; *Ipomoea vahliana* House; *Pharbitis acuminata* (Vahl) Choisy; *Pharbitis acuminata* var. *congesta* Choisy; *Pharbitis bogotensis* (Kunth) Choisy; *Pharbitis cathartica* (Poir.) Choisy; *Pharbitis mollis* (Kunth) Choisy; *Pharbitis rosea* Choisy

Climber.

Departmental distribution in Paraguay: Guairá, Ñeembucú, Paraguarí.

Voucher: *R. Spichiger et al. 5261* (CTES, G, MO) (http://www.ville-ge.ch/musinfo/bd/cjb/fdp/).

##### *Iseia luxurians* (Moric.) O’Donell, Bot. Soc. Argent. Bot. 5(1-2): 77. 1953.

Syn.: *Ipomoea grisebachiana* Meisn.; *Ipomoea jamesonii* Choisy; *Ipomoea luxurians* Moric.; *Ipomoea sericantha* Griseb. nom. illeg.; *Ipomoea sericea* Spreng. ex Choisy; *Jacquemontia luxurians* (Moric.) Hallier f.

Climber.

Departmental distribution in Paraguay: Ñeembucú.

Voucher: *T. Meyer 15965* (LIL) (cited in O’Donell 1953: 80).

#### CUCURBITACEAE

##### ►*Cayaponia citrullifolia* (Griseb.) Cogn. ex Griseb., Abh. Ges. Wiss. Goett. 24: 135. 1879.

Syn.: *Antagonia citrullifolia* Griseb.; *Cayaponia breviloba* (Griseb. ex Cogn.) Lillo, nom. nud.; *Cayaponia citrullifolia* (Griseb.) Cogn. ex Griseb. var. *breviloba* Griseb. ex Cogn.; *Cayaponia latifolia* Cogn.

Climber.

Departmental distribution in Paraguay: Alto Paraná, Alto Paraguay, Canindeyú, Central, Concepción, Cordillera, Guairá, Ñeembucú, Paraguarí.

Voucher: *M. Peña-Chocarro et al. 2325* (BM, CTES, FCQ).

##### *Melothria pendula L., Sp. Pl. 1: 35. 1753.

Syn.: *Melothria fluminensis* Gardner

Herb.

Departmental distribution in Paraguay: Alto Paraná, Amambay, Canindeyú, Central, Cordillera, Itapúa, Ñeembucú, Paraguarí, Presidente Hayes.

Voucher: *R. Spichiger et al. 5313* (G) (http://www.ville-ge.ch/musinfo/bd/cjb/fdp/).

##### ►*Melothria warmingii* Cogn., Fl. Bras. (Martius) 6(4): 27. 1878.

Climber.

Departmental distribution in Paraguay: Alto Paraná, Amambay, Caazapá, Central, Cordillera, Guairá, Itapúa, Misiones, Ñeembucú, Paraguarí.

Voucher: *M. Peña-Chocarro et al. 2327* (BM, FCQ).

##### ►*Momordica charantia* L., Sp. Pl. 2: 1009. 1753.

Syn.: *Momordica charantia* L. var. *abbreviata* Ser.

Climber.

Departmental distribution in Paraguay: Alto Paraguay, Ñeembucú.

Voucher: *M. Morinigo et al. 2* (BM, CTES, FCQ, G, MO, PY, SI).

#### CYPERACEAE

##### ►*Carex trachycystis* Griseb., Abh. Königl. Ges. Wiss. Göttingen 24: 314. 1879.

Syn.: *Carex bonariensis* Desf. ex Poir. forma *remota* Kük.; *Carex bonariensis* Desf. ex Poir. var. *remota* Kük.; *Carex bonariensis* Desf. ex Poir. var. *trachycystis* (Griseb.) Kük.; *Carex sororia* auct. non Kunth

Herb.

Departmental distribution in Paraguay: Ñeembucú, Presidente Hayes.

Voucher: *M. Peña-Chocarro et al. 2215* (BM, CTES, FCQ, G, MO, PY, SI).

##### ►*Cyperus aggregatus* (Willd.) Endl., Cat. Horti Vindob. 1: 93. 1842.

Syn.: *Cyperus argentinus* Boeckeler; *Cyperus cayennensis* Willd. ex Link; *Cyperus flavomariscus* Griseb.; *Cyperus flavus* (Vahl) Nees; *Cyperus flavus* (Vahl) Neesvar. *aggregatus* (Willd.) Kük.; *Cyperus flavus* (Vahl) Neesvar. *angustatus* Kük.; *Cyperus flavus* (Vahl) Neesvar. *argentinus* (Boeckeler) Kük. ex Osten; *Cyperus flavus* (Vahl) Neesvar. *laevis* (Kunth) Kük.; *Didymia cyperomorpha* Phil.; *Kyllinga cayennensis* Lam.; *Mariscus aggregatus* Willd.; *Mariscus cayennensis* (Lam.) Urb.; *Mariscus flavus* Vahl

Herb.

Departmental distribution in Paraguay: Alto Paraná, Alto Paraguay, Canindeyú, Central, Cordillera, Guairá, Itapúa, Misiones, Ñeembucú, Paraguarí, Presidente Hayes, San Pedro.

Voucher: *M. Peña-Chocarro et al. 2261* (FCQ).

##### *Cyperus digitatus Roxb., Fl. Ind., ed. 1820, 1: 205. 1820.

Syn.: *Cyperus digitatus* Roxb. forma *contractior* Kük.; *Cyperus digitatus* Roxb. var. *obtusifructus* Kük.; *Cyperus hieronymi* auct. non Boeckeler

Herb. In swamps.

Departmental distribution in Paraguay: Alto Paraguay, Central, Cordillera, Ñeembucú, Presidente Hayes.

Voucher: *T. Rojas 12615* (SI) (cited in Zuloaga et al. 2008: 331).

##### ►*Cyperus entrerianus* Boeckeler, Flora 61: 139. 1878.

Syn.: *Cyperus conoideus* Rich.; *Cyperus luzulae* (L.) Rottb. ex Retz. forma *conoideus* (Rich.) Nees; *Cyperus luzulae* (L.) Rottb. ex Retz. var. *entrerianus* (Boeckeler) Barros; *Cyperus luzulae* (L.) Rottb. ex Retz. var. *tucumanensis* (Boeckeler) C.B.Clarke ex Chodat; *Cyperus surinamensis* Rottb. var. *strictus* Kük.; *Cyperus tucumanensis* Boeckeler

Herb. In swamps.

Departmental distribution in Paraguay: Alto Paraguay, Caaguazú, Caazapá, Central, Concepción, Cordillera, Guairá, Misiones, Ñeembucú, Presidente Hayes, San Pedro.

Voucher: *J. De Egea et al. 651* (BM, FCQ, MO).

##### *Cyperus giganteus Vahl, Enum. Pl. [Vahl] 2: 364. 1805.

Syn.: *Chlorocyperus giganteus* (Vahl) Palla; *Cyperus comosus* Poir.

Herb. In swamps.

Departmental distribution in Paraguay: Alto Paraguay, Central, Concepción, Itapúa, Ñeembucú, Presidente Hayes.

Voucher: *J. De Egea et al. 804* (BM, FCQ).

##### *Cyperus haspan L., Sp. Pl. 1: 45. 1753.

Syn.: *Cyperus adenophorus* Schrad.; *Cyperus americanus* (Boeckeler) Palla; *Cyperus autummalis* (Rottb.) Vahl; *Cyperus cayennensis* Willd. ex Link; *Cyperus efoliatus* Boeckeler; *Cyperus haspan* L. subsp. *juncoides* (Lam.) Kük.; *Cyperus haspan* L. var. *adenophorus* (Schrad.) Kük.; *Cyperus haspan* L. var. *americanus* Boeckeler; *Cyperus haspan* L. var. *riparius* (Schrad. ex Nees) Kük.; *Cyperus juncoides* Lam.; *Cyperus riparius* Schrad. ex Nees; *Scirpus autumnalis* Rottb., nom. illeg.

Herb.

Departmental distribution in Paraguay: Caaguazú, Caazapá, Canindeyú, Central, Cordillera, Guairá, Itapúa, Ñeembucú.

Voucher: *J. De Egea et al. 695* (BM, FCQ).

##### *Cyperus impolitus Kunth, Enum. Pl. [Kunth] 2: 78. 1837.

Syn.: *Chlorocyperus balansae* (Maury) Palla; *Cyperus serrae* Palla; *Cyperus balansae* Maury; *Cyperus laetus* J.Presl & C.Presl var. *balansae* (Maury) Kük.; *Cyperus laetus* J.Presl & C.Presl var. *capitatus* Boeckeler; *Cyperus laetus* J.Presl & C.Presl var. *hassleri* (C.B.Clarke) Kük.; *Cyperus laetus* J.Presl & C.Presl var. *impolitus* (Kunth) Kük., pro parte; *Cyperus laetus* J.Presl & C.Presl *wacketii* Kük.; *Mariscus balansae* (Maury) C.B.Clarke; *Mariscus hassleri* C.B.Clarke; *Mariscus impolitus* (Kunth) J.Raynal

Aquatic herb.

Departmental distribution in Paraguay: Alto Paraná, Amambay, Caaguazú, Caazapá, Central, Concepción, Cordillera, Guairá, Misiones, Ñeembucú, Paraguarí, Presidente Hayes, San Pedro.

Voucher: *Spichiger et al. 5288* (G) (http://www.ville-ge.ch/musinfo/bd/cjb/fdp/).

##### *Cyperus laxus Lam., Tab. Encycl. 1: 146. 1791.

Syn.: *Cyperus diffusus* Vahl; *Cyperus diffusus* Vahlvar. *umbrosus* (Lindl. & Nees) Kük.; *Cyperus elegans* auct. non L.; *Cyperus limbatus* Maury; *Cyperus umbrosus* Lindl. & Nees ex Nees

Herb. In swamps.

Departmental distribution in Paraguay: Alto Paraná, Alto Paraguay, Amambay, Central, Concepción, Cordillera, Guairá, Misiones, Ñeembucú, Paraguarí.

Voucher: *A.G. Schulz 7728* (SI) (http://www.tropicos.org/).

#### ARACEAE

##### *Cyperus odoratus* L., Sp. Pl. 1: 46. 1753.

Syn.: *Cyperus acicularis* (Nees) Steud.; *Cyperus cephalophorus* J.Presl & C.Presl; *Cyperus conglobatus* Link; *Cyperus densiflorus* G.Mey.; *Cyperus engelmannii* Steud.; *Cyperus ferax* Rich.; *Cyperus ferax* Rich. subsp. *engelmanni* (Steud.) Kük.; *Cyperus ferax* Rich. subsp. *speciosus* (Vahl) Kük.; *Cyperus ferax* Rich. var. *acicularis* (Nees) Kük; *Cyperus ferax* Rich. var. *bulbiferus* Barros; *Cyperus ferax* Rich. var. *conglobatus* (Link) Kük.; *Cyperus ferax* Rich. var. *maximilianii* (Schrad. ex Nees) Boeckeler; *Cyperus ferox* Vahl; *Cyperus flexuosus* Vahl; *Cyperus hamiltonii* Kunth; *Cyperus huarmensis* (Kunth) M.C.Johnst.; *Cyperus jubaeiflorus* Rudge; *Cyperus maximilianii* (Schrad. ex Nees) Griseb.; *Cyperus pohlianus* (Nees) Kuntze; *Cyperus speciosus* Vahl; *Diclidium lenticulare* Schrad. ex Nees; *Diclidium lomentaceum* Nees; *Diclidium maximilianii* Schrad. ex Nees; *Diclidium odoratum* Schrad. ex Nees; *Diclidium uliginosum* Schrad. ex Nees; *Mariscus ferax* (Rich.) C.B.Clarke; *Mariscus pohlianus* Nees; *Torulinium confertum* Desv.; *Torulinium ferax* (Rich.) Urb.; *Torulinium odoratum* (L.) Hooper

Herb. In swamps.

Departmental distribution in Paraguay: Alto Paraná, Alto Paraguay, Amambay, Caaguazú, Caazapá, Central, Cordillera, Guairá, Itapúa, Ñeembucú, Paraguarí, Presidente Hayes.

Voucher: *M. Peña-Chocarro et al. 2339* (BM, CTES, FCQ).

##### *Cyperus prolixus Kunth, Nov. Gen. Sp. (quarto ed.) 1: 206. 1815.

Syn.: *Comostemon schottii* Nees; *Cyperus amplissimus* Steud.; *Cyperus bisumellatus* Steud.

Herb.

Departmental distribution in Paraguay: Amambay, Caazapá, Canindeyú, Central, Cordillera, Guairá, Misiones, Ñeembucú.

Voucher: *A.G. Schulz 7772* (G) (http://www.ville-ge.ch/musinfo/bd/cjb/fdp/).

##### *Cyperus reflexus Vahl, Enum. Pl. [Vahl] 2: 299. 1805.

Syn.: *Cyperus haematostachys* Steud.; *Cyperus reflexus* Vahl forma *capitata* Osten; *Cyperus reflexus* Vahl var. *macrostachys* Boeckeler; *Cyperus reflexus* Vahl forma *major* Maury; *Cyperus sellowii* Link

Herb.

Departmental distribution in Paraguay: Central, Ñeembucú.

Voucher: *R. Spichiger et al. 5290* (CTES, G, NY) (http://www.ville-ge.ch/musinfo/bd/cjb/fdp/).

##### *Cyperus surinamensis* Rottb., Descr. Pl. Rar. 20. 1772.

Syn.: *Cyperus denticulatus* Schrad. ex Schult.

Herb.

Departmental distribution in Paraguay: Alto Paraguay, Amambay, Canindeyú, Central, Cordillera, Guairá, Ñeembucú, Presidente Hayes.

Voucher: *T. Rojas 12616* (SI) (cited in Zuloaga et al. 2008: 343).

##### *Eleocharis bonariensis Nees, J. Bot. (Hooker) 2: 398. 1840.

Syn.: *Chaetocyperus bonariensis* (Nees) Nees; *Chaetocyperus obtusatus* Nees; *Eleocharis aciculariformis* Greenm.; *Eleocharis acicularis* (L.) Roem. & Schult. subsp. *bonariensis* (Nees) Osten; *Eleocharis striatula* E.Desv.; *Scirpus striatulus* (E.Desv.) Griseb.

Herb. In swamps.

Departmental distribution in Paraguay: Alto Paraná, Caaguazú, Caazapá, Central, Concepción, Cordillera, Guairá, Itapúa, Ñeembucú, Paraguarí.

Voucher: *R. Spichiger et al. 5291* (G) (http://www.ville-ge.ch/musinfo/bd/cjb/fdp/).

##### *Eleocharis contracta* Maury, Mém. Soc. Phys. Genève 31: 139. 1889.

Syn.: *Eleocharis nodulosa* (Roth) Schult. var. *subnodulosa* Svenson, pro parte; *Eleocharis nodulosa* (Roth) Schult. var. *tenuis* Boeckeler; *Eleocharis posadensis* Kük.

Aquatic herb.

Departmental distribution in Paraguay: Alto Paraná, Alto Paraguay, Amambay, Canindeyú, Central, Concepción, Cordillera, Itapúa, Misiones, Ñeembucú, Paraguarí, Presidente Hayes.

Voucher: *M. Peña-Chocarro et al. 2348* (BM, FCQ).

##### *Eleocharis elegans (Kunth) Roem. & Schult., Syst. Veg., ed. 15 bis [Roemer & Schultes] 2: 150. 1817.

Syn.: *Eleocharis geniculata* auct. non (L.) Roem. & Schult.; *Scirpus crassiculmis* Griseb.; *Scirpus elegans* Kunth

Aquatic herb.

Departmental distribution in Paraguay: Alto Paraná, Alto Paraguay, Amambay, Caaguazú, Canindeyú, Central, Concepción, Cordillera, Guairá, Itapúa, Misiones, Ñeembucú, Paraguarí, Presidente Hayes, San Pedro.

Voucher: *J. De Egea et al. 649b* (BM).

##### *Eleocharis flavescens (Poir.) Urb., Symb. Antill. 4(1): 116. 1903.

Syn.: *Eleocharis albivaginata* Boeckeler var. *flaccida* (Rchb. ex Spreng.) Boeckeler; *Eleocharis albivaginata* Boeckeler var. *humilis* Boeckeler; *Eleocharis albivaginata* Boeckeler var. *stricta* Boeckeler; *Eleocharis arechavaletae* Boeckeler; *Eleocharis flaccida* (Rchb. ex A.Spreng.) Urb.; *Eleocharis flaccida* (Rchb. ex A.Spreng.) Urb. var. *arechavaletae* Osten; *Eleocharis ochreata* (Nees) Steud.; *Eleocharis ochreata* (Nees) Steud. var. *flaccida* (Rchb. ex A.Spreng.) Boeckeler; *Eleogenus ochreatus* Nees; *Eleogenus ochreatus* Nees var. *flaccidus* Nees; *Eleogenus ochreatus* Nees var. *minor* Nees; *Scirpus flaccidus* Rchb. ex A.Spreng.; *Scirpus flavescens* Poir.; *Scirpus gaudichaudianus* Kunth; *Scirpus ochreatus* (Nees) Kunth; *Trichophyllum ochreatum* (Nees) House

Aquatic herb.

Departmental distribution in Paraguay: Amambay, Caaguazú, Caazapá, Concepción, Cordillera, Itapúa, Ñeembucú, Paraguarí.

Voucher: *M. Peña-Chocarro et al. 2271* (BM, FCQ).

##### ►*Eleocharis interstincta* (Vahl) Roem. & Schult., Syst. Veg., ed. 15 bis [Roem. 7 Schultes] 2: 149. 1817.

Syn.: *Scirpus interstincta* Vahl

Aquatic herb.

Departmental distribution in Paraguay: Amambay, Concepción, Cordillera, Ñeembucú, San Pedro.

Voucher: *M. Vera et al. 212* (BM, CTES, FCQ, MO).

##### *Eleocharis minima* Kunth, Enum. Pl. [Kunth] 2: 139. 1837.

Syn.: *Chaetocyperus polymorphus* Nees & Lindl., pro parte; *Eleocharis durandii* Boeckeler; *Eleocharis tenuissima* Boeckeler; *Eleocharis wrightiana* Boeckeler

Herb. In swamps.

Departmental distribution in Paraguay: Alto Paraná, Alto Paraguay, Amambay, Caaguazú, Caazapá, Central, Concepción, Cordillera, Itapúa, Misiones, Ñeembucú, Paraguarí, Presidente Hayes, San Pedro.

Voucher: *M.A. Walter 175* (BM).

##### ►*Eleocharis montana* (Kunth) Roem. & Schult., Syst. Veg., ed. 15 bis [Roemer & Schultes] 2: 153. 1817.

Syn.: *Eleocharis consanguinea* Kunth; *Eleocharis montana* (Kunth) Roem. & Schult. var. *nodulosa* (Roth) Svenson; *Eleocharis nodulosa* (Roth) Schult.; *Eleocharis nodulosa* (Roth) Schult. forma *trigyna* Barros; *Eleogenus nodulosus* (Roth) Nees; *Scirpus montanus* Kunth; *Scirpus nodulosus* Roth

Aquatic herb.

Departmental distribution in Paraguay: Alto Paraná, Alto Paraguay, Amambay, Caaguazú, Caazapá, Canindeyú, Central, Concepción, Cordillera, Guairá, Itapúa, Misiones, Ñeembucú, Paraguarí, Presidente Hayes, San Pedro.

Voucher: *M. Peña-Chocarro et al. 2349* (BM, FCQ).

##### *Eleocharis sellowiana* Kunth, Enum. Pl. [Kunth] 2: 149. 1837

Syn.: *Eleocharis albivaginata* Boeckeler var. *macrostachya* Boeckeler; *Eleocharis galapagensis* Svenson; *Eleocharis mesopotamica* Boeckeler; *Eleogenus sellowianus* (Kunth) Nees; *Scirpus sellowianus* (Kunth) Griseb.

Herb.

Departmental distribution in Paraguay: Caaguazú, Caazapá, Central, Cordillera, Guairá, Itapúa, Ñeembucú, Paraguarí, San Pedro.

Voucher: *M.A. Walter 142* (BM).

##### *Fimbristylis autumnalis* (L.) Roem. & Schult., Syst. Veg., ed. 15 bis [Roemer & Schultes] 2: 97. 1817.

Syn.: *Isolepis autumnalis* (L.) J.Presl & C.Presl; *Scirpus autumnalis* L.; *Scirpus mucronulatus* Michx.

Herb. In swamps.

Departmental distribution in Paraguay: Alto Paraná, Amambay, Caaguazú, Caazapá, Central, Concepción, Cordillera, Guairá, Ñeembucú, Paraguarí, San Pedro.

Voucher: *M.A. Walter 179* (BM).

##### *Fimbristylis complanata* (Retz.) Link, Hort. Berol. [Link] 1: 292. 1827.

Syn.: *Fimbristylis autumnalis* (L.) Roem. & Schult. var. *complanata* (Retz.) Kük.; *Fimbristylis autumnalis* (L.) Roem. & Schult. var. *complanata* (Retz.) Barros, comb. superfl.; *Scirpus complanatus* Retz.

Herb.

Departmental distribution in Paraguay: Caaguazú, Caazapá, Central, Cordillera, Itapúa, Ñeembucú, San Pedro.

Voucher: Cited in Zuloaga et al. 2008: 357.

##### *Fimbristylis dichotoma* (L.) Vahl, Enum. Pl. [Vahl] 2: 287. 1805.

Syn.: *Fimbristylis annua* (All.) Roem. & Schult.; *Fimbristylis annua* (All.) Roem. & Schult. forma *tomentosa* (Vahl) Kük.; *Fimbristylis annua* (All.) Roem. & Schult. var. *diphylla* (Retz.) Kük.; *Fimbristylis brachyphylla* Schult.; *Fimbristylis communis* Kunth; *Fimbristylis diphylla* (Retz.) Vahl; *Fimbristylis diphylla* (Retz.) Vahlvar. *tomentosa* (Vahl) Barros; *Fimbristylis dregeana* Kunth; *Fimbristylis ecklonii* Nees; *Fimbristylis laxa* Vahl; *Fimbristylis polymorpha* Boeckeler; *Fimbristylis subtristachya* Steud.; *Fimbristylis tomentosa* Vahl; *Scirpus annuus* All.; *Scirpus dichotomus* L.; *Scirpus diphyllus* Retz.

Aquatic herb.

Departmental distribution in Paraguay: Alto Paraguay, Amambay, Caaguazú, Caazapá, Central, Cordillera, Guairá, Itapúa, Misiones, Ñeembucú, Paraguarí, Presidente Hayes, San Pedro.

Voucher: *M. Peña-Chocarro et al. 2343* (BM, CTES, FCQ, MO).

##### *Fuirena umbellata Rottb., Descr. Icon. Rar. Pl. 70. 1773.

Syn.: *Scirpus umbellatus* (Rottb.) Kuntze

Herb.

Departmental distribution in Paraguay: Alto Paraguay, Canindeyú, Ñeembucú, San Pedro.

Voucher: *R. Spichiger et al. 5298* (G) (http://www.ville-ge.ch/musinfo/bd/cjb/fdp/).

##### ►*Kyllinga brevifolia* Rottb., Descr. Icon. Rar. Pl. 13. 1773.

Syn.: *Cyperus brevifolius* (Rottb.) Hassk.; *Cyperus sesquiflorus* (Torr.) Mattf. & Kük. ex Kük. forma *gracilis* (Boeckeler) Kük.; *Kyllinga brevifolia* Rottb. var. *longifolia* Boeckeler; *Kyllinga cruciformis* Schrad. ex Roem. & Schult.; *Kyllinga sororia* Kunth; *Kyllinga tenuis* Baldwin

Herb. In swamps.

Departmental distribution in Paraguay: Alto Paraná, Amambay, Caazapá, Central, Guairá, Ñeembucú.

Voucher: *M. Peña-Chocarro et al. 2218* (BM, CTES, FCQ, MO).

##### *Kyllinga odorata Vahl, Enum. Pl. [Vahl] 2: 382. 1805.

Syn.: *Cyperus sesquiflorus* (Torr.) Mattf. & Kük. ex Kük.; *Cyperus sesquiflorus* (Torr.) Mattf. & Kük. ex Kük. forma *bulbiferus* (Kük.) Kük.; *Kyllinga leucocephala* Baldwin; *Kyllinga martiana* Schrad. ex Nees; *Kyllinga monocephala* Kunth, nom. illeg.; *Kyllinga odorata* Vahl var. *bulbifera* Kük.; *Kyllinga odorata* Vahl var. *genuina* Osten, nom. illeg.; *Kyllinga odorata* Vahl var. *gracilis* Boeckeler; *Kyllinga odorata* Vahl var. *rigida* Boeckeler; *Kyllinga pellucido-albida* Boeckeler; *Kyllinga sesquiflora* Torr.; *Kyllinga triceps* auct. non Rottb.

Herb. In swamps.

Departmental distribution in Paraguay: Alto Paraná, Alto Paraguay, Amambay, Caaguazú, Central, Concepción, Cordillera, Guairá, Ñeembucú, Paraguarí, San Pedro.

Voucher: *M. Peña-Chocarro et al. 2260* (BM, CTES, FCQ, G, MO, SI).

##### *Kyllinga vaginata Lam., Tab. Encycl. 1: 148. 1791.

Syn.: *Cyperus obtusatus* (J.Presl & C.Presl) Mattf. & Kük.; *Kyllinga obtusata* J.Presl & C.Presl; *Kyllinga peruviana* Lam.; *Kyllinga pungens* Link; *Kyllinga rigida* Baldwin; *Kyllinga stricta* Schrad. ex Nees; *Kyllinga tenuis* Boeckeler, nom. illeg.

Herb. In swamps.

Departmental distribution in Paraguay: Alto Paraguay, Cordillera, Ñeembucú, Paraguarí.

Voucher: *J. De Egea et al. 701* (BM, FCQ).

##### ►*Pycreus flavicomus* (Michx.) C.D.Adams, Ann. Missouri Bot. Gard. 78(1): 254. 1991.

Syn.: *Cyperus albomarginatus* (Mart. & Schrad. ex Nees) Steud.; *Cyperus albomarginatus* (Mart. & Schrad. ex Nees) Steud. var. *pachyanthemos* Kük.; *Cyperus albomarginatus* (Mart. & Schrad. ex Nees) Steud. var. *sabulosus* (Mart. & Schrad. ex Nees) Kük.; *Cyperus flavicomus* Michx.; *Cyperus sabulosus* (Mart. & Schrad. ex Nees) Steud.; *Pycreus albomarginatus* Mart. & Schrad. ex Nees; *Pycreus sabulosus* Mart. & Schrad. ex Nees

Herb.

Departmental distribution in Paraguay: Ñeembucú, San Pedro.

Voucher: *S. Keel & L. Spinzi 1445* (FCQ).

##### *Pycreus lanceolatus (Poir.) C.B.Clarke, Consp. Fl. Afr. 5: 538. 1894.

Syn.: *Cyperus helvus* Liebm., nom. illeg.; *Cyperus lepidus* Phil.; *Cyperus lanceolatus* Poir.; *Cyperus olfersianus* Kunth; *Cyperus olfersianus* Kunthvar. *maximilianii* Boeckeler; *Pycreus helvus* (Liebm.) C.B.Clarke; *Pycreus olfersianus* (Kunth) Nees; *Pycreus propinquus* Nees

Aquatic herb.

Departmental distribution in Paraguay: Canindeyú, Central, Cordillera, Guairá, Itapúa, Ñeembucú, Paraguarí.

Voucher: *J. De Egea et al. 652* (FCQ).

##### *Rhynchospora asperula* (Nees) Steud., Syn. Pl. Glumac. 2(8-9): 144. 1855.

Syn.: *Calyptrostylis asperula* Nees; *Rhynchospora corymbosa* (L.) Britton var. *asperula* (Nees) Kük.

Herb.

Departmental distribution in Paraguay: Alto Paraná, Amambay, Caaguazú, Caazapá, Canindeyú, Central, Cordillera, Guairá, Itapúa, Misiones, Ñeembucú, Paraguarí, San Pedro.

Voucher: *S. Keel & L. Spinzi 1472* (FCQ).

##### ►*Rhynchospora corymbosa* (L.) Britton, Trans. New York Acad. Sci. 11: 84. 1892.

Syn.: *Calyptrostylis fascicularis* Nees; *Calyptrostylis florida* (Rudge) Nees; *Dichromena corymbosa* (L.) J.F.Macbr.; *Rhynchospora aurea* Vahl; *Rhynchospora corymbosa* (L.) Britton var. *florida* (Rudge) Kük.; *Rhynchospora florida* (Rudge) Schult.; *Rhynchospora surinamensis* (Rottb.) Nees; *Schoenus floridus* Rudge; *Schoenus surinamensis* Rottb.; *Scirpus corymbosus* L.

Herb.

Departmental distribution in Paraguay: Alto Paraguay, Amambay, Caaguazú, Canindeyú, Central, Concepción, Cordillera, Guairá, Itapúa, Ñeembucú, Paraguarí, Presidente Hayes, San Pedro.

Voucher: *M. Peña-Chocarro et al. 2226* (BM, CTES, FCQ, G, MO, SI).

##### ►*Rhynchospora praecincta* Maury ex Micheli, Neue Denkschr. Allg. Schweiz. Ges. Gesammten Naturwiss. 31(1): 146. 1890.

Syn.: *Rhynchospora hyalinolepis* Boeckeler

Herb.

Departmental distribution in Paraguay: Ñeembucú, Paraguarí.

Voucher: *S. Keel & L. Spinzi 1455* (FCQ).

##### *Rhynchospora rugosa (Vahl) Gale, Rhodora 46: 275. 1944.

Syn.: *Dichromena glauca* (Vahl) J.F.Macbr.; *Rhynchospora glauca* Vahl var. *altior* Kük.; *Schoenus rugosus* Vahl

Herb.

Departmental distribution in Paraguay: Amambay, Canindeyú, Ñeembucú.

Voucher: *R. Spichiger et al. 5235* (CTES, G) (http://www.ville-ge.ch/musinfo/bd/cjb/fdp/).

##### ►*Rhynchospora velutina* (Kunth) Boeckeler, Vidensk. Meddel. Dansk Naturhist. Foren. Kjøbenhavn 1869: 149. 1869.

Syn.: *Dichromena sellowiana* Kunth; *Dichromena velutina* Kunth; *Pachymitra rufa* Nees ex Boeckeler, nom. nud.; *Pachymitra velutina* Kunth ex Nees, nom. nud.; *Psilocarya divergens* Nees; *Psilocarya mexicana* Liebm.; *Psilocarya pauloensis* Boeckeler; Psilocarya pohlii Nees; *Psilocarya rufa* Nees; *Psilocarya sellowiana* (Kunth) Nees; *Psilocarya velutina* (Kunth) Nees; *Rhynchospora auraeformis* C.B.Clarke; *Rhynchospora kuekenthalii* H.Pfieff.; *Rhynchospora kuekenthalii* Herter, nom. illeg.; *Rhynchospora mexicana* C.Wright ex Sauvalle; *Rhynchospora robusta* (Kunth) Boeckeler; *Rhynchospora rufa* (Nees) Boeckeler; *Rhynchospora rufa* (Nees) Boeckeler var. *liebmanniana* C.B.Clarke; *Rhynchospora velutina* (Nees) Boeckeler var. *divergens* (Nees) Boeckeler ex Kük.; *Rhynchospora velutina* (Nees) Boeckeler var. *glabrescens* Boeckeler; *Rhynchospora velutina* (Nees) Boeckeler var. *pohlii* (Nees) Boeckeler ex Kük.; *Rhynchospora velutina* (Nees) Boeckeler var. *sellowiana* (Kunth) Boeckeler; *Schoenus mollis* Schrad.ex Nees, nom. nud.; *Schoenus velutinus* Schrad. ex Kunth., nom. nud.

Herb.

Departmental distribution in Paraguay: Canindeyú, Cordillera, Itapúa, Ñeembucú.

Voucher: *S. Keel & L. Spinzi 1453* (FCQ).

##### *Schoenoplectus californicus (C.A.Mey.) Soják, Čas. Nár. Mus., Odd. Přír. 140(3-4): 127. 1972.

Syn.: *Elytrospermum californicus* C.A.Mey.; *Malacochaete assimilis* Ces.; *Malacochaete chilense* Nees & Meyen ex Boeckeler; *Malacochaete oligostachya* Phil. ex Boeckeler; *Malacochaete riparia* Nees & Meyen; *Malacochaete sanguinolenta* Nees & Meyen ex Boeckeler; *Malacochaete tatora* Nees & Meyen, nom. nud.; *Schoenoplectus riparius* (J.Presl & C.Presl) Palla; *Scirpus californicus* (C.A.Mey.) Britton, comb. superfl.; *Scirpus californicus* (C.A.Mey.) Steud.; *Scirpus californicus* (C.A.Mey.) Steud. var. *tatora* (Kunth) Barros; *Scirpus californicus* (C.A.Mey.) Steud. subsp. *tatora* (Kunth) T.Koyama; *Scirpus chamissonis* Schrad. ex Boeckeler; *Scirpus decipiens* A.St.-Hil. ex Nees; *Scirpus rigidus* Schrad. ex Nees; *Scirpus riparius* J.Presl & C.Presl

Herb.

Departmental distribution in Paraguay: Alto Paraguay, Central, Cordillera, Ñeembucú, Presidente Hayes.

Voucher: Cited in Vogt and Mereles 2005: 13.

##### ►*Scleria melaleuca* Rchb. ex Schltdl. & Cham., Linnaea 6(1): 29. 1881.

Syn.: *Scleria pratensis* Lindl. ex Nees; *Scleria pterota* C.Presl, nom. nud.

Herb.

Departmental distribution in Paraguay: Alto Paraná, Amambay, Caaguazú, Canindeyú, Central, Cordillera, Guairá, Misiones, Ñeembucú, Paraguarí, San Pedro.

Voucher: *M. Peña-Chocarro et al. 2370* (BM, CTES, FCQ, G, MO, SI).

#### DIOSCOREACEAE

##### *Dioscorea subhastata Vell., Fl. Flumin. Icon. 10: t. 121. 1831.

Syn.: *Dioscorea friesii* R.Knuth; *Dioscorea guaranitica* Chodat & Hassl.; *Dioscorea guaranitica* Chodat & Hassl. forma *subcoriacea* Chodat & Hassl.; *Dioscorea guaranitica* Chodat & Hassl. var. *balansae* Pellegr.; *Dioscorea lagoa-santa* Uline ex R.Knuth; *Dioscorea lagoa-santa* Uline ex R.Knuthvar. *rotiflora* Uline; *Dioscorea piratinyensis* R.Knuth

Climber.

Departmental distribution in Paraguay: Alto Paraguay, Central, Cordillera, Guairá, Ñeembucú, Paraguarí, Presidente Hayes.

Voucher: *J. De Egea & M. Vera 478* (BM, CTES, FCQ, G, MO, PY, UNR).

#### EBENACEAE

##### *Diospyros inconstans* Jacq., Enum. Syst. Pl. 34. 1790.

Syn.: *Maba inconstans* (Jacq.) Griseb.; *Macreightia inconstans* (Jacq.) A.DC.

Tree.

Departmental distribution in Paraguay: Alto Paraná, Central, Guairá, Misiones, Ñeembucú, Paraguarí, San Pedro.

Voucher: *L. Bernardi 20478* (BM) (Cited in Zuloaga et al. 2008: 1987).

#### ERIOCAULACEAE

##### *Syngonanthus caulescens (Poir.) Ruhland, Bot. Jahrb. Syst. 30(2): 147. 1901.

Syn.: *Dupatya caulescens* (Poir.) Kuntze; *Eriocaulon caulescens* Poir.; *Eriocaulon splendens* Bong.; *Paepalanthus caulescens* (Poir.) Kunth

Herb.

Departmental distribution in Paraguay: Alto Paraná, Amambay, Caaguazú, Caazapá, Canindeyú, Central, Concepción, Cordillera, Guairá, Ñeembucú, Paraguarí, San Pedro.

Voucher: *R. Spichiger et al. 5362* (cited in Ramella 2009: 201).

#### ERYTHROXYLACEAE

##### ►*Erythroxylum cuneifolium* (Mart.) O.E.Schulz, Pflanzenr. (Engler) [Heft 29] 4. Fam. 134: 121. 1907.

Syn.: *Erythroxylum cuneifolium* (Mart.) O.E. Schulz var. *silvaticum* O.E.Schulz; *Erythroxylum cuneifolium* (Mart.) O.E. Schulz var. *squarrosum* O.E.Schulz; *Erythroxylum microphyllum* A.St.-Hil. var. *cuneifolium* Mart.

Shrub.

Departmental distribution in Paraguay: Alto Paraná, Alto Paraguay, Amambay, Caaguazú, Canindeyú, Central, Concepción, Cordillera, Guairá, Itapúa, Ñeembucú, Paraguarí, Presidente Hayes, San Pedro.

Voucher: *J. De Egea et al. 645* (BM, CTES, FCQ, G, MO, UNR).

##### *Erythroxylum* sp.

Shrub.

Voucher: *J. De Egea & M. Vera 472* (BM, FCQ).

#### EUPHORBIACEAE

##### ►*Acalypha communis* Müll.Arg., Linnaea 34: 23. 1865.

Syn.: *Acalypha agrestis* Morong ex Britton; *Acalypha apicalis* N.E.Br.; *Acalypha communis* Müll. Arg. var. *agrestis* (Morong ex Britton) Chodat; *Acalypha communis* Müll. Arg. forma *grandifolia* Müll.Arg.; *Acalypha communis* Müll. Arg. var. *guaranitica* Chodat & Hassl.; *Acalypha communis* Müll. Arg. var. *hirta* Müll.Arg.; *Acalypha communis* Müll. Arg. var. *salicifolia* Pax & K.Hoffm.; *Acalypha communis* Müll. Arg. var. *saltensis* Pax & K.Hoffm.; *Acalypha communis* Müll. Arg. var. *tomentella* Müll.Arg.; *Acalypha communis* Müll. Arg. var. *tomentosa* Müll.Arg.; *Acalypha cordobensis* Müll.Arg.; *Acalypha cordobensis* Müll.Arg. var. *rotundata* Griseb.; *Acalypha gracilis* Griseb., nom. illeg.; *Acalypha hirta* Spreng., nom. illeg.; *Acalypha montevidensis* Klotzsch ex Pax & K.Hoffm. nom. nud.; *Acalypha paraguariensis* Chodat & Hassl.; *Acalypha variabilis* Klotzsch ex Baill. var. *angustifolia* Baill.; *Ricinocarpus cordobensis* (Müll.Arg.) Kuntze

Herb or subshrub.

Departmental distribution in Paraguay: Alto Paraná, Alto Paraguay, Amambay, Caazapá, Concepción, Cordillera, Ñeembucú, Paraguarí, Presidente Hayes.

Voucher: *J. De Egea et al. 686* (BM, CTES, FCQ, G, MO).

##### *Acalypha multicaulis Müll.Arg., Linnaea 34: 53. 1865.

Syn.: *Acalypha multicaulis* Müll.Arg. var. *glabrescens* Pax & K.Hoffm; *Acalypha pruriens* Chodat & Hassl., nom. illeg.; *Ricinocarpus multicaulis* (Müll.Arg.) Kuntze

Herb.

Departmental distribution in Paraguay: Amambay, Caaguazú, Central, Cordillera, Guairá, Ñeembucú, Paraguarí, Presidente Hayes.

Voucher: *R. Spichiger et al. 5320* (G) (http://www.ville-ge.ch/musinfo/bd/cjb/fdp/).

##### *Adelia membranifolia* (Müll.Arg.) Chodat & Hassl., Bull. Herb. Boissier, sér. 2, 5: 604. 1905.

Syn.: *Adelia membranifolia* (Müll.Arg.) Chodat & Hassl. forma *hirsuta* Chodat & Hassl.; *Adelia membranifolia* (Müll.Arg.) Chodat & Hassl. var. *spinosa* Chodat & Hassl.; *Adelia spinosa* (Chodat & Hassl.) Pax & K.Hoffm.; *Adelia spinosa* (Chodat & Hassl.) Pax & K.Hoffm. var. *hassleri* Pax & K.Hoffm.; *Adelia spinosa* (Chodat & Hassl.) Pax & K.Hoffm. var. *hirsuta* (Chodat & Hassl.) Pax & K.Hoffm.; *Ricinella membranifolia* Müll.Arg.; *Ricinella membranifolia* Müll.Arg. var. *spinosa* Chodat & Hassl.

Tree or shrub.

Departmental distribution in Paraguay: Central, Concepción, Cordillera, Ñeembucú, Presidente Hayes, San Pedro.

Voucher: *A.G. Schulz 7961* (cited in Bernardi 1984: 61, as *Adelia spinosa*).

##### *Caperonia castaneifolia* (L.) A.St.-Hil., Hist. Pl. Remarq. Bresil 245. 1826.

Syn.: *Caperonia bahiensis* Müll.Arg. forma *latior* Chodat & Hassl.; *Caperonia latior* (Chodat & Hassl.) Pax & K.Hoffm.; *Croton castaneifolius* L.

Herb.

Departmental distribution in Paraguay: Amambay, Central, Concepción, Cordillera, Itapúa, Misiones, Ñeembucú, Paraguarí, Presidente Hayes, San Pedro.

Voucher: Cited in Zuloaga et al. 2008: 2021.

##### ►*Caperonia cordata* A.St.-Hil., Hist. Pl. Remarq. Bresil 245. 1826.

Syn.: *Caperonia cordata* var. *mollis* Pax & K. Hoffm.

Herb or subshrub.

Departmental distribution in Paraguay: Caazapá, Itapúa, Ñeembucú, Presidente Hayes.

Voucher: *J. De Egea & R. Elsam 829* (BM, FCQ).

##### ►*Caperonia glabrata* Pax & K. Hoffm., Pflanzenr. (Engler) [Heft 57] 4, Fam. 147, VI: 43. 1912.

Herb. In swamps.

Departmental distribution in Paraguay: Misiones, Ñeembucú, Presidente Hayes.

Voucher: *M. Peña-Chocarro et al. 2341* (BM, CTES, FCQ, G, MO).

##### *Caperonia palustris* (L.) A.St.-Hil., Hist. Pl. Remarq. Bresil 245. 1826.

Syn.: *Croton palustris* L.

Aquatic herb.

Departmental distribution in Paraguay: Alto Paraguay, Caaguazú, Central, Ñeembucú, Presidente Hayes.

Voucher: *M.A. Walter et al. 99* (BM).

##### *Chiropetalum griseum* Griseb., Abh. Königl. Ges. Wiss. Göttingen 24: 57. 1879.

Syn.: *Argythamnia grisea* (Griseb.) Allem & Irgang; *Argythamnia mollis* Kuntze, nom. nud.; *Chiropetalum cupreum* Pax & K.Hoffm.

Subshrub.

Departmental distribution in Paraguay: Alto Paraguay, Ñeembucú, Presidente Hayes.

Voucher: Cited in Zuloaga et al. 2008: 2024.

##### ►*Chiropetalum tricoccum* (Vell.) Chodat & Hassl., Bull. Herb. Boissier, sér. 2, 5: 502. 1905.

Syn.: *Argythamnia lineata* (Klotzsch) Baill., nom. illeg.; *Argythamnia tricocca* (Vell.) Müll.Arg.; *Chiropetalum lineatum* Klotzsch, nom. nud.; *Chiropetalum tricoccum* (Vell.) Chodat & Hassl. forma *latifolius* Chodat & Hassl.; *Desfontaena tricocca* Vell.

Subshrub.

Departmental distribution in Paraguay: Central, Cordillera, Ñeembucú, Paraguarí.

Voucher: *J. De Egea & R. Elsam 794* (BM, CTES, FCQ).

##### ►*Croton argenteus* L., Sp. Pl. 2: 1004. 1753.

Syn.: *Cicca argentea* (L.) Kuntze; *Cicca montevidensis* (Klotzsch ex Baill.) Kuntze; *Croton integer* (Chodat) Radcl.-Sm. & Govaerts; *Julocroton argenteus* (L.) Didr.; *Julocroton camporum* Chodat & Hassl.; *Julocroton elaeagnoides* S.Moore; *Julocroton integer* Chodat; *Julocroton integer* Chodat forma *parvifolia* Chodat & Hassl.; *Julocroton linearifolius* (Chodat & Hassl.) Croizat; *Julocroton montevidensis* Klotzsch ex Müll.Arg.; *Julocroton montevidensis* Klotzsch ex Müll.Arg. var. *linearifolius* Chodat & Hassl.

Herb.

Departmental distribution in Paraguay: Alto Paraguay, Amambay, Caazapá, Canindeyú, Central, Itapúa, Ñeembucú, Presidente Hayes.

Voucher: *F. Mereles 9723* (FCQ).

##### ►*Croton bonplandianus* Baill., Adansonia 4: 339. 1864.

Syn.: *Croton pauperulus* Müll. Arg.; *Croton rivinoides* Chodat; *Croton sparsiflorus* Morong

Herb.

Departmental distribution in Paraguay: Alto Paraguay, Cordillera, Ñeembucú, Presidente Hayes.

Voucher: *M. Perez et al. 1* (FCQ, UNP).

##### *Croton eskuchei* Ahumada, Darwiniana 37(1-2): 172. 1999.

Subshrub.

Departmental distribution in Paraguay: Central, Ñeembucú.

Voucher: *U.G. Eskuche 2527* (CTES) (cited in Ahumada 1999: 174).

##### ►*Croton gracilipes* Baill., Adansonia 4: 333. 1864.

Syn.: *Croton hasslerianus* Chodat

Shrub.

Departmental distribution in Paraguay: Alto Paraguay, Central, Concepción, Cordillera, Ñeembucú, Paraguarí, Presidente Hayes.

Voucher: *M. Peña-Chocarro et al. 2243* (BM, FCQ).

##### ►*Croton subpannosus* Müll.Arg. ex Griseb., Abh. Königl. Ges. Wiss. Göttingen 19: 96. 1874.

Syn.: *Croton dentosus* Griseb.; *Julocroton brittonianum* Morong; *Julocroton serratus* Müll.Arg.; *Julocroton subpannosus* Müll.Arg.; *Julocroton subpannosus* Müll.Arg. var. *chacoensis* Croizat; *Julocroton subpannosus* Müll.Arg. var. *formosensis* Croizat; *Julocroton subpannosus* Müll.Arg. var. *posadensis* Croizat; *Julocroton subpannosus* Müll.Arg. var. *uruguayensis* Croizat

Subshrub.

Departmental distribution in Paraguay: Cordillera, Ñeembucú.

Voucher: *M. Peña-Chocarro et al. 2242* (BM, CTES, FCQ, G, MO, SI).

##### ►*Croton urucurana* Baill., Adansonia 4: 335. 1864.

Syn.: *Croton dracona* Larragaña; *Croton succiruber* D.Parodi

Tree.

Departmental distribution in Paraguay: Alto Paraná, Amambay, Caazapá, Canindeyú, Concepción, Cordillera, Guairá, Itapúa, Ñeembucú, Paraguarí.

Voucher: *C. Vogt 132* (CTES, FCQ).

##### *Croton villosissimus (Chodat & Hassl.) Radcl.-Sm. & Govaerts, Kew Bull. 52: 186. 1997.

Syn.: *Julocroton villosissimus* Chodat & Hassl.

Shrub.

Departmental distribution in Paraguay: Canindeyú, Ñeembucú.

Voucher: *R. Spichiger et al. 5240* (G) (http://www.ville-ge.ch/musinfo/bd/cjb/fdp/).

##### *Croton* sp.

Subshrub.

Voucher: *M. Peña-Chocarro et al. 1975* (BM, CTES, FCQ, G, MO).

##### ►*Euphorbia klotzschii* Oudejans, Phytologia 67: 46. 1989.

Syn.: *Anisophyllum ovalifolium* Klotzsch & Garcke; *Chamaesyce ovalifolia* (Klotzsch & Garcke) Croizat; *Chamaesyce serpens* (Kunth) Small var. *montevidensis* (Boiss.) Croizat; *Euphorbia ovalifolia* (Klotzsch & Garcke) Boiss., nom. illeg.; *Euphorbia ovalifolia* var. *montevidensis* Boiss.; *Euphorbia serpens* Kunth var. *montevidensis* (Boiss.) Cabrera

Herb.

Departmental distribution in Paraguay: Alto Paraguay, Ñeembucú, Paraguarí.

Voucher: *M. Vera et al. 238* (BM, FCQ).

##### * *Microstachys ramosissima* A.St.-Hil., Prodr. (DC.) 15(2): 1187. 1866.

Syn.: *Actinostemon anisandrus* (Griseb.) Pax; *Actinostemon luquense* Moroni; *Dactylostemon anisandrus* Griseb.; *Sebastiania anisandra* (Griseb.) Lillo; *Sebastiania brasiliensis* Spreng. var. *ramosissima* (A.St.-Hil.) Müll.Arg.

Tree or shrub.

Departmental distribution in Paraguay: Alto Paraná, Alto Paraguay, Amambay, Caaguazú, Caazapá, Canindeyú, Central, Concepción, Cordillera, Guairá, Itapúa, Ñeembucú, Paraguarí, Presidente Hayes, San Pedro.

Voucher: *M. Peña-Chocarro et al. 2283* (BM, CTES, FCQ, G, MO, PY, SI).

Note: This taxon is treated as *Sebastiania* by Melo (2006) and Athiê de Souza (2011), but the combination has not yet been published; it was treated as *Sebastiania brasiliensis* by Zuloaga et al. (2008), but that species is confined to coastal Brazil (Athiê de Souza, pers. comm.).

##### ►*Philyra brasiliensis* Klotzsch, Arch. Naturgesch. 1: 199. 1841.

Syn.: *Argythamnia brasiliensis* (Klotzsch) Müll.Arg.; *Ditaxis brasiliensis* (Klotzsch) Baill.

Tree or shrub.

Departmental distribution in Paraguay: Central, Cordillera, Ñeembucú, Paraguarí.

Voucher: *L. Bernardi 18466* (BM).

##### *Sapium haematospermum Müll.Arg., Linnaea 34: 217. 1865,

Syn.: *Excoecaria biglandulosa* (L.) Müll.Arg. var. *stenophylla* Müll.Arg.; *Excoecaria haematosperma* (Müll.Arg.) Müll.Arg.; *Sapium aucuparium* Jacq. var. *stenophyllum* (Müll.Arg.) Griseb.; *Sapium biglandulosum* (L.) Müll.Arg. forma *longifolium* (Müll.Arg.) Müll.Arg.; *Sapium biglandulosum* (L.) Müll.Arg. var. *longifolium* Müll.Arg.; *Sapium biglandulosum* (L.) Müll.Arg. var. *stenophyllum* Müll.Arg.; *Sapium gibertii* Hemsl.; *Sapium haematospermum* Müll.Arg. forma *arborea* Chodat & Hassl.; *Sapium linearifolium* Hemsl.; *Sapium longifolium* (Müll.Arg.) Huber; *Sapium rojasii* H.Lév.; *Sapium stenophyllum* (Müll.Arg.) Huber; *Stillingia sylvatica* L. var. *paraguayensis* Morong

Shrub.

Departmental distribution in Paraguay: Alto Paraguay, Amambay, Caaguazú, Caazapá, Canindeyú, Central, Concepción, Cordillera, Ñeembucú, Paraguarí, Presidente Hayes.

Voucher: *S. Keel & L. Spinzi v-161* (FCQ).

##### *Sebastiania commersoniana (Baill.) L.B.Sm. & Downs, Fl. Ilustr. Catarin. 1 (Euforbiác.): 308. 1988.

Syn.: *Gymnanthes klotzschiana* Müll.Arg., nom. illeg.; *Gymnanthes marginata* Baill., nom. nud.; *Sebastiania klotzschiana* (Müll.Arg.) Müll.Arg.; *Sebastiania klotzschiana* (Müll.Arg.) Müll.Arg. var. *brachyclada* (Müll.Arg.) Pax & K.Hoffm.; *Sebastiania klotzschiana* (Müll.Arg.) Müll.Arg. var. *trichoneura* Müll.Arg.; *Sebastiania commersoniana* Baill.

Tree.

Departmental distribution in Paraguay: Alto Paraná, Caazapá, Canindeyú, Cordillera, Ñeembucú, Paraguarí.

Voucher: *R. Spichiger & et al. 5341* (G) (http://www.ville-ge.ch/musinfo/bd/cjb/fdp/).

##### ►*Tragia geraniifolia* Klotzsch ex Baill., Étude Euphorb. 461. 1858.

Syn.: *Tragia geraniifolia* Klotzsch ex Baill. var. *multifida* Griseb.

Herb.

Departmental distribution in Paraguay: Alto Paraguay, Ñeembucú.

Voucher: *M. Peña-Chocarro et al. 2240* (BM, CTES, FCQ, G, MO).

##### ►*Tragia volubilis* L., Sp. Pl. 2: 980. 1753.

Syn.: *Tragia volubilis* L. var. *guaranitica* Chodat & Hassl.

Herb.

Departmental distribution in Paraguay: Alto Paraná, Alto Paraguay, Amambay, Caaguazú, Caazapá, Canindeyú, Concepción, Ñeembucú, Paraguarí, San Pedro.

Voucher: *M. Peña-Chocarro et al. 2300* (BM, FCQ).

#### FABACEAE

##### ►*Aeschynomene denticulata* Rudd, Contr. U.S. Natl. Herb. 32: 69. 1955.

Herb.

Departmental distribution in Paraguay: Alto Paraguay, Cordillera, Guairá, Ñeembucú.

Voucher: *M. Peña-Chocarro et al. 2315* (BM, CTES, FCQ, G, MO, SI).

##### *Aeschynomene montevidensis Vogel, Linnaea 12: 83. 1838.

Syn.: *Aeschynomene montevidensis* Vogel var. *microphylla* Chodat & Hassl.; *Macromiscus brasiliensis* Turcz.

Herb or shrub.

Departmental distribution in Paraguay: Alto Paraná, Amambay, Canindeyú, Central, Concepción, Cordillera, Guairá, Ñeembucú, Presidente Hayes, San Pedro.

Voucher: *S. Keel & L. Spinzi 1475* (FCQ).

##### ►*Aeschynomene sensitiva* Sw., Prodr. 107. 1788.

Syn.: *Aeschynomene sensitiva* Sw. var. *paucifoliolata* Chodat & Hassl.; *Aeschynomene sensitiva* P.Beauv., nom. illeg.

Shrub.

Departmental distribution in Paraguay: Alto Paraguay, Amambay, Central, Cordillera, Guairá, Ñeembucú, Paraguarí, Presidente Hayes.

Voucher: *M.A. Walter 208* (BM).

##### *Anadenanthera peregrina* (L.) Speg., Physis (Buenos Aires) 6: 314. 1923.

Syn.: *Acacia angustiloba* DC.; *Acacia microphylla* Willd.; *Acacia niopo* (Humb. & Bonpl. ex Willd.) Kunth; *Acacia peregrina* (L.) Willd.; *Acacia falcata* (Benth.) Speg.; *Acacia peregrina* (L.) Speg. var. *falcata* (Benth.) Altschul; *Mimosa acacioides* Benth.; *Mimosa niopo* (Humb. & Bonpl. ex Willd.) Poir.; *Mimosa parvifolia* Poir.; *Mimosa peregrina* L.; *Niopa peregrina* (L.) Britton & Rose; *Piptadenia falcata* Benth.; *Piptadenia niopo* (Humb. & Bonpl. ex Willd.) Spruce; *Piptadenia peregrina* (L.) Benth.; *Piptadenia peregrina* (L.) Benth. var. *falcata* (Benth.) Chodat & Hassl.

Tree or shrub.

Departmental distribution in Paraguay: Alto Paraguay, Amambay, Canindeyú, Ñeembucú, Paraguarí.

Voucher: *L. Bernardi 18498* (BM).

##### *Bauhinia bauhinioides* (Mart.) J.F.Macbr., Contr. Gray Herb. 59: 22. 1919.

Syn.: *Bauhinia microphylla* Vogel; *Pauletia bauhinioides* (Mart.) A.Schmitz; *Perlebia bauhinioides* Mart.

Tree or shrub.

Departmental distribution in Paraguay: Alto Paraguay, Central, Concepción, Cordillera, Ñeembucú.

Voucher: *Reales 276* (LIL) (cited in Fortunato 1986: 544).

##### *Caesalpinia paraguariensis* (D.Parodi) Burkart, Darwiniana 10(1): 26. 1952.

Syn.: *Acacia paraguariensis* D.Parodi; *Caesalpinia coriaria* Micheli, nom. illeg.; *Caesalpinia melanocarpa* Griseb.

Tree.

Departmental distribution in Paraguay: Alto Paraguay, Central, Concepción, Ñeembucú, Presidente Hayes, San Pedro.

Voucher: *J. De Egea et al. 703* (BM, CTES, FCQ, G, MO, PY, UNR).

##### ►*Chamaecrista nictitans* (L.) Moench var. *patellaria* (DC. ex Collad.) Kartesz & Gandhi, Phytologia 71(2): 93. 1991.

Syn.: *Cassia biglandulosa* Bertol.; *Cassia patellaria* DC. ex Collad.; *Cassia patellaria* DC. ex Collad. var. *longifolia* Benth.; *Cassia patellaria* DC. ex Collad. var. *ramosa* Vogel; *Cassia villosissima* (Britton & Rose) Lundell; *Chamaecrista nictitans* var. *ramosa* (Vogel) H.S.Irwin & Barneby; *Chamaecrista patellaria* (DC. ex Collad.) Greene; *Chamaecrista villosissima* Britton & Rose

Herb.

Departmental distribution in Paraguay: Guairá, Ñeembucú.

Voucher: *F. Mereles 9728* (FCQ).

##### *Chamaecrista rotundifolia (Pers.) Greene, Pittonia 4(20D): 31. 1899.

Syn.: *Cassia bifoliolata* DC. ex Collad.; *Cassia bifoliolata* DC. ex Collad. var. *pentandra* (Raddi) Desv.; *Cassia bifoliolata* DC. ex Collad. var. *rotundifolia* (Pers.) Desv., nom. illeg.; *Cassia fabaginifolia* Kunth; *Cassia monophylla* Vell.; *Cassia pentandra* Raddi; *Cassia pentandria* Larrañaga, nom. illeg.; *Cassia rotundifolia* Pers.; *Cassia tenuivenosa* A.P.D.Jones; *Chamaecrista bifoliolata* (DC. ex Collad.) Greene

Herb or subshrub.

Departmental distribution in Paraguay: Alto Paraguay, Amambay, Caaguazú, Caazapá, Central, Cordillera, Ñeembucú.

Voucher: *A.G. Schulz 7754* (G) (http://www.ville-ge.ch/musinfo/bd/cjb/fdp/).

##### ►*Crotalaria micans* Link, Enum. Hort. Berol. Alt. 2: 228. 1822,

Syn.: *Crotalaria anagyroides* Kunth; *Crotalaria brachystachys* Benth.; *Crotalaria dombeyana* DC.; *Crotalaria stipulata* Vell.; *Crotalaria triphylla* Vell.

Herb or subshrub.

Departmental distribution in Paraguay: Alto Paraguay, Ñeembucú, San Pedro.

Voucher: *M. Ortiz 133* (FCQ).

##### ►*Desmanthus virgatus* (L.) Willd., Sp. Pl., ed. 4 [Willdenow] 4(2): 1047. 1806.

Syn.: *Acacia angustisiliqua* (Lam.) Desf.; *Acacia virgata* (L.) Gaertn.; *Acuan depressum* (Humb. & Bonpl. ex Willd.) Kuntze; *Acuan texanum* Britton & Rose; *Acuan tracyi* Britton & Rose; *Desmanthus depressus* Humb. & Bonpl. ex Willd.; *Desmanthus pratorum* Macfad.; *Desmanthus tatuhyensis* auct. non Hoehne; *Desmanthus tenellus* DC.; *Desmanthus virgatus* (L.) Willd. var. *depressus* (Humb. & Bonpl. ex Willd.) B.L.Turner; *Mimosa angustisiliqua* Lam., nom. superfl.; *Mimosa depressa* (Humb. & Bonpl. ex Willd.) Poir.; *Mimosa virgata* L.

Herb or subshrub.

Departmental distribution in Paraguay: Alto Paraguay, Guairá, Ñeembucú, San Pedro.

Voucher: *M. Peña-Chocarro et al. 2224* (BM, CTES, FCQ, MO).

##### ►*Desmodium cuneatum* Hook. & Arn., Bot. Misc. 3: 195. 1832.

Syn.: *Meibomia cuneata* (Hook. & Arn.) Hoehne; *Meibomia brevipes* (Vogel) Kuntze

Subshrub.

Departmental distribution in Paraguay: Alto Paraguay, Central, Guairá, Misiones, Ñeembucú, Paraguarí, Presidente Hayes.

Voucher: *F. Mereles 9735* (FCQ).

##### ►*Desmodium incanum* DC., Prodr. (DC.) 2: 332. 1825.

Syn.: *Desmodium canum* (J.F.Gmel.) Schinz & Thell.; *Desmodium frutescens* Schindl.; *Desmodium supinum* (Sw.) DC.; *Hedysarum canescens* Mill.; *Hedysarum canum* J.F.Gmel., nom. illeg.; *Hedysarum frutescens* Jacq., nom. illeg.; *Hedysarum incanum* Sw., nom. illeg.; *Hedysarum racemosum* Aubl.; *Meibomia cana* (J.F.Gmel.) Blake; *Meibomia incana* (DC.) Vail; *Meibomia incana* (Sw.) Hoehne, nom. illeg.

Herb.

Departmental distribution in Paraguay: Central, Itapúa, Misiones, Ñeembucú, Paraguarí.

Voucher: *M. Peña-Chocarro et al. 1997* (BM, CTES, FCQ, G, MO).

##### *Discolobium pulchellum* Benth., Comm. Legum. Gen. 42. 1837.

Shrub.

Departmental distribution in Paraguay: Alto Paraguay, Central, Ñeembucú.

Voucher: *A.G. Schulz 7778* (SI) (cited in Zuloaga et al. 2008: 2179).

##### ►*Dolichopsis paraguariensis* (Benth.) Hassl., Bull. Herb. Boissier, sér. 2, 7(3): 162. 1907.

Syn.: *Vigna paraguariensis* Benth.

Herb or climber.

Departmental distribution in Paraguay: Alto Paraguay, Caazapá, Ñeembucú, Presidente Hayes.

Voucher: *M. Peña-Chocarro et al. 2313* (BM, CTES, FCQ, G, MO, SI).

##### *Enterolobium contortisiliquum* (Vell.) Morong, Ann. New York Acad. Sci. 7: 102. 1893.

Syn.: *Acacia melalobiata* Rojas Acosta; *Calliandra pacara* Griseb.; *Enterolobium glaucescens* Mart.; *Enterolobium timbouva* auct. non Mart.; *Feuilleea pacara* (Griseb.) Kuntze; *Mimosa contortisiliqua* Vell.

Tree.

Departmental distribution in Paraguay: Alto Paraná, Central, Cordillera, Guairá, Ñeembucú, Paraguarí.

Voucher: *Z. Gálvez et al. 03* (FCQ, UNP).

##### *Eriosema platycarpon Micheli, Mém. Soc. Phys. Genève 28(7): 34. 1883.

Herb or subshrub.

Departmental distribution in Paraguay: Amambay, Canindeyú, Concepción, Cordillera, Guairá, Itapúa, Ñeembucú, Paraguarí, San Pedro.

Voucher: *R. Spichiger et al. 5227* (CTES, G) (http://www.ville-ge.ch/musinfo/bd/cjb/fdp/).

##### *Erythrina crista-galli* L., Mant. Pl. 1: 99. 1767.

Syn.: *Coladendron crista-galli* (L.) Kuntze; *Erythrina fasciculata* Benth.; *Erythrina laurifolia* Jacq.; *Erythrina pulcherrima* Tod.; *Erythrina speciosa* Tod.; *Micropteryx crista-galli* (L.) Walp.; *Micropteryx fasciculata* (Benth.) Walp.; *Micropteryx laurifolia* (Jacq.) Walp.

Tree or shrub.

Departmental distribution in Paraguay: Alto Paraná, Caaguazú, Canindeyú, Cordillera, Itapúa, Ñeembucú, Paraguarí.

Voucher: *L. Bernardi 18366* (BM).

##### *Erythrina dominguezii* Hassl., Physis (Buenos Aires) 6: 123. 1922.

Syn.: *Erythrina chacoensis* Speg., nom. nud.

Tree.

Departmental distribution in Paraguay: Alto Paraguay, Canindeyú, Ñeembucú.

Voucher: *L. Bernardi 18518* (BM).

##### *Galactia marginalis Benth., Comm. Legum. Gen. 62. 1837.

Syn.: *Cologania heterophylla* Gillies ex Hook. & Arn.; *Galactia heterophylla* (Gillies ex Hook. & Arn.) Vail, nom. illeg.

Herb or subshrub.

Departmental distribution in Paraguay: Ñeembucú, Paraguarí.

Voucher: *T. Rojas 12645* (AS) (http://www.tropicos.org/).

##### *Galactia* sp.

Herb?. habit not noted on label.

Voucher: *A.G. Schulz 7699* (G) (http://www.ville-ge.ch/musinfo/bd/cjb/fdp/).

##### *Geoffroea spinosa* Jacq., Enum. Syst. Pl. 28. 1760.

Syn.: *Geoffraea bredemeyeri* Kunth; *Geoffraea striata* (Willd.) J.F.Macbr., comb. superfl.; *Geoffraea striata* (Willd.) Morong; *Geoffroea superba* Bonpl.; *Robinia striata* Willd.

Tree.

Departmental distribution in Paraguay: Alto Paraguay, Central, Concepción, Ñeembucú, Paraguarí, Presidente Hayes.

Voucher: *L. Bernardi 18472* (BM).

##### ►*Gleditsia amorphoides* (Griseb.) Taub., Ber. Deutsch. Bot. Ges. 10(10): 638. 1892.

Syn.: *Garugandra amorphoides* Griseb.

Tree.

Departmental distribution in Paraguay: Central, Cordillera, Ñeembucú, Paraguarí.

Voucher: *J. De Egea et al. 724* (BM, CTES, FCQ, G, MO, PY, UNR).

##### ►*Holocalyx balansae* Micheli, Mém. Soc. Phys. Genève 28(7): 41. 1883.

Tree.

Departmental distribution in Paraguay: Alto Paraná, Amambay, Caazapá, Canindeyú, Central, Cordillera, Itapúa, Ñeembucú, Paraguarí, San Pedro.

Voucher: *J. De Egea & R. Elsam 825* (BM, CTES, FCQ, G, MO, SI).

##### ►*Indigofera asperifolia* Bong. ex Benth., Ann. Nat. Hist. 3: 431. 1839.

Syn.: *Anila asperifolia* (Bong. ex Benth.) Kuntze; *Indigofera asperifolia* Hochst. ex Baker, nom. illeg.; *Indigofera retrusa* N.E.Br.

Herb.

Departmental distribution in Paraguay: Caazapá, Canindeyú, Guairá, Misiones, Ñeembucú.

Voucher: *J. De Egea et al. 633* (FCQ).

##### *Indigofera microcarpa Desv., J. Bot. Agric. 3: 79. 1814.

Syn.: *Indigofera sabulicola* Benth.

Herb.

Departmental distribution in Paraguay: Central, Cordillera, Ñeembucú.

Voucher: *T. Rojas 12621* (AS) (http://www.tropicos.org/).

##### *Inga vera Willd. subsp. *affinis* (DC.) T.D.Penn., Gen. Inga: Bot. 716. 1997.

Syn.: *Feuilleea affinis* (DC.) Kuntze; *Feuilleea meissneriana* (Miq.) Kuntze; *Feuilleea uruguensis* (Hook. & Arn.) Kuntze; *Inga acutifolia* Benth.; *Inga affinis* DC.; *Inga arinensis* Hoehne; *Inga arrabideae* Steud.; *Inga meissneriana* Miq.; *Inga spuria* Humb. & Bonpl. ex Willd var. *sordida* Benth.; *Inga soluta* Pittier; *Inga uraguensis* Hook. & Arn.; *Inga uraguensis* Hook. & Arn. var. *parvifolia* Chodat & Hassl.; *Inga uraguensis* Hook. & Arn.var. *parvifolia* forma *tomentosula* Chodat & Hassl.; *Inga velloziana* Mart.; *Mimosa umbellata* Vell.

Tree.

Departmental distribution in Paraguay: Alto Paraná, Amambay, Caaguazú, Caazapá, Canindeyú, Central, Concepción, Cordillera, Ñeembucú, Paraguarí.

Voucher: *J. De Egea et al. 826* (FCQ).

##### *Lupinus gibertianus* C.P.Sm., Sp. Lupinorum 13: 206. 1940.

Syn.: *Cytisus heptaphyllus* Vell.; *Lupinus aspersus* C.P.Sm.; *Lupinus bonplandianus* C.P.Sm.; *Lupinus hassleranus* C.P.Sm.; *Lupinus heptaphyllus* (Vell.) Hassl.; *Lupinus heptaphyllus* (Vell.) Hassl. forma *hilarianus* Benth.; *Lupinus heptaphyllus* (Vell.) Hassl. forma *typicus* Hassl., nom. illeg.; *Lupinus hilarianus* Benth., nom. superfl.; *Lupinus propedubius* C.P.Sm.; *Lupinus sanctae-anae* C.P.Sm.; *Lupinus subumbellatus* C.P.Sm.

Herb.

Departmental distribution in Paraguay: Itapúa, Ñeembucú.

Voucher: *L. Bernardi 18479* (BM).

##### *Macroptilium lathyroides* (L.) Urb., Symb. Antill. 9(4): 457. 1928.

Syn.: *Lotus maritimus* Vell., nom. illeg.; *Phaseolus crotalarioides* Mart. ex Benth.; *Phaseolus hastifolius* C.Mart. ex Benth.; *Phaseolus lathyroides* L.; *Phaseolus lathyroides* L. forma *chacoensis* Hassl.; *Phaseolus lathyroides* L. forma *hirsutus* Hassl.; *Phaseolus lathyroides* L. forma *reapandus* Hassl.; *Phaseolus lathyroides* L. forma *typicus* Hassl., nom. illeg.; *Phaseolus lathyroides* L. var. *genuinus* Hassl., nom. illeg.; *Phaseolus lathyroides* L. var. *semierectus* (L.) Hassl.; *Phaseolus maritimus* (Vell.) Benth.; *Phaseolus psoraleoides* Wight & Arn.; *Phaseolus semierectus* L.; *Phaseolus semierectus* L. var. *angustifolius* Benth.; *Phaseolus semierectus* L. var. *nanus* Benth.; *Phaseolus semierectus* L. var. *subhastatus* Benth.; *Phaseolus strictus* Braun & Bouché

Herb.

Departmental distribution in Paraguay: Alto Paraguay, Amambay, Caazapá, Central, Itapúa, Ñeembucú, Presidente Hayes.

Voucher: *M. Peña-Chocarro et al. 1998* (BM, FCQ).

##### ►*Microlobius foetidus* (Jacq.) M.Sousa & G.Andrade subsp. *paraguensis* (Benth.) M.Sousa & G.Andrade, Anales Inst Biol. Univ. Nac. Autón. México, Bot. 63: 106. 1992.

Syn.: *Goldmania paraguensis* (Benth.) Brenan; *Piptadenia paraguensis* (Benth.) Lindm.; *Piptadenia quadrifolia* N.E.Br.; *Pithecellobium paraguense* Benth.

Tree.

Departmental distribution in Paraguay: Alto Paraguay, Central, Concepción, Ñeembucú, Paraguarí, Presidente Hayes.

Voucher: *C. Vogt 170* (CTES, FACEN, FCQ).

##### *Mimosa caaguazuensis* Barneby, Mem. New York Bot. Gard. 65: 704. 1991.

Syn.: *Mimosa nervosa* Bong. ex Benth. var. *macrophylla* Hassl.; *Mimosa procurrens* auct. non Benth.

Herb or subshrub.

Departmental distribution in Paraguay: Caaguazú, Canindeyú, Cordillera, Misiones, Ñeembucú.

Voucher: Cited in Zuloaga et al. 2008: 2227.

##### *Mimosa guaranitica Chodat & Hassl., Bull. Herb. Boissier, sér. 2, 4(6): 555. 1904.

Syn.: *Mimosa guaranitica* Chodat & Hassl. forma *heterotricha* Burkart

Subshrub.

Departmental distribution in Paraguay: Amambay, Central, Cordillera, Ñeembucú, Paraguarí, San Pedro.

Voucher: *R. Spichiger et al. 5238* (G) (http://www.ville-ge.ch/musinfo/bd/cjb/fdp/).

##### *Mimosa hexandra* Micheli, Mém. Soc. Phys. Genève 30(2/7): 91. 1889.

Syn.: *Mimosa acanthophora* Harms; *Mimosa bimucronata* (DC.) Kuntze forma *micheliana* Hassl.; *Mimosa bimucronata* (DC.) Kuntze forma *vepres* (Lindm.) Hassl.; *Mimosa bimucronata* (DC.) Kuntze subsp. *hexandra* (Micheli) Hassl.; *Mimosa bimucronata* (DC.) Kuntze var. *gymnocarpa* Hassl.; *Mimosa bimucronata* (DC.) Kuntze var. *hexandra* (Micheli) J.F.Macbr.; *Mimosa coroncoro* Killip & Dugand; *Mimosa fascifolia* Rizzini; *Mimosa hexandra* Micheli var. *tropica* S.Moore; *Mimosa hexandra* var. *vepres* (Lindm.) Chodat & Hassl.; *Mimosa vepres* Lindm.

Shrub.

Departmental distribution in Paraguay: Alto Paraguay, Central, Concepción, Ñeembucú, Paraguarí, Presidente Hayes.

Voucher: Cited in Zuloaga et al. 2008: 2235.

##### ►*Mimosa strigillosa* Torr. & A.Gray, Fl. N. Amer. 1(3): 399. 1840.

Syn.: *Mimosa dolichocephala* Harms; *Mimosa dolichocephala* Harmsvar. *sabulicola* (Chodat & Hassl.) Hassl.; *Mimosa sabulicola* Chodat & Hassl.

Herb or subshrub.

Departmental distribution in Paraguay: Alto Paraguay, Central, Concepción, Ñeembucú.

Voucher: *M. Peña-Chocarro et al. 1975* (BM, CTES, FCQ, G, MO).

##### *Peltophorum dubium (Spreng.) Taub., Nat. Pflanzenfam. 3(3): 176. 1892.

Syn.: *Brasilettia dubia* (Spreng.) Kuntze; *Caesalpinia dubia* Spreng.; *Peltophorum vogelianum* Benth.; *Peltophorum vogelianum* Benth. forma *ferruginea* Chodat & Hassl.; *Peltophorum vogelianum* Benth.Benth. forma *glabrata* Chodat & Hassl.; *Peltophorum vogelianum* Benth. forma *intermedia* Chodat & Hassl.

Tree.

Departmental distribution in Paraguay: Alto Paraná, Amambay, Caaguazú, Caazapá, Canindeyú, Cordillera, Guairá, Itapúa, Ñeembucú, Paraguarí.

Voucher: Cited in Vogt and Mereles 2005: 10.

##### *Prosopis affinis* Spreng., Syst. Veg., ed. 16 [Sprengel] 2: 326. 1825.

Syn.: *Prosopis algarrobilla* Griseb.; *Prosopis algarrobilla* Griseb. var. *nandubay* (Lorentz ex Griseb.) Hassl.; *Prosopis nandubey* Lorentz ex Griseb.

Tree.

Departmental distribution in Paraguay: Alto Paraguay, Central, Concepción, Cordillera, Ñeembucú, Paraguarí.

Voucher: *J. De Egea et al. 723* (BM, CTES, FCQ, G, MO, UNR).

##### *Prosopis alba* Griseb., Abh. Königl. Ges. Wiss. Göttingen 19: 131. 1874.

Syn.: *Prosopis atacamensis* Phil.; *Prosopis siliquastrum* (Cav. ex Lag.) DC. var. *longisiliqua* Phil.

Tree.

Departmental distribution in Paraguay: Alto Paraguay, Central, Ñeembucú, Presidente Hayes.

Voucher: *L. Bernardi 18422* (BM).

##### *Prosopis nigra* (Griseb.) Hieron., Bol. Acad. Nac. Ci. 4: 283. 1881.

Syn.: *Prosopis algarrobilla* Griseb. var. *nigra* Griseb.; *Prosopis dulcis* Kunth var. *australis* Benth.

Tree.

Departmental distribution in Paraguay: Alto Paraguay, Central, Misiones, Ñeembucú, Paraguarí, Presidente Hayes.

Voucher: *J. De Egea et al. 722* (FCQ).

##### *Prosopis* sp.

Tree.

Voucher: *A. Sánchez et al. 08* (FCQ, UNP).

##### ►*Pterogyne nitens* Tul., Ann. Sci. Nat., Bot. Sér. 2, 20: 140. 1843.

Syn.: *Pterogyne nitens* forma *parvifolia* Chodat & Hassl.

Tree.

Departmental distribution in Paraguay: Alto Paraguay, Amambay, Caaguazú, Canindeyú, Central, Cordillera, Ñeembucú, Paraguarí.

Voucher: *M. Peña-Chocarro et al. 2289* (BM, CTES, FCQ, G, MO, PY, SI).

##### *Rhynchosia corylifolia* Mart. ex Benth., Fl. Bras. (Martius) 15(1B): 202. 1862.

Syn.: *Rhynchosia corylifolia* Mart. ex Benth. forma *glabrior* Chodat & Hassl.; *Rhynchosia corylifolia* Mart. ex Benth. var. *discolor* Chodat & Hassl.; *Rhynchosia corylifolia* Mart. ex Benth. var. erecta Micheli ; *Rhynchosia corylifolia* Mart. ex Benth. var. *orbiculata* Chodat & Hassl.; *Rhynchosia reticulata* (Sw.) DC. forma *oblongifolia* Chodat & Hassl.; *Rhynchosia reticulata* (Sw.) DC. forma *ovalifolia* Chodat & Hassl.; *Rhynchosia reticulata* (Sw.) DC. forma *subumbellata* Chodat & Hassl.

Herb.

Departmental distribution in Paraguay: Alto Paraná, Caaguazú, Caazapá, Canindeyú, Central, Concepción, Cordillera, Guairá, Itapúa, Misiones, Ñeembucú, Paraguarí, Presidente Hayes, San Pedro.

Voucher: *E. Hassler 2276* (cited in Grear 1978: 119).

##### *Rhynchosia rojasii* Hassl., Repert. Spec. Nov. Regni Veg. 7: 77. 1909.

Syn.: *Rhynchosia ituana* Hoehne

Climber.

Departmental distribution in Paraguay: Alto Paraná, Amambay, Caaguazú, Guairá, Ñeembucú.

Voucher: *P. Jörgensen 4889* (A, F, NY, S, US) (cited in Grear 1978: 101).

##### ► *Senegalia bonariensis* (Gillies ex Hook. & Arn.) Seigler & Ebinger, Phytologia 88(1): 50. 2006.

Syn.: *Acacia bonariensis* Gillies ex Hook. & Arn.

Tree or shrub.

Departmental distribution in Paraguay: Caazapá, Canindeyú, Central, Cordillera, Misiones, Ñeembucú, Presidente Hayes.

Voucher: *J. De Egea et al. 728* (BM, CTES, FCQ, G, MO, UNR).

##### ►*Senna occidentalis* (L.) Link, Handbuch 2: 140. 1829.

Syn.: *Cassia caroliniana* Walter; *Cassia ciliata* Raf.; *Cassia falcata* L.; *Cassia foetida* Pers., nom. illeg.; *Cassia macradena* Collad.; *Cassia obliquifolia* Schrank; *Cassia occidentalis* L.; *Cassia occidentalis* L. var. *aristata* Collad.; *Cassia planisiliqua* L.; *Cassia planisiliqua* Lam., nom. illeg.; *Cassia plumieri* DC.; *Ditremexa occidentalis* (L.) Britton & Rose ex Britton & Wilson; *Senna occidentalis* (L.) Roxb., comb. superfl.

Herb or subshrub.

Departmental distribution in Paraguay: Alto Paraguay, Amambay, Caaguazú, Caazapá, Central, Cordillera, Guairá, Ñeembucú, Paraguarí, Presidente Hayes.

Voucher: *J. De Egea et al. 682* (BM, CTES, FCQ, G, MO, PY, SI, UNR).

##### ►*Senna pendula* (Willd.) H.S.Irwin & Barneby var. *paludicola* H.S.Irwin & Barneby, Mem. New York Bot. Gard. 35: 393. 1982.

Syn.: *Cassia bicapsularis* L. forma *pilosa* Chodat & Hassl.

Shrub.

Departmental distribution in Paraguay: Alto Paraguay, Central, Cordillera, Ñeembucú, Paraguarí, Presidente Hayes.

Voucher: *M. Morinigo et al. 05* (FCQ, UNP).

##### *Sesbania virgata* (Cav.) Pers., Syn. Pl. [Persoon] 2(2): 316. 1807.

Syn.: *Aeschynomene virgata* Cav.; *Coronilla virgata* (Cav.) Willd.; *Coursetia virgata* (Cav.) DC.; *Sesbania marginata* Benth.

Shrub.

Departmental distribution in Paraguay: Alto Paraguay, Central, Canindeyú, Concepción, Cordillera, Ñeembucú, Paraguarí, Presidente Hayes.

Voucher: *J. De Egea & R. Elsam 830* (BM, CTES, FCQ, G, MO, PY, SI, UNR).

##### ►*Stylosanthes guianensis* (Aubl.) Sw., Kongl. Vetensk. Acad. Nya Handl. 10: 301. 1789.

Syn.: *Trifolium guianense* Aubl.; *Stylosanthes gracilis* Kunth var. *vulgaris* Burkart; *Stylosanthes guianensis* (Aubl.) Sw. forma *esetosa* Hassl.; *Stylosanthes guianensis* forma *viscosissima* Hassl.

Herb.

Departmental distribution in Paraguay: Amambay, Caaguazú, Canindeyú, Concepción, Cordillera, Ñeembucú, Presidente Hayes, San Pedro.

Voucher: *F. Mereles 9731* (FCQ).

##### *Vachellia caven (Molina) Seigler & Ebinger, Phytologia 87(3): 148. 2005 [2006].

Syn.: *Acacia adenopa* Hook. & Arn.; *Acacia caven* (Molina) Molina; *Acacia cavenia* Colla, nom. illeg.; *Acacia cavenia* (Molina) Hook. & Arn.; *Acacia farnesiana* (L.) Willd. forma *cavenia* (Molina) E.C.Clos; *Acacia farnesiana* (L.) Willd. var. *brachicarpa* Kuntze; *Acacia farnesiana* (L.) Willd. var. *cavenia* (Molina) Kuntze; *Acacia farnesiana* (L.) Willd. var. *cavenia* (Molina) Arechav., comb. superfl.; *Acacia farnesiana* (L.) Willd. var. *heterocarpa* Kuntze; *Mimosa caven* Molina; *Mimosa cavenia* Molina; *Vachellia farnesiana* (L.) Wight & Arn. forma *brachypoda* Speg.; *Vachellia farnesiana* (L.) Wight & Arn. forma *cavenia* (Molina) Speg.

Shrub or small tree.

Departmental distribution in Paraguay: Alto Paraguay, Caaguazú, Canindeyú, Central, Concepción, Cordillera, Guairá, Ñeembucú, Paraguarí, Presidente Hayes, San Pedro.

Voucher: *Cited in Vogt and Mereles 2005: 10* (as *Acacia caven*).

##### ►*Vigna longifolia* (Benth.) Verdc., Kew Bull. 24(3): 541. 1970.

Syn.: *Phaseolus longifolius* Benth.; *Phaseolus ovatus* Benth.; *Phaseolus productus* Ducke; *Phaseolus schottii* Benth.; *Phaseolus schottii* Benth. forma *ovatus* (Benth.) Hassl.; *Phaseolus schottii* Benth. var. *genuinus* Hassl., nom. illeg.; *Phaseolus schottii* Benth. var. *longifolius* (Benth.) Hassl.

Climber.

Departmental distribution in Paraguay: Alto Paraguay, Ñeembucú, Presidente Hayes.

Voucher: *M. Vera et al. 209* (BM, CTES, FCQ, MO, PY).

##### ►*Zornia multinervosa* Burkart ex Bacigalupo, Darwiniana 21(1): 174. 1977 [1978].

Herb.

Departmental distribution in Paraguay: Misiones, Ñeembucú.

Voucher: *J. De Egea et al. 638* (BM, FCQ).

##### ►*Zygia morongii* Barneby & J.W.Grimes, Mem. New York Bot. Gard. 74(2): 108. 1997.

Syn.: *Pithecellobium cauliflorum* auct. non (Willd.) Mart.; *Zygia cauliflora* auct. non (Willd.) Killip ex Record

Shrub.

Departmental distribution in Paraguay: Central, Ñeembucú, Presidente Hayes.

Voucher: *J. De Egea & M. Vera 473* (BM, CTES, FCQ, MO).

#### GESNERIACEAE

##### ►*Sinningia elatior* (Kunth) Chautems, Candollea 45: 383. 1990.

Syn.: *Corytholoma igneum* (Mart.) Fritsch; *Corytholoma sceptrum* (Mart.) Decne. var. *ignea* (Mart.) Molfino; *Corytholoma strictum* (Hook. & Arn.) Decne.; *Gesneria elatior* Kunth; *Gesneria sceptrum* Mart. var. *igneum* Mart.; *Gesneria stricta* Hook. & Arn.; *Rechsteineria stricta* (Hook. & Arn.) Kuntze; *Sinningia stricta* (Hook. & Arn.) Wiehler

Herb.

Departmental distribution in Paraguay: Alto Paraná, Amambay, Caaguazú, Caazapá, Canindeyú, Central, Concepción, Cordillera, Guairá, Itapúa, Misiones, Ñeembucú, Paraguarí, San Pedro.

Voucher: *J. De Egea et al. 702* (BM, FCQ).

##### *Sinningia tubiflora* (Hook.) Fritsch, Nat. Pflanzenfam. 4(3b): 182. 1894.

Syn.: *Achimenes tubiflora* (Hook.) Britton; *Dolichodeira tubiflora* (Hook.) Hanst; *Gesneria tubiflora* (Hook.) Griseb.; *Gloxinia tubiflora* Hook.

Herb.

Departmental distribution in Paraguay: Caazapá, Central, Ñeembucú, Presidente Hayes, San Pedro.

Voucher: *T. Meyer 15939* (LIL) (cited in Toursarkissian 1969: 44).

##### *Sinningia warmingii* (Hiern) Chautems, Candollea 45: 386. 1990.

Syn.: *Corytholoma sceptrum* (Mart.) Decne. var. *arenosa* Chodat & Hassl.; *Corytholoma warmingii* (Hiern) Toursark.; *Gesneria warmingii* Hiern; *Rechsteineria lindleyi* (Hook.) Fritsch. var. *macrophylla* Hoehne; *Rechsteineria multiflora* Fritsch; *Rechsteineria schlickmannii* Hoehne; *Rechsteineria warmingii* (Hiern) Hjelmq.

Herb.

Departmental distribution in Paraguay: Alto Paraguay, Amambay, Canindeyú, Central, Concepción, Cordillera, Ñeembucú, Presidente Hayes, San Pedro.

Voucher: *M. Peña-Chocarro et al. 2200* (FCQ).

#### HALORAGACEAE

##### ►*Myriophyllum aquaticum* (Vell.) Verdc., Kew Bull. 28: 36. 1973.

Syn.: *Enydria aquatica* Vell.; *Myriophyllum brasiliense* Cambess.; *Myriophyllum proserpinacoides* Gillies ex Hook. & Arn.

Aquatic herb.

Departmental distribution in Paraguay: Alto Paraguay, Central, Cordillera, Guairá, Ñeembucú.

Voucher: *S. Keel & L. Spinzi v- 137* (FCQ).

#### HYDROCHARITACEAE

##### *Egeria najas* Planch., Ann. Sci. Nat., Bot., sér. 3, 11: 80. 1849.

Syn.: *Anacharis najas* (Planch.) Vict.; *Elodea guianensis* Rich. forma *longifolia* Chodat & Hassl.; *Elodea kochii* Herter; *Elodea najas* (Planch.) Casp.; *Elodea paraguayensis* Herter, nom. illeg.

Aquatic herb.

Departmental distribution in Paraguay: Cordillera, Ñeembucú, San Pedro.

Voucher: Cited in Zuloaga et al. 2008: 421.

##### ►*Limnobium laevigatum* (Humb. & Bonpl. ex Willd.) Heine, Adansonia, n.s. 8(3): 315. 1968.

Syn.: *Hydrocharis stolonifera* (G.Mey.) Kuntze; *Hydromystria laevigata* (Humb. & Bonpl. ex Willd.) Hunz.; *Hydromystria laevigata* (Willd.) Díaz-Mir. & Philcox, comb. superfl.; *Hydromystria sinclairi* (Benth.) Hauman; *Hydromystria stolonifera* G.Mey.; *Limnobium sinclairi* Benth.; *Limnobium spongia* (Bosc) Steud. subsp. *laevigatum* (Humb. & Bonpl. ex Willd.) Lowden; *Limnobium stoloniferum* (G.Mey.) Griseb.; *Salvinia laevigata* Humb. & Bonpl. ex Willd.

Aquatic herb.

Departmental distribution in Paraguay: Ñeembucú, Presidente Hayes.

Voucher: *M.A. Walter 127* (BM).

#### HYDROLEACEAE

##### ►*Hydrolea elatior* Schott, Syst. Veg., ed. 16 [Sprengel] 4(2): 404. 1827.

Syn.: *Hydrolea albiflora* (Chodat & Hassl.) Brand; *Hydrolea albiflora* (Chodat & Hassl.) Brandvar. *depressa* Brand; *Hydrolea cryptantha* Brand; *Hydrolea cryptantha* Brand var. *meridionalis* Hassl.; *Hydrolea glabra* Schumach. & Thonn. forma *albiflora* Chodat & Hassl.; *Hydrolea glabra* Schumach. & Thonn. var. *spinosa* Chodat & Hassl.; *Hydrolea multiflora* Mart. ex Choisy

Herb.

Departmental distribution in Paraguay: Alto Paraguay, Amambay, Caaguazú, Ñeembucú, Presidente Hayes.

Voucher: *J. De Egea et al. 694* (BM, CTES, FCQ, G, MO, UNR).

##### ►*Hydrolea spinosa* L. var. *paraguayensis* (Chodat) L.J.Davenp., Rhodora 90: 184. 1988.

Syn.: *Hydrolea megapotamica* Spreng.; *Hydrolea mollis* Chodat; *Hydrolea paraguayensis* Chodat; *Hydrolea paraguayensis* Chodat forma *grandifolia* Chodat & Hassl.; *Hydrolea paraguayensis* Chodatvar. *inermis* Chodat & Hassl.; *Hydrolea spinosa* L. var. *megapotamica* (Spreng.) Brand; *Nama megapotamica* (Spreng.) Kuntze

Herb or subshrub.

Departmental distribution in Paraguay: Alto Paraguay, Caazapá, Central, Cordillera, Ñeembucú, Paraguarí.

Voucher: *M.A. Walter 25* (BM).

#### HYPOXIDACEAE

##### *Hypoxis decumbens* L., Syst. Nat., ed. 10, 2: 986. 1758.

Syn.: *Hypoxis decumbens* var. *major* Seub.

Herb.

Departmental distribution in Paraguay: Alto Paraná, Caazapá, Canindeyú, Central, Cordillera, Guairá, Misiones, Ñeembucú.

Voucher: *M. Peña-Chocarro et al. 1984* (FCQ).

#### IRIDACEAE

##### *Cipura paludosa Aubl. subsp. *boliviensis* Ravenna, Onira 1(5): 40. 1988.

Herb.

Departmental distribution in Paraguay: Alto Paraguay, Amambay, Caazapá, Concepción, Cordillera, Guairá, Ñeembucú, San Pedro.

Voucher: *R. Spichiger et al. 5204* (G) (cited in Ramella 2008: 279).

##### ►*Cypella armosa* Ravenna

Syn.: *Cypella gracilis* (Herb.) Klatt forma *humilis* Baker

Herb.

Departmental distribution in Paraguay: Alto Paraná, Caaguazú, Caazapá, Cordillera, Guairá, Ñeembucú, Paraguarí, Presidente Hayes, San Pedro.

Voucher: *F. Mereles 9733* (FCQ).

##### *Cypella exilis Ravenna, Nordic J. Bot. 1: 492. 1981.

Syn.: *Cypella gracilis* (Klatt) Baker, nom. illeg.; *Polia gracilis* Klatt

Herb.

Departmental distribution in Paraguay: Canindeyú, Central, Concepción, Ñeembucú.

Voucher: *R. Spichiger et al. 5283* (G) (cited in Ramella 2008: 280).

##### ►*Cypella herbertii* (Lindl.) Herb., Bot. Mag. 53: t. 2637. 1826.

Syn.: *Cypella bonariensis* (Ten.) Niederl.; *Moraea hebertii* Lindl.; *Polia bonariensis* Ten.; *Tigridia herbertii* (Lindl.) Herb.

Herb.

Departmental distribution in Paraguay: Ñeembucú, Presidente Hayes.

Voucher: *M. Peña-Chocarro et al. 1974* (BM, CTES, FCQ, MO).

##### *Herbertia lahue* (Molina) Goldblatt subsp. *amoena* (Griseb) Goldblatt, Ann. Missouri Bot. Gard. 64(2): 370. 1977 [1978].

Syn.: *Alophia amoena* (Griseb.) Kuntze; *Alophia lahue* (Molina) Espinosa subsp. *amoena* (Griseb.) Ravenna; *Calydorea furcata* (Klatt) Baker; *Herbertia amoena* Griseb.; *Nemastylis furcata* Klatt; *Trifurcia lahue* (Molina) Goldblatt subsp. *amoena* (Griseb.) Goldblatt

Herb.

Departmental distribution in Paraguay: Itapúa, Misiones, Ñeembucú.

Voucher: Cited in Zuloaga et al. 2008: 430.

##### *Neomarica sp.

Herb.

Voucher: *L. Bernardi 18482* (G) (cited in Ramella 2008: 282).

##### *Sisyrinchium capillare E.P.Bicknell, Bull. Torrey Bot. Club 26(12): 608. 1899.

Herb.

Departmental distribution in Paraguay: Ñeembucú.

Voucher: *R. Spichiger et al. 5218* (G) (http://www.ville-ge.ch/musinfo/bd/cjb/fdp/).

##### *Sisyrinchium chilense Hook., Bot. Mag. 54: t. 2786. 1827.

Syn.: *Sisyrinchium azureum* Phil.; *Sisyrinchium graminifolium* auct. non Lindl.; *Sisyrinchium iridifolium* auct. non Kunth; *Sisyrinchium iridifolium* subsp. *valdivianum* (Phil.) Ravenna; *Sisyrinchium ramosum* var. *chilense* (Hook.) Herb.; *Sisyrinchium scabrum* Cham. & Schltdl. var. *exaltatum* Klatt; *Sisyrinchium uniflorum* Gay ex Phil.

Herb.

Departmental distribution in Paraguay: Ñeembucú.

Voucher: *R. Spichiger et al. 5244* (G) (cited in Ramella 2008: 280).

##### ►*Sisyrinchium commutatum* Klatt, Hamburger Garten- Blumenzeitung 16: 164. 1860.

Syn.: *Sisyrinchium secundiflorum* Klatt

Herb.

Departmental distribution in Paraguay: Central, Cordillera, Itapúa, Misiones, Ñeembucú, Paraguarí.

Voucher: *M. Peña-Chocarro et al. 1987* (BM, CTES, FCQ).

##### *Sisyrinchium* sp.

Herb.

Voucher: *L. Bernardi 18430* (G) (cited in Ramella 2008: 265).

#### JUNCACEAE

##### ►*Juncus microcephalus* Kunth, Nov. Gen. Sp. (quarto ed.) 1: 237. 1815 [1816].

Syn.: *Juncus floribundus* Kunth; *Juncus floribundus* Phil., nom. illeg.; *Juncus luzuloxyphium* Griseb.; *Juncus microcephalus* Kunth forma *conglobatus* Barros; *Juncus microcephalus* Kunth var. *floribundus* (Kunth) Kunth; *Juncus microcephalus* Kunth var. *intermedius* Kunth; *Juncus microcephalus* Kunth var. *typicus* Buchenau, nom. illeg.; *Juncus microcephalus* Kunth var. *virens* Griseb.; *Juncus rudis* Kunth; *Juncus sellowianus* Kunth; *Juncus timotensis* Barros

Herb.

Departmental distribution in Paraguay: Caaguazú, Central, Guairá, Ñeembucú, Paraguarí, San Pedro.

Voucher: *J. De Egea et al. 650* (BM, CTES, FCQ, MO).

##### ►*Juncus tenuis* Willd., Sp. Pl., ed. 4 [Willdenow] 2: 214. 1799.

Syn.: *Juncus smithii* Kunth; *Juncus subtenuis* Herter; *Juncus tenuis* Willd. var. *multicornis* E.Mey.; *Juncus tenuis* Willd. var. *williamsii* Fernald

Herb.

Departmental distribution in Paraguay: Central, Ñeembucú.

Voucher: *J. De Egea et al. 806* (BM, CTES, FCQ, MO).

#### LAMIACEAE

##### *Hyptis lappacea Benth., Labiat. Gen. Spec. 103. 1833.

Syn.: *Hyptis cinerea* Morong; *Hyptis cinerea* Morongvar. *genuina* Briq., nom. illeg.; *Hyptis cinerea* Morongvar. *stenophylla* Briq.; *Hyptis globifera* auct. non G.Mey.; *Hyptis michelii* Briq. ex Micheli; *Hyptis trichoneura* Briq. ex Micheli; *Mesosphaerum cinereum* (Morong) Briq.; *Mesosphaerum lappaceum* (Benth.) Kuntze; *Mesosphaerum trichoneurum* (Briq. ex Micheli) Briq.

Herb.

Departmental distribution in Paraguay: Alto Paraná, Alto Paraguay, Caaguazú, Caazapá, Central, Concepción, Cordillera, Guairá, Ñeembucú, Paraguarí, Presidente Hayes.

Voucher: *M. Peña-Chocarro et al. 2319* (BM, CTES, FCQ, G, MO, PY, SI).

##### ►*Ocimum ovatum* Benth., Labiat. Gen. Spec. 13. 1832.

Syn.: *Ocimum balansae* Briq.; *Ocimum hassleri* Briq.; *Ocimum hassleri* Briq. var. *acutatum* Briq.; *Ocimum hassleri* Briq. var. *obtusifolium* Briq.; *Ocimum neurophyllum* Briq.; *Ocimum procurrens* Epling; *Ocimum tweedianum* Benth.

Herb.

Departmental distribution in Paraguay: Alto Paraná, Amambay, Canindeyú, Ñeembucú, San Pedro.

Voucher: *M. Peña-Chocarro et al. 2244* (BM, FCQ).

##### *Rhabdocaulon strictus* (Benth.) Epling, Repert. Spec. Nov. Regni Veg. 85: 137. 1936.

Syn.: *Cunila stricta* Benth.

Herb.

Departmental distribution in Paraguay: Ñeembucú.

Voucher: *T. Meyer 15999* (UC) (cited in Epling and Játiva 1966: 258).

##### *Salvia aridicola* Briq., Bull. Herb. Boissier sér. 2, 7: 606. 1907.

Syn.: *Salvia dumeticola* Briq.

Subshrub.

Departmental distribution in Paraguay: Amambay, Caaguazú, Canindeyú, Ñeembucú.

Voucher: *E. Hassler 5979* (G) (cited in Santos 1996: 39).

##### ►*Scutellaria racemosa* Pers., Syn. Pl. (Persoon) 2(1): 136. 1806.

Syn.: *Scutellaria bonariensis* Willd. ex Benth.; *Scutellaria hastata* Larrañaga; *Scutellaria heterophylla* Willd. ex Benth.; *Scutellaria rojasii* Briq.; *Scutellaria rumicifolia* Kunth

Herb.

Departmental distribution in Paraguay: Caaguazú, Canindeyú, Central, Concepción, Cordillera, Itapúa, Misiones, Ñeembucú, Paraguarí, Presidente Hayes.

Voucher: *M. Peña-Chocarro et al. 2256* (BM, CTES, FCQ, MO).

##### *Teucrium vesicarium Mill., Gard. Dict. (ed. 8) Teucrium no. 17. 1768.

Syn.: *Teucrium carthaginense* Lange; *Teucrium hirtum* Willd.; *Teucrium inflatum* Sw.; *Teucrium mollifolium* Larrañaga; *Teucrium palustre* Kunth; *Teucrium picardae* Krug & Urb.; *Teucrium tenuipes* Epling; *Teucrium vesicarium* Mill. var. *palustre* Epling

Herb.

Departmental distribution in Paraguay: Alto Paraná, Central, Cordillera, Guairá, Ñeembucú, Paraguarí, Presidente Hayes.

Voucher: *R. Spichiger et al. 5306* (CTES, G, MO) (http://www.ville-ge.ch/musinfo/bd/cjb/fdp/).

#### LAURACEAE

##### ►*Nectandra angustifolia* (Schrad.) Nees & Mart., Linnaea 8: 48. 1833.

Syn.: *Nectandra angustifolia* (Schrad.) Nees & Mart. var. *falcifolia* Nees; *Nectandra falcifolia* (Nees) J.A.Castigl. ex Mart.Crov. & Piccinini; *Nectandra megapotamica* (Spreng.) Mez; *Nectandra membranacea* (Spreng.) Hassl.; *Nectandra membranacea* (Spreng.) Hassl. var. *falcifolia* (Nees) Hassl.; *Nectandra saligna* Nees; *Ocotea angustifolia* Shrad.

Tree.

Departmental distribution in Paraguay: Alto Paraná, Canindeyú, Itapúa, Ñeembucú, Paraguarí.

Voucher: *S. Keel & L. Spinzi v-160* (FCQ).

##### ►*Ocotea acutifolia* (Nees) Mez, Jahrb. Königl. Bot. Gart. Berlin 5: 340. 1889.

Syn.: *Oreodaphne acutifolia* Nees; *Oreodaphne acutifolia* Nees var. *prolifera* Nees

Tree.

Departmental distribution in Paraguay: Amambay, Central, Itapúa, Ñeembucú, Paraguarí.

Voucher: *C. Vogt 150* (FCQ).

##### *Ocotea diospyrifolia (Meisn.) Mez, Jahrb. Königl. Bot. Gart. Berlin 5: 374. 1889.

Syn.: *Ocotea pseudocorymbosa* Hassl.; *Ocotea suaveolens* (Meisn.) Benth. & Hook. ex Hieron.; *Ocotea suaveolens* (Meisn.) Benth. & Hook. ex Hieron. var. *robusta* Hassl.; *Oreodaphne diospyrifolia* Meisn.; *Oreodaphne suaveolens* Meisn.; *Strychnodaphne suaveolens* Griseb.

Tree.

Departmental distribution in Paraguay: Alto Paraguay, Amambay, Caazapá, Canindeyú, Central, Cordillera, Ñeembucú, Paraguarí, Presidente Hayes, San Pedro.

Voucher: *M. Peña-Chocarro et al. 2368* (BM, CTES, FCQ, G, MO, SI, UNR).

##### *Ocotea spectabilis (Meisn.) Mez, Jahrb. Königl. Bot. Gart. Berlin 5: 372. 1889.

Syn.: *Oreodaphne spectabilis* Meisn.

Tree.

Departmental distribution in Paraguay: Alto Paraná, Central, Cordillera, Ñeembucú.

Voucher: *R. Spichiger et al. 5301* (G) (http://www.ville-ge.ch/musinfo/bd/cjb/fdp/).

#### LEMNACEAE

##### *Lemna aequinoctialis* Welw., Apont. 578. 1858 [1859].

Syn.: *Lemna angolensis* Hegelm.; *Lemna paucicostata* Hegelm.; *Lemna perpusilla* auct. non Torr.; *Lemna trinervis* Small

Aquatic herb.

Departmental distribution in Paraguay: Alto Paraguay, Caaguazú, Itapúa, Ñeembucú, Presidente Hayes, San Pedro.

Voucher: *M.A. Walter 44* (FLAS) (cited in Landolt 1986: 238).

##### *Spirodela intermedia* W.Koch, Ber. Schweiz. Bot. Ges. 41(1): 113. 1932.

Syn.: *Lemna polyrrhiza* L. var. *maxima* Griseb.; *Lemna biperforata* W.Koch; *Spirodela biperforata* W.Koch

Aquatic herb.

Departmental distribution in Paraguay: Alto Paraguay, Central, Cordillera, Ñeembucú.

Voucher: *M.A. Walter 43* (FLAS) (cited in Landolt 1986: 240).

#### LENTIBULARIACEAE

##### ►*Utricularia foliosa* L., Sp. Pl. 1: 14. 1753.

Syn.: *Utricularia oligosperma* A.St.-Hil.

Aquatic herb.

Departmental distribution in Paraguay: Alto Paraguay, Central, Concepción, Cordillera, Ñeembucú, Presidente Hayes.

Voucher: *J. De Egea et al. 687* (BM, CTES, FCQ, MO).

#### LYTHRACEAE

##### *Cuphea bonplandii* Lourteig, Lilloa 9: 346. 1943.

Herb.

Departmental distribution in Paraguay: Cordillera, Guairá, Misiones, Ñeembucú, Paraguarí.

Voucher: *A.G. Schulz 7710* (cited in Duré and Morelo 2010: 24).

##### *Cuphea carthagenensis* (Jacq.) J.F.Macbr., Publ. Field Columb. Mus., Bot. Ser. 8: 124. 1930.

Syn.: *Cuphea balsamona* Cham. & Schltdl.; *Lythrum carthagenense* Jacq.

Herb or subshrub.

Departmental distribution in Paraguay: Alto Paraná, Amambay, Caaguazú, Caazapá, Canindeyú, Central, Concepción, Cordillera, Guairá, Itapúa, Misiones, Ñeembucú, Paraguarí, Presidente Hayes, San Pedro.

Voucher: *M. Peña-Chocarro et al. 1970B* (BM, FCQ).

##### *Cuphea glutinosa* Cham. & Schltdl., Linnaea 2: 369. 1827.

Syn.: *Cuphea hyssopifolia* Kunth var. *brachyphylla* Griseb.; *Cuphea thymoides* Griseb., nom. illeg.

Herb or subshrub.

Departmental distribution in Paraguay: Alto Paraná, Caaguazú, Central, Cordillera, Guairá, Itapúa, Misiones, Ñeembucú, Paraguarí.

Voucher: *R. Duré et al. 721* (cited in Duré and Morelo 2010: 51).

##### *Cuphea lysimachioides* Cham. & Schltdl., Linnaea 2: 374. 1827.

Syn.: *Cuphea hassleri* Koehne; *Cuphea lysimachioides* Cham. & Schltdl. forma *brevipes* Koehne; *Cuphea lysimachioides* Cham. & Schltdl. var. *crassifolia* Chodat; *Cuphea lysimachioides* Cham. & Schltdl. var. *dubia* Koehne; *Cuphea lysimachioides* Cham. & Schltdl. var. *villosa* Koehne

Herb.

Departmental distribution in Paraguay: Alto Paraná, Alto Paraguay, Amambay, Caaguazú, Caazapá, Canindeyú, Central, Concepción, Cordillera, Guairá, Itapúa, Misiones, Ñeembucú, Paraguarí, Presidente Hayes, San Pedro.

Voucher: *J. De Egea et al. 637* (BM, CTES, FCQ, G, MO).

##### *Cuphea racemosa* (L.f.) Spreng. var. *palustris* Lourteig, Lilloa 9: 342. 1943.

Herb.

Departmental distribution in Paraguay: Alto Paraná, Caaguazú, Caazapá, Canindeyú, Central, Cordillera, Guairá, Itapúa, Misiones, Ñeembucú, Paraguarí, San Pedro.

Voucher: *A.G. Schulz 7664* (cited in Duré and Morelo 2010: 90).

##### *Cuphea racemosa* (L.f.) Spreng. var. *racemosa*, Syst. Veg. (ed. 16) [Sprengel] 2: 455. 1825.

Herb or subshrub.

Departmental distribution in Paraguay: Alto Paraná, Caaguazú, Caazapá, Canindeyú, Central, Cordillera, Guairá, Itapúa, Misiones, Ñeembucú, Paraguarí, San Pedro.

Voucher: *M. Peña-Chocarro et al. 2248A* (BM, FCQ).

##### *Heimia salicifolia* (Kunth) Link, Enum. Hort. Berol. Alt. 2: 3. 1822.

Syn.: *Nesaea salicifolia* Kunth

Shrub or subshrub.

Departmental distribution in Paraguay: Alto Paraná, Alto Paraguay, Amambay, Caaguazú, Caazapá, Canindeyú, Central, Concepción, Cordillera, Guairá, Itapúa, Misiones, Ñeembucú, Paraguarí, Presidente Hayes.

Voucher: *J. De Egea et al. 681* (BM, CTES, FCQ, MO).

##### *Pleurophora saccocarpa* Koehne, Bot. Jahrb. Syst. 2: 426. 1882.

Syn.: *Pleurophora annulosa* Koehne; *Pleurophora saccocarpa* Koehne var. *fiebrigii* Koehne; *Pleurophora saccocarpa* Koehne var. *glabrescens* Koehne; *Pleurophora saccocarpa* Koehne var. *hirtella* Koehne; *Pleurophora saccocarpa* Koehne var. *velutina* Koehne

Shrub.

Departmental distribution in Paraguay: Alto Paraná, Alto Paraguay, Amambay, Canindeyú, Central, Concepción, Misiones, Ñeembucú, Paraguarí, Presidente Hayes.

Voucher: *M. Peña-Chocarro et al. 1986* (BM, CTES, FCQ, G, MO, PY, SI).

#### MALPIGHIACEAE

##### *Galphimia australis* Chodat, Arch. Sci. Phys. Nat. 24: 500. 1890.

Syn.: *Galphimia australis* Chodat forma *angustifolia* Chodat; *Galphimia brasiliensis* (L.) A.Juss. forma *angustifolia* (Chodat) Nied.; *Galphimia brasiliensis* (L.) A.Juss. var. *australis* (Chodat) Chodat; *Galphimia brasiliensis* (L.) A.Juss. var. *pubescens* A.Juss.

Subshrub.

Departmental distribution in Paraguay: Alto Paraná, Amambay, Caaguazú, Canindeyú, Concepción, Cordillera, Guairá, Itapúa, Misiones, Ñeembucú, Paraguarí, Presidente Hayes.

Voucher: *E. Purvey 390* (CTES, MO) (cited in Anderson 2007: 51).

##### ►*Heteropterys glabra* Hook. & Arn., Bot. Misc. 3: 57. 1833.

Syn.: *Banisteria tenuis* Lindl.; *Heteropterys angustifolia* Griseb.; *Heteropterys angustifolia* Griseb. forma *angustissima* Nied.; *Heteropterys angustifolia* Griseb. forma *lanceolata* Nied.; *Heteropterys lanceolata* (Nied.) Herter; *Heteropterys pseudoangustifolia* Chodat

Shrub.

Departmental distribution in Paraguay: Alto Paraná, Alto Paraguay, Amambay, Central, Concepción, Cordillera, Guairá, Misiones, Ñeembucú, Paraguarí, Presidente Hayes, San Pedro.

Voucher: *M. Peña-Chocarro et al. 2356* (BM, CTES, FCQ, MO).

##### ►*Heteropterys umbellata* A.Juss., Fl. Bras. Merid. (quarto ed.) 3: 25. 1832.

Subshrub.

Departmental distribution in Paraguay: Alto Paraguay, Concepción, Ñeembucú.

Voucher: *L. Bernardi 18427* (BM).

##### ►*Janusia guaranitica* (A.St.-Hil.) A.Juss., Ann. Sci. Nat., Bot., sér. 2, 13: 251. 1840.

Syn.: *Aspicarpa guaranitica* (A.St.-Hil.) Hassl.; *Gaudichaudia barbeyi* (Chodat) Chodat; *Gaudichaudia guaranitica* A.St.-Hil.; *Janusia barbeyi* Chodat; *Janusia guaranitica* (A.St.-Hil.) A.Juss. forma *cordifolia* Nied., nom. superfl.; *Janusia guaranitica* (A.St.-Hil.) A.Juss. forma *glabrata* Nied.; *Janusia guaranitica* (A.St.-Hil.) A.Juss. var. *barbeyi* (Chodat) Kuntze; *Janusia prolixa* Arechav.

Climber.

Departmental distribution in Paraguay: Alto Paraguay, Amambay, Caaguazú, Canindeyú, Central, Concepción, Cordillera, Guairá, Ñeembucú, Paraguarí, Presidente Hayes, San Pedro.

Voucher: *M. Peña-Chocarro et al. 2237* (BM, FCQ).

##### *Stigmaphyllon bonariense* (Hook. & Arn.) C.E.Anderson, Brittonia 48(4): 543. 1996.

Syn.: *Banisteria bonariensis* Hook. & Arn.; *Stigmaphyllon heterophyllum* Hook.; *Stigmaphyllon littorale* A.Juss.; *Stigmaphyllon littorale* A.Juss. var. *trilobum* Nied.

Climber.

Departmental distribution in Paraguay: Alto Paraná, Alto Paraguay, Itapúa, Misiones, Ñeembucú.

Voucher: *T. Meyer 15893* (CTES) (cited in Anderson 1997: 238).

#### MALVACEAE

##### ►*Abutilon pauciflorum* A.St.-Hil., Fl. Bras. Merid. (quarto ed.) 1: 206. 1827.

Syn.: *Abutilon melanocarpum* A.St.-Hil. & Naudin; *Abutilon mollissimum* auct. non (Cav.) Sweet; *Abutilon pauciflorum* A.St.-Hil. forma *longe-corniculatum* Hassl.; *Abutilon pauciflorum* A.St.-Hil. var. *cano-tomentosum* Hassl.; *Abutilon pedunculare* auct. non Humb.; Bonpl. & Kunth; *Abutilon rugosulum* Hochr. ex Chodat & Hassl.

Subshrub.

Departmental distribution in Paraguay: Central, Cordillera, Guairá, Ñeembucú, Paraguarí, Presidente Hayes.

Voucher: *M. Peña-Chocarro et al. 2236* (BM, FCQ).

##### *Ayenia aprica* Cristóbal, Opera Lilloana 4: 126. 1960.

Subshrub.

Departmental distribution in Paraguay: Amambay, Caaguazú, Cordillera, Guairá, Itapúa, Ñeembucú, Paraguarí.

Voucher: *U.G. Eskuche 2533-15* (CTES) (cited in Cristóbal 2007: 83).

##### *Byttneria rhamnifolia* Benth., J. Bot. (Hooker) 3: 164. 1851.

Syn.: *Byttneria campestris* S.Moore; *Byttneria charagmocarpa* S.Moore

Shrub.

Departmental distribution in Paraguay: Ñeembucú.

Voucher: *T. Meyer 16145* (LIL) (cited in Cristóbal 2007: 54).

##### *Byttneria scabra* L., Syst. Nat.,ed. 10, 2: 939. 1759.

Syn.: *Byttneria scabra* L. var. *dentata* A.St.-Hil.; *Byttneria scabra* L. var. *serrata* K.Schum.

Shrub.

Departmental distribution in Paraguay: Alto Paraná, Alto Paraguay, Amambay, Caaguazú, Canindeyú, Central, Concepción, Cordillera, Guairá, Misiones, Ñeembucú, Paraguarí, San Pedro.

Voucher: *A.G. Schulz 7796* (CTES) (cited in Cristóbal 2007: 30).

##### ►*Cienfuegosia drummondii* (A.Gray) Lewton, Bull. Torrey Bot. Club 37(9): 475. 1910.

Syn.: *Cienfuegosia sulphurea* (A.St.-Hil.) Garcke forma *intermedia* Chodat & Hassl.; *Cienfuegosia sulphurea* (A.St.-Hil.) Garcke var. *drummondii* (A.Gray) Hochr.; *Cienfuegosia sulphurea* (A.St.-Hil.) Garcke var. *glabra* Gürke; *Cienfuegosia sulphurea* (A.St.-Hil.) Garcke var. *major* Hassl.; *Fuegosia drummondii* A.Gray; *Fuegosia pulverulenta* (Griseb.) Hochr.; *Fuegosia sulphurea* A.St.-Hil. var. *trifida* Griseb. ex Rodrigo, nom. nud.; *Hibiscus drummondii* (A.Gray) Kuntze; *Hibiscus pulverulentus* Griseb.

Herb.

Departmental distribution in Paraguay: Alto Paraguay, Central, Cordillera, Guairá, Ñeembucú, Paraguarí, Presidente Hayes.

Voucher: *J. De Egea & R. Elsam 811* (BM, CTES, FCQ).

##### *Cienfuegosia sulphurea* (A.St.-Hil.) Garcke, Bonplandia 8: 150. 1860.

Syn.: *Cienfuegosia drummondii* (A.Gray) Lewton var. *pubescens* Hassl., nom. nud.; *Cienfuegosia sulphurea* (A.St.-Hil.) Garcke var. *genuina* Gürke, nom. illeg.; *Cienfuegosia sulphurea* (A.St.-Hil.) Garcke var. *trifida* Griseb. ex Seckt, nom. nud.; *Fugosia sulphurea* A.St.-Hil.; *Hibiscus jussieui* Kuntze

Herb.

Departmental distribution in Paraguay: Concepción, Ñeembucú.

Voucher: *A.G. Schulz 7978* (CTES, LIL) (cited in Krapovickas 2003a: 29).

##### ►*Cienfuegosia ulmifolia* Fryxell, Brittonia 19(1): 35. 1967.

Herb.

Departmental distribution in Paraguay: Alto Paraguay, Concepción, Ñeembucú, Presidente Hayes.

Voucher: *C. Vogt & A. Contreras 711* (CTES, FACEN, FCQ).

##### *Corchorus hirtus* L., Sp. Pl., ed. 2, 1: 747. 1762.

Syn.: *Corchorus hirtus* L. var. *cuyabensis* K.Schum.; *Corchorus hirtus* L. var. *orinocensis* (Kunth) K.Schum.; *Corchorus orinocensis* Kunth; *Corchorus pilolobus* Link

Herb or subshrub.

Departmental distribution in Paraguay: Alto Paraná, Alto Paraguay, Amambay, Canindeyú, Central, Concepción, Cordillera, Ñeembucú, Presidente Hayes, San Pedro.

Voucher: Cited in Zuloaga et al. 2008: 3072.

##### *Guazuma ulmifolia* Lam., Encycl. [Lamarck] 3: 52. 1789.

Syn.: *Guazuma tomentosa* Kunth; *Guazuma ulmifolia* Lam. var. *tomentosa* (Kunth) K. Schum.

Tree.

Departmental distribution in Paraguay: Alto Paraguay, Amambay, Central, Concepción, Cordillera, Guairá, Ñeembucú, Paraguarí, San Pedro.

Voucher: *A.G. Schulz 7818* (CTES, LIL) (cited in Cristóbal 2007: 20).

##### *Luehea divaricata Mart., Nov. Gen. Sp. Pl. (Martius) 1: 101. 1826.

Syn.: *Luehea paniculata* auct. non Mart.; *Thespesia brasiliensis* Spreng.

Tree.

Departmental distribution in Paraguay: Alto Paraná, Amambay, Caaguazú, Caazapá, Canindeyú, Central, Concepción, Guairá, Itapúa, Ñeembucú, Paraguarí, San Pedro.

Voucher: *C. Vogt 173* (CTES, FCQ).

##### *Melochia arenosa* Benth., J. Bot. (Hooker) 4: 127. 1842.

Syn.: *Melochia cinerea* A.St.-Hil. & Naudin var. *discolor* Hassl., nom. nud.

Subshrub.

Departmental distribution in Paraguay: Alto Paraguay, Concepción, Cordillera, Ñeembucú.

Voucher: *T. Rojas 12680* (CTES, LIL) (cited in Cristóbal 2007: 118).

##### ►*Melochia canescens* Cristóbal, Bonplandia (Corrientes) 9(1-12): 43, 46. 1996.

Subshrub.

Departmental distribution in Paraguay: Alto Paraguay, Ñeembucú.

Voucher: *J. De Egea & R. Elsam 815* (FCQ).

##### *Melochia hermannioides* A.St.-Hil., Fl. Bras. Merid. (quarto ed.) 1: 163. 1825.

Syn.: *Melochia hermannioides* A.St.-Hil. forma *heterophylla* Hassl.; *Melochia hermannioides* A.St.-Hil. var. *lacinulata* (K.Schum. & Hassl.) Hassl.; *Melochia hermannioides* A.St.-Hil. var. *lanceolata* Hassl.; *Melochia lacinulata* K.Schum. & Hassl.; *Melochia parvifolia* Kunth forma *roseiflora* K.Schum. & Hassl.

Herb.

Departmental distribution in Paraguay: Caaguazú, Canindeyú, Central, Concepción, Cordillera, Guairá, Misiones, Ñeembucú, Paraguarí, Presidente Hayes, San Pedro.

Voucher: *J. De Egea et al. 737* (FCQ).

##### *Melochia pilosa* (Mill.) Fawc. & Rendle, Fl. Jam. 5: 164. 1926.

Syn.: *Melochia ulmarioides* A.St.-Hil.; *Melochia venosa* Sw.; *Sida pilosa* Mill.

Subshrub.

Departmental distribution in Paraguay: Alto Paraná, Alto Paraguay, Amambay, Caaguazú, Caazapá, Canindeyú, Concepción, Cordillera, Guairá, Itapúa, Misiones, Ñeembucú, Paraguarí, San Pedro.

Voucher: *R.H. Chodat s.n.* (G) (cited in Cristóbal 2007: 130).

##### *Melochia pyramidata* L. var. *hieronymi* K.Schum., Fl. Bras. (Martius) 12(3): 35. 1886.

Syn.: *Melochia pyramidata* L. forma *intermedia* Hassl.; *Melochia pyramidata* L. forma *transitoria* K.Schum. & Hassl.; *Melochia pyramidata* L. var. *pseudotomentosa* Hassl.; *Melochia tomentosa* L. var. *mattogrossensis* R.E.Fr.

Subshrub.

Departmental distribution in Paraguay: Alto Paraguay, Caaguazú, Caazapá, Canindeyú, Central, Concepción, Cordillera, Guairá, Misiones, Ñeembucú, Paraguarí, Presidente Hayes, San Pedro.

Voucher: *A.G. Schulz 7920* (CTES, G, LIL, MO) (cited in Cristóbal 2007: 104).

##### ►*Modiolastrum malvifolium* (Griseb.) K.Schum., Fl. Bras. (Martius) 12(3): 277. 1886.

Syn.: *Malvastrum modioliforme* (Kuntze) K.Schum.; *Malvastrum tweediei* Baker f.; *Malveopsis modioliformis* Kuntze; *Modiola malvifolia* Griseb.

Herb.

Departmental distribution in Paraguay: Alto Paraguay, Central, Itapúa, Ñeembucú, Paraguarí, Presidente Hayes.

Voucher: *M. Peña-Chocarro et al. 2246* (BM, FCQ).

##### *Pavonia hastata* Cav., Diss. 3: 138. 1787.

Syn.: *Greevesia cleisocalyx* F.Muell.; *Lassa hastata* (Cav.) Kuntze; *Pavonia hastata* Cav. forma *brevifolia* Gürke; *Pavonia hastata* Cav. var. *pubescens* Gürke; *Sida bonariensis* Willd. ex Spreng.

Subshrub.

Departmental distribution in Paraguay: Caaguazú, Caazapá, Canindeyú, Cordillera, Guairá, Misiones, Ñeembucú, Paraguarí, San Pedro.

Voucher: *A.G. Schulz 7797* (CTES) (cited in Fryxell 1999: 118).

##### ►*Pavonia hieronymi* Gürke, Fl. Bras. (Martius) 12(3): 209. 1892.

Syn.: *Asterochlaena hieronymi* (Gürke) Hassl.; *Asterochlaena hieronymi* (Gürke) Hassl. forma *angustiphylla* Hassl.; *Asterochlaena hieronymi* (Gürke) Hassl. subsp. *brevipila* Hassl.; *Asterochlaena hieronymi* (Gürke) Hassl. var. *grandiflora* (Chodat & Hassl.) Hassl.; *Asterochlaena hieronymi* (Gürke) Hassl.var. *montana* Hassl.; *Asterochlaena hieronymi* (Gürke) Hassl.var. *pseudoapiculata* Hassl.; *Asterochlaena morongii* (S.Moore) Hassl.; *Lassa hieronymi* (Gürke) Kuntze; *Pavonia geminiflora* Moric var. *grandiflora* Chodat & Hassl.; *Pavonia hieronymi* Gürke subsp. *tomentella* R.E.Fr.; *Pavonia morongii* S.Moore; *Pavonia sidifolia* Kunth forma *morongii* (S.Moore) Hassl.

Subshrub.

Departmental distribution in Paraguay: Alto Paraguay, Central, Concepción, Cordillera, Ñeembucú, Presidente Hayes, San Pedro.

Voucher: *J. De Egea et al. 632* (BM, FCQ, MO).

##### *Pavonia patuliloba* Hochr., Bull. Herb. Boissier, sér. 2, 5: 297. 1905.

Syn.: *Pavonia subhastata* Triana & Planch. forma *patuliloba* (Hochr.) Hassl.; *Pavonia subhastata* Triana & Planch. forma *quinqueloba* Hassl.; *Pavonia subhastata* Triana & Planch. subsp. *paludosa* Hassl.

Shrub.

Departmental distribution in Paraguay: Alto Paraguay, Central, Concepción, Ñeembucú.

Voucher: *T. Meyer 16114* (CTES) (cited in Fryxell 1999: 27).

##### *Pavonia vitifolia* Hochr., Bull. Herb. Boissier, sér. 2, 5: 297. 1905.

Shrub.

Departmental distribution in Paraguay: Alto Paraguay, Amambay, Caaguazú, Concepción, Guairá, Ñeembucú, Paraguarí, San Pedro.

Voucher: *A.G. Schulz 7908* (CTES, MO) (cited in Fryxell 1999: 31).

##### *Sida anomala A.St.-Hil., Fl. Bras. Merid. (quarto ed.) 1: 140. 1827.

Syn.: *Sida ciliaris* L. var. *anomala* (A.St.-Hil.) K.Schum.; *Sida ciliaris* L. forma *rubra* Kuntze

Herb.

Departmental distribution in Paraguay: Alto Paraguay, Caaguazú, Central, Cordillera, Guairá, Ñeembucú, Presidente Hayes, San Pedro.

Voucher: *T. Meyer 16204* (LIL) (cited in Krapovickas 2007: 222).

##### *Sida rhombifolia L., Sp. Pl. 2: 684. 1753.

Syn.: *Sida rhombifolia* L. var. *canariensis* (Willd.) Griseb.; *Sida rhombifolia* L. var. *rhomboidea* (Roxb.) Mast.; *Sida rhomboidea* Roxb.; *Sida subrhombiformis* Larrañaga

Herb or subshrub.

Departmental distribution in Paraguay: Alto Paraguay, Cordillera, Ñeembucú, Presidente Hayes.

Voucher: *L. Bernardi 18520* (MO) (http://www.tropicos.org/).

##### *Sida rodrigoi* Monteiro, Anais Congr. Soc. Bot. Brasil 15: 64. 1967.

Shrub.

Departmental distribution in Paraguay: Caaguazú, Caazapá, Central, Cordillera, Guairá, Ñeembucú, Paraguarí, Presidente Hayes.

Voucher: *A.G. Schulz 7869* (CTES) (cited in Krapovickas 2003b: 110).

##### *Sida urens* L., Syst. Nat, ed. 10, 2: 1145. 1759.

Herb.

Departmental distribution in Paraguay: Alto Paraná, Alto Paraguay, Amambay, Caaguazú, Caazapá, Central, Concepción, Cordillera, Guairá, Itapúa, Misiones, Ñeembucú, Paraguarí, Presidente Hayes.

Voucher: *A.G. Schulz 7804* (CTES) (cited in Krapovickas 2007: 37).

##### ►*Sidastrum paniculatum* (L.) Fryxell, Brittonia 30(4): 453. 1978.

Syn.: *Sida paniculata* L.

Subshrub.

Departmental distribution in Paraguay: Alto Paraguay, Caaguazú, Canindeyú, Central, Cordillera, Guairá, Misiones, Ñeembucú, Paraguarí, Presidente Hayes, San Pedro.

Voucher: *M. Peña-Chocarro et al. 2309* (BM, CTES, FCQ).

##### *Triumfetta semitriloba* Jacq., Enum. Syst. Pl. 22. 1760.

Syn.: *Triumfetta abutiloides* A.St.-Hil., A.Juss. & Cambess.; *Triumfetta tricuspis* A.St.-Hil.

Shrub.

Departmental distribution in Paraguay: Alto Paraná, Amambay, Caaguazú, Caazapá, Canindeyú, Central, Concepción, Cordillera, Guairá, Itapúa, Misiones, Ñeembucú, Paraguarí.

Voucher: Cited in Zuloaga et al. 2008: 3074.

##### *Waltheria carmensarae* J.G.Saunders, Darwiniana 43: 202. 2005.

Subshrub.

Departmental distribution in Paraguay: Itapúa, Ñeembucú.

Voucher: *A.G. Schulz 7700* (CTES, LIL, TEX) (cited in Saunders 2007: 166).

##### *Waltheria communis* A.St.-Hil., Fl. Bras. Merid. (quarto ed.) 1: 155. 1825.

Syn.: *Waltheria communis* A.St.-Hil. var. *tomentella* auct. non K. Schum.; *Waltheria douradinha* A.St.-Hil.

Herb.

Departmental distribution in Paraguay: Alto Paraná, Alto Paraguay, Amambay, Caaguazú, Caazapá, Canindeyú, Central, Concepción, Cordillera, Guairá, Itapúa, Misiones, Ñeembucú, Paraguarí, Presidente Hayes, San Pedro.

Voucher: *U.G. Eskuche 2533-11* (CTES, SI) (cited in Saunders 2007: 154).

#### MARANTACEAE

##### ►*Maranta sobolifera* L.Andersson, Nordic. J. Bot. 6(6): 742. 1986.

Herb.

Departmental distribution in Paraguay: Amambay, Caaguazú, Caazapá, Canindeyú, Central, Cordillera, Guairá, Ñeembucú, Paraguarí.

Voucher: *M. Vera et al. 224* (BM, CTES, FCQ).

##### ►*Thalia geniculata* L., Sp. Pl. 2: 566. 1753.

Syn.: *Maranta geniculata* (L.) Lam.

Herb. In swamps.

Departmental distribution in Paraguay: Alto Paraguay, Central, Concepción, Ñeembucú, Paraguarí, Presidente Hayes.

Voucher: *M. Peña-Chocarro et al. 2321* (BM, FCQ).

#### MAYACACEAE

##### ►*Mayaca fluviatilis* Aubl., Hist. Pl. Guiane 1: 42. 1775.

Syn.: *Mayaca aubletii* Michx.; *Mayaca caroliniana* Gand.; *Mayaca vandellii* Schott & Endl.

Aquatic herb.

Departmental distribution in Paraguay: Caaguazú, Canindeyú, Concepción, Guairá, Misiones, Ñeembucú, Paraguarí.

Voucher: *M.A. Walter 89* (BM).

#### MELIACEAE

##### *Guarea macrophylla Vahl subsp. *spicaeflora* (A.Juss.) T.D.Penn., Fl. Neotrop. 28: 287. 1981.

Syn.: *Guarea angustifolia* C.DC.; *Guarea balansae* C.DC.; *Guarea diversifolia* C.DC.; *Guarea dumetorum* C.DC.; *Guarea fiebrigii* C.DC.; *Guarea frutescens* C.DC.; *Guarea hassleri* C.DC.; *Guarea hassleri* C.DC. var. *esulcata* C.DC; *Guarea leucantha* C.DC.; *Guarea nemorensis* C.DC.; *Guarea parvifoliola* C.DC.; *Guarea ripicola* C.DC.; *Guarea silvicola* C.DC.; *Guarea spiciflora* A.Juss.; *Guarea subnudipetala* C.DC.

Tree.

Departmental distribution in Paraguay: Amambay, Caaguazú, Caazapá, Canindeyú, Central, Concepción, Cordillera, Ñeembucú, Paraguarí, San Pedro.

Voucher: *R. Spichiger et al. 5304* (G) (http://www.ville-ge.ch/musinfo/bd/cjb/fdp/).

##### ►*Trichilia catigua* A.Juss., Fl. Bras. Merid. (quarto ed.) 2: 77. 1829.

Syn.: *Trichilia affinis* A.Juss.; *Trichilia catigua* A.Juss. var. *longifolia* C.DC.; *Trichilia catigua* A.Juss. var. *pallens* C.DC.; *Trichilia polyclada* C.DC.

Tree.

Departmental distribution in Paraguay: Alto Paraná, Alto Paraguay, Amambay, Caaguazú, Caazapá, Canindeyú, Central, Concepción, Cordillera, Guairá, Ñeembucú, Paraguarí, Presidente Hayes, San Pedro.

Voucher: *M. Vera et al. 228* (BM, FCQ).

##### *Trichilia elegans* A.Juss., Fl. Bras. Merid. (quarto ed.) 2: 79. 1829.

Syn.: *Trichilia elegans* A.Juss. var. *latifoliola* C.DC.; *Trichilia graciliflora* Harms; *Trichilia hassleri* C.DC.; *Trichilia hirsuta* C.DC.; *Trichilia warmingii* C.DC.; *Trichilia warmingii* C.DC. var. *macrophylla* C.DC.

Tree.

Departmental distribution in Paraguay: Alto Paraná, Amambay, Caaguazú, Caazapá, Canindeyú, Central, Concepción, Cordillera, Guairá, Ñeembucú, Paraguarí, Presidente Hayes, San Pedro.

Voucher: *J. De Egea et al. 734B* (BM, CTES, FCQ, G, MO, PY, SI, UNR).

#### MENISPERMACEAE

##### ►*Cissampelos pareira* L., Sp. Pl. 2: 1031. 1753.

Syn.: *Cissampelos auriculata* Miers; *Cissampelos australis* A.St.-Hil.; *Cissampelos hederacea* Miers; *Cissampelos litoralis* A.St.-Hil.; *Cissampelos monoica* A.St.-Hil.; *Cissampelos pareira* L. forma *emarginato-mucronata* Chodat & Hassl.; *Cissampelos pareira* L. forma *reniformis* Chodat & Hassl.; *Cissampelos pareira* L. var. *australis* (A.St.-Hil.) Diels; *Cissampelos pareira* L. var. *caapeba* Eichler; *Cissampelos pareira* L. var. *gardneri* Diels; *Cissampelos pareira* L. var. *momoica* (A.St.-Hil.) Eichler; *Cissampelos pareira* L. var. *tamoides* (Willd.) Diels

Climber.

Departmental distribution in Paraguay: Alto Paraguay, Amambay, Caaguazú, Caazapá, Central, Concepción, Cordillera, Guairá, Ñeembucú, Paraguarí, Presidente Hayes, San Pedro.

Voucher: *M. Peña-Chocarro et al. 2222* (BM, FCQ).

##### ►*Hyperbaena hassleri* Diels, Pflanzenr. (Engler) [Heft 46] 4, Fam. 94: 201. 1910.

Syn.: *Abuta parvifolia* Rusby ex Moldenke, nom. nud.; *Hyperbaena ovalifolia* Chodat & Hassl. ex Moldenke, nom. nud.

Climber.

Departmental distribution in Paraguay: Central, Concepción, Guairá, Ñeembucú.

Voucher: *M. Peña-Chocarro et al. 2211A* (BM, CTES, FCQ).

#### MENYANTHACEAE

##### ►*Nymphoides indica* (L.) Kuntze, Revis. Gen. Pl. 2:429. 1891.

Syn.: *Limnanthemum humboldtianum* (Kunth) Griseb.; *Menyanthes indica* L.; *Nymphoides humboldtiana* (Kunth) Kuntze; *Villarsia humboltiana* Kunth

Aquatic herb.

Departmental distribution in Paraguay: Amambay, Central, Cordillera, Guairá, Ñeembucú, Paraguarí, Presidente Hayes.

Voucher: *C. Vogt 166* (CTES, FCQ).

#### MORACEAE

##### ►*Dorstenia brasiliensis* Lam., Encycl. (Lamarck) 2(1): 317. 1786.

Syn.: *Dorstenia brasiliensis* Lam. forma *balansae* Chodat; *Dorstenia brasiliensis* Lam. var. *guaranitica* Chodat & Vischer; *Dorstenia brasiliensis* Lam. var. *major* Chodat & Hassl.; *Dorstenia brasiliensis* Lam. var. *palustris* Hassl.; *Dorstenia brasiliensis* Lam. var. *tomentosa* (Fisch. & C.A.Mey.) Hassl.; *Dorstenia brasiliensis* Lam. var. *tubicina* (Ruiz & Pav.) Chodat & Vischer; *Dorstenia brasiliensis* Lam. var. *typica* Hassl., nom. illeg.; *Dorstenia montevidensis* Field & Gardner; *Dorstenia schulzii* Carauta, C. Valente & Dunn de Araujo; *Dorstenia tomentosa* Fisch. & C.A.Mey.; *Dorstenia tubicina* Ruiz & Pav.; *Dorstenia tubicina* Ruiz & Pav. forma *major* Hassl.; *Dorstenia tubicina* Ruiz & Pav. forma *subexcentrica* Hassl.; *Dorstenia tubicina* Ruiz & Pav. forma *typica* Hassl., nom. illeg.; *Dorstenia tubicina* Ruiz & Pav. var. *genuina* Hassl., nom. illeg.; *Dorstenia tubicina* Ruiz & Pav. var. *opifera* (Mart.) Hassl.

Herb.

Departmental distribution in Paraguay: Alto Paraguay, Amambay, Caaguazú, Central, Cordillera, Guairá, Ñeembucú, San Pedro.

Voucher: *F. Mereles 9722* (FCQ).

##### *Ficus adhatodifolia Schott ex Spreng., Syst. Veg., ed. 16 [Sprengel] 4(App.): 409. 1827.

Syn.: *Ficus anthelmintica* Mart.; *Ficus anthelmintica* Mart. var. *missionum* Hauman; *Ficus guapoi* D.Parodi; *Ficus morongii* Hassl.; *Pharmacosycea adhatodifolia* (Schott) Miq.; *Pharmacosycea anthelminthica* (Mart.) Miq.

Tree.

Departmental distribution in Paraguay: Alto Paraguay, Amambay, Caazapá, Central, Concepción, Cordillera, Guairá, Itapúa, Ñeembucú, Paraguarí, San Pedro.

Voucher: *R. Spichiger et al. 5351* (G) (http://www.ville-ge.ch/musinfo/bd/cjb/fdp/).

##### ►*Ficus luschnathiana* (Miq.) Miq., Ann. Mus. Bot. Lugduno-Batavi 3: 298. 1867.

Syn.: *Ficus diabolica* Herter; *Ficus diabolica* Herter forma *laurina* Herter; *Ficus diabolica* Herter forma *major* Herter; *Ficus diabolica* Herter forma *minor* Herter; *Ficus horquetensis* Chodat & Vischer; *Ficus ibapohi* Orb. ex Rojas Acosta; *Ficus monckii* Hassl.; *Ficus monckii* Hassl. forma *subcuneata* Hassl.; *Ficus monckii* Hassl. var. *sanmartiniana* Parodi; *Ficus speciosa* Rojas Acosta; *Urostigma luschnathianum* Miq.

Tree.

Departmental distribution in Paraguay: Alto Paraná, Amambay, Caazapá, Canindeyú, Central, Cordillera, Guairá, Itapúa, Ñeembucú, Paraguarí, Presidente Hayes.

Voucher: *J. De Egea & M. Vera 475* (BM, CTES, FCQ, G, MO, UNR).

##### *Maclura tinctoria* (L.) Steud. subsp. *mora* (Griseb.) Vázq.Avila, Darwiniana 26: 323. 1985.

Syn.: *Chlorophora mora* (Griseb.) Lillo; *Chlorophora reticulata* Herzog; *Chlorophora tinctoria* (L.) Gaudich. subsp. *mora* (Griseb.) Hassl.; *Chlorophora tinctoria* (L.) Gaudich. var. *mora* (Griseb.) Lillo; *Ioxylon mora* (Griseb.) Kuntze; *Maclura mora* Griseb.; *Maclura trilobata* Rojas Acosta

Tree.

Departmental distribution in Paraguay: Alto Paraguay, Ñeembucú, Presidente Hayes.

Voucher: *J. De Egea & R. Elsam 828* (BM, CTES, FCQ, G, MO, PY, SI, UNR).

##### *Sorocea sprucei* (Baill.) J.F.Macbr. subsp. *saxicola* (Hassl.) C.C.Berg, Proc. Kon. Ned. Akad. Wetensch., C 88(4): 391. 1985.

Syn.: *Maclura brasiliensis* (Mart.) Endl. var. *reticulata* Chodat; *Sorocea saxicola* Hassl.; *Sorocea saxicola* Hassl. forma *subrepanda* Hassl.; *Sorocea saxicola* Hassl. var. *dentata* Hassl.; *Trophisomia edulis* Rojas Acosta

Tree or shrub.

Departmental distribution in Paraguay: Alto Paraguay, Canindeyú, Central, Concepción, Cordillera, Misiones, Ñeembucú, Paraguarí, Presidente Hayes, San Pedro.

Voucher: *J. De Egea & R. Elsam 822* (BM, FCQ).

#### MYRTACEAE

##### *Blepharocalyx salicifolius (Kunth) O.Berg, Linnaea 27(4): 413. 1854 [1856].

Syn.: For complete synonymy see World Checklist of Myrtaceae (Govaerts et al. 2008).

Shrub or tree.

Departmental distribution in Paraguay: Alto Paraná, Amambay, Caaguazú, Caazapá, Canindeyú, Cordillera, Guairá, Ñeembucú, Paraguarí.

Voucher: *L. Bernardi 18376* (G) (http://www.ville-ge.ch/musinfo/bd/cjb/fdp/).

##### *Campomanesia xanthocarpa Mart. exO.Berg, Fl. Bras. (Martius) 14(1): 451. 1857.

Syn.: *Eugenia xanthocarpa* Mart., nom. nud.

Tree.

Departmental distribution in Paraguay: Alto Paraná, Amambay, Caazapá, Canindeyú, Central, Cordillera, Guairá, Misiones, Ñeembucú, Paraguarí.

Voucher: *R. Spichiger et al. 5356* (G) (http://www.ville-ge.ch/musinfo/bd/cjb/fdp/).

##### ►*Eugenia florida* DC., Prodr. (DC.) 3: 283. 1828.

Syn.: *Eugenia atropunctata* Steud.; *Eugenia atropunctata* Steud. var. *gracilis* O.Berg; *Eugenia atropunctata* Steud. var. *robusta* O.Berg; *Eugenia coloradoensis* Standl.; *Eugenia gardneriana* O.Berg; *Eugenia gardneriana* O.Bergvar. *depauperata* O.Berg; *Eugenia gardneriana* O.Bergvar. *dives* O.Berg; *Eugenia gardneriana* O.Bergvar. *ovata* O.Berg; *Eugenia gardneriana* O.Bergvar. *rigida* O.Berg; *Eugenia melanosticta* Standl., nom. illeg.; *Eugenia membranacea* O.Berg; *Eugenia moraviana* O.Berg var. *gardneriana* (O.Berg) Mattos; *Eugenia oligoneura* O.Berg; *Eugenia patula* DC.; *Eugenia perorebi* Parodi ex Speg. & Girola; *Eugenia racemifera* Sagot; *Eugenia seriatoracemosa* Kiaersk.; *Eugenia sylvatica* Cambess.; *Eugenia tinge-lingua* S.Moore

Tree.

Departmental distribution in Paraguay: Amambay, Canindeyú, Cordillera, Ñeembucú, Paraguarí.

Voucher: *L. Bernardi 18375* (BM).

##### *Eugenia hiemalis Cambess., Fl. Bras. Merid. (quarto ed.) 2: 259. 1830.

Syn.: *Eugenia bicolor* O.Berg; *Eugenia cycliantha* D.Legrand; *Eugenia hyemalis* Cambess. var. *marginata* (O.Berg) D.Legrand; *Eugenia lindbergiana* O.Berg; *Eugenia multiflora* Cambess.; *Eugenia multiflora* Cambess. var. *glabra* O.Berg; *Eugenia multiflora* Cambess. var. *lutescens* Cambess.; *Eugenia multiflora* Cambess. var. *rubiginosa* Cambess.; *Eugenia polycarpa* O.Berg; *Eugenia polycarpa* O.Bergvar. *bimarginata* O.Berg; *Eugenia polycarpa* O.Bergvar. *marginata* O.Berg; *Eugenia polycarpa* O.Bergvar. *ovata* O.Berg; *Luma multiflora* Herter; *Myrciaria itacurubiensis* Barb.Rodr. ex Chodat & Hassl.

Tree.

Departmental distribution in Paraguay: Caazapá, Ñeembucú, Paraguarí.

Voucher: *L. Bernardi 18475* (BM).

##### ►*Eugenia mansoi* O.Berg, Fl. Bras. (Martius) 14(1): 223. 1857.

Syn.: *Eugenia vincifolia* O.Berg

Tree.

Departmental distribution in Paraguay: Alto Paraná, Alto Paraguay, Central, Concepción, Cordillera, Ñeembucú, Paraguarí.

Voucher: *J. De Egea & R. Elsam 789* (ASU, BM, CTES, FCQ, G, MO).

##### ►*Eugenia modesta* DC., Prodr. (DC.) 3: 279.1828.

Syn.: *Eugenia bella* Cambess.; *Eugenia modesta* DC. var. *brasiliensis* O.Berg; *Eugenia racemulosa* O.Berg; *Eugenia racemulosa* O.Bergvar. *grandifolia* O.Berg; *Eugenia racemulosa* O.Bergvar. *parvifolia* O.Berg

Tree.

Departmental distribution in Paraguay: Ñeembucú.

Voucher: *J. De Egea & R. Elsam 813* (BM, CTES, FCQ, MO).

##### *Eugenia paracatuana O.Berg, Fl. Bras. (Martius) 14(1): 588. 1857.

Syn.: *Eugenia moraviana* O.Berg var. *impunctata* O.Berg.

Tree or shrub.

Departmental distribution in Paraguay: Caaguazú, Canindeyú, Central, Cordillera, Guairá, Ñeembucú, Paraguarí, Presidente Hayes.

Voucher: *L. Bernardi 18453* (MO) (http://www.tropicos.org/).

##### *Eugenia punicifolia* (Kunth) DC., Prodr. (DC.) 3: 267. 1828.

Syn.: For complete synonymy see World Checklist of Myrtaceae (Govaerts et al. 2008).

Shrub.

Departmental distribution in Paraguay: Amambay, Caazapá, Canindeyú, Cordillera, Ñeembucú, Paraguarí, Presidente Hayes.

Voucher: *M. Peña-Chocarro et al. 2354* (ASU, BM, CTES, FCQ, MO).

##### *Eugenia pyriformis Cambess., Fl. Bras. Merid. (quarto ed.) 2: 336. 1832.

Syn.: *Eugenia dumicola* Barb.Rodr.; *Eugenia turbinata* O.Berg; *Myrciaria dumicola* (Barb.Rodr.) Chodat & Hassl.; *Pseudomyrcianthes pyriformis* (Cambess.) Kausel

Tree.

Departmental distribution in Paraguay: Alto Paraná, Amambay, Caaguazú, Caazapá, Canindeyú, Concepción, Cordillera, Guairá, Ñeembucú, Paraguarí, San Pedro.

Voucher: *L. Bernardi 20491* (G) (http://www.ville-ge.ch/musinfo/bd/cjb/fdp/).

##### *Eugenia uniflora L., Sp. Pl. 1: 470. 1753.

Syn.: For complete synonymy see World Checklist of Myrtaceae (Govaerts et al. 2008).

Tree.

Departmental distribution in Paraguay: Alto Paraguay, Amambay, Caaguazú, Caazapá, Canindeyú, Central, Concepción, Cordillera, Guairá, Ñeembucú, Paraguarí, Presidente Hayes, San Pedro.

Voucher: *L. Bernardi 18456* (G) (http://www.ville-ge.ch/musinfo/bd/cjb/fdp/).

##### *Myrcia multiflora (Lam.) DC., Prodr. (DC.) 3: 244. 1828.

Syn.: For complete synonymy see World Checklist of Myrtaceae (Govaerts et al. 2008).

Tree.

Departmental distribution in Paraguay: Alto Paraná, Caaguazú, Canindeyú, Cordillera, Ñeembucú, Paraguarí, San Pedro.

Voucher: *R. Spichiger et al. 5305* (G) (http://www.ville-ge.ch/musinfo/bd/cjb/fdp/).

##### ►*Myrcia selloi* (Spreng.) N.Silveira, Loefgrenia 89: 5. 1986.

Syn.: *Myrtus selloi* Spreng.; for complete synonymy see World Checklist of Myrtaceae (Govaerts et al. 2008).

Tree.

Departmental distribution in Paraguay: Ñeembucú.

Voucher: *M. Peña-Chocarro et al. 2225* (ASU, BM, CTES, FCQ, G, MO, PY, SI, UNR).

##### ►*Myrcianthes pungens* (O.Berg) D.Legrand, Bol. Fac. Agron. Univ. Montevideo 101: 52. 1969.

Syn.: *Acreugenia pungens* (O.Berg) Kausel; *Eugenia pungens* O.Berg; *Eugenia ybaviyu* Parodi; *Luma pungens* (O.Berg) Herter

Tree.

Departmental distribution in Paraguay: Alto Paraná, Canindeyú, Central, Guairá, Ñeembucú, Paraguarí.

Voucher: *M. Peña-Chocarro et al. 2204* (BM, CTES, FCQ, G, MO).

##### ●*Plinia cauliflora* (DC.) Kausel, Ark. Bot. 3: 508. 1956.

Syn.: *Eugenia cauliflora* (Mart.) DC.; *Eugenia jaboticaba* (Vell.) Kiaersk.; *Myrcia jaboticaba* (Vell.) Baill.; *Myrciaria cauliflora* (Mart.) O.Berg; *Myrciaria jaboticaba* (Vell.) O.Berg; *Myrtus cauliflora* Mart.; *Myrtus jaboticaba* Vell.; *Plinia jaboticaba* (Vell.) Kausel

Tree.

Departmental distribution in Paraguay: Ñeembucú.

Voucher: *J. De Egea & R. Elsam 788* (ASU, BM, CTES, FCQ, G, MO).

##### *Plinia peruviana* (Poir) Govaerts, World Checklist Myrtaceae 44. 2008.

Syn.: *Eugenia cauliflora* Miq., nom. illeg.; *Eugenia guapurium* DC., nom. superfl.; *Eugenia rabeniana* Kiaersk.; *Guapurium fruticosum* Spreng.; *Guapurium peruvianum* Poir.; *Myrciaria guapurium* (DC.) O.Berg, nom. superfl.; *Myrciaria peruviana* (Poir.) Mattos; *Myrciaria peruviana* (Poir.) Mattosvar. *trunciflora* (O.Berg) Mattos; *Myrciaria trunciflora* O.Berg; *Plinia trunciflora* (O.Berg) Kausel

Tree or shrub.

Departmental distribution in Paraguay: Ñeembucú.

Voucher: *L. Bernardi 18441* (cited in Ramella 2010: 159, as *Plinia trunciflora*).

##### *Psidium grandifolium* DC., Prodr. (DC.) 3: 234. 1828.

Syn.: For complete synonymy see World Checklist of Myrtaceae (Govaerts et al. 2008).

Shrub.

Departmental distribution in Paraguay: Amambay, Caaguazú, Caazapá, Canindeyú, Itapúa, Ñeembucú, Paraguarí, San Pedro.

Voucher: Cited in Zuloaga et al. 2008: 2614.

##### *Psidium guajava L., Sp. Pl. 1: 470. 1753.

Syn.: For complete synonymy see World Checklist of Myrtaceae.

Tree or shrub.

Departmental distribution in Paraguay: Alto Paraguay, Amambay, Caaguazú, Caazapá, Canindeyú, Central, Cordillera, Guairá, Ñeembucú, Paraguarí, Presidente Hayes.

Voucher: *M. Peña-Chocarro et al. 2211B* (BM, FCQ).

##### *Psidium guineense Sw., Prodr. (Swartz) 77. 1788.

Syn.: For complete synonymy see World Checklist of Myrtaceae.

Tree or Shrub.

Departmental distribution in Paraguay: Amambay, Caazapá, Canindeyú, Central, Concepción, Cordillera, Ñeembucú, Paraguarí.

Voucher: *L. Bernardi 18396* (BM).

##### *Psidium nutans* O.Berg, Fl. Bras. (Martius) 14(1): 394. 1857.

Syn.: *Guajava nutans* (O.Berg) Kuntze

Tree or Shrub.

Departmental distribution in Paraguay: Cordillera, Ñeembucú.

Voucher: *M. Peña-Chocarro et al. 2366* (BM, CTES, FCQ, G, MO, SI).

#### NYCTAGINACEAE

##### *Pisonia aculeata L., Sp. Pl. 1: 1026. 1753.

Syn.: *Pisonia yaguapinda* D.Parodi

Climber.

Departmental distribution in Paraguay: Alto Paraná, Alto Paraguay, Caazapá, Canindeyú, Cordillera, Guairá, Ñeembucú, Paraguarí, Presidente Hayes.

Voucher: *C. Vogt 131* (CTES, FCQ).

##### ►*Pisonia hassleriana* Heimerl, Oesterr. Bot. Z. 56: 426. 1906.

Syn.: *Guapira hassleriana* (Heimerl) Lundell; *Torrubia hassleriana* (Heimerl) Standl.

Tree.

Departmental distribution in Paraguay: Concepción, Ñeembucú.

Voucher: *J. De Egea & R. Elsam 820* (BM, CTES, FCQ, MO).

#### NYMPHAEACEAE

##### ►*Nymphaea gardneriana* Planch., Fl. Serres Jard. Eur. 8: 120. 1852.

Aquatic herb.

Departmental distribution in Paraguay: Alto Paraguay, Ñeembucú, Paraguarí, San Pedro.

Voucher: *C. Vogt 145* (FCQ).

##### *Nymphaea prolifera* Wiersema, Brittonia 36: 219. 1984.

Aquatic herb.

Departmental distribution in Paraguay: Alto Paraguay, Cordillera, Ñeembucú, Paraguarí.

Voucher: *M. Peña-Chocarro et al. 2371* (BM, CTES, FCQ, MO, SI).

##### ►*Victoria cruziana* A.D.Orb., Ann. Sci. Nat., Bot., sér. 2, 13: 57. 1840.

Aquatic herb.

Departmental distribution in Paraguay: Central, Ñeembucú, Presidente Hayes.

Voucher: *J. De Egea et al. 738* (BM, CTES, FCQ, G, MO, UNR).

#### OCHNACEAE

##### *Sauvagesia erecta* L., Sp. Pl. 1: 203. 1753.

Syn.: Extensive synonymy not cited.

Herb.

Departmental distribution in Paraguay: Alto Paraná, Amambay, Caaguazú, Canindeyú, Cordillera, Guairá, Itapúa, Ñeembucú, Presidente Hayes, San Pedro.

Voucher: *E. Hassler 3815* (G) (cited in Sastre 1971: 46).

#### ONAGRACEAE

##### ►*Ludwigia irwinii* Ramamoorthy, Monogr. Syst. Bot. Missouri Bot. Gard. 19: 66. 1987.

Syn.: *Jussiaea lanceolata* Cambess.

Subshrub.

Departmental distribution in Paraguay: Caazapá, Ñeembucú, Paraguarí.

Voucher: *S. Keel & L. Spinzi 1444* (FCQ).

##### *Ludwigia lagunae (Morong) H.Hara, J. Jap. Bot. 28(10): 292. 1953.

Syn.: *Jussiaea brachycarpa* Lam. subsp. *epilobioides* (Chodat & Hassl.) Hassl.; *Jussiaea brachycarpa* Lam. var. *genuina* Hassl., nom. illeg.; *Jussiaea brachycarpa* Lam. var. *grandiflora* Hassl.; *Jussiaea brachycarpa* Lam. var. *paraguayensis* (Chodat) Hassl.; *Jussiaea brachycarpa* Lam. var. *parviflora* (Chodat & Hassl.) Hassl.; *Jussiaea brachycarpa* Lam. var. *puberula* Hassl.; *Jussiaea brachycarpa* Lam. Micheli, nom. illeg.; *Jussiaea epilobioides* Chodat & Hassl.; *Jussiaea epilobioides* Chodat & Hassl. var. *parviflora* Chodat & Hassl.; *Jussiaea lagunae* Morong; *Jussiaea lagunae* Morongvar. *paraguayensis* (Chodat) Munz; *Jussiaea lagunae* Morongvar. *typica* Munz, nom. illeg.; *Jussiaea leveilleana* Bertoni; *Jussiaea paraguayensis* Chodat; *Jussiaea suffruticosa* L. var. *epilobioides* (Chodat & Hassl.) Bertoni; *Jussiaea suffruticosa* L. var. *paraguayensis* (Chodat) Bertoni; *Jussiaea suffruticosa* L. var. *parviflora* (Chodat & Hassl.) Bertoni

Herb or subshrub.

Departmental distribution in Paraguay: Alto Paraguay, Central, Cordillera, Guairá, Ñeembucú, Presidente Hayes, San Pedro.

Voucher: *R. Spichiger et al. 5272* (BM, CTES, G, MO).

##### ►*Ludwigia major* (Micheli) Ramamoorthy, Monogr. Syst. Bot. Missouri Bot. Gard. 19: 84. 1987.

Syn.: *Jussiaea longifolia* DC. forma *filifolia* Chodat & Hassl.; *Jussiaea longifolia* DC. var. *apaensis* Hassl.; *Jussiaea longifolia* DC. var. *major* Micheli; *Jussiaea pseudonarcissus* Chodat & Hassl. forma *filifolia* (Chodat & Hassl.) Hassl.; *Jussiaea pseudonarcissus* Chodat & Hassl. var. *leptophylla* Chodat & Hassl.

Herb.

Departmental distribution in Paraguay: Amambay, Caaguazú, Caazapá, Canindeyú, Central, Cordillera, Misiones, Ñeembucú, Paraguarí, Presidente Hayes.

Voucher: *M. Vera et al. 214* (BM, CTES, FCQ, MO).

##### *Ludwigia martii* (Micheli) Ramamoorthy, Monogr. Syst. Bot. Missouri Bot. Gard. 19: 64. 1987.

Syn.: *Jussiaea martii* Micheli; *Jussiaea myrtifolia* Cambess. forma *foliosa* Hassl.; *Jussiaea myrtifolia* Cambess. forma *pohliana* Hassl., nom. superfl.; *Jussiaea nervosa* Poir. forma *microphylla* Chodat & Hassl.; *Jussiaea nervosa* Poir. var. *microphylla* (Chodat & Hassl.) Bertoni; *Jussiaea sericea* Cambess. var. *villosissima* Micheli

Herb or subshrub.

Departmental distribution in Paraguay: Amambay, Caaguazú, Cordillera, Ñeembucú.

Voucher: *A.G. Schulz 7677* (CTES, MO) (cited in Ramamoorthy and Zardini 1987: 66).

##### *Ludwigia neograndiflora (Munz) H.Hara, J. Jap. Bot. 28(10): 293. 1953.

Syn.: *Jussiaea neograndiflora* Munz

Herb.

Departmental distribution in Paraguay: Amambay, Caaguazú, Caazapá, Central, Cordillera, Guairá, Ñeembucú, Paraguarí.

Voucher: *M.A. Walter 56* (MO) (http://www.tropicos.org/).

##### ►*Ludwigia octovalvis* (Jacq.) P.H.Raven, Kew Bull. 15(3): 476. 1962.

Syn.: *Jussiaea macropoda* C.Presl; *Jussiaea octonervia* Lam.; *Jussiaea suffruticosa* L. forma *linearifolia* (Hassl.) Munz; *Jussiaea suffruticosa* L. var. *ligustrifolia* (Kunth) Griseb.; *Jussiaea suffruticosa* L. forma *linearifolia* (Hassl.) Munz; *Jussiaea suffruticosa* L. var. *linearifolia* Hassl.; *Jussiaea suffruticosa* L. var. *macropoda* (C.Presl) Munz; *Jussiaea suffruticosa* L. var. *sessiliflora* (Micheli) Hassl.; *Ludwigia octovalvis* (Jacq.) P.H.Raven subsp. *macropoda* (C.Presl) P.H.Raven; *Oenothera octovalvis* Jacq.

Herb or shrub.

Departmental distribution in Paraguay: Alto Paraguay, Amambay, Central, Cordillera, Ñeembucú, Paraguarí, Presidente Hayes.

Voucher: *M. Peña-Chocarro et al. 1996* (BM, CTES, FCQ, MO).

##### ►*Ludwigia peploides* (Kunth) P.H.Raven, Reinwardtia 6(4): 393. 1963.

Syn.: *Jussiaea peploides* Kunth; *Jussiaea repens* auct. non L.; *Jussiaea repens* L. var. *peploides* (Kunth) Griseb.; *Jussiaea repens* L. var. *ramulosa* Hassl.; *Jussiaea swartziana* DC.

Herb.

Departmental distribution in Paraguay: Alto Paraguay, Caaguazú, Central, Concepción, Cordillera, Guairá, Ñeembucú, Presidente Hayes.

Voucher: *S. Keel & L. Spinzi v-133* (FCQ).

##### ►*Ludwigia pseudonarcissus* (Chodat & Hassl.) Ramamoorthy, Monogr. Syst. Bot. Missouri Bot. Gard. 19: 76. 1987.

Syn.: *Jussiaea lithospermifolia* Micheli var. *meridionalis* Hassl., nom. superfl.; *Jussiaea longifolia* DC. forma *grandiflora* Hassl.; *Jussiaea longifolia* DC. forma *parviflora* Hassl., nom. superfl.; *Jussiaea longifolia* DC. subsp. *pseudonarcissus* (Chodat & Hassl.) Hassl.; *Jussiaea longifolia* DC. var. *intermedia* Hassl.; *Jussiaea nervosa* Poir. forma *salicina* Chodat & Hassl.; *Jussiaea pseudonarcissus* Chodat & Hassl.

Herb or subshrub.

Departmental distribution in Paraguay: Amambay, Caazapá, Central, Cordillera, Guairá, Ñeembucú, Paraguarí.

Voucher: *F. Mereles 9738* (FCQ).

#### ORCHIDACEAE

##### *Acianthera recurva (Lindl.) Pridgeon & M.W.Chase, Lindleyana 16(4): 246. 2001.

Sin.: *Pleurothallis bistuberculata* Barb.Rodr.; *Pleurothallis curitybensis* Kraenzl.; *Pleurothallis lamproglossa* Schltr.; *Pleurothallis leucorhoda* Schltr.; *Pleurothallis lilacina* Barb. Rodr.var. *microphylla* Barb.Rodr.; *Pleurothallis recurva* Lindl.; *Pleurothallis recurva* Lindl.var. *microphylla* (Barb.Rodr.) Garay; *Specklinia recurva* (Lindl.) F.Barros

Epiphyte.

Departmental distribution in Paraguay: Guairá, Ñeembucú.

Voucher: *A.G. Schulz 511* (CTES, SP) (cited in Schinini 2010: 46).

##### *Brassavola tuberculata Hook., Bot. Mag. 56: t. 2878. 1829.

Syn.: *Brassavola cebolleta* Rchb.f.; *Brassavola chacoensis* Kraenzl.; *Brassavola fragans* Barb.Rodr.; *Brassavola perrinii* Lindl.; *Brassavola perrinii* Lindl. var. *pluriflora* Hauman; *Brassavola revoluta* Barb.Rodr.

Epiphyte.

Departmental distribution in Paraguay: Central, Guairá, Ñeembucú, San Pedro.

Voucher: *J. De Egea et al. 684* (BM, FCQ).

##### *Campylocentrum neglectum (Rchb.f. & Warm.) Cogn., Bull. Herb. Boissier, sér. 2, 1: 145. 1901.

Syn.: *Aeranthus neglectum* Rchb.f. & Warm.; *Campylocentrum neglectum* (Rchb.f. & Warm.) Cogn. var. *angustifolium* Cogn.

Epiphyte.

Departmental distribution in Paraguay: Alto Paraná, Caazapá, Canindeyú, Central, Concepción, Ñeembucú, Presidente Hayes, San Pedro.

Voucher: *C. Vogt & P. Rios 124* (CTES, FCQ).

##### ►*Galeandra styllomisantha* (Vell.) Hoehne, Arq. Bot. Estado São Paulo 2: 146. 1952.

Syn.: *Galeandra juncea* Lindl.; *Orchis stylomisantha* Vell.

Herb.

Departmental distribution in Paraguay: Amambay, Canindeyú, Concepción, Ñeembucú, Presidente Hayes.

Voucher: *J. De Egea et al. 713* (BM, FCQ).

##### ►*Habenaria gourlieana* Gillies ex Lindl., Gen. Sp. Orchid. Pl. 309. 1835.

Syn.: *Habenaria burkartiana* Hoehne; *Habenaria fastor* Warm. ex Hoehne; *Habenaria spegazziniana* Kraenzl.; *Kusibabella burkartiana* (Hoehne) Szlach.; *Kusibabella gourlieana* (Gillies ex Lindl.) Szlach.; *Macrocentrum mendocinum* Phil.

Herb. In swamps.

Departmental distribution in Paraguay: Caaguazú, Ñeembucú, San Pedro.

Voucher: *J. De Egea et al. 700* (BM, FCQ).

##### *Habenaria johannensis* Barb.Rodr., Gen. Sp. Orchid. 2: 251. 1881.

Syn.: *Habenaria vaupelli* Rchb.f. & Warm.

Herb.

Departmental distribution in Paraguay: Cordillera, Guairá, Ñeembucú.

Voucher: *T. Rojas 9334* (SP, SPF) (cited in Batista et al. 2006: 25).

##### *Oeceoclades maculata (Lindl.) Lindl., Gen. Sp. Orchid. Pl. 237. 1835.

Syn.: *Angraecum maculatum* Lindl.; *Eulophia maculata* Lindl.; *Eulophidium maculatum* (Lindl.) Pfitzer; *Eulophidium maculatum* (Lindl.) Pfitzervar. *pterocarpum* Hauman; *Oeceoclades maculata* (Lindl.) Lindl. var. *pterocarpa* (Hauman) Garay & P.Taylor

Epiphyte. Introduced.

Departmental distribution in Paraguay: Alto Paraná, Caaguazú, Canindeyú, Cordillera, Guairá, Ñeembucú, Paraguarí, Presidente Hayes, San Pedro.

Voucher: *J. De Egea et al. 732* (FCQ).

##### *Oncidium* sp.

Epiphyte.

Voucher: *M. Vera et al. 221* (FCQ).

##### *Sacoila lanceolata (Aubl.) Garay, Bot. Mus. Leafl. 28(4): 351. 1980 [1982].

Syn.: *Limodorum lanceolatum* Aubl.; *Sacoila apetala* (Kraenzl.) Garay; *Satyrium orchidioides* Sw.; *Skeptrostachys sancti-jacobi* (Kraenzl.) Garay; *Stenorrhynchos apetalum* Kraenzl.; *Stenorrhynchos australe* Lindl.; *Stenorrhynchos lanceolatum* (Aubl.) Rich. ex Spreng.; *Stenorrhynchos orchidioides* (Sw.) Rich.; *Stenorrhynchos orchidioides* (Sw.) Rich.(Sw.) Rich. var. *australis* Cogn.; *Stenorrhynchos sancti-jacobi* Kraenzl.; *Stenorrhynchos speciosus* Rchb. f.

Herb.

Departmental distribution in Paraguay: Amambay, Alto Paraguay, Alto Paraná, Boquerón, Caaguazú, Canindeyú, Cordillera, Central, Guairá, Paraguarí, Ñeembucú, Presidente Hayes, San Pedro.

Voucher: *A.G. Shulz 7899* (SI) (cited in Schinini 2010: 235).

##### *Sacoila* sp.

Epiphyte.

Voucher: *C. Vogt & A. Contreras 716* (FCQ).

##### *Trichocentrum pumilum (Lind.) M.W.Chase & N.H.Williams, Lindleyana 16(2): 137. 2001.

Syn.: *Epidendrum ligulatum* Vell.; *Lophiaris pumila* (Lindl.) Braem; *Oncidium pumilum* Lindl.; *Oncidium pumilum* Lindl. var. *angustifolium* Cogn.; *Oncidium pumilum* Lindl. var. *robustum* Cogn.

Epiphyte.

Departmental distribution in Paraguay: Caazapá, Canindeyú, Itapúa, Ñeembucú, Paraguarí, San Pedro.

Voucher: *L. Bernardi 18433* (G) (cited in Schinini 2010: 258).

#### OXALIDACEAE

##### ►*Oxalis paludosa* A.St.-Hil., Fl. Bras. Merid. (quarto ed.) 1: 96. 1824.

Syn.: *Acetosella montevidensis* (Progel) Kuntze; *Acetosella paludosa* (A.St.-Hil.) Kuntze; *Oxalis corrientesensis* R.Knuth; *Oxalis duricaulis* R.Knuth; *Oxalis montevidensis* Progel

Herb.

Departmental distribution in Paraguay: Central, Cordillera, Ñeembucú, Paraguarí.

Voucher: *M. Peña-Chocarro et al. 1982* (BM, CTES, FCQ, MO).

#### PASSIFLORACEAE

##### *Passiflora amethystina J.C.Mikan, Del. Fl. Faun. Bras. 20. 1820.

Syn.: *Passiflora bangii* Mast.; *Passiflora laminensis* Barb.Rodr.; *Passiflora lilacina* M.Roem., nom. nud.; *Passiflora onychina* Lindl.; *Passiflora violacea* Vell.

Climber.

Departmental distribution in Paraguay: Alto Paraná, Alto Paraguay, Caazapá, Canindeyú, Guairá, Ñeembucú, Presidente Hayes, San Pedro.

Voucher: *R. Spichiger et al. 5355* (CTES, G) (http://www.ville-ge.ch/musinfo/bd/cjb/fdp/).

##### ►*Passiflora caerulea* L., Sp. Pl. 2: 959. 1753.

Syn.: *Passiflora caerulea* L. var. *angustifolia* Don; *Passiflora caerulea* L. var. *glauca* Mast.; *Passiflora caerulea* L. var. *glaucophylla* Don; *Passiflora caerulea* L. var. *imbricata* Mast.; *Passiflora caerulea* L. var. *regnellii* Mast.

Climber

Departmental distribution in Paraguay: Alto Paraguay, Caaguazú, Canindeyú, Central, Cordillera, Guairá, Misiones, Ñeembucú, Paraguarí, Presidente Hayes.

Voucher: *J. De Egea et al. 729* (BM, CTES, FCQ, MO).

##### *Passiflora chrysophylla* Chodat, Bull. Herb. Boissier 7(app.1): 75. 1899.

Syn.: *Passiflora australis* Chodat & Hassl.; *Passiflora chrysophylla* Chodat forma *apaensis* Chodat & Hassl.; *Passiflora chrysophylla* Chodat forma *solanacea* Chodat & Hassl.; *Passiflora chrysophylla* Chodat var. *concepcionis* Chodat & Hassl.; *Passiflora chrysophylla* Chodat var. *hastata* Chodat; *Passiflora chrysophylla* Chodat var. *sericea* Chodat & Hassl.; *Passiflora foetida* L. var. *vitacea* Mast.

Climber.

Departmental distribution in Paraguay: Alto Paraná, Alto Paraguay, Amambay, Caaguazú, Canindeyú, Central, Concepción, Cordillera, Itapúa, Misiones, Ñeembucú, Paraguarí, Presidente Hayes, San Pedro.

Voucher: *A.G. Schulz 7895* (CTES) (cited in Deginani 2001: 73)

##### *Passiflora foetida* L., Sp. Pl. 2: 959. 1753.

Syn.: *Passiflora foetida* L. var. *balansae* Chodat; *Passiflora foetida* L. var. *gardneri* Killip; *Passiflora foetida* L. var. *nigelliflora* (Hook.) Mast.; *Passiflora foetida* L. var. *sericea* Chodat & Hassl.; *Passiflora nigelliflora* Hook.

Climber.

Departmental distribution in Paraguay: Alto Paraná, Alto Paraguay, Amambay, Caaguazú, Caazapá, Canindeyú, Central, Concepción, Cordillera, Guairá, Itapúa, Ñeembucú, Paraguarí, Presidente Hayes, San Pedro.

Voucher: *A.G. Schulz 7856* (CTES) (cited in Deginani 2001: 87).

##### *Passiflora gibertii* N.E.Br., Trans. & Proc. Bot. Soc. Edinburgh 20: 58. 1856.

Climber.

Departmental distribution in Paraguay: Central, Concepción, Cordillera, Ñeembucú, Paraguarí, Presidente Hayes.

Voucher: *C. Céspedes 59* (FCQ).

##### *Passiflora misera* Kunth, Nov. Gen. Sp. Pl. (quarto ed.) 2: 136. 1817.

Syn.: *Passiflora maximiliana* Bory; *Passiflora maximiliana* Boryvar. *acutiloba* Chodat; *Passiflora maximiliana* Boryvar. *expansa* Chodat & Hassl.; *Passiflora maximiliana* Boryvar. *retusa* Chodat & Hassl.; *Passiflora translinearis* Rusby

Climber.

Departmental distribution in Paraguay: Alto Paraguay, Amambay, Caaguazú, Central, Concepción, Cordillera, Guairá, Itapúa, Misiones, Ñeembucú, Paraguarí, Presidente Hayes.

Voucher: *J. De Egea & M. Vera 474* (BM, FCQ).

##### *Passiflora suberosa* L., Sp. Pl. 2: 958. 1753.

Syn.: *Passiflora flexuosa* Gardner; *Passiflora minima* L.

Climber.

Departmental distribution in Paraguay: Alto Paraná, Alto Paraguay, Amambay, Caaguazú, Caazapá, Central, Concepción, Cordillera, Guairá, Misiones, Ñeembucú, Paraguarí, Presidente Hayes.

Voucher: *A.G. Schulz 7698* (CTES) (cited in Deginani 2001: 108)

##### *Passiflora* sp.

Climber.

Voucher: *M. Peña-Chocarro et al 2352* (FCQ).

##### ►*Piriqueta morongii* Rolfe, Ann. New York Acad. Sci. 7: 115. 1892.

Syn.: *Piriqueta australis* (Urb.) Arbo; *Piriqueta viscosa* Griseb. subsp. *australis* Urb; *Piriqueta viscosa* Griseb. var. *australis* (Urb.) Urb.

Herb.

Departmental distribution in Paraguay: Alto Paraguay, Amambay, Central, Concepción, Cordillera, Guairá, Ñeembucú, Paraguarí, Presidente Hayes, San Pedro.

Voucher: *J. De Egea et al. 737* (FCQ).

##### *Turnera grandiflora* (Urb.) Arbo, Candollea 40: 176. 1985.

Syn.: *Turnera ulmifolia* L. var. *grandiflora* Urb.; *Turnera ulmifolia* L. var. *surinamensis* auct. non Urb.

Herb.

Departmental distribution in Paraguay: Alto Paraguay, Amambay, Caaguazú, Canindeyú, Central, Concepción, Cordillera, Guairá, Itapúa, Misiones, Ñeembucú, Paraguarí, Presidente Hayes, San Pedro.

Voucher: *J. De Egea et al. 630* (BM, CTES, FCQ, MO).

##### *Turnera orientalis* (Urb.) Arbo, Candollea 40: 177. 1985.

Syn.: *Turnera ulmifolia* auct. non L.; *Turnera ulmifolia* L. var. *orientalis* Urb.

Shrub or subshrub.

Departmental distribution in Paraguay: Alto Paraná, Caaguazú, Canindeyú, Concepción, Itapúa, Misiones, Ñeembucú, Presidente Hayes, San Pedro.

Voucher: *T. Meyer 16354* (CTES) (cited in Arbo 1987: 54).

##### *Turnera weddelliana* Urb. ex Rolfe, Jahrb. Königl. Bot. Gart. Berlin 2: 90. 1883.

Shrub.

Departmental distribution in Paraguay: Alto Paraguay, Amambay, Central, Concepción, Cordillera, Misiones, Ñeembucú, Paraguarí, Presidente Hayes.

Voucher: Cited in Zuloaga et al. 2008: 3085.

##### *Turnera* sp.

Herb.

Voucher: *J. De Egea et al. 704* (FCQ).

#### PHYLLANTHACEAE

##### *Phyllanthus fluitans* Benth. ex Müll.Arg., Linnaea 32: 36. 1863.

Aquatic herb.

Departmental distribution in Paraguay: Ñeembucú, Presidente Hayes.

Voucher: *T. Meyer 16168* (C, LIL, S) (cited in Lourteig 1955: 74).

##### *Phyllanthus niruri L., Sp. Pl. 2: 981. `753.

Syn.: *Phyllanthus lathyroides* Kunth; *Phyllanthus microphyllus* Mart.; *Phyllanthus niruri* L. forma *microphyllus* (Müll.Arg.) G.L.Webster; *Phyllanthus niruri* L. subsp. *lathyroides* (Kunth) G.L.Webster

Herb.

Departmental distribution in Paraguay: Caazapá, Canindeyú, Cordillera, Guairá, Itapúa, Ñeembucú, Paraguarí.

Voucher: *R. Spichiger et al. 5354* (G) (http://www.ville-ge.ch/musinfo/bd/cjb/fdp/).

##### ►*Phyllanthus stipulatus* (Raf.) G.L.Webster, Contr. Gray Herb. 176: 53. 1955.

Syn.: *Moeroris stipulata* Raf.

Herb.

Departmental distribution in Paraguay: Caaguazú, Central, Cordillera, Guairá, Ñeembucú, Paraguarí.

Voucher: *M. Peña-Chocarro et al. 2342* (BM, CTES, FCQ, MO).

#### PHYTOLACCACEAE

##### ►*Petiveria alliacea* L., Sp. Pl. 1: 342. 1753.

Syn.: *Mapa graveolens* Vell.; *Petiveria corrientina* Rojas Acosta; *Petiveria paraguayensis* D.Parodi

Herb.

Departmental distribution in Paraguay: Alto Paraná, Alto Paraguay, Caaguazú, Caazapá, Canindeyú, Central, Cordillera, Guairá, Ñeembucú, Paraguarí.

Voucher: *M. Peña-Chocarro et al. 2301* (BM, CTES, FCQ, G, MO, PY, SI).

##### ►*Rivina humilis* L., Sp. Pl. 1: 121. 1753.

Syn.: *Rivina humilis* L. var. *glabra* L.; *Rivina laevis* L.; *Rivina laevis* L. var. *pubescens* Griseb.; *Rivina paraguayensis* D.Parodi

Herb.

Departmental distribution in Paraguay: Alto Paraguay, Amambay, Canindeyú, Central, Concepción, Cordillera, Guairá, Itapúa, Ñeembucú, Paraguarí, Presidente Hayes, San Pedro.

Voucher: *M. Peña-Chocarro et al. 2310* (BM, CTES, FCQ, G, MO, SI).

##### *Seguieria aculeata* Jacq., Select. Stirp. Amer. Hist. 170. 1763.

Syn.: *Albertokuntzea parvifolia* (Benth.) Kuntze; *Seguieria elliptica* R.E.Fr., nom. illeg.; *Seguieria guaranitica* Speg.; *Seguieria guaranitica* Speg. var. *microphylla* Heimerl; *Seguieria parvifolia* Benth.; *Seguieria securigera* Heimerl; *Seguieria votshii* H.Walter

Shrub.

Departmental distribution in Paraguay: Alto Paraná, Amambay, Caaguazú, Canindeyú, Central, Concepción, Cordillera, Guairá, Ñeembucú, Paraguarí, Presidente Hayes, San Pedro.

Voucher: *E. Hassler 1847b* (G, K) (cited in Rohwer 1982: 252).

#### PIPERACEAE

##### ►*Peperomia aceroana* C.DC., Bull Torrey Bot. Club 25: 572. 1898.

Syn.: *Peperomia malmeana* Dahlst.; *Peperomia pseudoreflexa* C.DC.

Epiphyte.

Departmental distribution in Paraguay: Alto Paraguay, Ñeembucú.

Voucher: *J. De Egea & M. Vera 480* (BM, CTES, FCQ, G, MO).

##### ►*Peperomia blanda* (Jacq.) Kunth, Nov. Gen. Sp. (quarto ed.) 1: 67. 1815.

Syn.: *Peperomia blanda* (Jacq.) Kunth var. *dissimilis* (Kunth) C.DC.; *Peperomia blanda* (Jacq.) Kunth var. *reticulata* C.DC.; *Peperomia ciliata* Kunth; *Peperomia dissimilis* Kunth; *Peperomia hirsuta* Miq.; *Peperomia increscens* Miq.; *Peperomia langsdorffii* (Miq.) Miq. var. *dissimilis* (Kunth) Dahlst.; *Peperomia radicans* C.DC.; *Peperomia rojasii* C.DC.; *Peperomia san-pedritoi* Trel.; *Piper blandum* Jacq.

Epiphyte.

Departmental distribution in Paraguay: Alto Paraguay, Ñeembucú, Presidente Hayes.

Voucher: *M. Vera et al. 222* (BM, FCQ).

##### *Piper amalago L., Sp. Pl. 1: 29. 1753.

Syn.: *Enckea sieberi* Miq.; *Piper amalago* L. var. *medium* (Jacq.) Yunck.; *Piper henshenii* C.DC.; *Piper medium* Jacq.; *Piper plantagineum* Lam.; *Piper reticulatum* Vell., nom. illeg.; *Piper saururoides* Desv. ex Ham.; *Piper sieberi* (Miq.) C.DC.; *Piper syringifolium* Balb. ex Kunth, nom. illeg.

Shrub.

Departmental distribution in Paraguay: Alto Paraná, Amambay, Caaguazú, Caazapá, Canindeyú, Central, Concepción, Cordillera, Guairá, Itapúa, Ñeembucú, Paraguarí, San Pedro.

Voucher: *M. Vera et al. 234* (BM, CTES, FCQ, G, MO).

#### PLANTAGINACEAE

##### *Angelonia gardneri* Hook., Bot. Mag. 66: t. 3754. 1839.

Herb.

Departmental distribution in Paraguay: Alto Paraguay, Amambay, Central, Cordillera, Ñeembucú, Paraguarí, Presidente Hayes, San Pedro.

Voucher: *M. Peña-Chocarro et al. 2350* (BM, CTES, FCQ).

##### *Angelonia integerrima Spreng., Syst. Veg., ed. 16 [Sprengel] 4(2): 235. 1827.

Herb.

Departmental distribution in Paraguay: Caaguazú, Caazapá, Canindeyú, Central, Concepción, Cordillera, Guairá, Itapúa, Ñeembucú, Paraguarí, San Pedro.

Voucher: *J. De Egea & R. Elsam 814* (BM, FCQ).

##### *Bacopa dubia Chodat & Hassl., Bull. Herb. Boissier, sér. 2, 4: 290. 1904.

Herb.

Departmental distribution in Paraguay: Ñeembucú.

Voucher: *R. Spichiger et al. 5281* (CTES, G, MO) (http://www.ville-ge.ch/musinfo/bd/cjb/fdp/).

##### ►*Bacopa salzmanii* (Benth.) Wetts. ex Edwall, Bol. Commiss. Geogr. Estado São Paulo 13: 181. 1879.

Syn.: *Bacopa humilis* (Pennell) Standl.; *Bacopa lilacina* (Pennell) Standl.; *Bacopa violacea* (Pennell) Standl.; *Herpestis salzmannii* Benth.; *Monocardia humilis* Pennell; *Monocardia lilacina* Pennell; *Monocardia violacea* Pennell

Herb.

Departmental distribution in Paraguay: Canindeyú, Cordillera, Ñeembucú.

Voucher: *J. De Egea et al. 698* (BM, FCQ).

##### ►*Bacopa scabra* (Benth.) Descole & Borsini var. *laxiflora* (Benth.) V.C.Souza, Fl. Fanerogam. Estado de São Paulo 3: 303. 2003.

Syn.: *Bacopa auriculata* (B.L.Rob.) Greenm.; *Bacopa laxiflora* (Benth.) Wettst. ex Edwall; *Caconapea auriculata* (B.L.Rob.) Pennell; *Herpestis auriculata* B.L.Rob.; *Herpestis laxiflora* Benth.; *Herpestis parvula* S.Moore; *Mella laxiflora* (Benth.) Pennell

Herb.

Departmental distribution in Paraguay: Concepción, Ñeembucú.

Voucher: *M. Peña-Chocarro et al. 2312* (BM, CTES, FCQ, G, MO).

##### * *Mecardonia procumbens* (Mill.) Small var. *flagellaris* (Cham. & Schltdl.) V.C.Souza, Acta Bot. Bras. 11(2): 186. 1997.

Syn.: *Bacopa chamaedryoides* (Kunth) Wettst. forma *intermedia* Chodat & Hassl.; *Bacopa chamaedryoides* (Kunth) Wettst. var. *flagellaris* (Cham. & Schltdl.) Chodat & Hassl.; *Bacopa flagellaris* (Cham. & Schltdl.) Wettst.; *Bacopa radicata* (Benth.) Descole & Borsini; *Bacopa veronicifolia* (Cham. & Schltdl.) Descole & Borsini; *Bacopa versicolor* Herter & Melch.; *Herpestis flagellaris* Cham. & Schltdl.; *Herpestis flagellaris* Cham. & Schltdl. var. *veronicaefolia* Cham. & Schltdl.; *Herpestis radicata* Benth.; *Mecardonia flagellaris* (Cham. & Schltdl.) Rossow; *Mecardonia flagellaris* (Cham. & Schltdl.) Rossowsubsp. *radicata* (Benth.) Rossow; *Mecardonia montevidensis* (Spreng.) Pennell var. *veronicifolia* (Cham. & Schltdl.) G.Dawson; *Mecardonia radicata* (Benth.) Pennell; *Moniera flagellaris* (Cham. & Schltdl.) Kuntze; *Moniera procumbens* (Mill.) Kuntze forma *albiflora* Kuntze; *Moniera procumbens* (Mill.) Kuntze var. *flagellaris* (Cham. & Schltdl.) Kuntze; *Moniera radicata* (Benth.) Kuntze

Herb.

Departmental distribution in Paraguay: Alto Paraguay, Central, Concepción, Cordillera, Guairá, Ñeembucú, Paraguarí, Presidente Hayes, San Pedro.

Voucher: *M. Peña-Chocarro et al. 1973* (BM, FCQ).

##### *Mecardonia procumbens* (Mill.) Small var. *herniarioides* (Cham.) V.C.Souza, Acta Bot. Bras. 11(2): 187. 1997.

Syn.: *Bacopa chamaedryoides* (Kunth) Wettst. var. *microphylla* (J.A.Schmidt) Edwall; *Bacopa herniarioides* (Cham.) Wettst.; *Herpestis chamaedryoides* Kunth var. *microphylla* J.A.Schmidt; *Herpestis herniarioides* Cham.; *Mecardonia herniarioides* (Cham.) Pennell; *Mecardonia tenella* (Cham.) Pennell var. *microphylla* (J.A.Schmidt) Rossow; *Moniera herniarioides* (Cham.) Kuntze

Herb.

Departmental distribution in Paraguay: Itapúa, Ñeembucú.

Voucher: *T. Meyer 15975* (LIL)(cited in Rossow 1987: 471, as *Mecardonia tenella* var. *microphylla*).

##### *Mecardonia procumbens* (Mill.) Small var. *tenella* (Cham. & Schltdl.) V.C.Souza, Acta Bot. Bras. 11(2): 185. 1997.

Syn.: *Bacopa tenella* (Cham. & Schltdl.) Wettst.; *Bacopa uruguayensis* Herter & Melch.; *Herpestis tenella* Cham. & Schltdl.; *Mecardonia tenella* (Cham. & Schltdl.) Pennell; *Moniera tenella* (Cham. & Schltdl.) Kuntze

Herb.

Departmental distribution in Paraguay: Alto Paraná, Alto Paraguay, Cordillera, Guairá, Itapúa, Misiones, Ñeembucú, Paraguarí.

Voucher: *T. Meyer 16149* (LIL), (cited in Rossow 1987: 469, as *Mecardonia tenella* var. *tenella*).

##### ►*Plantago myosuros* Lam., Tab. Encycl. 1: 342. 1791.

Syn.: *Plantago barbata* G.Forst. var. *taraxacoides* (Speg.) Pilg.; *Plantago chubutensis* Pilg.; *Plantago myosuros* Lam. subsp. *nudiuscula* Pilg.; *Plantago myosuros* Lam. var. *angustifolia* Pilg.; *Plantago myosuros* Lam. var. *hirta* Speg.; *Plantago myosuros* Lam. var. *major* Pilg.; *Plantago myosuros* Lam. var. *nudiuscula* Pilg.; *Plantago myosuros* Lam. var. *parviflora* Speg.; *Plantago myosuros* Lam. var. *taraxacoides* Speg.; *Plantago nigritella* Pilg.; *Plantago pseudomyosuros* Pilg.; *Plantago rojasii* Pilg.; *Plantago taraxacoides* (Speg.) Pilg.

Herb.

Departmental distribution in Paraguay: Caaguazú, Central, Cordillera, Misiones, Ñeembucú, Presidente Hayes, San Pedro.

Voucher: *M. Peña-Chocarro et al. 2228* (BM, CTES, FCQ, MO).

##### *Plantago tomentosa* Lam., Tab. Encycl. 1: 340. 1791.

Syn.: *Plantago achalensis* Pilg.; *Plantago achalensis* Pilg. forma *minor* Pilg.; *Plantago achalensis* Pilg. var. *hirtula* Pilg.; *Plantago affinis* Decne.; *Plantago arechavaletae* Pilg.; *Plantago bicallosa* Decne. var. *hirsutior* Pilg.; *Plantago grisebachii* Hieron.; *Plantago hypolasia* Pilg.; *Plantago oreades* Decne. var. *lanuginosa* Griseb.; *Plantago paralias* Decne.; *Plantago paralias* Decne. subsp. *affinis* (Decne.) Pilg.; *Plantago paralias* Decne. subsp. *dasystachys* (Pilg.) Pilg.; *Plantago paralias* Decne. subsp. *grisebachii* (Hieron.) Pilg.; *Plantago paralias* Decne. subsp. *hypolasia* (Pilg.) Pilg.; *Plantago paralias* Decne. subsp. *leiocalyx* (Pilg.) Pilg.; *Plantago paralias* Decne. subsp. *petiolata* (Pilg.) Pilg.; *Plantago paralias* Decne. subsp. *selloana* (Pilg.) Pilg.; *Plantago paralias* Decne. subsp. *schlechtendaliana* (Pilg.) Pilg.; *Plantago paralias* Decne. var. *achalensis* (Pilg.) Pilg.; *Plantago paralias* Decne. var. *brevifolia* Pilg.; *Plantago paralias* Decne. var. *cordobensis* (Pilg.) Pilg.; *Plantago paralias* Decne. var. *glabrescens* (Pilg.) Pilg.; *Plantago paralias* Decne. var. *lasiophylla* (Pilg.) Pilg.; *Plantago paralias* Decne. var. *mollior* (Pilg.) Pilg.; *Plantago paralias* Decne. var. *saxicola* (Pilg.) Pilg.; *Plantago tomentosa* Lam. subsp. *affinis* (Decne.) Pilg.; *Plantago tomentosa* Lam. subsp. *balansae* Pilg.; *Plantago tomentosa* Lam. subsp. *dasystachys* Pilg.; *Plantago tomentosa* Lam. subsp. *grisebachii* (Hieron.) Pilg.; *Plantago tomentosa* Lam. subsp. *hypolasia* (Pilg.) Pilg.; *Plantago tomentosa* Lam. subsp. *leiocalyx* Pilg.; *Plantago tomentosa* Lam. subsp. *paralias* (Decne.) Pilg.; *Plantago tomentosa* Lam. subsp. *petiolata* Pilg.; *Plantago tomentosa* Lam. subsp. *schlechtendaliana* Pilg.; *Plantago tomentosa* Lam. subsp. *selloana* Pilg.; *Plantago tomentosa* Lam. var. *achalensis* Pilg.; *Plantago tomentosa* Lam. var. *brevifolia* Pilg.; *Plantago tomentosa* Lam. var. *cordobensis* Pilg.; *Plantago tomentosa* Lam. var. *glabrescens* J.A.Schmidt; *Plantago tomentosa* Lam. var. *lasiophylla* Pilg.; *Plantago tomentosa* Lam. var. *mollior* Pilg.; *Plantago tomentosa* Lam. var. *saxicola* Pilg.

Herb.

Departmental distribution in Paraguay: Alto Paraguay, Caaguazú, Caazapá, Canindeyú, Central, Cordillera, Guairá, Ñeembucú, Paraguarí, Presidente Hayes, San Pedro.

Voucher: *T. Meyer 15992* (LIL) (cited in Rahn 1974: 145).

##### *Scoparia dulcis L., Sp. Pl. 1: 116. 1753.

Syn.: *Capraria dulcis* (L.) Kuntze; *Scoparia dulcis* L. var. *tenuifolia* Griseb.; *Scoparia nudicaulis* Chodat & Hassl.; *Scoparia procumbens* Jacq.; *Scoparia purpurea* Ridl.

Herb.

Departmental distribution in Paraguay: Alto Paraguay, Canindeyú, Central, Cordillera, Ñeembucú, Paraguarí, Presidente Hayes.

Voucher: *R. Spichiger et al. 5330* (G) (http://www.ville-ge.ch/musinfo/bd/cjb/fdp/).

##### ►*Scoparia montevidensis* (Spreng.) R.E.Fr., Ark. Bot. 6(9): 22. 1906.

Syn.: *Microcarpaea montevidensis* Spreng.; *Capraria montevidensis* (Spreng.) Kuntze; *Scoparia annua* Cham. & Schltdl.; *Scoparia excelsa* R.E.Fr.; *Scoparia grisebachii* Fritsch; *Scoparia flava* Cham. & Schltdl.; *Scoparia glandulifera* (Fritsch) Descole & Borsini; *Scoparia macrantha* R.E.Fr.; *Scoparia millefoliata* Fritsch; *Scoparia montevidensis* (Spreng.) R.E.Fr. var. *flava* (Cham. & Schltdl.) Chodat; *Scoparia montevidensis* (Spreng.) R.E.Fr. var. *glandulifera* (Fritsch) R.E.Fr.; *Scoparia montevidensis* (Spreng.) R.E.Fr. var. *grisebachii* (Fritsch) Chodat; *Scoparia neglecta* R.E.Fr.

Herb.

Departmental distribution in Paraguay: Alto Paraguay, Caaguazú, Caazapá, Central, Concepción, Ñeembucú, Presidente Hayes.

Voucher: *M. Peña-Chocarro et al. 2208* (BM, CTES, FCQ, G, MO, SI).

##### *Scoparia pinnatifida Cham., Linnaea 8: 22. 1833.

Syn.: *Scoparia multifida* G.Don

Herb.

Departmental distribution in Paraguay: Ñeembucú.

Voucher: *R. Spichiger et al. 5220* (G) (http://www.ville-ge.ch/musinfo/bd/cjb/fdp/).

##### ►*Stemodia lanceolata* Benth., Prodr. (DC.) 10: 384. 1846.

Syn.: *Stemodiacra lanceolata* Benth.; *Stemodiacra lanceolata* Benth. var. *angustifolia* (Chodat & Hassl.) Minod

Herb.

Departmental distribution in Paraguay: Alto Paraguay, Ñeembucú, Presidente Hayes.

Voucher: *M. Peña-Chocarro et al. 2248* (BM, FCQ).

##### ►*Stemodia palustris* A.St.-Hil., Hist. Pl. Remarq. Bresil 216. 1824.

Syn.: *Stemodia gratioliifolia* A.St.-Hil.; *Stemodia linearifolia* Morong; *Stemodia palustris* A.St.-Hil. var. *simplex* J.A.Schmidt

Herb.

Departmental distribution in Paraguay: Alto Paraguay, Central, Cordillera, Ñeembucú, Paraguarí.

Voucher: *M. Peña-Chocarro et al. 2331* (BM, CTES, FCQ, G, MO, SI).

##### *Stemodia stricta Cham. & Schltdl., Linnaea 3: 10. 1828.

Syn.: *Stemodia hyptoides* Cham. & Schltdl. var. *stricta* (Cham. & Schltdl.) G.Dawson; *Stemodia scoparioides* Hassl. ex Minod; *Stemodia stricta* (Cham. & Schltdl.) Kuntze; *Stemodia stricta* Cham. & Schltdl. var. *multidentata* Minod; *Stemodia stricta* Cham. & Schltdl. var. *paucidentata* Minod; *Stemodiacra stricta* (Cham. & Schltdl.) Kuntze var. *glabriuscula* Kuntze

Herb.

Departmental distribution in Paraguay: Alto Paraguay, Ñeembucú.

Voucher: *R. Spichiger et al. 5264* (CTES, G, MO) (http://www.ville-ge.ch/musinfo/bd/cjb/fdp/).

##### ►*Stemodia verticillata* (Mill) Hassl., Trab. Mus. Farmacol. 21: 110. 1909.

Syn.: *Capraria humilis* Aiton; *Erinus verticillatus* Mill.; *Lendneria humilis* (Aiton) Minod; *Lendneria verticillata* (Mill.) Britton; *Stemodia arenaria* Kunth; *Stemodia humilis* (Aiton) G.Dawson; *Stemodia macrotricha* Colla; *Stemodia parviflora* W.T.Aiton; *Stemodiacra verticillata* (Mill.) Kuntze

Herb.

Departmental distribution in Paraguay: Caazapá, Canindeyú, Cordillera, Ñeembucú.

Voucher: *M. Peña-Chocarro et al. 2209* (BM, CTES, FCQ).

#### POACEAE

##### ►*Acroceras zizanioides* (Kunth) Dandy, J. Bot. 69(2): 54. 1931.

Syn.: *Acroceras oryzoides* (Sw.) Stapf; *Panicum oryzoides* Sw., nom. illeg.; *Panicum zizanioides* Kunth

Herb.

Departmental distribution in Paraguay: Alto Paraná, Amambay, Caaguazú, Central, Cordillera, Guairá, Ñeembucú, Paraguarí, Presidente Hayes, San Pedro.

Voucher: *F. Mereles 9730* (FCQ).

##### *Andropogon bicornis L., Sp. Pl. 2: 1046. 1753.

Syn.: *Andropogon bicornis* L. var. *gracillimus* Hack.

Herb.

Departmental distribution in Paraguay: Canindeyú, Ñeembucú, Paraguarí.

Voucher: *C. Vogt 127* (CTES, FACEN, FCQ).

##### ►*Andropogon lateralis* Nees, Fl. Bras. Enum. Pl. 2(1): 329. 1829.

Syn.: *Andropogon brevis* Trin.; *Andropogon herzogii* Hack.; *Andropogon incanus* Hack.; *Andropogon incanus* Hack. var. *lateralis* (Nees) Hack.; *Andropogon incanus* Hack. var. *ramosissimus* Hack.; *Andropogon incanus* Hack. var. *trichocoleus* Hack.; *Andropogon lateralis* Nees var. *incanus* (Hack.) Henrard; *Andropogon lateralis* Nees var. *ramosissimus* (Hack.) Henrard; *Andropogon lateralis* Nees var. *trichocoleus* (Hack.) Henrard; *Andropogon virginicus* auct. non L.; *Andropogon virginicus* L. subsp. *genuinus* auct. non Hack., nom. illeg.

Herb.

Departmental distribution in Paraguay: Alto Paraná, Amambay, Ñeembucú, Paraguarí.

Voucher: *S. Keel & L. Spinzi 1459* (FCQ).

##### *Andropogon* sp.

Herb.

Voucher: *R. Spichiger et al. 5232* (cited in Ramella 2009: 249).

##### *Anthaenantia lanata* (Kunth) Benth., J. Linn. Soc., Bot. 19: 39. 1881.

Syn.: *Anthaenantia lanata* (Kunth) Benth. var. *mollis* (Nees) Arechav.; *Leptocoryphium lanatum* (Kunth) Nees; *Leptocoryphium lanatum* (Kunth) Neesvar. *molle* (Nees) Döll; *Milium juncoides* Speg.; *Milium lanatum* (Kunth) Roem. & Schult.; *Paspalum dentatosulcatum* Arechav.; *Paspalum lanatum* Kunth

Herb.

Departmental distribution in Paraguay: Alto Paraná, Amambay, Caaguazú, Canindeyú, Central, Concepción, Cordillera, Guairá, Ñeembucú, Paraguarí, Presidente Hayes, San Pedro.

Voucher: *T. Meyer 15915* (LIL) (cited in Zuloaga et al. 1994: 197, as *Leptocoryphium lanatum*)

##### *Aristida circinalis Lindm., Kongl. Svenska Vetenskapsakad. Handl., n.s. 34(6): 13. 1900.

Syn.: *Aristida acuminata* Hack.; *Aristida aristiglumis* Caro; *Aristida leptochaeta* Hack.; *Aristida misionum* Mez; *Aristida rosacea* Mez; *Aristida succedanea* Henrard

Herb.

Departmental distribution in Paraguay: Alto Paraguay, Amambay, Caaguazú, Central, Guairá, Misiones, Ñeembucú, Paraguarí, San Pedro.

Voucher: *U.G. Eskuche 2527-8* (G) (http://www.ville-ge.ch/musinfo/bd/cjb/fdp/).

##### *Axonopus compressus (Sw.) P.Beauv., Ess. Agrostogr. 12. 1812.

Syn.: *Agrostis compressa* (Sw.) Poir., nom. illeg.; *Anastrophus compressus* Schltr. ex Döll, nom. illeg.; *Anastrophus platycaulis* (Poir.) Nash ex Small; *Anastrophus platycaulmis* Schltdl. ex. B.D.Jacks.; *Axonopus compressus* (Sw.) P.Beauv. var. *affinis* (Chase) Henderson; *Axonopus compressus* (Sw.) P.Beauv. var. *australis* G.A.Black; *Axonopus compressus* (Sw.) P.Beauv. var. *jesuiticus* Araujo; *Axonopus compressus* (Sw.) P.Beauv. var. *macropodius* (Steud.) G.A.Black; *Axonopus jesuiticus* (Araujo) Valls; *Digitaria platicaulis* (Poir.) Desv.; *Milium compressum* Sw.; *Paspalum compressum* (Sw.) Nees, comb. superfl.; *Paspalum compressum* (Sw.) Raspail; *Paspalum depressum* Steud.; *Paspalum filostachyum* A.Rich. ex Steud.; *Paspalum furcatum* Flüggé var. *parviflorum* Döll; *Paspalum macropodium* Steud.; *Paspalum tristachyon* Lam.; *Paspalum uniflorum* Salzm. ex Steud.

Herb.

Departmental distribution in Paraguay: Caaguazú, Caazapá, Central, Cordillera, Guairá, Itapúa, Ñeembucú, Misiones, Paraguarí.

Voucher: *T. Rojas 5804* (BAA) (http://www.ville-ge.ch/musinfo/bd/cjb/fdp/).

##### ►*Axonopus siccus* (Nees) Kuhlm., Com. Lin. Telegr., Bot. 67(11): 87. 1922.

Syn.: *Axonopus barbatus* (Nees) Kuhlm., nom. illeg.; *Axonopus barbatus* Chase ex Parodi, nom. illeg.; *Axonopus barbigerus* (Kunth) Hitchc.; *Axonopus barbigerus* (Kunth) Hitchc. var. *venturii* G.A.Black; *Axonopus malmei* G.A.Black; *Axonopus rojasii* G.A.Black; *Axonopus siccus* (Nees) Kuhlm. var. *laxior* (Döll) G.A.Black; *Axonopus siccus* (Nees) Kuhlm. var. *scaber* (Pilg.) G.A.Black; *Axonopus ulei* (Hack.) Dedecca; *Paspalum barbatum* Nees ex Trin., nom. illeg.; *Paspalum barbatum* (Trin.) Schult. var. *glabrum* Döll; *Paspalum barbatum* (Trin.) Schult. var. *laxius* Döll; *Paspalum barbatum* (Trin.) Schult. var. *scabrum* Pilg.; *Paspalum barbigerum* Kunth; *Paspalum siccum* Nees; *Paspalum ulei* Hack.

Herb.

Departmental distribution in Paraguay: Amambay, Caaguazú, Central, Concepción, Cordillera, Guairá, Itapúa, Misiones, Ñeembucú, Paraguarí, San Pedro.

Voucher: *S. Keel & L. Spinzi 1463* (FCQ).

##### *Bothriochloa exaristata* (Nash) Henrard, Blumea 4(3): 520. 1941.

Syn.: *Amphilophis exaristatus* Nash; *Andropogon exaristatus* (Nash) Hitchc.; *Andropogon hassleri* Hack.; *Andropogon saccharoides* Sw. var. *hassleri* (Hack.) Ekman; *Andropogon saccharoides* Sw. var. *submuticus* Vasey ex Hack.; *Bothriochloa hassleri* (Hack.) Cabrera, comb. superfl.; *Bothriochloa hassleri* (Hack.) Henrard

Herb.

Departmental distribution in Paraguay: Alto Paraguay, Amambay, Central, Concepción, Ñeembucú, Paraguarí, Presidente Hayes.

Voucher: *T. Rojas 12665* (US) (cited in Vega 2000: 153).

##### *Briza uniolae (Nees) Nees ex Steud., Syn. Pl. Glumac. 1: 283. 1854.

Syn.: *Briza flava* Desv.; *Briza spicigera* (J.Presl) Steud.; *Briza uniolae* (Nees) Steud. var. *modestior* Döll; *Briza uniolae* (Nees) Steud. var. *robustior* Döll; *Chascolytrum spicigerum* J.Presl; *Eragrostis uniolae* Nees; *Poa anomala* Kunth; *Poidium uniolae* (Nees) Matthei

Herb.

Departmental distribution in Paraguay: Caazapá, Central, Guairá, Ñeembucú.

Voucher: *M. Peña-Chocarro et al. 2269* (FCQ).

##### *Chusquea ramosissima Lindm., Kongl. Svenska Vetenskapsakad. Handl., n.s. 34(6): 24. 1900.

Syn.: *Chusquea affinis* Munro ex A.Camus; *Chusquea phacellophora* Pilg.

Herb.

Departmental distribution in Paraguay: Alto Paraná, Canindeyú, Guairá, Itapúa, Ñeembucú, Paraguarí.

Voucher: Cited in Vogt and Mereles 2005: 10.

##### *Coleataenia prionitis* (Nees) Soreng, J. Bot. Res. Inst. Texas 4(2): 692. 2010.

Syn.: *Coleataenia gynerioides* Griseb.; *Panicum gynerioides* (Griseb.) Pilg.; *Panicum prionitis* Nees; *Panicum prionitis* Nees subsp. *gynerioides* (Griseb.) Roseng.; B.R.Arrill. & Izag.; *Panicum prionitis* Nees var. *pallidum* Kuntze; *Panicum prionitis* Nees var. *varium* Kuntze

Herb.

Departmental distribution in Paraguay: Alto Paraná, Caaguazú, Central, Concepción, Cordillera, Guairá, Ñeembucú, Paraguarí, Presidente Hayes.

Voucher: *T. Meyer 16083* (LIL) (cited in Zuloaga et al. 1994: 290, as *Panicum prionitis*).

##### *Digitaria cuyabensis (Trin.) Parodi, Physis (Buenos Aires) 8: 378. 1926.

Syn.: *Panicum cuyabense* Trin.

Herb.

Departmental distribution in Paraguay: Alto Paraguay, Amambay, Central, Cordillera, Ñeembucú, Paraguarí, Presidente Hayes.

Voucher: *T. Rojas 12600a* (BAA, LIL) (cited in Zuloaga et al. 1994: 96).

##### *Digitaria eriostachya* Mez, Bot. Jahrb. Syst. 56(Beibl. 125): 8. 1921.

Syn.: *Digitaria fallens* Parodi

Herb.

Departmental distribution in Paraguay: Caaguazú, Caazapá, Central, Cordillera, Guairá, Misiones, Ñeembucú, Paraguarí.

Voucher: *B. Rosengurtt 5525* (MVFA) (cited in Zuloaga et al. 1994: 101).

##### *Digitaria laxa* (Rchb.) Parodi, Physis (Buenos Aires) 9: 13. 1928.

Syn.: *Panicum recalvum* (Nees) Kunth; *Reimaria laxa* Rchb.

Herb.

Departmental distribution in Paraguay: Central, Concepción, Ñeembucú, Presidente Hayes.

Voucher: *T. Rojas 12854* (LIL) (cited in Zuloaga et al. 1994: 118).

##### *Digitaria similis* Beetle ex Gould, J. Arnold Arbor. 60(2): 320. 1979.

Syn.: *Trichachne affinis* Swallen

Herb.

Departmental distribution in Paraguay: Alto Paraguay, Concepción, Cordillera, Ñeembucú.

Voucher: *B. Rosengurtt 5530* (MVFA) (Zuloaga et al. 1994: 131).

##### *Echinochloa polystachya* (Kunth) Hitchc. var. *spectabilis* (Nees) Mart.Crov., Revista Argent. Agron. 9: 318. 1942.

Syn.: *Echinochloa spectabilis* (Nees) Link; *Panicum spectabile* Nees ex Trin.; *Pseudechinolaena spectabilis* (Nees ex Trin.) Herter

Herb.

Departmental distribution in Paraguay: Central, Ñeembucú, Presidente Hayes.

Voucher: *J.R. Ramírez 153* (BAA) (cited in Zuloaga et al. 1994: 152)

##### ►*Eleusine tristachya* (Lam.) Lam., Tab. Encycl. 1: 203. 1791 [1792].

Syn.: *Cynosurus tristachyos* Lam.; *Eleusine indica* (L.) Gaertn. var. *condensata* Döll; *Eleusine oligostachya* Link; *Eleusine tristachya* (Lam.) Kunth, comb. superfl.

Herb.

Departmental distribution in Paraguay: Central, Misiones, Ñeembucú, Paraguarí.

Voucher: *M. Peña-Chocarro et al. 2265* (BM, FCQ).

##### *Elionurus sp.

Herb.

Voucher: *R. Spichiger et al. 5230* (G) (cited in Ramella 2009: 249).

##### *Eragrostis lugens Nees, Fl. Bras. Enum. Pl. 2(1): 505. 1829.

Syn.: *Eragrostis flaccida* Lindm.; *Eragrostis lugens* Nees forma *pallida* Hack.; *Eragrostis lugens* Nees subsp. *flaccida* (Lindm.) Hack.; *Eragrostis lugens* Nees var. *glabrata* Döll; *Eragrostis pilosa* (L.) P.Beauv. var. *lugens* (Nees) Griseb.; *Poa lugens* (Nees) Kunth

Herb.

Departmental distribution in Paraguay: Alto Paraguay, Central, Concepción, Guairá, Ñeembucú, Paraguarí.

Voucher: *J. De Egea et al. 654-A* (BM, CTES, G, MO, SI).

##### *Eragrostis mexicana* (Hornem.) Link var. *virescens* (J.Presl) S.D.Koch & Sánchez Vega, Phytologia 58(6): 380. 1985.

Syn.: *Eragrostis rahmeri* Phil.; *Eragrostis scabra* Phil.; *Eragrostis virescens* J.Presl; *Poa virescens* (J.Presl) Kunth

Herb.

Departmental distribution in Paraguay: Alto Paraguay, Caaguazú, Central, Ñeembucú.

Voucher: *T. Rojas 12619* (BAA) (cited in Nicora 1998: 100, as *Eragrostis virescens*).

##### *Eriochloa punctata (L.) Desv. ex Ham., Prodr. Pl. Ind. Occid. 5. 1825.

Syn.: *Eriochloa punctata* (L.) Desv. ex Ham. forma *intermedia* Parodi; *Eriochloa punctata* (L.) Desv. ex Ham. var. *parodii* Herter; *Milium punctatum* L.

Herb.

Departmental distribution in Paraguay: Amambay, Caaguazú, Central, Concepción, Ñeembucú, Paraguarí, Presidente Hayes.

Voucher: *T. Rojas 12604* (AS, MO) (http://www.tropicos.org/).

##### *Eustachys distichophylla* (Lag.) Nees, Fl. Bras. Enum. Pl. 2(1) : 418. 1829.

Syn.: *Chloris confertifolia* Trin.; *Chloris distichophylla* Lag.; *Chloris distichophylla* Lag. var. *acuminata* (Trin.) Hack; *Chloris distichophylla* Lag. var. *argentina* Hack.

Herb.

Departmental distribution in Paraguay: Alto Paraná, Alto Paraguay, Amambay, Caaguazú, Caazapá, Canindeyú, Central, Concepción, Cordillera, Guairá, Itapúa, Misiones, Ñeembucú, Paraguarí, Presidente Hayes, San Pedro.

Voucher: *T. Meyer 16178* (LIL) (cited in Molina 1996: 243).

##### *Guadua sp.

Herb.

Voucher: Cited in Vogt and Mereles 2005: 13.

##### *Gymnopogon legrandii Roseng., B.R.Arrill. & Izag., Bol. Fac. Agron. Univ. Montevideo 102: 22. 1968.

Syn.: *Gymnopogon biflorus* Pilg. var. *ciliatilemma* Burkart; *Gymnopogon swallenii* J.P.Smith

Herb.

Departmental distribution in Paraguay: Ñeembucú.

Voucher: *A.G. Schulz 7762* (cited in Ramella 2009: 247).

##### ►*Hymenachne amplexicaulis* (Rudge) Nees, Fl. Bras. Enum. Pl. 2(1): 1829.

Syn.: *Agrostis monostachya* Poir.; *Panicum amplexicaule* Rudge

Herb.

Departmental distribution in Paraguay: Alto Paraná, Alto Paraguay, Amambay, Caaguazú, Caazapá, Central, Concepción, Cordillera, Guairá, Itapúa, Ñeembucú, Paraguarí, Presidente Hayes, San Pedro.

Voucher: *M. Peña-Chocarro et al. 2320* (BM, CTES, FCQ, MO).

##### ►*Hymenachne pernambucensis* (Spreng.) Zuloaga, Amer. J. Bot. 90: 817. 2003.

Syn.: *Agrostis pernambucensis* Spreng.; *Panicum excelsum* Nees; *Panicum pernambucense* (Spreng.) Mez ex Pilg.; *Panicum rivulare* Trin.; *Panicum urticans* L.B.Sm. & Wassh.

Herb.

Departmental distribution in Paraguay: Alto Paraná, Amambay, Caaguazú, Caazapá, Central, Cordillera, Guairá, Ñeembucú, Paraguarí, San Pedro.

Voucher: *S. Keel & L. Spinzi v-142* (FCQ).

##### *Ichnanthus tenuis (J.Presl) Hitchc. & Chase, Contr. U.S. Natl. Herb. 18: 334. 1917.

Syn.: *Ichnanthus parodii* K.E.Rogers var. *villosissimus* K.E.Rogers; *Oplismenus tenuis* J.Presl

Herb.

Departmental distribution in Paraguay: Alto Paraná, Caazapá, Canindeyú, Concepción, Cordillera, Guairá, Ñeembucú, Paraguarí.

Voucher: *T. Rojas 5822* (BAA) (http://www.ville-ge.ch/musinfo/bd/cjb/fdp/).

##### ►*Lasiacis sorghoidea* (Desv. ex Ham.) Hitchc. & Chase, Contr. U.S. Natl. Herb. 18: 338. 1917.

Syn.: *Lasiacis guaranitica* (Speg.) Parodi; *Lasiacis patentiflora* Hitchc. & Chase;

*Lasiacis sorghoidea* (Desv. ex Ham.) Hitchc. & Chase var. *patentiflora* (Hitchc. & Chase) Davidse; *Panicum guaraniticum* Speg.; *Panicum sorghoideum* Desv. ex Ham.

Herb.

Departmental distribution in Paraguay: Alto Paraná, Alto Paraguay, Amambay, Caazapá, Canindeyú, Central, Concepción, Cordillera, Guairá, Ñeembucú, Paraguarí, Presidente Hayes, San Pedro.

Voucher: *M. Peña-Chocarro et al. 2295* (BM, CTES, FCQ, G, MO, PY, SI).

##### ►*Mnesithea selloana* (Hack.) de Koning & Sosef, Blumea 31: 292. 1986.

Syn.: *Coelorachis selloana* (Hack.) A.Camus; *Coelorachis selloana* (Hack.) Herter, comb. superfl.; *Manisuris selloana* (Hack.) Kuntze; *Rottboellia selloana* Hack.

Herb.

Departmental distribution in Paraguay: Guairá, Ñeembucú.

Voucher: *M. Peña-Chocarro et al. 2340* (FCQ).

##### *Ocellochloa stolonifera* (Poir.) Zuloaga & Morrone, Syst. Bot. 34(4): 690. 2009.

Syn.: *Panicum frondescens* G.Mey.; *Panicum stoloniferum* Poir.

Herb.

Departmental distribution in Paraguay: Alto Paraná, Caazapá, Concepción, Cordillera, Guairá, Misiones, Ñeembucú, Paraguarí, San Pedro.

Voucher: *L. Bernardi 18476* (G) (http://www.ville-ge.ch/musinfo/bd/cjb/fdp/).

##### *Oplismenus hirtellus (L.) P.Beauv. subsp. *setarius* (Lam.) Mez ex Ekman, Ark. Bot. 13(10): 33. 1913.

Syn.: *Oplismenus setarius* (Lam.) Roem. & Schult.; *Orthopogon setarius* (Lam.) Spreng.; *Panicum setarium* Lam.

Herb.

Departmental distribution in Paraguay: Alto Paraná, Caaguazú, Canindeyú, Central, Concepción, Cordillera, Guairá, Misiones, Ñeembucú, Paraguarí, San Pedro.

Voucher: *M. Vera et al. 225* (BM, CTES, FCQ).

##### *Oplismenus* sp.

Herb.

Voucher: *R. Spichiger et al. 5344* (G) (Ramella 2009: 249).

##### ►*Oryza latifolia* Desv., J.Bot. Agric. 1: 77. 1813.

Syn.: *Oryza alta* Swallen; *Oryza latifolia* Desv. var. *grandispiculis* A.Chev.; *Oryza sativa* L. var. *latifolia* (Desv.) Döll

Herb.

Departmental distribution in Paraguay: Alto Paraguay, Concepción, Cordillera, Ñeembucú, Presidente Hayes.

Voucher: *M. Peña-Chocarro et al. 2322* (BM, CTES, FCQ, G, MO, PY, SI).

##### ►*Panicum bergii* Arechav., Anales Mus. Nac. Montevideo 1: 147. 1894.

Syn.: *Panicum bergii* Arechav. forma *convoluta* R.A.Palacios; *Panicum burkartii* Zuloaga; *Panicum pilcomayense* Hack.

Herb.

Departmental distribution in Paraguay: Alto Paraguay, Central, Concepción, Misiones, Ñeembucú, Paraguarí, Presidente Hayes.

Voucher: *J. de Egea et al. 654-B* (FCQ).

##### *Panicum dichotomiflorum* Michx., Fl. Bor.-Amer. 1: 48. 1803.

Syn.: *Panicum chloroticum* Nees ex Trin.; *Panicum proliferum* Lam. var. *chloroticum* (Nees ex Trin.) Hack.; *Panicum proliferum* Lam. var. *xanthochlorum* Hack. ex Bertoni, nom. nud.

Herb.

Departmental distribution in Paraguay: Alto Paraná, Alto Paraguay, Amambay, Central, Cordillera, Guairá, Itapúa, Ñeembucú, Paraguarí, Presidente Hayes, San Pedro.

Voucher: *M.A. Walter 161* (K) (cited in Zuloaga et al. 1994: 230).

##### *Panicum hylaeicum* Mez, Notizbl. Bot. Gart. Berlin-Dahlem 7: 75. 1917.

Syn.: *Panicum doellii* Mez; *Panicum laxum* Sw. var. *amplissimum* Hack.

Herb.

Departmental distribution in Paraguay: Central, Concepción, Cordillera, Ñeembucú, Presidente Hayes.

Voucher: *A. Reales 231* (LIL) (cited in Zuloaga et al. 1994: 246).

##### ►*Panicum schwackeanum* Mez, Bot. Jahbr. Syst. 56(Beibl. 125): 1. 1921.

Syn.: *Panicum helobium* Mez ex Henrard

Herb.

Departmental distribution in Paraguay: Alto Paraná, Amambay, Caaguazú, Canindeyú, Central, Cordillera, Ñeembucú, San Pedro.

Voucher: *S. Keel & L. Spinzi 1448* (FCQ).

##### *Parodiophyllochloa pantricha (Hack.) Zuloaga & Morrone, Syst. Bot. 33(1): 73. 2008.

Syn.: *Dichanthelium pantrichum* (Hack.) Davidse; *Panicum pantrichum* Hack.; *Panicum protractum* Mez; *Panicum warmingii* Mez

Herb.

Departmental distribution in Paraguay: Amambay, Caaguazú, Caazapá, Concepción, Cordillera, Guairá, Ñeembucú.

Voucher: *T. Rojas 5765* (BAA) (http://www.ville-ge.ch/musinfo/bd/cjb/fdp/).

##### *Paspalum almum* Chase, J. Wash. Acad. Sci. 23(3): 137. 1933.

Syn.: *Paspalum hexastachyum* Parodi; *Panicum ovale* Nees ex Steud. var. *apiculatum* Hack.

Herb.

Departmental distribution in Paraguay: Alto Paraguay, Central, Cordillera, Misiones, Ñeembucú, Paraguarí, Presidente Hayes.

Voucher: *T. Rojas 12599* (BAA, LIL, US) (cited in Zuloaga and Morrone 2005: 31).

##### *Paspalum arundinellum* Mez, Bot. Jahbr. Syst. 56(Beibl. 125): 11. 1921.

Herb.

Departmental distribution in Paraguay: Alto Paraguay, Caaguazú, Central, Cordillera, Guairá, Ñeembucú, Paraguarí, Presidente Hayes.

Voucher: *T. Rojas 12606* (LIL, US) (cited in Zuloaga and Morrone 2005: 40).

##### *Paspalum conspersum* Schrad., Mant. 2 (Schultes) 174. 1824.

Syn.: *Paspalum virgatum* L. var. *conspersum* (Schrad.) Döll

Herb.

Departmental distribution in Paraguay: Alto Paraná, Alto Paraguay, Amambay, Canindeyú, Central, Cordillera, Ñeembucú, Presidente Hayes.

Voucher: *T. Rojas 12606a* (LIL, US) (cited in Zuloaga and Morrone 2005: 68).

##### *Paspalum notatum* Flüggé, Gram. Monogr., Paspalum 106. 1810.

Syn.: *Paspalum notatum* Flüggé var. *latiflorum* Döll; *Paspalum notatum* Flüggé var. *typicum* Parodi, nom. illeg.; *Paspalum saltense* Arechav.; *Paspalum uruguayense* Arechav.

Herb.

Departmental distribution in Paraguay: Alto Paraguay, Amambay, Caaguazú, Caazapá, Central, Concepción, Cordillera, Guairá, Itapúa, Ñeembucú, Paraguarí, Presidente Hayes.

Voucher: *J.R. Ramírez 131* (US) (cited in Zuloaga and Morrone 2005: 183).

##### ►*Paspalum plenum* Chase, Contr. U.S. Natl. Herb. 28: 202. 1929.

Herb.

Departmental distribution in Paraguay: Amambay, Canindeyú, Central, Cordillera, Ñeembucú.

Voucher: *M. Peña-Chocarro et al. 2223* (BM, CTES, FCQ, G, MO, PY, SI, UNR).

##### *Paspalum plicatulum* Michx., Fl. Bor.-Amer. 1: 45. 1803.

Syn.: *Panicum plicatulum* (Michx.) Kuntze; *Paspalum lenticulare* Kunth forma *intumescens* (Döll) Killeen; *Paspalum montevidense* Spreng.; *Paspalum plicatulum* Michx. subsp. *montevidense* (Spreng.) Roseng. B.R.Arrill. & Izag.; *Paspalum plicatulum* Michx. var. *genuinum* Parodi, nom. illeg.; *Paspalum plicatulum* Michx. var. *glabrum* Arechav.; *Paspalum plicatulum* Michx. var. *intumescens* Döll; *Paspalum plicatulum* Michx. var. *longipilum* Hack.; *Paspalum plicatulum* Michx. var. *villosissimum* Pilg.; *Paspalum ramosum* Swallen

Herb.

Departmental distribution in Paraguay: Alto Paraguay, Amambay, Caaguazú, Central, Concepción, Cordillera, Guairá, Itapúa, Misiones, Ñeembucú, Paraguarí, Presidente Hayes, San Pedro.

Voucher: *M. Peña-Chocarro et al. 2232* (BM, CTES, FCQ, G, MO).

##### ►*Paspalum rufum* Nees ex Steud., Syn. Pl. Glumac. 1: 26. 1853.

Syn.: *Paspalum giganteum* Arechav., nom. illeg.; *Paspalum hassleri* Hack.; *Paspalum ostenii* Herter

Herb.

Departmental distribution in Paraguay: Caaguazú, Central, Cordillera, Guairá, Ñeembucú, Paraguarí, Presidente Hayes, San Pedro.

Voucher: *S. Keel & L. Spinzi 1460* (FCQ).

##### *Paspalum simplex* Morong, Ann. New York Acad. Sci. 7: 258. 1893.

Syn.: *Paspalum malacophyllum* Trin. var. *linearifolium* Hack.

Herb.

Departmental distribution in Paraguay: Alto Paraná, Alto Paraguay, Central, Cordillera, Itapúa, Misiones, Ñeembucú, Paraguarí, Presidente Hayes.

Voucher: *B. Rosengurtt 5527* (K, P, US) (cited in Zuloaga and Morrone 2005: 241).

##### *Paspalum wrightii* Hitchc. & Chase, Contr. U.S. Natl. Herb. 18(7): 310. 1917.

Syn.: *Paspalum hydrophilum* Henrard; *Paspalum plicatulum* Michx. var. *multinode* Hack.; *Paspalum virgatum* L. var. *subplicatum* Hack.

Herb.

Departmental distribution in Paraguay: Alto Paraguay, Ñeembucú, Presidente Hayes.

Voucher: *J.R. Ramírez 132* (CTES) (cited in Zuloaga and Morrone 2005: 261).

##### ►*Pharus lappulaceus* Aubl., Hist. Pl. Guiane 2: 859. 1775.

Syn.: *Pharus angustifolius* (Nees) Döll; *Pharus brasiliensis* Raddi; *Pharus glaber* Kunth; *Pharus glaber* Kunthvar. *pubescens* Döll; *Pharus lancifolius* Desv. ex Ham.; *Pharus micranthus* Schrad. ex Nees

Herb.

Departmental distribution in Paraguay: Alto Paraná, Caazapá, Canindeyú, Ñeembucú, Paraguarí.

Voucher: *M. Vera et al. 223* (BM, CTES, FCQ, G, MO).

##### *Schizachyrium* sp.

Herb.

Voucher: Cited in Vogt and Mereles 2005: 11.

##### ►*Setaria fiebrigii* R.A.W.Herrm., Beitr. Biol. Pflanzen 10(1): 56. 1910.

Syn.: *Setaria onurus* (Willd.) Griseb. forma *ramulosa* Hack.

Herb.

Departmental distribution in Paraguay: Alto Paraguay, Caazapá, Central, Concepción, Guairá, Itapúa, Misiones, Ñeembucú, Paraguarí, Presidente Hayes.

Voucher: *M. Peña-Chocarro et al. 2268* (BM, CTES, FCQ).

##### *Setaria globulifera* (Steud.) Griseb., Abh. Königl. Ges. Wiss. Göttingen 24: 307. 1879.

Syn.: *Panicum globuliferum* Steud.; *Setaria berroi* Hack.; *Setaria dura* R.A.W.Herrm.; *Setaria paucifolia* (Morong) Lindm. var. *planifolia* Hack.

Herb.

Departmental distribution in Paraguay: Alto Paraguay, Caazapá, Central, Itapúa, Ñeembucú, San Pedro.

Voucher: *T. Meyer 16142* (LIL) (cited in Pensiero 1999: 61).

##### *Setaria lachnea* (Nees) Kunth, Enum. Pl. [Kunth] 1: 154. 1833.

Syn.: *Chaetochloa argentina* (R.A.W.Herrm.) Hitchc.; *Panicum lachneum* Nees; *Setaria argentina* R.A.W.Herrm.; *Setaria leiantha* Hack.; *Setaria setosa* (Sw.) P.Beauv. forma *leianthina* Hack.; *Setaria setosa* (Sw.) P.Beauv. forma *microstachya* Hack.

Herb.

Departmental distribution in Paraguay: Alto Paraguay, Central, Concepción, Cordillera, Guairá, Ñeembucú, Paraguarí.

Voucher: *F. Kurtz 22* (CORD) (cited in Pensiero 1999: 70, as *Curtz*).

##### ►*Setaria parviflora* (Poir.) Kerguélen, Lejeunia, n.s. 120: 161. 1987.

Syn.: *Cenchrus parviflorus* Poir.; *Panicum dasyurum* Willd. ex Nees; *Panicum flavum* Nees; *Panicum geniculatum* Poir.; *Panicum imberbe* Poir. var. *dasyurum* (Willd. ex Nees) Döll; *Panicum penicillatum* Willd. ex Nees, nom. illeg.; *Setaria discolor* Hack.; *Setaria flava* (Nees) Kunth; *Setaria geniculata* P.Beauv.; *Setaria geniculata* P.Beauv. var. *pauciseta* Desv.; *Setaria glauca* (L.) P.Beauv. var. *imberbis* (Poir.) Griseb.; *Setaria glauca* (L.) P.Beauv. var. *penicillata* (Willd.) Griseb.; *Setaria gracilis* Kunth; *Setaria gracilis* Kunth forma *breviglumis* Colla; *Setaria gracilis* Kunth forma *breviseta* Steud.; *Setaria gracilis* Kunth forma *brevispica* L.; *Setaria gracilis* Kunth forma *flaviseta* Kausel; *Setaria gracilis* Kunth forma *longiseta* Speg.; *Setaria gracilis* Kunth forma *megalantha* D.Legrand; *Setaria gracilis* Kunth forma *purpurascens* Burret; *Setaria gracilis* Kunth forma *radicans* Molina; *Setaria gracilis* Kunthvar. *glauco-caesia* Arechav.; *Setaria gracilis* Kunthvar. *purpurascens* (Kunth) Arechav.; *Setaria imberbis* (Poir.) Roem. & Schult.; *Setaria imberbis* (Poir.) Roem. & Schult. forma *brevispica* Steud.; *Setaria imberbis* (Poir.) Roem. & Schult. forma *flaviseta* L.; *Setaria imberbis* (Poir.) Roem. & Schult. forma *longiseta* Hack.; *Setaria imberbis* (Poir.) Roem. & Schult. forma *radicans* Speg.; *Setaria imberbis* (Poir.) Roem. & Schult. forma *setis-longioribus* Kausel; *Setaria imberbis* (Poir.) Roem. & Schult. forma *uberior* Phil.; *Setaria imberbis* (Poir.) Roem. & Schult. var. *genuina* Hack., nom. illeg.; *Setaria imberbis* (Poir.) Roem. & Schult. var. *gracilis* (Kunth) Hack; *Setaria imberbis* (Poir.) Roem. & Schult. var. *purpurascens* (Kunth) Hack.; *Setaria penicillata* (Willd.) J.Presl; *Setaria purpurascens* Kunth

Herb.

Departmental distribution in Paraguay: Alto Paraná, Alto Paraguay, Amambay, Caaguazú, Caazapá, Canindeyú, Central, Concepción, Cordillera, Guairá, Itapúa, Ñeembucú, Paraguarí, Presidente Hayes.

Voucher: *M. Peña-Chocarro et al. 2266* (BM, CTES, FCQ, G, MO, SI).

##### *Setaria sulcata* Raddi, Agrostogr. Bras. 50. 1823.

Syn.: *Agrostis flabellata* Salzm. ex Steud., nom. nud.; *Chaetochloa poiretiana* (Schult.) Hitchc.; *Panicum poiretianum* Schult.; *Setaria poiretiana* (Schult.) Kunth

Herb.

Departmental distribution in Paraguay: Alto Paraná, Amambay, Caaguazú, Caazapá, Canindeyú, Central, Guairá, Itapúa, Ñeembucú, Paraguarí, Presidente Hayes, San Pedro.

Voucher: *Curtz 414* (CORD) (cited in Pensiero 1999: 99, as *Setaria poiretiana*).

##### ►*Setaria vulpiseta* (Lam.) Roem. & Schult., Syst. Veg., ed. 15 bis [Roemer & Schultes] 2: 495. 1817.

Syn.: *Panicum vulpisetum* Lam.; for complete synonymy see Penseiro (2003) or http://www.tropicos.org/Name/25511505?projectid=10.

Herb.

Departmental distribution in Paraguay: Alto Paraná, Amambay, Caaguazú, Caazapá, Canindeyú, Central, Concepción, Cordillera, Guairá, Itapúa, Ñeembucú, Paraguarí, San Pedro.

Voucher: *M. Vera et al. 236* (BM, CTES, FCQ, MO).

##### ►*Steinchisma laxa* (Sw.) Zuloaga, Amer. J. Bot. 90: 817. 2003.

Syn.: *Panicum laxum* Sw.; for complete synonymy see Zuloaga and Morrone (2003) or http://www.tropicos.org/Name/50232607?projectid=10.

Herb.

Departmental distribution in Paraguay: Alto Paraná, Alto Paraguay, Amambay, Caaguazú, Caazapá, Canindeyú, Central, Concepción, Cordillera, Guairá, Misiones, Ñeembucú, Paraguarí, Presidente Hayes, San Pedro.

Voucher: *M. Peña-Chocarro et al. 2214* (BM, FCQ).

##### *Stephostachys mertensii* (Roth) Zuloaga & Morrone, Taxon 59(5): 1540. 2010.

Syn.: *Panicum megiston* Schult.; *Panicum mertensii* Roth

Herb.

Departmental distribution in Paraguay: Amambay, Central, Concepción, Ñeembucú, Paraguarí, Presidente Hayes, San Pedro.

Voucher: *J.R. Ramírez 151* (BAA) (cited in Zuloaga et al. 1994: 256, as *Panicum mertensii*).

##### *Tridens brasiliensis* (Nees & Steud.) Parodi, Revista Argent. Agron. 4(4): 245. 1937.

Syn.: *Triodia brasiliensis* (Nees ex Steud.) Lindm.; *Triodia figueirae* Arechav.; *Uralepis brasiliensis* Nees ex Steud.

Herb.

Departmental distribution in Paraguay: Central, Concepción, Ñeembucú, Paraguarí, Presidente Hayes, San Pedro.

Voucher: *M. Peña-Chocarro et al. 2230* (BM, CTES, FCQ, G, MO).

#### POLYGALACEAE

##### ►*Acanthocladus albicans* A.W.Benn., Fl. Bras. (Martius) 13(3): 46. 1874.

Syn.: *Polygala albicans* (A.W.Benn.) Grondona; *Polygala bennetti* Chodat

Tree.

Departmental distribution in Paraguay: Alto Paraguay, Amambay, Central, Concepción, Cordillera, Ñeembucú, Presidente Hayes.

Voucher: *J. De Egea & R. Elsam 793* (BM, FCQ).

##### *Polygala hygrophila Kunth, Nov. Gen. Sp. (quarto ed.) 5: 395. 1821 [1823].

Syn.: *Polygala weddelliana* Chodat

Herb.

Departmental distribution in Paraguay: Caaguazú, Caazapá, Canindeyú, Ñeembucú.

Voucher: *R. Spichiger et al. 5221* (G) (http://www.ville-ge.ch/musinfo/bd/cjb/fdp/).

##### *Polygala molluginifolia* A.St.-Hil. & Moq., Mém. Mus. Hist. Nat. 17: 349. 1828.

Syn.: *Polygala areguensis* A.W.Benn.; *Polygala galioides* auct. non Poir.; *Polygala molluginifolia* forma *albiflora* Kuntze; *Polygala molluginifolia* A.St.-Hil. & Moq. forma *rosea* Kuntze; *Polygala orthiocarpa* Chodat; *Polygala verticillata* L. var. *molluginifolia* (A.St.-Hil. & Moq.) Arechav.

Herb.

Departmental distribution in Paraguay: Alto Paraná, Alto Paraguay, Caaguazú, Canindeyú, Central, Concepción, Cordillera, Guairá, Itapúa, Misiones, Ñeembucú, Paraguarí, Presidente Hayes, San Pedro.

Voucher: *M. Peña-Chocarro et al. 1972* (BM, CTES, FCQ, G, MO).

##### *Polygala timoutoides* Chodat, Mém. Soc. Phys. Genève 30(pt. 2, no. 8): 112. 1889.

Syn.: *Polygala timoutou* Aubl. var. *nana* auct. non A.W.Benn.

Herb.

Departmental distribution in Paraguay: Amambay, Caaguazú, Canindeyú, Central, Concepción, Cordillera, Guairá, Misiones, Ñeembucú, Paraguarí, Presidente Hayes, San Pedro.

Voucher: Cited in Zuloaga et al. 2008: 2780.

#### POLYGONACEAE

##### *Coccoloba cordata* Cham., Bot. Jahrb. Syst. 13: 201. 1891.

Syn.: *Coccoloba candolleana* Meisn.; *Coccoloba cordata* Cham. var. *praecox* Hassl.; *Coccoloba tiliacea* Lillo, nom. illeg.

Tree or shrub.

Departmental distribution in Paraguay: Alto Paraguay, Canindeyú, Central, Concepción, Itapúa, Ñeembucú, Paraguarí, Presidente Hayes.

Voucher: *M. Vera et al. 211* (BM, CTES, FCQ, G, MO).

##### ►*Persicaria acuminata* (Kunth) M.Gómez, Anales Inst. Segunda Enseñ. 2: 278. 1896.

Syn.: *Polygonum acuminatum* Kunth; *Polygonum acuminatum* Kunth var. *glabrescens* Meisn.; *Polygonum acuminatum* Kunth var. *microstemom* Mart. ex Meisn.; *Polygonum acuminatum* Kunth var. *setigerum* (Wedd.) Meisn.; *Polygonum alfredi* Pilg.; *Polygonum bettfreundianum* Lindau; *Polygonum floribundum* Wedd.; *Polygonum setigerum* Wedd.

Herb.

Departmental distribution in Paraguay: Alto Paraná, Alto Paraguay, Amambay, Caaguazú, Caazapá, Canindeyú, Central, Concepción, Cordillera, Guairá, Misiones, Ñeembucú, Paraguarí, Presidente Hayes, San Pedro.

Voucher: *M. Peña-Chocarro et al. 2344* (BM, CTES, FCQ, G, MO, PY, SI, UNR).

##### ►*Persicaria ferruginea* (Wedd.) Soják, Preslia 46(2): 153. 1974.

Syn.: *Polygonum ferrugineum* Wedd.; *Polygonum gummifera* Wedd.; *Polygonum spectabile* Mart. ex Meisn.; *Polygonum spectabile* Mart. ex Meisn. var. *minor* Chodat

Herb.

Departmental distribution in Paraguay: Central, Concepción, Cordillera, Ñeembucú, Paraguarí, Presidente Hayes.

Voucher: *M.A. Walter 85* (BM).

##### ►*Persicaria hispida* (Kunth) M.Gómez, Anales Inst. Segunda Enseñ. 2: 278. 1896.

Syn.: *Polygonum hispidum* Kunth

Herb.

Departmental distribution in Paraguay: Alto Paraguay, Central, Cordillera, Ñeembucú, Presidente Hayes.

Voucher: *M.A. Walter 80* (BM).

##### ►*Persicaria hydropiperoides* (Michx.) Small, Fl. S.E. U.S. [Small] 378, 1330. 1903.

Syn.: *Polygonum hydropiperoides* Michx. var. *setaceum* (Baldwin ex Elliott) Gleason; *Polygonum hydropiperoides* Michx. var. *virgatum* (Cham. & Schltdl.) Meisn.; *Polygonum setaceum* Baldwin ex Elliott; *Polygonum virgatum* Cham. & Schltdl.

Herb.

Departmental distribution in Paraguay: Caazapá, Central, Cordillera, Guairá, Itapúa, Misiones, Ñeembucú, Paraguarí, Presidente Hayes, San Pedro.

Voucher: *M. Peña-Chocarro et al. 2231* (BM, CTES, FCQ, G, MO, SI).

##### ►*Persicaria meisneriana* (Cham. & Schltdl.) M.Gómez, Anales Inst. Segunda Enseñ. 2: 278. 1896.

Syn.: *Polygonum beyrichianum* Cham. & Schltdl.; *Polygonum chamissoeanum* Wedd.; *Polygonum meisnerianum* Cham. & Schltdl.; *Polygonum meisnerianum* Cham. & Schltdl. var. *beyrichianum* (Cham. & Schltdl.) Meisn.; *Polygonum meisnerianum* Cham. & Schltdl. var. *setosum* Chodat

Herb.

Departmental distribution in Paraguay: Alto Paraná, Amambay, Caazapá, Central, Cordillera, Guairá, Itapúa, Misiones, Ñeembucú, Paraguarí, San Pedro.

Voucher: *S. Keel & L. Spinzi v-171* (FCQ).

##### *Persicaria paraguayensis* (Wedd.) S.T.Kim & Donoghue, Amer. J. Bot. 95(9): 1135. 2008.

Syn.: *Polygonum paraguayense* Wedd.

Herb.

Departmental distribution in Paraguay: Central, Concepción, Cordillera, Ñeembucú, Presidente Hayes, San Pedro.

Voucher: *F. Kurtz 417* (cited in Cialdella and Brandbyge 2001: 60, as *Polygonum paraguayense*).

##### *Persicaria punctata* (Elliot) Small, Fl. S.E. U.S. [Small]. 379, 1330. 1903.

Syn.: *Polygonum acre* Kunth, nom. illeg.; *Polygonum epilobioides* Wedd.; *Polygonum punctatum* Elliott

Herb.

Departmental distribution in Paraguay: Alto Paraná, Alto Paraguay, Amambay, Caazapá, Canindeyú, Central, Concepción, Cordillera, Guairá, Itapúa, Ñeembucú, Paraguarí, Presidente Hayes, San Pedro.

Voucher: *M.A. Walter 82* (BM).

##### *Ruprechtia laxiflora* Meisn., Fl. Bras. (Martius) 5(1): 56. 1855.

Syn.: *Enneatypus nordenskjoeldii* Herzog; *Enneatypus nordenskjoeldii* Herzog forma *glaberrimus* Herzog; *Magonia laxiflora* (Meisn.) Kuntze; *Magonia polystachya* (Griseb.) Kuntze; *Magonia viraru* (Griseb.) Kuntze; *Ruprechtia polystachya* Griseb.; *Ruprechtia viraru* Griseb.; *Triplaris polystachya* (Griseb.) Kuntze

Tree.

Departmental distribution in Paraguay: Alto Paraná, Caazapá, Canindeyú, Central, Concepción, Cordillera, Guairá, Itapúa, Misiones, Ñeembucú, Paraguarí, Presidente Hayes, San Pedro.

Voucher: *L. Bernardi 18478* (AAU, BM, G).

#### PONTEDERIACEAE

##### *Eichhornia azurea* (Sw.) Kunth, Enum. Pl. [Kunth] 4: 129. 1843.

Syn.: *Piaropus azureus* (Sw.) Raf.; *Pontederia aquatica* Vell.; *Pontederia azurea* Sw.

Aquatic herb.

Departmental distribution in Paraguay: Alto Paraná, Central, Cordillera, Guairá, Misiones, Ñeembucú, Paraguarí, Presidente Hayes.

Voucher: *M. Peña-Chocarro et al. 2317* (BM, FCQ).

##### ►*Eichhornia crassipes* (Mart.) Solms, Monogr. Phan. 4: 527. 1883.

Syn.: *Eichhornia speciosa* Kunth; *Heteranthera formosa* Miq.; *Piaropus mesomelas* Raf.; *Pontederia crassipes* Mart.

Aquatic herb.

Departmental distribution in Paraguay: Central, Concepción, Guairá, Ñeembucú, Presidente Hayes, San Pedro.

Voucher: *M.A. Walter 3* (BM).

##### ►*Heteranthera limosa* (Sw.) Willd., Ges. Naturf. Freunde Berlin Neue Schriften 3: 439. 1801.

Syn.: *Heteranthera alismoides* Humb. ex Link; *Heteranthera limosa* (Sw.) Willd. forma *albiflora* Benke; *Leptanthus ovalis* Michx.; *Lunania uniflora* Raf., nom. illeg.; *Phrynium limosum* (Sw.) Kuntze; *Pontederia limosa* Sw.; *Pontederia triandra* Banks ex Mart.; *Schollera limosa* (Sw.) Raf.; *Triexastima uniflora* Raf.

Aquatic herb.

Departmental distribution in Paraguay: Alto Paraguay, Central, Cordillera, Ñeembucú, Presidente Hayes.

Voucher: *J. De Egea et al. 736* (BM, FCQ).

##### *Heteranthera reniformis* Ruiz & Pav., Fl. Peruv. [Ruiz & Pavon] 1: 43. 1798.

Syn.: *Buchosia aquatica* Vell.; *Heterandra reniformis* (Ruiz & Pav.) P.Beauv.; *Heteranthera acuta* Willd.; *Heteranthera virginica* Steud., nom. illeg.; *Leptanthus peruviana* Pers.; *Leptanthus reniformis* (Ruiz & Pav.) Michx.; *Leptanthus virginica* Pers.; *Phrynium reniformis* (Ruiz & Pav.) Kuntze; *Phrynium reniformis* (Ruiz & Pav.) Kuntzevar. *acuta* (Willd.) Kuntze; *Schollera reniformis* (Ruiz & Pav.) Kuntze

Herb. In swamps.

Departmental distribution in Paraguay: Amambay, Caaguazú, Canindeyú, Central, Concepción, Cordillera, Guairá, Misiones, Ñeembucú, Paraguarí, San Pedro.

Voucher: *A G. Schulz 7830* (cited in Ramella 2008: 352).

##### *Pontederia cordata* L., Sp. Pl. 1: 288. 1753.

Syn.: *Narukila cordata* (L.) Nieuwl.; *Pontederia angustifolia* Pursh; *Pontederia cordata* L. forma *brasiliensis* Solms; *Pontederia cordata* L. var. *angustifolia* (Pursh) Torr.; *Pontederia cordata* L. var. *lanceolata* Griseb.; *Pontederia cordata* L. var. *lancifolia* (Muhl.) Torr.; *Pontederia lanceolata* Nutt.; *Pontederia lanceolata* Nutt. forma *brasiliensis* (Solms) Fernald; *Pontederia lancifolia* Muhl.; *Pontederia nymphaeifolia* Kunth; *Pontederia rotundifolia* L.f. var. *nymphaeifolia* (Kunth) Solms; *Unisema cordata* (L.) Farw.; *Unisema media* Raf.

Herb. In swamps.

Departmental distribution in Paraguay: Alto Paraguay, Amambay, Caaguazú, Canindeyú, Central, Concepción, Cordillera, Guairá, Itapúa, Misiones, Ñeembucú, Paraguarí, Presidente Hayes, San Pedro.

Voucher: *M. Peña-Chocarro et al. 2238* (BM, CTES, FCQ, G, MO, SI).

##### *Pontederia rotundifolia* L.f., Suppl. Pl. 192. 1781 [1782].

Syn.: *Pontederia cordifolia* Mart.; *Pontederia eriantha* Miq.; *Pontederia renniformis* Larrañaga; *Reussia grazielae* Machado; *Reussia rotundifolia* (L.f.) A.Cast.

Herb. In swamps.

Departmental distribution in Paraguay: Central, Concepción, Cordillera, Ñeembucú, Presidente Hayes.

Voucher: *M. Peña-Chocarro et al. 2359* (BM, CTES, FCQ, G, MO, SI).

##### ►*Pontederia subovata* (Seub.) Lowden, Rhodora 75: 478. 1973.

Syn.: *Eichhornia subovata* Seub.; *Reussia subovata* (Seub.) Solms

Aquatic herb.

Departmental distribution in Paraguay: Alto Paraguay, Central, Concepción, Ñeembucú, Presidente Hayes.

Voucher: *M.A. Walter 2* (BM).

#### PORTULACACEAE

##### ►*Portulaca cryptopetala* Speg., Anales Soc. Ci. Argent. 82: 217. 1916.

Syn.: *Portulaca cryptopetala* Speg. forma *phenopetala* Speg.; *Portulaca cryptopetala* Speg. var. *diversifolia* (Poelln.) D.Legrand; *Portulaca cryptopetala* Speg. var. *legrandii* (Poelln.) D.Legrand; *Portulaca cryptopetala* Speg. var. *poellnitziana* (D.Legrand) D.Legrand; *Portulaca diversifolia* D.Legrand, nom. illeg.; *Portulaca diversifolia* var. *legrandii* (Poelln.) D.Legrand; *Portulaca legrandii* Poelln.; *Portulaca poellnitziana* D.Legrand

Herb.

Departmental distribution in Paraguay: Alto Paraguay, Ñeembucú, Paraguarí.

Voucher: *M. Peña-Chocarro et al. 1981A* (BM, FCQ).

##### ►*Portulaca umbraticola* Kunth, Nov. Gen. Sp. (quarto ed.) 6: 72. 1823.

Syn.: *Portulaca coronata* Small; *Portulaca denudata* Poelln.; *Portulaca lanceolata* Engelm.; *Portulaca plano*-*operculata* Kuntze

Herb.

Departmental distribution in Paraguay: Ñeembucú, Presidente Hayes.

Voucher: *J. De Egea et al. 641* (BM, CTES, FCQ, MO).

##### *Portulaca* sp.

Herb.

Voucher: *M. Peña-Chocarro et al. 1989* (FCQ).

#### RANUNCULACEAE

##### *Clematis montevidensis* Spreng., Syst. Veg., ed. 16 [Sprengel] 2: 667. 1825.

Syn.: *Clematis campestris* A.St.-Hil. var. *mendocina* (Phil.) Hauman & Irigoyen; *Clematis hilarii* Spreng.; *Clematis hilarii* Sprengvar. *guaranitica* A.St.-Hil. & Tul.; *Clematis hilarii* Sprengvar. *montevidensis* (Spreng.) Speg.; *Clematis hilarii* Sprengvar. *triloba* (A.St.-Hil.) Speg.; *Clematis triloba* A.St.-Hil., nom. illeg.

Climber.

Departmental distribution in Paraguay: Alto Paraná, Alto Paraguay, Amambay, Canindeyú, Central, Cordillera, Itapúa, Misiones, Ñeembucú, Paraguarí, Presidente Hayes.

Voucher: *Anon. s.n.* (US) (cited in Molero 1985: 12).

#### RHAMNACEAE

##### *Gouania latifolia Reissek, Fl. Bras. (Martius) 11(1): 103. 1861.

Syn.: *Gouania mollis* Reissek

Climber.

Departmental distribution in Paraguay: Alto Paraguay, Caazapá, Canindeyú, Central, Concepción, Cordillera, Guairá, Ñeembucú, Paraguarí.

Voucher: *L. Bernardi 20499* (G) (http://www.ville-ge.ch/musinfo/bd/cjb/fdp/).

##### *Ziziphus joazeiro* Mart., Reise Bras. (Spix & Mart.) 581. 1828.

Syn.: *Ziziphus cotinifolia* Reissek; *Ziziphus gardneri* Reissek; *Ziziphus guaranitica* Malme

Tree or shrub.

Departmental distribution in Paraguay: Alto Paraguay, Central, Concepción, Cordillera, Ñeembucú, San Pedro.

Voucher: *A. Reales 252* (G, LIL) (cited in Tortosa and Cusato 1991: 171).

#### RUBIACEAE

##### ►*Borreria brachystemonoides* Cham. & Schltdl., Linnaea 3: 314. 1828.

Herb or subshrub.

Departmental distribution in Paraguay: Caazapá, Central, Concepción, Itapúa, Ñeembucú.

Voucher: *M. Peña-Chocarro et al. 2262* (BM, CTES, FCQ, MO).

##### ►*Borreria eryngioides* Cham. & Schltdl., Linnaea 3: 316. 1828.

Syn.: *Bigelovia eryngioides* (Cham. & Schltdl.) Hook. & Arn.; *Borreria diffusa* DC.; *Borreria suaveolens* G.Mey. var. *tenera* auct. non K.Schum.; *Borreria tenera* auct. non DC.; *Spermacoce diffusa* auct. non Pohl ex DC.; *Spermacoce eryngioides* (Cham. & Schltdl.) Kuntze

Herb or subshrub.

Departmental distribution in Paraguay: Alto Paraguay, Caaguazú, Central, Concepción, Guairá, Misiones, Ñeembucú, Paraguarí, Presidente Hayes.

Voucher: *M. Peña-Chocarro et al. 2267* (BM, CTES, FCQ).

##### *Borreria verticillata (L.) G.Mey., Prim. Fl. Esseq. 83. 1818.

Syn.: *Borreria capitata* (Ruiz & Pav.) DC. forma *ferruginea* auct. non (A.St.-Hil.) Steyerm.; *Spermacoce verticillata* L.

Herb.

Departmental distribution in Paraguay: Caazapá, Canindeyú, Cordillera, Ñeembucú.

Voucher: *R. Spichiger et al. 5224* (G) (http://www.ville-ge.ch/musinfo/bd/cjb/fdp/).

##### *Cephalanthus glabratus (Spreng.) K.Schum., Fl. Bras. (Martius) 6(6): 128. 1888.

Syn.: *Buddleja glabrata* Spreng.; *Cephalanthus sarandi* Cham. & Schltdl., nom. superfl.

Shrub.

Departmental distribution in Paraguay: Alto Paraná, Caazapá, Canindeyú, Central, Concepción, Cordillera, Itapúa, Ñeembucú, Paraguarí, Presidente Hayes, San Pedro.

Voucher: *C. Vogt 151* (CTES, FACEN, FCQ).

##### ►*Chomelia obtusa* Cham. & Schltdl., Linnaea 4: 185. 1829.

Syn.: *Anisomeris obtusa* (Cham. & Schltdl.) K.Schum.; *Anisomeris obtusa* (Cham. & Schltdl.) K.Schum. var. *inermis* Chodat & Hassl.

Tree or shrub.

Departmental distribution in Paraguay: Caaguazú, Caazapá, Canindeyú, Central, Concepción, Cordillera, Itapúa, Ñeembucú, Paraguarí.

Voucher: *M. Vera 227* (BM, CTES, FCQ, G, MO, SI).

##### *Diodia kuntzei* K.Schum., Fl. Bras. (Martius) 6(6): 15. 1888.

Herb.

Departmental distribution in Paraguay: Central, Concepción, Cordillera, Ñeembucú, San Pedro.

Voucher: *J. De Egea et al. 689* (BM, FCQ, MO).

##### ►*Galianthe brasiliensis* (Spreng.) E.L.Cabral & Bacigalupo, Ann. Missouri Bot. Gard. 84: 861. 1997.

Syn.: *Diodia anthospermoides* Cham. & Schltdl.; *Diodia brasiliensis* Spreng.; *Diodia brasiliensis* Spreng. var. *microphylla* (Cham. & Schltdl.) Standl.; *Diodia polymorpha* Cham. & Schltdl.; *Diodia polymorpha* Cham. & Schltdl.var. *macrophylla* Cham. & Schltdl.; *Diodia polymorpha* Cham. & Schltdl. var. *microphylla* Cham. & Schltdl.

Subshrub.

Departmental distribution in Paraguay: Alto Paraná, Itapúa, Ñeembucú.

Voucher: *S. Keel & L. Spinzi 1466* (FCQ).

##### ►*Galium hypocarpium* (L.) Endl.ex Griseb., Fl. Brit. W. I. 351. 1861.

Syn.: *Galium relbun* (Cham. & Schltdl.) Clos; *Relbunium croceum* (Ruiz & Pav.) K.Schum.; *Relbunium hypocarpium* (L.) Hemsl.; *Relbunium hypocarpium* (L.) Hemsl. subsp. *fluminense* (Vell.) Ehrend.; *Relbunium hypocarpium* (L.) Hemsl. subsp. *grandifolium* Ehrend.; *Relbunium hypocarpium* (L.) Hemsl. subsp. *nitidum* (Kunth) Ehrend.; *Relbunium ovale* (Ruiz & Pav.) K.Schum.; *Relbunium wettsteinii* Zahlbr.; *Rubia relbun* Cham. & Schltdl.; *Valantia hypocarpia* L.

Herb.

Departmental distribution in Paraguay: Alto Paraná, Caazapá, Central, Cordillera, Guairá, Ñeembucú, Paraguarí.

Voucher: *M. Peña-Chocarro et al. 2345* (BM, CTES, FCQ, G, MO, SI).

##### *Galium megapotamicum* Spreng., Syst. Nat., ed. 4 [Sprengel] 4(2): 39. 1827.

Syn.: *Relbunium megapotamicum* (Spreng.) Ehrend.; *Relbunium megapotamicum* (Spreng.) Ehrend. subsp. *camporum* Ehrend.

Herb or subshrub.

Departmental distribution in Paraguay: Alto Paraná, Amambay, Caaguazú, Caazapá, Canindeyú, Cordillera, Guairá, Ñeembucú, Paraguarí, San Pedro.

Voucher: *T. Rojas 11061* (A) (cited in Dempster 1990: 336).

##### ►*Galium richardianum* (Gillies ex Hook. & Arn.) Endl. ex Walp., Repert. Bot. Syst. 2: 459. 1843.

Syn.: *Galium chaetophorum* Griseb.; *Galium chaetophorum* Griseb. var. *prostratum* Kuntze; *Galium chaetophorum* Griseb. var. *strictum* Kuntze; *Galium ciliatum* Ruiz & Pav. var. *pusillum* (Gillies ex Hook. & Arn.) Kuntze; *Galium ciliatum* Ruiz & Pav. var. *richardianum* (Gillies ex Hook. & Arn.) Kuntze; *Galium patagonicum* Kuntze; *Galium pusillum* auct. non L.; *Relbunium chaetophorum* (Griseb.) K.Schum.; *Relbunium equinocarpum* Hassl.; *Relbunium patagonicum* (Kuntze) K.Schum.; *Relbunium pusillum* (Gillies ex Hook. & Arn.) K.Schum.; *Relbunium richardianum* (Gillies ex Hook. & Arn.) Hicken; *Relbunium richardianum* (Gillies ex Hook. & Arn.) Hickensubsp. *patagonicum* (Kuntze) Ehrend.; *Rubia richardiana* Gillies ex Hook. & Arn.

Herb.

Departmental distribution in Paraguay: Caaguazú, Ñeembucú.

Voucher: *M. Peña-Chocarro et al. 2255* (BM, CTES, FCQ).

##### ►*Guettarda uruguensis* Cham. & Schltdl., Linnaea 4: 183. 1829.

Shrub.

Departmental distribution in Paraguay: Alto Paraná, Alto Paraguay, Caazapá, Guairá, Itapúa, Misiones, Ñeembucú, Paraguarí, Presidente Hayes.

Voucher: *M. Peña-Chocarro et al. 2304* (BM, CTES, FCQ, G, MO).

##### *Hamelia patens* Jacq., Enum. Syst. Pl. 16. 1760.

Syn.: *Hamelia erecta* Jacq.

Shrub.

Departmental distribution in Paraguay: Alto Paraná, Amambay, Caazapá, Canindeyú, Concepción, Cordillera, Guairá, Ñeembucú, Paraguarí, San Pedro.

Voucher: *R. Spichiger et al. 5302* (G) (http://www.ville-ge.ch/musinfo/bd/cjb/fdp/).

##### *Machaonia brasiliensis (Hoffmanns. ex Humb.) Cham. & Schltdl., Linnaea 4: 2. 1829.

Syn.: *Cinchona brasiliensis* Hoffmanns. ex Humb.

Shrub.

Departmental distribution in Paraguay: Alto Paraguay, Amambay, Central, Itapúa, Ñeembucú, Presidente Hayes.

Voucher: *J. De Egea et al. 720* (BM, CTES, FCQ, G, MO).

##### *Oldenlandia salzmannii* (DC.) Benth. & Hook. ex B.D.Jacks., Index Kew. 2: 58. 1893.

Syn.: *Anotis salzmannii* DC.; *Hedyotis inconspicua* F.Phil.; *Hedyotis muscosa* A.St.-Hil.; *Hedyotis perpusilla* Hook. & Arn.; *Hedyotis pilosa* Poepp.; *Hedyotis salzmannii* (DC.) Steud.; *Hedyotis theesifolia* A.St.-Hil.; *Hedyotis uniflora* DC.; *Oldenlandia theesifolia* (A.St.-Hil.) K.Schum.; *Oldenlandia uniflora* Ruiz & Pav.

Herb.

Departmental distribution in Paraguay: Alto Paraná, Caaguazú, Caazapá, Central, Cordillera, Misiones, Ñeembucú, Paraguarí.

Voucher: *M.A. Walter 8* (BM).

##### *Psychotria carthagenensis Jacq., Enum. Syst. Pl. 16. 1760.

Syn.: *Mapouria catharinensis* Müll.Arg.; *Psychotria foveolata* Ruiz & Pav.

Shrub.

Departmental distribution in Paraguay: Alto Paraná, Amambay, Caaguazú, Caazapá, Canindeyú, Central, Concepción, Cordillera, Guairá, Itapúa, Misiones, Ñeembucú, Paraguarí, Presidente Hayes, San Pedro.

Voucher: *L. Bernardi 18474* (BM, G, MO).

##### *Randia armata (Sw.) DC., Prodr. (DC.) 4: 387. 1830.

Syn.: *Basanacantha spinosa* (Jacq.) K.Schum.; *Gardenia armata* Sw.; *Mussaenda spinosa* Jacq.; *Randia spinosa* (Jacq.) H.Karst., nom. illeg.

Shrub.

Departmental distribution in Paraguay: Alto Paraná, Alto Paraguay, Amambay, Canindeyú, Central, Concepción, Cordillera, Guairá, Misiones, Ñeembucú, Paraguarí, Presidente Hayes, San Pedro.

Voucher: *J. De Egea & R. Elsam 801* (BM, CTES, FCQ, G, MO).

##### *Richardia brasiliensis Gomes, Mem. Ipecac. Bras. 31. 1801.

Syn.: *Richardia pilosa* auct. non Ruiz & Pav.; *Richardia rosea* (A.St.-Hil.) Schult.; *Richardia scabra* auct. non L.; *Richardsonia brasiliensis* (B.A.Gomes) Hayne; *Richardsonia brasiliensis* (Gomes) Haynevar. *dubia* Beauverd & Felipp.; *Richardsonia rosea* A.St.-Hil.; *Richardsonia scabra* auct. non (L.) A.St.-Hil.

Herb.

Departmental distribution in Paraguay: Alto Paraguay, Caaguazú, Caazapá, Canindeyú, Central, Concepción, Cordillera, Guairá, Itapúa, Ñeembucú, Paraguarí, San Pedro.

Voucher: *R. Spichiger et al. 5249* (G) (http://www.ville-ge.ch/musinfo/bd/cjb/fdp/).

##### ►*Richardia stellaris* (Cham. & Schltdl.) Steud., Nomencl. Bot. (ed. 2) 459. 1841.

Syn.: *Richardsonia astroites* K.Schum.; *Richardsonia stellaris* Cham. & Schltdl.; *Richardsonia stellaris* Cham. & Schltdl.forma *linearifolia* Chodat & Hassl.; *Richardsonia stellaris* Cham. & Schltdl. forma *robusta* Chodat & Hassl.

Herb.

Departmental distribution in Paraguay: Central, Concepción, Cordillera, Ñeembucú, Paraguarí.

Voucher: *M. Peña-Chocarro et al. 2263* (FCQ).

##### ►*Spermacoce glabra* Michx., Fl. Bor.-Amer. 1: 82. 1803.

Syn.: *Diodia glabra* (Michx.) Pers.; *Spermacoceodes glabrum* (Michx.) Kuntze; *Spermacoceodes glabrum* (Michx.) Kuntzevar. *rectum* Bacigalupo

Herb.

Departmental distribution in Paraguay: Alto Paraguay, Ñeembucú, Paraguarí, Presidente Hayes.

Voucher: *S. Keel & L. Spinzi v-168* (FCQ).

##### *Spermacoce schumannii* (Standl. ex Bacigalupo) P.G.Delprete, Fl. Ilustr. Catarin. Rubiaceas 2: 754. 2005.

Syn.: *Borreria flavovirens* Bacigalupo & E.L.Cabral, nom. illeg.; *Diodia gymnocephala* (DC.) K.Schum.; *Diodia schumannii* Standl. ex Bacigalupo, nom. nud.

Subshrub.

Departmental distribution in Paraguay: Alto Paraná, Alto Paraguay, Caaguazú, Cordillera, Guairá, Itapúa, Misiones, Ñeembucú, Paraguarí, San Pedro.

Voucher: *S.Keel & L. Spinzi v-162* (FCQ).

#### RUTACEAE

##### *Pilocarpus pennatifolius Lem., Jard. Fleur. 3: t. 263. 1852.

Syn.: *Pilocarpus pennatifolius* Lem. forma *brasiliensis* Hassl.; *Pilocarpus pennatifolius* Lem. forma *gracilis* (Chodat & Hassl.) Hassl.; *Pilocarpus pennatifolius* Lem. forma *intermedius* Hassl.; *Pilocarpus pennatifolius* Lem. forma *latifoliolatus* Hassl.; *Pilocarpus pennatifolius* Lem. forma *paraguariensis* Hassl.; *Pilocarpus pennatifolius* Lem. forma *typicus* Hassl., nom. illeg.; *Pilocarpus pennatifolius* Lem. var. *selloanus* (Engl.) Hassl.; *Pilocarpus selloanus* Engl.; *Pilocarpus selloanus* Engl. forma *brevipedicellata* Chodat & Hassl.; *Pilocarpus selloanus* Engl. var. *gracilis* Chodat & Hassl.

Tree.

Departmental distribution in Paraguay: Alto Paraná, Amambay, Caaguazú, Caazapá, Canindeyú, Central, Concepción, Cordillera, Guairá, Itapúa, Ñeembucú, Paraguarí, Presidente Hayes, San Pedro.

Voucher: *C. Vogt 155* (CTES, FCQ).

##### ►*Zanthoxylum fagara* (L.) Sarg., Gard. & Forest 3(112): 186. 1890.

Syn.: *Fagara hyemalis* (A.St.-Hil.) Engl.; *Fagara nigrescens* R.E.Fr.; *Fagara pterota* L.; *Fagara pterota* L. var. *guaranitica* Chodat & Hassl.; *Schinus fagara* L.; *Zanthoxylum hyemale* A.St.-Hil.; *Zanthoxylum pterota* (L.) Kunth; *Zanthoxylum pterota* (L.) Kunthvar. *guaraniticum* (Chodat & Hassl.) P.G.Waterman

Tree.

Departmental distribution in Paraguay: Alto Paraná, Amambay, Caaguazú, Caazapá, Canindeyú, Central, Concepción, Cordillera, Guairá, Itapúa, Misiones, Ñeembucú, Paraguarí, San Pedro.

Voucher: *J. De Egea et al. 731* (BM, CTES, FCQ, MO).

##### *Zanthoxylum petiolare* A.St.-Hil. & Tul., Ann. Sci. Nat., Bot., sér. 2, 17: 140. 1842.

Syn.: *Fagara hieronymi* Engl.; *Fagara naranjillo* (Griseb.) Engl.; *Fagara naranjillo* (Griseb.) Engl. var. *angustifolia* Lillo; *Fagara naranjillo* (Griseb.) Engl. var. *paraguariensis* (Chodat & Hassl.) Escal.; *Fagara niederleinii* Engl.; *Fagara paraguariensis* Chodat & Hassl.; *Fagara paraguariensis* Chodat & Hassl. forma *angustifolia* Chodat & Hassl.; *Fagara paraguariensis* Chodat & Hassl. forma *fruticosa* Chodat & Hassl.; *Fagara paraguariensis* Chodat & Hassl. forma *latifolia* Chodat & Hassl.; *Fagara rhoifolia* (Lam.) Engl. var. *niederleinii* (Engl.) Nájera; *Zanthoxylum naranjillo* Griseb.; *Zanthoxylum naranjillo* Griseb. var. *angustifolium* (Lillo) P.G.Waterman; *Zanthoxylum paraguariensis* (Chodat & Hassl.) Standl.

Tree.

Departmental distribution in Paraguay: Alto Paraná, Alto Paraguay, Amambay, Caaguazú, Caazapá, Canindeyú, Central, Concepción, Cordillera, Guairá, Itapúa, Misiones, Ñeembucú, Paraguarí, Presidente Hayes, San Pedro.

Voucher: *M. Peña-Chocarro et al. 2203* (BM, CTES, FCQ, MO).

##### ►*Zanthoxylum rigidum* Humb. & Bonpl. ex Willd., Sp. Pl., ed. 4 [Willdenow] 4(2): 756. 1806.

Syn.: *Fagara hassleriana* Chodat; *Fagara hassleriana* Chodatvar. *angustifolia* Chodat & Hassl.; *Fagara rigida* (Humb. & Bonpl. ex Willd.) Engl.; *Zanthoxylum hasslerianum* (Chodat) Pirani; *Zanthoxylum rigidum* Humb. & Bonpl. ex Willd. subsp. *hasslerianum* (Chodat) Reynel

Tree.

Departmental distribution in Paraguay: Alto Paraguay, Amambay, Concepción, Cordillera, Ñeembucú, Paraguarí, Presidente Hayes.

Voucher: *C. Vogt 153* (CTES, FCQ).

#### SALICACEAE

##### *Banara arguta* Briq., Annuaire Conserv. Jard. Bot. Genève 4: 223. 1900.

Syn.: *Banara brasiliensis* auct. non (Schott) Benth.; *Banara glandulosa* (Desv.) Speg.; *Banara guianensis* Aubl. var. *argentina* Lillo; *Banara guianensis* auct. non Aubl.; *Banara tomentosa* auct. non Clos

Tree or shrub.

Departmental distribution in Paraguay: Alto Paraguay, Amambay, Central, Concepción, Cordillera, Ñeembucú, Presidente Hayes.

Voucher: *A. Schinini 7684* (CTES) (cited in Soloaga et al. 2000: 13).

##### *Casearia sylvestris* Sw., Fl. Ind. Occid. 2: 752. 1798.

Tree or shrub.

Departmental distribution in Paraguay: Alto Paraná, Amambay, Caazapá, Canindeyú, Central, Concepción, Cordillera, Guairá, Itapúa, Misiones, Ñeembucú, Paraguarí, Presidente Hayes, San Pedro.

Voucher: *C. Vogt 159* (CTES, FACEN, FCQ).

##### *Prockia crucis* L., Syst. Nat., ed. 10. 2: 1074. 1759.

Syn.: *Prockia crucis* L. var. *septemnervia* (Spreng.) Sleumer; *Prockia glabra* Briq.; *Prockia grandiflora* Herzog; *Prockia hassleri* Briq.; *Prockia septemnervia* Spreng.; *Trilix crucis* (L.) Griseb.

Tree or shrub.

Departmental distribution in Paraguay: Alto Paraná, Amambay, Caaguazú, Canindeyú, Central, Concepción, Cordillera, Guairá, Ñeembucú, Paraguarí, San Pedro.

Voucher: *J. De Egea et al. 827* (BM).

##### ►*Salix humboldtiana* Willd., Sp. Pl., ed. 4 [Willdenow] 4(2): 657. 1806.

Syn.: *Salix chilensis* Molina var. *crispa* Stuck. ex Seckt; *Salix magellanica* Lam.

Tree.

Departmental distribution in Paraguay: Central, Ñeembucú, Paraguarí, San Pedro.

Voucher: *L. Bernardi 18458* (BM).

##### *Xylosma venosa* N.E.Br., Trans. & Proc. Bot. Soc. Edinburgh 20: 46. 1893.

Syn.: *Myroxylon salzmannii* auct. non (Clos) Kuntze; *Xylosma balansae* Briq.; *Xylosma paraguayensis* Briq.

Tree or shrub.

Departmental distribution in Paraguay: Alto Paraná, Alto Paraguay, Amambay, Caazapá, Canindeyú, Central, Concepción, Cordillera, Guairá, Itapúa, Misiones, Ñeembucú, Paraguarí, Presidente Hayes, San Pedro.

Voucher: *J. De Egea et al. 683* (BM, CTES, FCQ, G, MO, PY, UNR).

#### SANTALACEAE

##### ►*Phoradendron bathyoryctum* Eichler, Fl. Bras. (Martius) 5(2): 123. 1868.

Syn.: *Phoradendron balansae* Trel.; *Phoradendron balansae* Trel. forma *hassleri* Trel.; *Phoradendron balansae* Trel. forma *morongii* Trel.; *Phoradendron hieronymi* Trel.; *Phoradendron ulophyllum* Eichler

Shrub, parasite.

Departmental distribution in Paraguay: Central, Guairá, Ñeembucú, Paraguarí.

Voucher: *J. De Egea et al. 361* (BM, CTES, FCQ, G, MO).

##### ►*Phoradendron liga* (Gillies ex Hook. & Arn.) Eichler, Fl. Bras. (Martius) 5(2): 134m. 1868.

Syn.: *Viscum liga* Gillies ex Hook. & Arn.

Shrub, parasite.

Departmental distribution in Paraguay: Alto Paraguay, Central, Misiones, Ñeembucú, Paraguarí, Presidente Hayes, San Pedro.

Voucher: *J. De Egea et al. 362* (BM, CTES, FCQ, G, MO, PY, UNR).

#### SAPINDACEAE

##### ►*Cardiospermum grandiflorum* Sw., Prodr. (Swartz) 64. 1788.

Syn.: *Cardiospermum duarteanum* Cambess.

Climber.

Departmental distribution in Paraguay: Alto Paraná, Alto Paraguay, Amambay, Caaguazú, Caazapá, Canindeyú, Central, Concepción, Cordillera, Guairá, Itapúa, Ñeembucú, Paraguarí, San Pedro.

Voucher: *M. Peña-Chocarro et al. 2280* (BM, CTES, FCQ, G, MO, PY, SI, UNR).

##### ►*Cardiospermum halicacabum* L. var. *microcarpum* (Kunth) Blume, Rumphia 3: 185. 1847.

Syn.: *Cardiospermum microcarpum* Kunth

Climber.

Departmental distribution in Paraguay: Alto Paraguay, Amambay, Canindeyú, Central, Concepción, Cordillera, Guairá, Itapúa, Ñeembucú, Paraguarí, Presidente Hayes, San Pedro.

Voucher: *M. Peña-Chocarro et al. 2298* (BM, CTES, FCQ, G, MO, PY, SI, UNR).

##### ►*Cupania vernalis* Cambess., Fl. Bras. Merid. (quarto ed.) 1: 387. 1825.

Syn.: *Cupania uraguensis* Hook. & Arn.

Tree.

Departmental distribution in Paraguay: Alto Paraná, Amambay, Caaguazú, Caazapá, Canindeyú, Central, Cordillera, Guairá, Itapúa, Misiones, Ñeembucú, Paraguarí, San Pedro.

Voucher: *M. Peña-Chocarro et al. 2217* (BM, CTES, FCQ, G, MO, PY, SI, UNR).

##### *Diplokeleba floribunda* N.E.Br., Trans. & Proc. Bot. Soc. Edinburgh 20: 50. 1894.

Tree.

Departmental distribution in Paraguay: Alto Paraguay, Amambay, Central, Concepción, Cordillera, Guairá, Ñeembucú, Paraguarí, Presidente Hayes, San Pedro.

Voucher: *L. Bernardi 18429* (BM, G).

##### *Houssayanthus incanus* (Radlk.) Ferrucci, Candollea 41: 218. 1986.

Syn.: *Houssayanthus fiebrigii* (F.A.Barkley) Hunz.; *Serjania humifusa* Radlk.; *Serjania incana* Radlk.; *Serjania incana* Radlk. forma *glabriuscula* Radlk.; *Urvillea fiebrigii* F.A.Barkley

Climber.

Departmental distribution in Paraguay: Alto Paraguay, Central, Concepción, Cordillera, Ñeembucú, Presidente Hayes.

Voucher: *A. Reales 270* (cited in Ferrucci 1991: 47).

##### *Houssayanthus monogynus* (Schltdl.) Ferrucci, Candollea 42: 806. 1987.

Syn.: *Houssayanthus sparrei* (F.A.Barkley) Ferrucci; *Serjania monogyna* Schltdl.; *Urvillea sparrei* F.A.Barkley

Climber.

Departmental distribution in Paraguay: Central, Misiones, Ñeembucú, Paraguarí.

Voucher: *T. Rojas 12681* (cited in Ferrucci 1991: 48).

##### ►*Paullinia elegans* Cambess., Fl. Bras. Merid. (quarto ed.) 1: 370. 1825.

Syn.: *Serjania paniculata* auct. non Kunth

Climber.

Departmental distribution in Paraguay: Alto Paraná, Amambay, Caaguazú, Caazapá, Canindeyú, Central, Cordillera, Guairá, Itapúa, Misiones, Ñeembucú, Paraguarí, Presidente Hayes, San Pedro.

Voucher: *M. Peña-Chocarro et al. 2330* (BM, CTES, FCQ, G, MO, PY, SI).

##### *Paullinia pinnata* L., Sp. Pl. 1: 365. 1753.

Syn.: *Paullinia angusta* N.E.Br.

Climber.

Departmental distribution in Paraguay: Alto Paraná, Alto Paraguay, Canindeyú, Central, Concepción, Guairá, Misiones, Ñeembucú, Paraguarí, Presidente Hayes, San Pedro.

Voucher: *M. Peña-Chocarro et al. 2286* (BM, CTES, FCQ, G, MO, PY, SI).

##### *Serjania caracasana* (Jacq.) Willd., Sp. Pl., ed. 4 [Willdenow] 2(1): 465. 1799.

Syn.: *Paullinia caracasana* Jacq.; *Serjania caracasana* (Jacq.) Willd. forma *nitidula* Radlk.; *Serjania caracasana* (Jacq.) Willd. forma *puberula* Radlk.; *Serjania grandiflora* auct. non Cambess.; *Serjania platyptera* (Radlk.) F.A.Barkley

Climber.

Departmental distribution in Paraguay: Alto Paraguay, Amambay, Canindeyú, Central, Concepción, Cordillera, Ñeembucú, Paraguarí, Presidente Hayes, San Pedro.

Voucher: *de Pompéry s.n.* (cited in Ferrucci 1991: 78).

##### *Serjania perulacea* Radlk., Consp. Sect. Sp. Serj. 11. 1874.

Syn.: *Serjania meyeri* F.A.Barkley; *Serjania tristis* auct. non Radlk.

Climber.

Departmental distribution in Paraguay: Alto Paraguay, Amambay, Central, Concepción, Cordillera, Guairá, Itapúa, Misiones, Ñeembucú, Paraguarí, Presidente Hayes, San Pedro.

Voucher: *C. Vogt 136* (CTES, FACEN, FCQ).

#### SAPOTACEAE

##### *Chrysophyllum gonocarpum (Mart. & Eichler) Engl., Bot. Jahrb. Syst. 12(5): 523. 1890.

Syn.: *Chloroluma gonocarpa* (Mart. & Eichler) Baill. ex Aubrév.; *Chrysophyllum lucumifolium* Griseb.; *Chrysophyllum lucumifolium* Griseb. forma *obtusata* Chodat & Hassl.; *Chrysophyllum nemoralis* Rojas Acosta, nom. nud.; *Martiusella gonocarpa* (Mart. & Eichler) Pierre; *Sapota gonocarpa* Mart. & Eichler; *Sideroxylon bolivianum* Rusby; *Sideroxylon reticulatum* Britton

Tree.

Departmental distribution in Paraguay: Alto Paraná, Amambay, Caazapá, Canindeyú, Central, Concepción, Cordillera, Guairá, Itapúa, Ñeembucú, Paraguarí, San Pedro.

Voucher: *J. De Egea & R. Elsam 819* (BM, FCQ).

##### *Chrysophyllum marginatum* (Hook. & Arn.) Radlk., Actes Occasion Exposition Universelle Anvers Coincidence Exposition Int. Hort. 170. 1887.

Syn.: *Chrysophyllum ebenaceum* Mart.; *Chrysophyllum ebenaceum* Mart. var. *longifolium* Miq.; *Chrysophyllum grisebachii* (Hieron.) Mez; *Chrysophyllum martianum* A.DC.; *Chrysophyllum maytenoides* Mart.; *Chrysophyllum maytenoides* Mart. var. *tenue* Kuntze; *Chrysophyllum myrtifolium* Mart.; *Cynodendron marginatum* (Hook. & Arn.) Baehni; *Micropholis paraguayensis* Dubard; *Myrsine grisebachii* Hieron.; *Myrsine marginata* Hook. & Arn.; *Myrsine marginata* Hook. & Arn. var. *arborea* Griseb.; *Sideroxylon myrtifolium* (Mart.) Speg.

Tree or shrub.

Departmental distribution in Paraguay: Alto Paraná, Amambay, Caaguazú, Caazapá, Canindeyú, Central, Concepción, Cordillera, Guairá, Itapúa, Misiones, Ñeembucú, Paraguarí, San Pedro.

Voucher: *J. De Egea & R. Elsam 790* (BM, CTES, FCQ, G, MO).

##### *Pouteria gardneriana* (A.DC.) Radlk., Sitzungber. Math.-Phys. Cl. Königl. Bayer. Akad. Wiss. München 12: 333. 1882.

Syn.: *Labatia ostenii* Hassl.; *Labatia sapota-anguai* Rojas Acosta; *Lucuma gardneriana* A.DC.; *Pouteria suavis* Hemsl.

Tree.

Departmental distribution in Paraguay: Alto Paraguay, Concepción, Cordillera, Ñeembucú, Paraguarí.

Voucher: *L. Bernardi 18469* (BM).

##### *Pouteria glomerata* (Miq.) Radlk., Sitzungber. Math.-Phys. Cl. Königl. Bayer. Akad. Wiss. München 12: 333. 1882.

Syn.: *Labatia glomerata* (Miq.) Radlk., nom. illeg.; *Lucuma glomerata* Miq.; *Neolabatia glomerata* (Miq.) Aubrév.; *Richardella glomerata* (Miq.) Baehni

Tree.

Departmental distribution in Paraguay: Alto Paraná, Alto Paraguay, Amambay, Caaguazú, Caazapá, Canindeyú, Central, Concepción, Cordillera, Guairá, Itapúa, Misiones, Ñeembucú, Paraguarí, Presidente Hayes, San Pedro.

Voucher: *Mayer 15845* (K, MO, NSW) (cited in Pennington 1990: 420).

#### SIMAROUBACEAE

##### ►*Castela coccinea* Griseb., Abh. Königl. Ges. Wiss. Göttingen 19: 107. 1874.

Syn.: *Ximenia americana* L. var. *pubens* Griseb.

Shrub.

Departmental distribution in Paraguay: Alto Paraguay, Concepción, Guairá, Ñeembucú, Presidente Hayes.

Voucher: *J. De Egea & M. Vera 471* (BM, CTES, FCQ, G, MO).

##### *Picramnia sellowii* Planch., London J. Bot. 5: 578. 1846.

Syn.: *Picramnia pendula* Tul.; *Picramnia sellowii* Planch. forma *glabrescens* Chodat & Hassl.; *Picramnia sellowii* Planch. forma *hirsuta* Chodat & Hassl.; *Picramnia sellowii* Planch. forma *intermedia* Chodat & Hassl.; *Picramnia sellowii* Planch. var. *latifolia* Engl.

Tree or shrub.

Departmental distribution in Paraguay: Alto Paraná, Alto Paraguay, Amambay, Caaguazú, Caazapá, Central, Concepción, Cordillera, Guairá, Itapúa, Misiones, Ñeembucú, Paraguarí, Presidente Hayes, San Pedro.

Voucher: *T. Meyer 15885* (cited in Rubens Pirani 1987: 17).

#### SMILACACEAE

##### ►*Smilax campestris* Griseb., Fl. Bras. (Martius) 3(1): 15. 1842.

Syn.: *Smilax campestris* Griseb. var. *marginulata* (Mart. ex Griseb.) A.DC.; *Smilax campestris* Griseb. var. *rubiginosa* (Griseb.) A.DC.; *Smilax marginulata* Mart. ex Griseb.; *Smilax montana* Griseb.; *Smilax rubiginosa* Griseb.; *Smilax scalaris* Griseb.; *Smilax viminea* Griseb.

Climber.

Departmental distribution in Paraguay: Caaguazú, Caazapá, Canindeyú, Central, Cordillera, Guairá, Itapúa, Ñeembucú, Paraguarí.

Voucher: *R. García et al. 03* (FCQ, UNP).

##### ►*Smilax cognata* Kunth, Enum. Pl. [Kunth] 5: 175. 1850.

Syn.: *Smilax montevidensis* Hort. ex Steud., nom. nud.

Climber.

Departmental distribution in Paraguay: Caazapá, Cordillera, Guairá, Itapúa, Ñeembucú.

Voucher: *J. De Egea & M. Vera 476* (BM, CTES, FCQ, G, MO).

#### SOLANACEAE

##### *Bouchetia anomala* (Miers) Britton & Rusby, Trans. New York Acad. Sci. 7: 12. 1887.

Syn.: *Bouchetia anomala* (Miers) Britton & Rusby subsp. *staticifolia* (Sendtn.) Loes.; *Bouchetia anomala* (Miers) Loes., nom. illeg.; *Bouchetia erecta* auct. non Dunal; *Nicotiana staticifolia* (Sendtn.) Kuntze; *Nierembergia anomala* Miers; *Nierembergia staticifolia* Sendtn.; *Salpiglossis anomala* (Miers) D’Arcy

Herb.

Departmental distribution in Paraguay: Ñeembucú.

Voucher: *J. De Egea et al. 640* (BM, CTES, FCQ, MO).

##### *Brunfelsia australis* Benth., Prodr. (DC.) 10: 200. 1846.

Syn.: *Brunfelsia hopeana* (Hook.) Benth. var. *australis* (Benth.) J.A.Schmidt.; *Brunfelsia paraguayensis* Chodat; *Brunfelsia uniflora* (Pohl) D.Don forma *intermedia* Hassl.; *Brunfelsia uniflora* (Pohl) D.Don forma *obovatifolia* Hassl.; *Franciscea australis* (Benth.) Miers

Tree or shrub.

Departmental distribution in Paraguay: Amambay, Caazapá, Canindeyú, Central, Cordillera, Guairá, Ñeembucú, Paraguarí, Presidente Hayes, San Pedro.

Voucher: *M. Peña-Chocarro et al. 2358* (BM, CTES, FCQ, G, MO).

##### *Brunfelsia uniflora (Pohl.) D.Don, Edinburgh New Philos. J. 86. July. 1829.

Syn.: *Brunfelsia hopeana* (Hook.) Benth.; *Brunfelsia hopeana* (Hook.) Benth. var. *pubescens* Benth.; *Brunfelsia uniflora* (Pohl) D.Don var. *pubescens* (Benth.) R.E.D.Baker & N.W.Simmonds; *Franciscea hopeana* Hook.; *Franciscea uniflora* Pohl

Tree or shrub.

Departmental distribution in Paraguay: Cordillera, ñeembucú, Paraguarí, San Pedro

Voucher: *R. Spichiger et al. 5345* (G) (http://www.ville-ge.ch/musinfo/bd/cjb/fdp/).

Note: Determination of these specimens is not certain; they could represent abberant individuals of *Brunfelsia australis*.

##### ►*Capsicum chacoense* Hunz., Darwiniana 9: 228. 1950.

Syn.: *Capsicum chacoense* Hunz. var. *tomentosum* Hunz.

Shrub.

Departmental distribution in Paraguay: Alto Paraguay, Ñeembucú, Presidente Hayes.

Voucher: *M. Peña-Chocarro et al. 2311* (BM, FCQ).

##### ►*Cestrum laevigatum* Schltdl., Linnaea 7: 59. 1832.

Syn.: *Cestrum laevigatum* Schltdl. var. *evolutum* Schltdl.; *Cestrum laevigatum* Schltdl. var. *paraguayense* Francey; *Cestrum laevigatum* Schltdl. var. *pauperculum* Francey

Tree or shrub.

Departmental distribution in Paraguay: Caazapá, Guairá, Ñeembucú.

Voucher: *C. Vogt 130* (FCQ).

##### ►*Cestrum strigilatum* Ruiz & Pav., Fl. Peruv. [Ruiz & Pavon] 2: 29. 1799.

Syn.: *Cestrum calycinum* Kunth; *Cestrum calycinum* Kunthvar. *tenuiflorum* Francey; *Cestrum strigilatum* Ruiz & Pav. var. *calycinum* (Kunth) Kuntze; *Cestrum strigilatum* Ruiz & Pav. var. *laxiflorum* Kuntze; *Cestrum viridiflorum* Hook.

Shrub.

Departmental distribution in Paraguay: Alto Paraná, Amambay, Caazapá, Canindeyú, Central, Cordillera, Guairá, Ñeembucú, Paraguarí, Presidente Hayes.

Voucher: *L. Bernardi 18506* (BM).

##### ●*Datura ferox* L., Demonstr. Pl. 6. 1753.

Herb.

Departmental distribution in Paraguay: Ñeembucú.

Voucher: *L. Bernardi 18508* (MO) (http://www.tropicos.org/).

##### *Lycium morongii Britton, Ann. New York Acad. Sci. 7: 180. 1893.

Syn.: *Lycium morongii* Britton forma *parvifolium* C.L.Hitchc.; *Lycium morongii* Britton var. *indutum* Hassl., pro parte; *Lycium morongii* Britton var. *parvifolium* (C.L.Hitchc.) F.A.Barkley; *Lycium morongii* Britton var. *typicum* Hassl., nom. illeg.

Tree or shrub.

Departmental distribution in Paraguay: Alto Paraguay, Central, Concepción, Ñeembucú, Paraguarí, Presidente Hayes.

Voucher: *L. Bernardi 20483* (BM).

##### *Nicotiana glauca Graham, Edinburgh New Philos. J. 5: 175. 1828.

Syn.: *Acnistus virgatus* Griseb.; *Nicotiana glauca* Graham forma *lateritia* Lillo; *Nicotiana glauca* var. *decurrens* Comes; *Nicotiana glauca* Graham var. *grandiflora* Comes; *Nicotiana glauca* Graham var. *typica* Millán, nom. illeg.; *Nicotidendron glauca* (Graham) Griseb.

Shrub or subshrub.

Departmental distribution in Paraguay: Alto Paraguay, Cordillera, Ñeembucú, Paraguarí.

Voucher: *A.R. Duré 63* (MO) (http://www.tropicos.org/).

##### ►*Petunia inflata* R.E.Fr., Kongl. Svenska Vetensk. Acad. Handl. 34(5): 35. 1911.

Syn.: *Petunia integrifolia* (Hook.) Schinz & Thell. subsp. *inflata* (R.E.Fr.) Wijsman; *Stimoryne integrifolia* (Hook.) Wijsman subsp. *inflata* (R.E.Fr.) Wijsman

Herb.

Departmental distribution in Paraguay: Central, Cordillera, Itapúa, Ñeembucú.

Voucher: *M. Peña-Chocarro et al. 2245* (BM, CTES, FCQ, G, MO, SI).

##### *Salpichroa origanifolia (Lam.) Baill., Hist. Pl. (Baillon) 9: 288. 1888.

Syn.: *Atropa origanifolia* (Lam.) Desf.; *Atropa rhomboidea* Hook.; *Jaborosa montevidensis* Casar.; *Physalis origanifolia* Lam.; *Salpichroa rhomboidea* (Hook.) Miers; *Salpichroa rhomboidea* (Hook.) Miersvar. *divaricata* Miers; *Salpichroa rhomboidea* (Hook.) Miersvar. *mollis* Dammer; *Salpichroa rhomboidea* (Hook.) Miersvar. *pubescens* Miers; *Salpichroa rhomboidea* (Hook.) Miersvar. *mollis* Dammer

Herb or subshrub.

Departmental distribution in Paraguay: Amambay, Central, Ñeembucú, Paraguarí.

Voucher: *L. Bernardi 20486* (MO) (http://www.tropicos.org/).

##### *Solanum americanum* Mill., Gard. Dict., ed. 8. Solanum no. 5. 1768.

Syn.: *Solanum adventitium* Polg.; *Solanum americanum* Mill. var. *nodiflorum* (Jacq.) Edmonds; *Solanum curtipes* Bitter; *Solanum nigrum* L. var. *americanum* (Mill.) O.E.Schulz.; *Solanum nodiflorum* Jacq.; *Solanum nodiflorum* Jacq. var. *acuminatum* Chodat; *Solanum nodiflorum* Jacq. var. *microphyllum* Hassl.; *Solanum nodiflorum* Jacq. var. *sapucayense* Chodat; *Solanum oleraceum* Vell., nom. illeg.; *Solanum sciaphilum* Bitter

Herb or subshrub.

Departmental distribution in Paraguay: Alto Paraná, Alto Paraguay, Amambay, Caazapá, Canindeyú, Central, Cordillera, Guairá, Itapúa, Ñeembucú, Paraguarí, Presidente Hayes.

Voucher: *R. Spichiger et al. 5257* (G) (http://www.ville-ge.ch/musinfo/bd/cjb/fdp/).

##### *Solanum caavurana* Vell., Fl. Flumin. 86. 1829.

Syn.: *Solanum caavurana* Vell. forma *angustifolia* Chodat & Hassl.; *Solanum caavurana* Vell. forma *pauciflora* Chodat; *Solanum megalocarpon* C.V.Morton

Shrub or subshrub.

Departmental distribution in Paraguay: Alto Paraná, Amambay, Caaguazú, Canindeyú, Central, Concepción, Cordillera, Guairá, Itapúa, Misiones, Ñeembucú, Paraguarí, Presidente Hayes, San Pedro.

Voucher: *M. Peña-Chocarro et al. 2297* (BM, CTES, FCQ, G, MO, PY, SI).

##### ●*Solanum capsicoides* All., Mélanges Philos. Math. Roy. Soc. Turin 5: 64. 1773.

Syn.: *Solanum arrebenta* Vell.; *Solanum ciliare* Willd.; *Solanum ciliatum* Lam.; *Solanum sinuatifolium* Vell.; *Solanum sphaerocarpum* Moric.

Shrub.

Departmental distribution in Paraguay: Ñeembucú.

Voucher: *L. Bernardi 18428* (MO) (http://www.tropicos.org/).

##### ►*Solanum chenopodioides* Lam., Tab. Encycl. 2: 18. 1794.

Syn.: *Solanum aguaraquiya* Piso ex Sendtn.; *Solanum chenopodifolium* Dunal; *Solanum gracile* Dunal, nom. illeg.; *Solanum gracile* Dunal var. *microphyllum* Dunal; *Solanum gracilius* Herter; *Solanum isabellii* Dunal; *Solanum nigrum* L. var. *aguaraquiya* (Sendtn.) Reiche, pro parte; *Solanum nigrum* L. var. *frutescens* auct. non A.Br.; *Solanum ottonis* Hyl.; *Solanum pterocaulon* Dunal var. *aguaraquiya* (Piso ex Sendtn.) Dunal; *Solanum sublobatum* Willd.; *Solanum subspatulatum* Sendtn.; *Solanum vile* Bitter

Herb.

Departmental distribution in Paraguay: Ñeembucú, Presidente Hayes.

Voucher: *J. De Egea & R. Elsam 807* (BM, CTES, FCQ, G, MO, SI).

##### *Solanum glaucophyllum* Desf., Tab. École Bot., ed. 3, 3: 396. 1829.

Syn.: *Solanum glaucescens* Baclé ex Dunal, nom. nud.; *Solanum glaucum* Bertol.; *Solanum malacoxylon* Sendtn.; *Solanum malacoxylon* Sendtn. forma *albo-marginatum* (Chodat) Hassl.; *Solanum malacoxylon* Sendtn. forma *vulgare* Hassl.; *Solanum malacoxylon* Sendtn. var. *albo-marginatum* Chodat; *Solanum malacoxylon* Sendtn. var. *angustissimum* Kuntze; *Solanum malacoxylon* Sendtn. var. *genuinum* Hassl., nom. illeg.; *Solanum malacoxylon* Sendtn. var. *latifolium* Kuntze; *Solanum malacoxylon* Sendtn. var. *subvirescens* Hassl.; *Solanum melanoxylon* Sendtn.; *Solanum pulverulentum* auct. non Pers.

Shrub.

Departmental distribution in Paraguay: Alto Paraguay, Central, Concepción, Cordillera, Misiones, Ñeembucú, Paraguarí, Presidente Hayes, San Pedro.

Voucher: *L. Bernardi 18517* (BM).

##### *Solanum granuloso-leprosum* Dunal, Prodr. (DC.) 13(1): 115. 1852.

Syn.: *Solanum auriculatum* auct. non Aiton; *Solanum verbascifolium* auct. non L.; *Solanum verbascifolium* L. forma *eupulverulentum* Hassl.

Tree or shrub.

Departmental distribution in Paraguay: Alto Paraná, Amambay, Caazapá, Canindeyú, Central, Concepción, Cordillera, Guairá, Ñeembucú, Paraguarí, San Pedro.

Voucher: *J. De Egea & R. Elsam 798* (BM, CTES, FCQ, MO).

##### *Solanum heleonastes* S.Knapp, Ann. Missouri Bot. Gard. 72: 560. 1985.

Tree or shrub.

Departmental distribution in Paraguay: Caazapá, Ñeembucú.

Voucher: *S. Keel & L. Spinzi 1479* (FCQ).

##### *Solanum palinacanthum Dunal, Prodr. (DC.) 13(1): 245. 1852.

Syn.: *Solanum chloroleucum* Dunal; *Solanum jubeba* Vell.; *Solanum macronema* Sendtn.; *Solanum manoelii* Moric.; *Solanum paniculatum* L. forma *canescens* Hassl.; *Solanum paniculatum* L. forma *flavescens* Hassl.; *Solanum paniculatum* L. forma *repandum* Hassl.; *Solanum paniculatum* L. subsp. *pseudoauriculatum* (Chodat & Hassl.) Hassl.; *Solanum paniculatum* L. var. *ellipticum* Chodat; *Solanum paniculatum* L. var. *integrifolium* Dunal; *Solanum pseudoauriculatum* Chodat & Hassl.

Shrub.

Departmental distribution in Paraguay: Alto Paraguay, Amambay, Caazapá, Canindeyú, Central, Concepción, Itapúa, Misiones, Ñeembucú, San Pedro.

Voucher: *L. Bernardi 18490* (BM).

##### *Solanum pseudocapsicum* L., Sp. Pl. 184. 1753.

Syn.: *Solanum capsicastrum* Link ex Schauer; *Solanum capsicastrum* Link ex Schauervar. *caaguazuense* Chodat; *Solanum diflorum* Vell.; *Solanum diflorum* Vell. var. *angustifolium* Kuntze; *Solanum diflorum* Vell. var. *pulverulentum* Chodat; *Solanum ipecacuanha* Chodat; *Solanum ipecacuanha* Chodatvar. *calvescens* Chodat; *Solanum ipecacuanha* Chodatvar. *obovata* Chodat; *Solanum pseudocapsicum* L. forma *calvescens* (Chodat) Hassl.; *Solanum pseudocapsicum* L. forma *pilosum* Kuntze; *Solanum pseudocapsicum* L. forma *pilosulum* Hassl.; *Solanum pseudocapsicum* L. subsp. *diflorum* (Vell.) Hassl.; *Solanum pseudocapsicum* L. var. *ambiguum* Hassl.; *Solanum pseudocapsicum* L. var. *hygrophilum* (Schltdl.) Hassl.; *Solanum pseudocapsicum* L. var. *normale* Kuntze, nom. illeg.; *Solanum pseudocapsicum* L. var. *parvifolium* Kuntze; *Solanum pseudocapsicum* L. var. *sendtnerianum* Hassl.; *Solanum tucumanense* Griseb.

Subshrub.

Departmental distribution in Paraguay: Alto Paraguay, Amambay, Caaguazú, Caazapá, Canindeyú, Central, Cordillera, Guairá, Misiones, Ñeembucú, Presidente Hayes, San Pedro.

Voucher: Cited in Zuloaga et al. 2008: 3041.

##### *Solanum robustum* H.L.Wendl., Flora 27: 784. 1844.

Syn.: *Solanum alatum* Seem. & J.A.Schmidt, nom. illeg.; *Solanum concepcionis* Chodat & Hassl.; *Solanum concepcionis* Chodat & Hassl. var. *robustius* Chodat; *Solanum concepcionis* Chodat & Hassl. var. *typicum* Chodat, nom. illeg.; *Solanum robustum* H.L.Wendl. forma *decurrens* Hassl.; *Solanum robustum* H.L.Wendl. forma *rupestre* Hassl.; *Solanum robustum* H.L.Wendl. forma *typicum* Hassl., nom. illeg.; *Solanum robustum* H.L.Wendl. var. *concepcionis* (Chodat & Hassl.) Hassl.; *Solanum robustum* H.L.Wendl. var. *decurrens* Hassl.; *Solanum robustum* H.L.Wendl. var. *laxepilosum* Hassl.

Tree or shrub.

Departmental distribution in Paraguay: Alto Paraná, Central, Cordillera, Guairá, Itapúa, Misiones, Ñeembucú, Paraguarí, Presidente Hayes, San Pedro.

Voucher: *J. De Egea & R. Elsam 799* (BM, FCQ).

##### *Solanum sisymbriifolium* Lam., Tab. Encycl. 2: 25. 1794.

Syn.: *Solanum balbisii* Dunal, nom. illeg.; *Solanum balbisii* Dunalvar. *bipinnata* Hook.; *Solanum balbisii* Dunalvar. *purpureum* Hook.; *Solanum bipinnatifidum* Larrañaga; *Solanum sisymbriifolium* Lam. forma *albiflorum* Kuntze; *Solanum sisymbriifolium* Lam. forma *lilacinum* Kuntze; *Solanum sisymbriifolium* Lam. var. *brevilobum* Dunal; *Solanum sisymbriifolium* Lam. var. *heracleifolium* Sendtn.; *Solanum sisymbriifolium* Lam. var. *macrocarpum* Kuntze

Herb or subshrub.

Departmental distribution in Paraguay: Alto Paraguay, Central, Cordillera, Ñeembucú, Paraguarí, Presidente Hayes.

Voucher: *J. De Egea & R. Elsam 808* (BM, CTES, FCQ, G, MO).

##### ►*Solanum viarum* Dunal, Prodr. (DC.) 13(1): 240. 1852.

Syn.: *Solanum chloranthum* DC.; nom. illeg.; *Solanum reflexum* auct. non Schrank; *Solanum viridiflorum* Schltdl.

Shrub.

Departmental distribution in Paraguay: Alto Paraná, Alto Paraguay, Caazapá, Central, Cordillera, Guairá, Ñeembucú, Paraguarí, San Pedro.

Voucher: *C. Vogt 171* (CTES, FACEN, FCQ).

##### *Vassobia breviflora (Sendtn.) Hunz., Kurtziana 10: 23. 1977.

Syn.: *Acnistus breviflorus* Sendtn.; *Acnistus breviflorus* Sendtn. forma *glabratus* Hassl.; *Acnistus breviflorus* Sendtn. forma *indutus* Hassl.; *Acnistus breviflorus* Sendtn. var. *glabratus* Sendtn.; *Acnistus bornmulleri* Dammer; *Acnistus galanderi* Dammer; *Acnistus glabratus* (Sendtn.) Dammer; *Acnistus hauthalii* Dammer; *Acnistus lycioides* Dammer; *Acnistus mollis* Dammer; *Acnistus parviflorus* Griseb.; *Acnistus parviflorus* Griseb. var. *arboreus* (Griseb.) Griseb.; *Acnistus schunckii* Dammer; *Acnistus sellowii * Dammer; *Acnistus spinescens* (Sendtn.) Dammer; *Acnistus spinosus* Dammer; *Acnistus ulei* Dammer; *Bassovia pyraster* Dunal; *Bassovia pyraster* Dunalvar. *glabriusculum* Dunal; *Bassovia spina-alba* (Dunal) Griseb.; *Capsicum breviflorum* (Sendtn.) Hunz.; *Capsicum glandulosum* Dunal; *Capsicum pyraster* (Dunal) Kuntze; *Dierbachia breviflora* (Sendtn.) Kuntze; *Dierbachia parviflora* (Sendtn.) Kuntze; *Dierbachia breviflora* (Sendtn.) Sleumer; *Fregirardia spina-alba* Dunal; *Iochroma arboreum* Griseb.

Tree.

Departmental distribution in Paraguay: Alto Paraná, Amambay, Caaguazú, Caazapá, Canindeyú, Central, Cordillera, Guairá, Itapúa, Misiones, Ñeembucú, Paraguarí, San Pedro.

Voucher: *M. Morinigo et al. 4* (BM, CTES, FCQ, G, MO).

#### TYPHACEAE

##### *Typha domingensis Pers., Syn. Pl. [Persoon] 2: 532. 1807.

Syn.: *Typha tenuifolia* Kunth

Herb.

Departmental distribution in Paraguay: Alto Paraguay, Central, Concepción, Cordillera, Ñeembucú, Paraguarí, Presidente Hayes.

Voucher: Cited in Vogt and Mereles 2005: 13.

#### ULMACEAE

##### ►*Phyllostylon rhamnoides* (J.Poiss.) Taub., Oesterr. Bot. Z. 40(11): 409. 1890.

Syn.: *Samaroceltis rhamnoides* J.Poiss.

Tree.

Departmental distribution in Paraguay: Alto Paraguay, Central, Concepción, Ñeembucú, Paraguarí.

Voucher: *M. Vera et al. 235* (BM, FCQ).

#### URTICACEAE

##### *Cecropia pachystachya Trécul, Ann. Sci. Nat., Bot., sér. 3, 8: 80. 1847.

Syn.: *Cecropia adenopus* Mart. ex Miq.; *Cecropia adenopus* Mart. ex Miq. var. *lyratiloba* (Miq.) Hassl.; *Cecropia adenopus* Mart. ex Miq. var. *macrophylla* Hassl.; *Cecropia adenopus* Mart. ex Miq. var. *vulgaris* Hassl., nom. illeg.; *Cecropia catarinensis* Cuatrec.; *Cecropia cinerea* Miq.; *Cecropia glauca* Rojas Acosta; *Cecropia lyratiloba* Miq.

Tree.

Departmental distribution in Paraguay: Alto Paraná, Amambay, Caaguazú, Caazapá, Canindeyú, Central, Cordillera, Guairá, Ñeembucú, Paraguarí.

Voucher: Cited in Vogt and Mereles 2005: 11.

##### *Urera aurantiaca Wedd., Ann. Sci. Nat., Bot., sér. 3, 18: 201. 1852.

Syn.: *Urera aurantiaca* var. *scandens* (D.Parodi) Hassl.; *Urera scandens* D.Parodi

Shrub.

Departmental distribution in Paraguay: Central, Cordillera, Ñeembucú, Paraguarí, Presidente Hayes.

Voucher: *C. Vogt 174* (CTES, FACEN, FCQ).

##### ►*Urera baccifera* (L.) Gaudich. ex Wedd., Ann. Sci. Nat., Bot., sér. 3, 18: 199. 1852.

Syn.: *Urera horrida* (Kunth) Miq.; *Urtica baccifera* L.; *Urtica grandidentata* Liebm., nom. illeg.; *Urtica horrida* Kunth

Shrub.

Departmental distribution in Paraguay: Alto Paraná, Alto Paraguay, Caazapá, Canindeyú, Central, Ñeembucú, Paraguarí, San Pedro.

Voucher: *C. Vogt 156* (CTES, FCQ).

##### ►*Urera caracasana* (Jacq.) Gaudich. ex Griseb., Fl. Brit. W.I. 154. 1859.

Syn.: *Urera jacquini* Wedd.; *Urtica caracasana* Jacq.

Shrub.

Departmental distribution in Paraguay: Alto Paraná, Ñeembucú, Paraguarí.

Voucher: *M. Morinigo et al. 3* (BM, CTES, FCQ).

#### VERBENACEAE

##### ►*Aloysia gratissima* (Gillies & Hook.) Tronc. var. *chacoensis* (Moldenke) Botta, Darwiniana 22: 89. 1979.

Syn.: *Aloysia chacoensis* Moldenke

Shrub.

Departmental distribution in Paraguay: Alto Paraguay, Ñeembucú, Presidente Hayes.

Voucher: *J. De Egea et al. 707* (BM, CTES, FCQ, G, MO).

##### *Aloysia virgata* (Ruiz & Pav.) Pers., Syn. Pl. [Persoon] 2(1): 139. 1809.

Syn.: *Aloysia urticoides* Cham.; *Aloysia virgata* (Ruiz & Pav.) Pers. var. *argutidentata* Moldenke; *Aloysia virgata* (Ruiz & Pav.) Pers. var. *laxa* (Chodat) Moldenke; *Aloysia virgata* (Ruiz & Pav.) Pers. var. *platyphylla* (Briq.) Moldenke, nom. invalid.; *Lippia arborea* Rojas Acosta; *Lippia urticoides* (Cham.) Steud.; *Lippia urticoides* (Cham.) Steud. var. *laxa* Chodat; *Lippia virgata* (Ruiz & Pav.) Steud. var. *elliptica* Briq.; *Lippia virgata* (Ruiz & Pav.) Steud. var. *platyphylla* Briq.; *Priva virgata* (Ruiz & Pav.) Spreng.; *Verbena virgata* Ruiz & Pav.; *Zapania virgata* (Ruiz & Pav.) Poir.

Shrub.

Departmental distribution in Paraguay: Alto Paraná, Alto Paraguay, Amambay, Canindeyú, Central, Concepción, Cordillera, Guairá, Itapúa, Misiones, Ñeembucú, Paraguarí, Presidente Hayes, San Pedro.

Voucher: *M. Peña-Chocarro et al. 2362* (BM, CTES, FCQ, G, MO, SI).

##### ►*Glandularia aristigera* (S.Moore) Tronc., Darwiniana 14(4): 636. 1968.

Syn.: *Glandularia tenuisecta* (Briq.) Small; *Verbena aristigera* S.Moore; *Verbena tenuisecta* Briq.

Herb.

Departmental distribution in Paraguay: Alto Paraguay, Central, Concepción, Cordillera, Guairá, Itapúa, Misiones, Ñeembucú, Paraguarí, Presidente Hayes.

Voucher: *M. Peña-Chocarro et al. 1991* (BM, CTES, FCQ, G, MO).

##### *Glandularia incisa* (Hook.) Tronc., Fl. Prov. Buenos Aires 4(5): 135. 1965.

Syn.: *Verbena incisa* Hook.; *Verbena megapotamica* Spreng. forma *truncatula* Briq.

Herb.

Departmental distribution in Paraguay: Alto Paraguay, Caaguazú, Central, Cordillera, Guairá, Ñeembucú, Paraguarí, Presidente Hayes, San Pedro.

Voucher: *M. Peña-Chocarro et al. 2205* (BM, CTES, FCQ, MO).

##### *Glandularia megapotamica (Spreng.) Cabrera & G.Dawson, Revista Mus. La Plata, Secc. Bot. 5: 357. 1944.

Syn.: *Verbena megapotamica* Spreng.; *Verbena megapotamica* Spreng. var. *tweediana* (Niven) Kuntze; *Verbena phlogiflora* Cham. var. *macilenta* Schauer; *Verbena tweediana* Niven

Herb.

Departmental distribution in Paraguay: Alto Paraná, Alto Paraguay, Amambay, Canindeyú, Cordillera, Guairá, Ñeembucú, Paraguarí.

Voucher: *R. Spichiger et al. 5245* (CTES, G) (http://www.ville-ge.ch/musinfo/bd/cjb/fdp/).

##### ►*Glandularia sessilis* (Cham.) Tronc., Fl. Ilustr. Entre Ríos 5: 247. 1979.

Syn.: *Verbena morongii* Britton; *Verbena schulzii* Moldenke; *Verbena sessilis* (Cham.) Kuntze; *Verbena stellarioides* Cham. var. *sessilis* Cham.

Herb.

Departmental distribution in Paraguay: Alto Paraguay, Caazapá, Concepción, Itapúa, Ñeembucú, Paraguarí, Presidente Hayes.

Voucher: *M. Peña-Chocarro et al. 2318* (BM, CTES, FCQ, PY).

##### *Glandularia tomophylla* (Briq.) P.Peralta, Darwiniana 45: 241. 2007.

Syn.: *Verbena tomophylla* Briq.

Herb.

Departmental distribution in Paraguay: Central, Ñeembucú.

Voucher: Cited in Zuloaga et al. 2008: 3113.

##### *Lantana balansae* Briq., Annuaire Conserv. Jard. Bot. Genève 7-8: 300. 1904.

Syn.: *Lantana balansae* Briq. forma *albiflora* Osten & Moldenke; *Lantana balansae* Briq. var. *peduncularis* Briq.

Shrub.

Departmental distribution in Paraguay: Alto Paraguay, Caaguazú, Caazapá, Central, Concepción, Cordillera, Ñeembucú, Presidente Hayes.

Voucher: Cited in Zuloaga et al. 2008: 3119.

##### *Lantana fucata* Lindl., Bot. Reg. 10: 788 (t. 798). 1824.

Syn.: *Camara lilacina* (Desf.) Kuntze var. *parvifolia* Kuntze; *Lantana balansae* Briq. var. *hatschbachii* Moldenke; *Lantana czermakii* Briq.; *Lantana lilacina* Desf.; *Lantana lilacina* Desf. var. *media* (Kuntze) Briq.; *Lantana lilacina* Desf. var. *parvifolia* (Kuntze) Briq.

Shrub.

Departmental distribution in Paraguay: Alto Paraná, Alto Paraguay, Amambay, Central, Concepción, Cordillera, Guairá, Itapúa, Misiones, Ñeembucú, Paraguarí, Presidente Hayes.

Voucher: Cited in Zuloaga et al. 2008: 3120.

##### ►*Lantana montevidensis* (Spreng.) Briq., Annuaire Conserv. Jard. Bot. Genève 7-8: 301. 1904.

Syn.: *Camara montevidensis* (Spreng.) Kuntze; *Camara sellowiana* (Link & Otto) Kuntze; *Lantana sellowiana* Link & Otto; *Lippia montevidensis* Spreng.

Shrub.

Departmental distribution in Paraguay: Alto Paraná, Caaguazú, Misiones, Ñeembucú.

Voucher: *J. De Egea et al. 643* (BM, FCQ, MO).

##### *Lantana trifolia L., Sp. Pl. 2: 626. 1753.

Syn.: *Camara trifolia* (L.) Kuntze; *Lantana celtidifolia* Kunth; *Lantana fiebrigii* Hayek; *Lantana trifolia* L. forma *albiflora* Moldenke; *Lantana trifolia* L. var. *rigidiuscula* Briq.; *Lantana trifolia* L. var. *vulgata* Briq.

Shrub.

Departmental distribution in Paraguay: Alto Paraguay, Amambay, Caazapá, Central, Concepción, Cordillera, Guairá, Itapúa, Misiones, Ñeembucú, Paraguarí, San Pedro.

Voucher: *R. Spichiger et al. 5254* (CTES, G, MO) (http://www.ville-ge.ch/musinfo/bd/cjb/fdp/).

##### *Lippia alba* (Mill.) N.E.Br. ex Britton & P.Wilson, Sci. Surv. Porto Rico & Virgin Islands 6: 141. 1925.

Syn.: *Lantana alba* Mill.; *Lantana geminata* (Kunth) Spreng.; *Lippia citrata* Cham.; *Lippia geminata* Kunth; *Lippia geminata* Kunthvar. *microphylla* Griseb.; *Lippia globiflora* (L’Hér.) Kuntze var. *geminata* (Kunth) Kuntze; *Lippia globiflora* (L’Hér.) Kuntze var. *normalis* Kuntze

Shrub.

Departmental distribution in Paraguay: Alto Paraná, Alto Paraguay, Amambay, Caaguazú, Caazapá, Canindeyú, Central, Concepción, Guairá, Itapúa, Ñeembucú, Presidente Hayes.

Voucher: Cited in Zuloaga et al. 2008: 3123.

##### *Lippia angustifolia* Cham., Linnaea 7: 377. 1832.

Syn.: *Lippia bothriura* Briq.

Subshrub.

Departmental distribution in Paraguay: Caaguazú, Canindeyú, Central, Concepción, Cordillera, Misiones, Ñeembucú, San Pedro.

Voucher: *S. Keel & L. Spinzi 1473* (FCQ).

##### *Lippia asperrima* Cham., Linnaea 7: 215. 1832.

Syn.: *Lippia asperrima* Cham. var. *rotundata* Moldenke; *Lippia asperrima* Cham. forma *angustifolia* Moldenke; *Lippia contermina* Briq. var. *contermina* Briq.; *Lippia contermina* Briq. var. *hirsuta* Moldenke; *Lippia phaeocephala* Briq.; *Lippia rodriguezii* Moldenke; *Lippia turnerifolia* Cham. var. *camporum* Griseb.

Subshrub.

Departmental distribution in Paraguay: Alto Paraná, Alto Paraguay, Caaguazú, Caazapá, Central, Cordillera, Guairá, Itapúa, Misiones, Ñeembucú, Paraguarí, Presidente Hayes.

Voucher: *T. Meyer 16038* (NY) (http://www2.darwin.edu.ar/).

##### *Lippia lippioides (Cham.) Rusby, Mem. Torrey Bot. Club 6: 106. 1896.

Syn.: *Camara lippioides* (Cham.) Kuntze; *Lantana chamissonis* (D.Dietr.) Benth. & Hook.; *Lantana chamissonis* (Schauer) Briq., nom. illeg.; *Lippia chamissonis* D.Dietr., nom. superfl.; *Riedelia lippioides* Cham.

Subshrub.

Departmental distribution in Paraguay: Alto Paraná, Alto Paraguay, Caazapá, Central, Cordillera, Guairá, Misiones, Ñeembucú, Paraguarí, San Pedro.

Voucher: *R. Spichiger et al. 5258* (CTES, G, MO) (http://www.ville-ge.ch/musinfo/bd/cjb/fdp/, as *Lantana chamissonis*).

##### ►*Phyla canescens* (Kunth) Greene, Pittonia 4: 48. 1899.

Syn.: *Lippia canescens* Kunth; *Lippia litoralis* Phil.; *Lippia nodiflora* (L.) Michx. var. *minor* Gillies & Hook.; *Lippia nodiflora* (L.) Michx. var. *pusilla* Briq.; *Lippia nodiflora* (L.) Michx. var. *rosea* (D.Don) Munz; *Lippia uncinuligera* Nees ex Walp.; *Phyla nodiflora* (L.) Greene var. *canescens* (Kunth) Moldenke; *Phyla nodiflora* (L.) Greene var. *pusilla* Moldenke; *Phyla nodiflora* (L.) Greene var. *rosea* (D.Don) Moldenke; *Zapania canescens* Gilbert; *Zapania nodiflora* (L.) Lam. var. *rosea* D.Don

Herb.

Departmental distribution in Paraguay: Alto Paraguay, Ñeembucú, Presidente Hayes.

Voucher: *M. Peña-Chocarro et al. 2258* (BM, CTES, FCQ, G, MO).

##### *Phyla fruticosa (Mill.)K.Kenn. ex Wunderlin & B.F.Hansen, Bot. Explor. (Florida) 3: 38. 2003.

Syn.: *Phyla strigulosa* (M.Martens & Galeotti) Moldenke

Herb.

Departmental distribution in Paraguay: Alto Paraguay, Boquerón, Central, Cordillera, Ñeembucú, Paraguarí, Presidente Hayes.

Voucher: *T. Meyer 16163* (LIL) (http://www.tropicos.org/).

##### *Stachytarpheta cayennensis (Rich.) Vahl, Enum. Pl. [Vahl] 1: 208. 1804.

Syn.: *Stachytarpheta australis* Moldenke; *Stachytarpheta cayennensis* (Rich) Vahl forma *alba* Moldenke ex Tronc. & Botta; *Stachytarpheta cayennensis* (Rich) Vahl forma *albiflora* Moldenke; *Stachytarpheta cayennensis* (Rich) Vahl var. *virescens* Briq.; *Stachytarpheta dichotoma* (Ruiz & Pav.) Vahl; *Stachytarpheta maximiliani* Schauer var. *ciliaris* Moldenke; *Stachytarpheta patens* Moldenke; *Verbena cayennensis* Rich.; *Verbena dichotoma* Ruiz & Pav.

Herb.

Departmental distribution in Paraguay: Alto Paraná, Alto Paraguay, Amambay, Caaguazú, Caazapá, Canindeyú, Central, Concepción, Cordillera, Guairá, Itapúa, Misiones, Ñeembucú, Paraguarí, Presidente Hayes, San Pedro.

Voucher: *M. Peña-Chocarro et al. 1999* (BM, CTES, FCQ, MO).

##### *Verbena bonariensis* L., Sp. Pl. 1: 30. 1753.

Syn.: *Verbena bonariensis* L. forma *albiflora* Moldenke; *Verbena elongata* Salisb.; *Verbena incompta* P.W.Michael; *Verbena trichotoma* Moench

Herb.

Departmental distribution in Paraguay: Alto Paraná, Caazapá, Central, Guairá, Itapúa, Misiones, Ñeembucú, Paraguarí, San Pedro.

Voucher: *A.G. Schulz 7808* (CTES) (cited in O’Leary et al. 2007: 583).

##### *Verbena ephedroides* Cham., Linnaea 7: 261. 1832.

Syn.: *Verbena ephedroides* Cham. var. *entreriensis* Tronc.

Herb.

Departmental distribution in Paraguay: Ñeembucú.

Voucher: *S. Keel & L. Spinzi 1468* (FCQ).

##### *Verbena litoralis* Kunth var. *litoralis*, Nov. Gen. Sp. (quarto ed.) 2(7): 276. 1818.

Syn.: *Verbena affinis* M.Martens & Galeotti; *Verbena approximata* Briq.; *Verbena bonariensis* L. var. *litoralis* (Kunth) Gillies & Hook.; *Verbena caracasana* Kunth; *Verbena cordobensis* Briq.; *Verbena integrifolia* Sessé & Moc.; *Verbena integrifolia* forma *albiflora* Moldenke; *Verbena litoralis* Kunth forma *albiflora* (Moldenke) Moldenke; *Verbena litoralis* Kunth forma *angustifolia* Chodat; *Verbena litoralis* Kunth var. *albiflora* Moldenke; *Verbena litoralis* Kunth var. *caracasana* (Kunth) Briq.; *Verbena litoralis* Kunth var. *glabrior* Benth.; *Verbena litoralis* Kunth var. *leptostachya* Schauer; *Verbena litoralis* Kunth var. *pycnostachya* Schauer, nom. illeg.; *Verbena nudiflora* Nutt. ex Turcz.

Herb.

Departmental distribution in Paraguay: Alto Paraná, Amambay, Caaguazú, Caazapá, Canindeyú, Central, Concepción, Cordillera, Guairá, Itapúa, Misiones, Ñeembucú, Paraguarí, San Pedro.

Voucher: *M. Peña-Chocarro et al. 2206* (BM, FCQ).

##### *Verbena litoralis* Kunth var. *brevibracteata* (Kuntze) N.O’Leary, Ann. Missouri Bot. Gard. 94: 598. 2007.

Syn.: *Verbena bonariensis* L. var. *brevibracteata* Kuntze; *Verbena brasiliensis* Vell.; *Verbena brasiliensis* Vell. var. *subglabrata* Moldenke; *Verbena chacensis* Moldenke; *Verbena hanseni* Greene; *Verbena intermedia* Gillies & Hook. ex Hook. forma *glabrescens* Hauman; *Verbena litoralis* Kunth forma *congesta* (Moldenke) Moldenke; *Verbena litoralis* Kunth var. *brasiliensis* (Vell.) Briq., nom. inval.; *Verbena litoralis* Kunth var. *brasiliensis* (Vell.) Munir; *Verbena litoralis* Kunth var. *congesta* Moldenke; *Verbena paucifolia* Turcz.; *Verbena quadrangularis* Vell.

Herb.

Departmental distribution in Paraguay: Alto Paraguay, Central, Ñeembucú, San Pedro.

Voucher: Cited in Zuloaga et al. 2008: 3142.

#### VIOLACEAE

##### *Hybanthus bigibbosus (A.St.-Hil.) Hassl., Bull. Soc. Bot. Genève, sér. 2, 1: 213. 1909.

Syn.: *Hybanthus bigibbosus* (A.St.-Hil.) Hassl. var. *paraguariensis* (Chodat) Hassl.; *Ionidium bigibbosum* A.St.-Hil.; *Ionidium bigibbosum* A.St.-Hil. var. *paraguarense* Chodat; *Solea bigibbosa* (A.St.-Hil.) Spreng.

Shrub.

Departmental distribution in Paraguay: Alto Paraná, Amambay, Caazapá, Canindeyú, Central, Cordillera, Itapúa, Ñeembucú, Paraguarí, San Pedro.

Voucher: *R. Spichiger et al. 5322* (G) (http://www.ville-ge.ch/musinfo/bd/cjb/fdp/).

##### *Hybanthus communis (A.St.-Hil.) Taub., Nat. Pflanzenfam. 6(119): 333. 1895.

Syn.: *Hybanthus communis* (A.St.-Hil.) Taub.forma *circaeoides* (Chodat) Schulze-Menz; *Hybanthus communis* (A.St.-Hil.) Taub.forma *glabrifolius* (Chodat) Schulze-Menz; *Hybanthus communis* (A.St.-Hil.) Taub.var. *circaeoides* (Chodat) Hassl.; *Hybanthus communis* (A.St.-Hil.) Taub.var. *glabrifolius* (Chodat) Hassl.; *Hybanthus guaraniticus* (A.St.-Hil.) Baill.; *Ionidium commune* A.St.-Hil.; *Ionidium commune* A.St.-Hil. var. *circaeoides* Chodat; *Ionidium commune* A.St.-Hil. var. *glabrifolium* Chodat; *Ionidium guaraniticum* A.St.-Hil.; *Ionidium sylvaticum* A.St.-Hil.; *Solea communis* (A.St.-Hil.) Spreng.; *Solea guaranitica* (A.St.-Hil.) Spreng.; *Solea sylvatica* (A.St.-Hil.) Spreng.

Herb.

Departmental distribution in Paraguay: Alto Paraná, Alto Paraguay, Amambay, Caazapá, Canindeyú, Cordillera, Guairá, Itapúa, Ñeembucú, Paraguarí, San Pedro.

Voucher: *R. Spichiger et al. 5331* (G) (http://www.ville-ge.ch/musinfo/bd/cjb/fdp/).

##### ►*Hybanthus parviflorus* (Mutis ex.L.f.) Baill., Traité Bot. Méd. Phan. 2(1): 841. 1884.

Syn.: *Calceolaria bangii* Rusby; *Hybanthus glutinosus* (Vent.) Taub.; *Hybanthus parviflorus* (Mutis ex L.f.) Baill. var. *argentinensis* Sparre; *Hybanthus parviflorus* (Mutis ex L.f.) Baill. var. *bangii* (Rusby) Sparre; *Hybanthus parviflorus* (Mutis ex L.f.) Baill. var. *glutinosus* (Vent.) Hassl.; *Ionidium glutinosum* Vent.; *Ionidium glutinosum* Vent. var. *paraguayense* Chodat; *Ionidium microphyllum* Willd. ex Roem. & Schult.; *Ionidium parviflorum* (L.f.) Vent.; *Viola parviflora* L.f.; *Viola venezuelensis* Steyerm.

Herb or subshrub.

Departmental distribution in Paraguay: Alto Paraguay, Central, Cordillera, Ñeembucú, Presidente Hayes, San Pedro.

Voucher: *M. Peña-Chocarro et al. 2249* (BM, CTES, FCQ).

#### VITACEAE

##### ►*Cissus palmata* Poir., Encycl. (Lamarck) Suppl. 1. 107. 1810.

Syn.: *Cissus bonariesis* Hook. & Arn.; *Cissus gibertii* (Baker) Planch.; *Cissus palmata* Poir. var. *balansana* Planch.; *Cissus paraguayensis* Planch.; *Vitis bakeri* Herter; *Vitis gibertii* Baker; *Vitis palmata* (Poir.) Baker

Climber.

Departmental distribution in Paraguay: Alto Paraguay, Central, Concepción, Itapúa, Misiones, Ñeembucú, Presidente Hayes, San Pedro.

Voucher: *M. Peña-Chocarro et al. 2367* (FCQ).

##### *Cissus spinosa* Cambess., Fl. Bras. Merid. (quarto ed.) 1: 345.1828.

Syn.: *Cissus hasslerianus* Chodat

Climber.

Departmental distribution in Paraguay: Alto Paraguay, Central, Concepción, Ñeembucú, Presidente Hayes.

Voucher: *Schultz 7906* (CTES) (cited in Lombardi 2000: 163).

##### *Cissus verticillata (L.) Nicolson & C.E.Jarvis, Taxon 33: 727. 1984.

Syn.: *Cissus canescens* Lam.; *Cissus compressicaulis* Ruiz & Pav.; *Cissus ovata* Lam.; *Cissus sicyoides* L.; *Cissus sicyoides* L. forma *apensis* Chodat & Hassl.; *Cissus sicyoides* L. forma *aristolochiifolia* Planch.; *Cissus sicyoides* L. forma *balansae* Planch.; *Cissus sicyoides* L. forma *foliata* Chodat & Hassl.; *Cissus sicyoides* L. forma *marmorata* Chodat & Hassl.; *Cissus sicyoides* L . forma *palmata* Hassl.; *Cissus sicyoides* L. forma *paraguayensis* Chodat & Hassl.; *Cissus smilacina* Kunth; *Cissus tamoides* Cambess.; *Cissus tucumana* Suess.; *Cissus umbrosa* Kunth; *Cissus verticillata* (L.) Nicholson & C.E.Jarvis subsp. *lacinata* (Baker) Lombarda; *Viscum verticillatum* L.; *Vitis sicyoides* (L.) Miq.; *Vitis sicyoides* (L.) Miq. forma *lobata* Baker

Climber.

Departmental distribution in Paraguay: Alto Paraguay, Alto Paraná, Amambay, Caaguazú, Canindeyú, Central, Concepción, Cordillera, Guairá, Itapúa Misiones, Ñeembucú, Paraguarí, Presidente Hayes, San Pedro.

Voucher: *M. Vera 218* (FCQ).

#### XYRIDACEAE

##### ►*Xyris jupicai* Rich., Actes Soc. Hist. Nat. Paris 1: 106. 1792.

Syn.: *Xyris jupicai* Rich. var. *brachylepis* Malme; *Xyris jupicai* Rich. var. *major* (Mart.) L.B.Sm. & Downs; *Xyris laxifolia* Mart. var. *minor* Mart.; *Xyris macrocephala* Vahl; *Xyris macrocephala* Vahl var. *minor* (Mart.) L.A.Nilsson; *Xyris sellowiana* Kunth forma *humilis* Kunth

Herb.

Departmental distribution in Paraguay: Amambay, Caaguazú, Canindeyú, Cordillera, Guairá, Ñeembucú.

Voucher: *F. Gonzalez-Parini et al. 1683* (FCQ).

## Appendix 1

Taxon checklist, habitat description and departmental distribution of vascular plants in Paraguay (doi: 10.3897/phytokeys.9.2279.app) File format: EXCEL spreadsheet.
